# Mathematical modeling of SARS-CoV-2 variant substitutions in European countries: transmission dynamics and epidemiological insights

**DOI:** 10.3389/fpubh.2024.1339267

**Published:** 2024-05-15

**Authors:** Víctor López de Rioja, Aida Perramon-Malavez, Sergio Alonso, Cristina Andrés, Andrés Antón, Antoni E. Bordoy, Jordi Càmara, Pere-Joan Cardona, Martí Català, Daniel López, Sara Martí, Elisa Martró, Verónica Saludes, Clara Prats, Enrique Alvarez-Lacalle

**Affiliations:** ^1^Computational Biology and Complex Systems Group, Department of Physics, Universitat Politècnica de Catalunya, Barcelona, Spain; ^2^Microbiology Department, Vall D’Hebron Hospital Universitari, Vall D’Hebron Institut de Recerca, Vall D’Hebron Barcelona Hospital Campus, Barcelona, Spain; ^3^Biomedical Research Networking Center in Infectious Diseases, Instituto de Salud Carlos III (ISCIII), Madrid, Spain; ^4^Microbiology Department, Northern Metropolitan Clinical Laboratory, Germans Trias i Pujol University Hospital and Research Institute, Badalona, Spain; ^5^Microbiology Department, Hospital Universitari de Bellvitge, IDIBELL-UB, L’Hospitalet de Llobregat, Barcelona, Spain; ^6^Research Network for Respiratory Diseases (CIBERES), Instituto de Salud Carlos III (ISCIII), Madrid, Spain; ^7^Centre for Statistics in Medicine, Nuffield Department of Orthopaedics, Rheumatology and Musculoskeletal Sciences, University of Oxford, Oxford, United Kingdom; ^8^Biomedical Research Center Network for Epidemiology and Public Health, Instituto de Salud Carlos III (ISCIII), Madrid, Spain

**Keywords:** SARS-COV-2 variants, transmissibility, vaccination rates, epidemiological timing, effective reproduction number, epidemiological modeling, variant substitution, genomic surveillance

## Abstract

**Background:**

Countries across Europe have faced similar evolutions of SARS-CoV-2 variants of concern, including the Alpha, Delta, and Omicron variants.

**Materials and methods:**

We used data from GISAID and applied a robust, automated mathematical substitution model to study the dynamics of COVID-19 variants in Europe over a period of more than 2 years, from late 2020 to early 2023. This model identifies variant substitution patterns and distinguishes between residual and dominant behavior. We used weekly sequencing data from 19 European countries to estimate the increase in transmissibility (Δβ)  between consecutive SARS-CoV-2 variants. In addition, we focused on large countries with separate regional outbreaks and complex scenarios of multiple competing variants.

**Results:**

Our model accurately reproduced the observed substitution patterns between the Alpha, Delta, and Omicron major variants. We estimated the daily variant prevalence and calculated Δβ between variants, revealing that: (i) Δβ increased progressively from the Alpha to the Omicron variant; (ii) Δβ showed a high degree of variability within Omicron variants; (iii) a higher Δβ was associated with a later emergence of the variant within a country; (iv) a higher degree of immunization of the population against previous variants was associated with a higher Δβ for the Delta variant; (v) larger countries exhibited smaller Δβ, suggesting regionally diverse outbreaks within the same country; and finally (vi) the model reliably captures the dynamics of competing variants, even in complex scenarios.

**Conclusion:**

The use of mathematical models allows for precise and reliable estimation of daily cases of each variant. By quantifying Δβ, we have tracked the spread of the different variants across Europe, highlighting a robust increase in transmissibility trend from Alpha to Omicron. Additionally, we have shown that the geographical characteristics of a country, as well as the timing of new variant entrances, can explain some of the observed differences in variant substitution dynamics across countries.

## 1 Introduction

Since the emergence of the severe acute respiratory syndrome coronavirus 2 (SARS-CoV-2) in late 2019, the virus responsible for the Coronavirus disease 2019 (COVID-19) pandemic has evolved significantly, leading to the emergence of different viral variants responsible for successive waves of infection. To date, over 750 million confirmed cases and more than 6.5 million deaths have been documented worldwide (1). In particular, several European countries have experienced waves of infection from these variants, highlighting the need for vigilant surveillance and a deep understanding of their transmission dynamics.

Among these variants, some have been designated by the World Health Organization (WHO) as Variants of Concern (VOCs) or Variants of Interest (VOIs) due to their potential impact on transmissibility, disease severity, and the efficacy of diagnostics and vaccines (2, 3). The VOCs, including Alpha, Beta, Gamma, Delta, and various Omicron variants exhibit distinct characteristics affecting their transmissibility and potential for immune evasion.

The evolution of SARS-CoV-2 has resulted in significant genomic changes that have had a substantial impact on its spread across Europe (4). Initially dominated by various lineages such as B.1.177, B.1.160, and B.1.258, the introduction of the more transmissible Alpha variant changed the landscape in European countries, leading to an increase in case numbers and hospitalizations (5–7). This was followed by the Delta variant, which overtook Alpha and challenged public health efforts due to its high transmissibility, potential for increased disease severity, and partial immune evasion (8–10). Subsequently, Omicron and its different subvariants (BA.1, BA.2, BA.5, BQ.1, and XBB.1) have emerged sequentially, replacing each other with unique substitution patterns (11–13).

Understanding the dynamics of variant substitution and changes in transmissibility is essential to design effective public health strategies. In this study, we explore these substitution dynamics across Europe using a mathematical model to unravel similarities and differences between countries. We aimed to have a model that can process large datasets and automatically detect variant behavior, providing a valuable tool for understanding the historical evolution of the pandemic and plausible short-to-mid-term scenarios for a large number of countries.

We estimate the daily prevalence of each SARS-CoV-2 variant by country and calculate the increase in transmissibility, Δβ, for each substitution and country. We also examine how factors such as initial emergence day and country surface area affect variant behavior. Using corrected daily number of cases, we assess shifts in the effective reproduction number, $R_{t}$ , associated with emerging variants. Special attention is given to large countries and scenarios where multiple variants are in strong competition, providing nuanced insights into complex dynamics. To our knowledge, while some studies have conducted similar analyses on a large database (14–16), none have obtained comprehensive results across Europe.

## 2 Materials and methods

Data on the weekly number of sequenced variants and the number of vaccinated individuals in European countries were obtained from the European Center for Disease Prevention and Control (ECDC) database. In contrast, daily COVID-19 case numbers for each country were obtained from the WHO database, as the ECDC only provides data on a weekly basis. Additionally, demographic, geographic, and statistical data were obtained directly from the official European statistical website. See Data Availability Statement for more information.

### 2.1 SARS-CoV-2 variants data bases

Our study was based on official variant sequencing data for each European country, obtained from the ECDC website. These data, available on a weekly basis (17), were derived from two primary sources: the Global Initiative on Sharing All Influenza Data (GISAID) (18) and the European Surveillance System (TESSy). Throughout the COVID-19 pandemic, both databases have provided up-to-date case numbers, variant distributions across Europe, and other essential information such as vaccination rates. Both databases are comprehensively represented in Supplementary Tables 1A,B. However, depending on the data source, the number of samples per country and date varied considerably. We found significant inconsistencies suggesting that the epidemiologic surveillance centers reported exclusively to only one source, or data appeared to be duplicated, indicating that some countries reported the same sequenced samples to both GISAID and TESSy. Differences in data processing and quality control procedures between the two databases may also contribute to these discrepancies. See Supplementary material S1 for a detailed description of both data sources.

Given these issues, we decided to use only GISAID data in the main manuscript and leave the TESSy analysis for the Supplementary material. There were two main reasons for this decision: GISAID has been the most widely used source in the literature, and it provides data that allowed us to analyze the first significant substitution (*pre-Alpha* vs. Alpha), which was not possible with TESSy data, as it provides sequenced variant data primarily since early 2021. By relying exclusively on GISAID, we increased the rigor of our study, albeit at the cost of excluding some countries from our analysis. We explored a combination of the two sources to test the robustness of our results and to provide results for more countries in Supplementary material S11.

### 2.2 Data: GISAID database overview

Data from GISAID source in ECDC database on variant sequencing began in early 2020 for most countries, initially presenting a limited number of samples and high variability. Regarding variant assignation, many of these samples are labeled as “Other” in the ECDC database as they predate the definition of VOCs and VOIs by the health authorities at the time of their occurrence.^1^ As mentioned in before, various lineages circulated in Europe before the Alpha VOC took hold. Among these, B.1.177 was dominant in many countries, with significant proportions of other lineages, such as B.1.160, and B.1.258, also present. These, along with smaller proportions of additional variants, will be referred to hereafter as the *pre-Alpha* set to maintain consistency with the source. As the pandemic progressed across Europe, the number of samples sequenced increased in all countries, with peaks during the waves driven primarily by the Alpha, Delta, and Omicron VOCs. From the period of Alpha dominance to the present, our “Other” category refers to a mix of residual variants found in different countries, not exclusively the *pre-Alpha* set mentioned above, and not exclusively the “Other” category from the GISAID source.

Data from some countries presented issues which did not allow us to conduct the analysis. Some countries, including Hungary, Liechtenstein, and Malta, were excluded from our analysis due to low sampling rates and significant variability in their data. Additionally, we decided not to use the GISAID data from countries with different problems in the datasets such as Austria, Cyprus, Estonia, Greece, Iceland, Luxembourg, Portugal, and Slovakia. Given the different nature of the problems, we refer the reader to the Supplementary material S2 for an explanation of each country. In the end, our study included data from 19 countries: Belgium, Bulgaria, Croatia, Czechia, Denmark, Finland, France, Germany, Ireland, Italy, Latvia, Lithuania, the Netherlands, Norway, Poland, Romania, Slovenia, Spain, and Sweden.

### 2.3 Substitution model and estimation of cases per variant

In this study, we introduce an enhanced version of a previously employed substitution model (19). Our updated model not only facilitates the analysis of substitution patterns among various SARS-CoV-2 variants across different countries but also automatically determines the behavior of each variant during the substitution process. It distinguishes between variants that are major drivers of substitution and those that have a marginal presence. Additionally, the model calculates the increase in transmissibility, Δβ, for each variant and substitution process. A brief explanation of the model (mathematical and computational) is given below. For a more detailed discussion, refer to Supplementary material S3.

The simplest scenario for a substitution model involves only two variants: the initially dominant one (1) and the one that will ultimately prevail (2). We assume that both variants evolve according to an exponential dynamic equation $N_{i}=N_{i,0}\;e^{\beta_{i}t}$, and that the mean time between infection and maximum infectivity of individuals τ is fixed for both variants. Thus, we can relate the exponent $\beta_{i}$ to the transmissibility and thus to the effective reproduction number as $\beta_{i}=\frac{\ln R_{t}{\;}_{i}}{\tau }$. Since τ is fixed, the increase in transmissibility parameter,  $\Delta \beta =\beta_{2}-\beta_{1}$ , remains constant, although $\beta_{1}$ and $\beta_{2}$ may change with time. In this case, we derive the following equation for the fraction of the emerging variant during the substitution period:


(1) $$p_{2}\left(t\right)=\frac{\xi_{0}e^{\Delta \beta \;t}}{1+\xi_{0}e^{\Delta \beta \;t}},$$

where $\xi_{0}$ is the initial ratio between variant 2 (the future dominant variant) and variant 1 (the previously dominant variant). For the latter variant, which is in decline, $p_{1}\left(t\right)=1-p_{2}\left(t\right)$.

These equations are useful when we aim to compare Δβ between only two variants. However, in general, they may not adequately capture the dynamics of the virus within a country when multiple variants circulate simultaneously. Results from this simplified approach are presented in Supplementary material S7, while a comprehensive analysis of all Δβ values can be found in the Supplementary material S12.

When three or more variants are involved, additional equations are included to account for the increased complexity. Assume that N variants are competing to dominate the national viral landscape. For each of these competing variants, i, we can rewrite the previous (Equation 1) as:


(2) $$p_{i}\left(t\right)=\frac{\xi_{0,i}e^{\Delta \beta_{i}t}}{1+\sum_{\begin{array}{c} j=1\ldots i\ldots N\\ \; \end{array}}\xi_{0,j}e^{\Delta \beta_{j}t.}}$$

Again, the fraction corresponding to the descending variant can be estimated as $p_{desc}\left(t\right)=1-\underset{j=1\ldots i\ldots N}{\sum }p_{j}\left(t\right).$

But, as explained before, not all variants show a peak behavior. Some variants present different trends: remain constant, increase linearly, decrease… In such cases, we can simulate each shape of the different variants competing for dominance, as before, but excluding those considered as residuals. Now, consider $N^{\prime }$ variants that behave as a peak or wave (those competing to dominate the national viral landscape) and $M^{\prime }$ variants that can be considered as residual. The total number of variants is now $N^{\prime }+M^{\prime }+1$ (to account for the one that would decline). Then, if variant k is one of the residual variants in the substitution, i.e., $k=1,\ldots ,M^{\prime }$, the dynamics of the system can be written as a function of the emerging variants:


(3) $$p_{i}\left(t\right)=\frac{\xi_{0,i}e^{\Delta \beta_{i}t}}{1+\sum_{\begin{array}{c} j=1\ldots i\ldots N\\ k\neq j \end{array}}\xi_{0,j}e^{\Delta \beta_{j}t}},$$

the residual variants:


(4) $$p_{k}\left(t\right)=\xi_{0,k}+\Delta \beta_{k}t,$$

and the descendent variant:


(5) $$p_{desc}\left(t\right)=1-\sum_{\begin{array}{c} j=1\ldots i\ldots N\\ k\neq j \end{array}}p_{j}\left(t\right).$$

Where $\xi_{0,i}$ and $\Delta \beta_{i}$ represent the initial ratio and the increase in transmissibility between the variant i and all other competing variants, respectively, $i=1,\ldots ,N^{\prime }$ and $k=1,\ldots ,M^{\prime }$.

Thus, Equation 2 is replaced by the previous system of equations, where Equation 3 explains the behavior of variants with significant relative importance compared to others, Equation 4 describes the response of the residual variants, and finally, Equation 5 addresses the variant of decreasing significance in the national viral landscape. These mathematical equations are employed in our computational approach to model the dynamics of SARS-CoV-2 variants, adapting the model to each substitution and country. The algorithm collects and categorizes weekly samples of each variant, defines a time window for variant substitution, and automatically determines the number of variants involved. It then identifies the residual variants and optimizes the fit for those that exhibit substitution behavior. This iterative process continues until a final substitution model is established, providing detailed statistics such as daily percentages, confidence intervals, and key metrics related to the substitution process, such as Δβ. Furthermore, as it extracts the estimated daily percentages of each variant in each substitution process, we can also estimate the daily cases of COVID-19 associated with a particular variant.

In the Supplementary material we use a variety of mathematical approaches to test its robustness. Besides the nonlinear regression mentioned above, we use a Monte Carlo simulation algorithm and a weighted nonlinear regression. Both are explained in more detail in the Supplementary material S4, and results can be found in the Supplementary Table S8.

### 2.4 Effective reproduction number analysis

We employ an empirical method for estimating the time-dependent effective reproduction number ($R_{t}$), a critical parameter for quantifying disease transmissibility over time. $R_{t}$ indicates the average number of people that a single infected individual will transmit the disease to at a specific point in time. It provides insights into whether an epidemic is growing or declining. If $R_{t}>1$, each existing infection is expected to generate more than one new infection, implying that the spread is increasing. Conversely, when $R_{t}<1$, each existing infection causes less than one new infection, decreasing the spread. We used the relationship between $R_{t}$ and variant substitution processes to examine how the emergence of new variants affects the total number of cases. We use the definition of a previous empirical model (20), but with certain modifications (21) to account for irregularities in daily reported cases due to weekends or holidays.

Clear daily patterns led us to assign a global weight, $W_{j,d}$, to each weekday (d), depending on the specific country (j):


(6) $$W_{j,d}=\frac{1}{N_{w}}\sum_{t\in j,d}w\left(t\right),$$

where $N_{w}$ is the number of weeks in the series, and w(t) is the ratio of the day. This pattern takes into account weekends and, for example, December 25th, or January 1st.

Using these weights, we generate a corrected series of new cases for each country. Let us denote $n_{j}\left(t\right)$ as the number of new cases in a country j for a given day t. We then construct the corrected series of new cases $C_{j}\left(t\right)$ as follows:


(7) $$C_{j}\left(t\right)=\frac{n_{j}\left(t\right)}{W_{j,d}},$$

where d is the weekday associated with day t. Finally, with these new values, we can estimate the $R_{t}$ as the ratio:


(8) $$R_{t}\left(t\right)=\frac{C_{j}\left(t\right)+C_{j}\left(t-1\right)+C_{j}\left(t-2\right)}{C_{j}\left(t-5\right)+C_{j}\left(t-6\right)+C_{j}\left(t-7\right)}.$$

## 3 Results

In this section, we present the results using the primarily selected database (GISAID) and the nonlinear method explained in Sections 2.3, 2.4 (further information in Supplementary materials S3, S4). The results of the other mathematical approaches mentioned in the previous section can be found in Supplementary Table S8, achieving similar results and conclusions.

### 3.1 Temporal evolution of SARS-CoV-2 variants and effective reproduction number

Here, for the temporal evolution of SARS-CoV-2 variants in Europe, we use Spain as a case study. The same analyses for other countries are presented in the Supplementary material. The study yields three types of results: (i) the evolution of the VOC substitutions Alpha, Delta, and Omicron in 19 European countries; (ii) the evolution of the six major substitutions (Alpha, Delta, and the Omicron variants BA.1, BA.2, BA.5, and BQ.1) in 18 European countries;^2^ and (iii) the dynamics of the dominant variants in each substitution process in these 18 countries. We present the first and second results in the main manuscript and relegate the third to Supplementary material S7.

**Figure 1:**
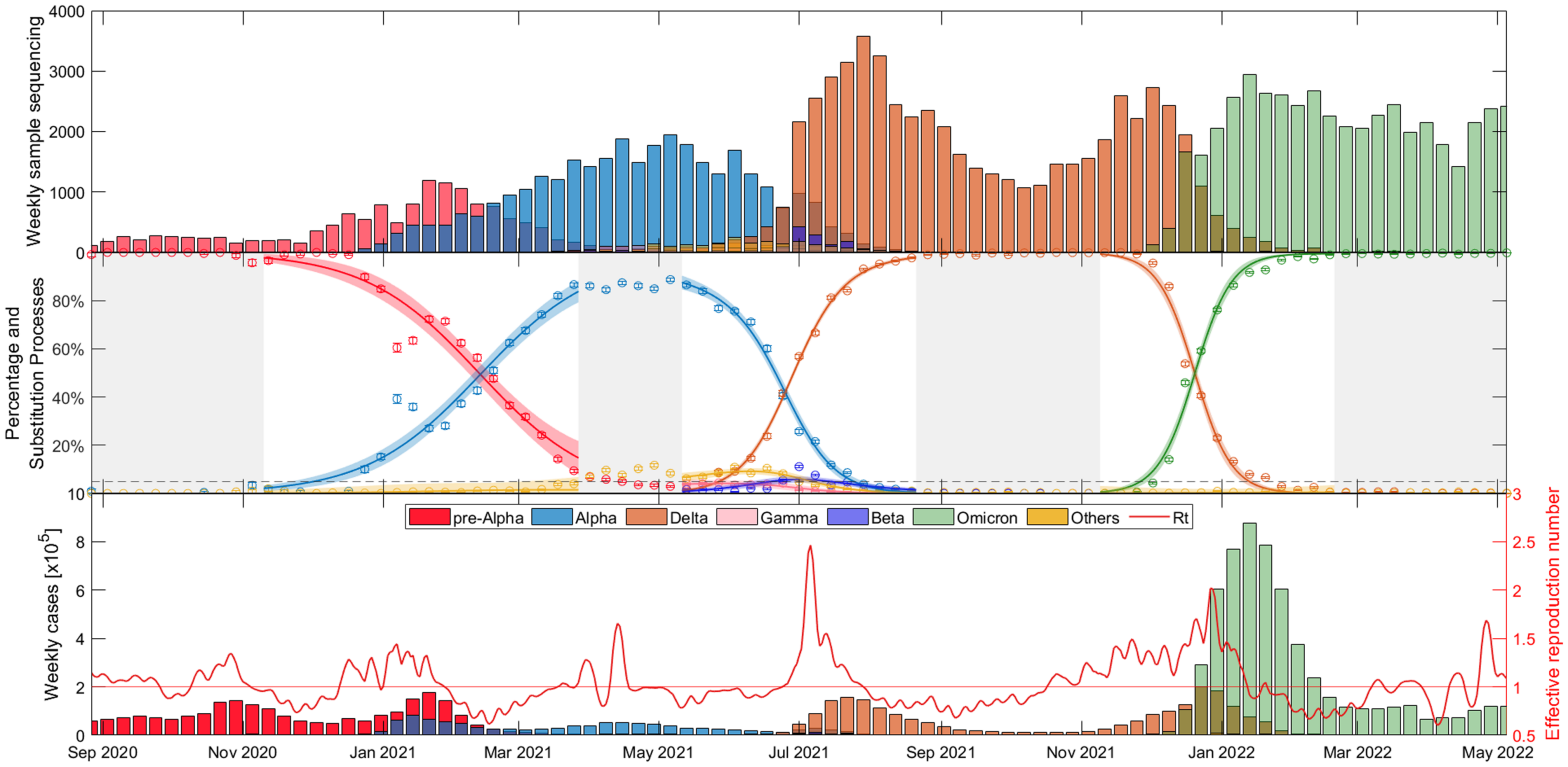
Evolutionary dynamics of SARS-CoV-2 main VOC substitutions (Alpha, Delta, and Omicron) in Spain over time: (top) Weekly sample sequencing, (middle) measured percentage data and mathematical substitution model, and (bottom) estimated weekly COVID-19 cases of each variant and the associated effective reproduction number. The 18 remaining countries and Europe figures are displayed in Suppl. Mat. Text S5.

**Figure 2:**
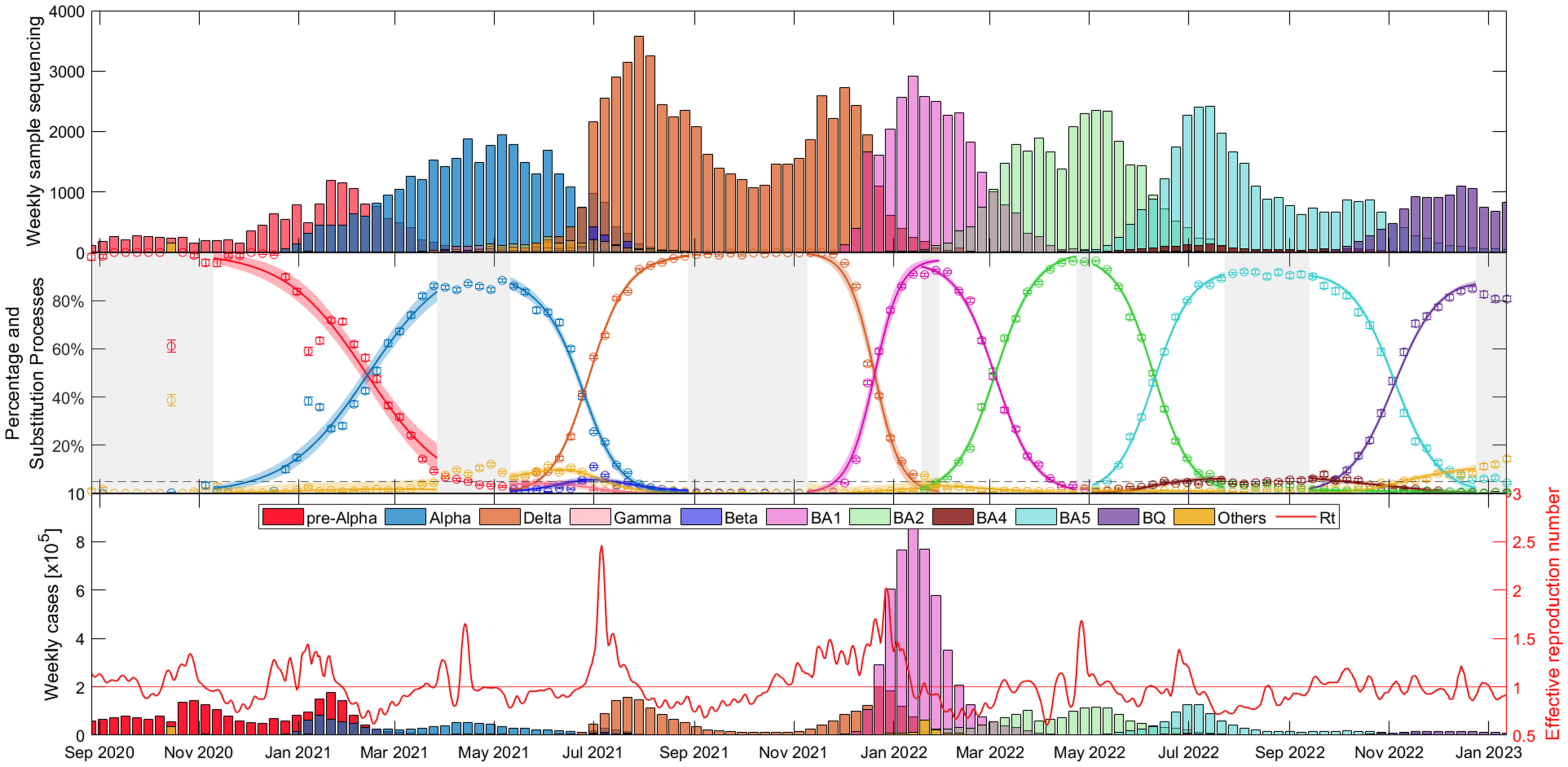
Evolutionary dynamics of Alpha, Delta, and Omicron variants (BA.1, BA.2, BA.5, and, BQ.1 subvariants) substitutions in Spain over time: (top) Weekly sample sequencing, (middle) measured percentage data and mathematical substitution model, and (bottom) estimated weekly COVID-19 cases of each variant and the associated effective reproduction number. The 17 remaining countries and Europe are displayed in Suppl. Mat. Text S6.

The weekly evolution of the samples of each variant, the sampling percentage and the fitted substitution model for each transition period, the estimated number of reported COVID-19 cases corresponding to each variant from our model, and the evolution of the empirical effective reproduction number are shown in Figure 1 (substitution dynamics in Spain from fall 2020 to spring 2022) and Figure 2 (from fall 2020 to early 2023, also including Omicron subvariants). The fitted curves in the middle plot are calculated from Equations 3-5, while Rt (red solid line in the bottom plot) is estimated from Equations 6-8.

As shown in Figure 1, the time window includes the end of the dominance of the *pre-Alpha* variants (red), the slow rise of the Alpha VOC (blue), the increase in dominance of the Delta VOC (orange) along with a notable increase in cases and the $R_{t}$ during the early summer of 2021, and after several months of dominance of the Delta variant, the rapid emergence of Omicron (green) in just over a month, during November–December 2021, along with the highest peak in case numbers.

Figure 2 shows essentially the same as Figure 1, but this time with a wider time window (fall 2020 – early 2023) and separating the Omicron variant into its different major subvariants: BA.1 (pink), BA.2 (light green), BA.4 (brown), BA.5 (light blue), and BQ.1 (purple). It is interesting to note that the duration of dominance varies significantly among SARS-CoV-2 variants and subvariants. While Alpha and Delta dominated for more than 5 and 6 months respectively, the early Omicron subvariants BA.1 and BA.2 showed relatively shorter durations of dominance, lasting only 2.5 and 3.5 months, respectively. BA.5 dominated for almost 5 months and then BQ.1 also displayed a duration of approximately 3.5 months. Additional details on the specific competition between dominant variants in each substitution process can be found in Supplementary material S7, confirming observations similar to those in Figure 2.

Figures 1, 2 provide a comprehensive view of the dynamics and evolution of SARS-CoV-2 variant substitutions in Spain, highlighting the effectiveness of the substitution model in capturing the changes in the dominant variants over time. Each of these substitution processes and figures are described in detail in Supplementary material S8. The results from Spain can be used as a reference for understanding the patterns and trends in the other European countries, presented in Supplementary materials S5, S6.

### 3.2 Statistical analyses of transmissibility and epidemiological indicators

This section focuses on statistical evaluations of variant transmissibility of the Alpha, Delta, and Omicron, and other epidemiological indicators across the 19 European countries studied. Detailed examinations of the subsequent Omicron subvariants and two-variant analyses are relegated to Supplementary materials S10, S12, respectively.

#### 3.2.1 Comparing the transmissibility of the Alpha, Delta, and Omicron variants

We first examine the increase in transmissibility Δβ between the three main VOCs. It is important to note that other variants, although residual, may play a role during the substitution process influencing Δβ. For an exclusive dominant variant result consult Supplementary material S12, but these variants rarely exceed 10% of the national landscape.

**Figure 3:**
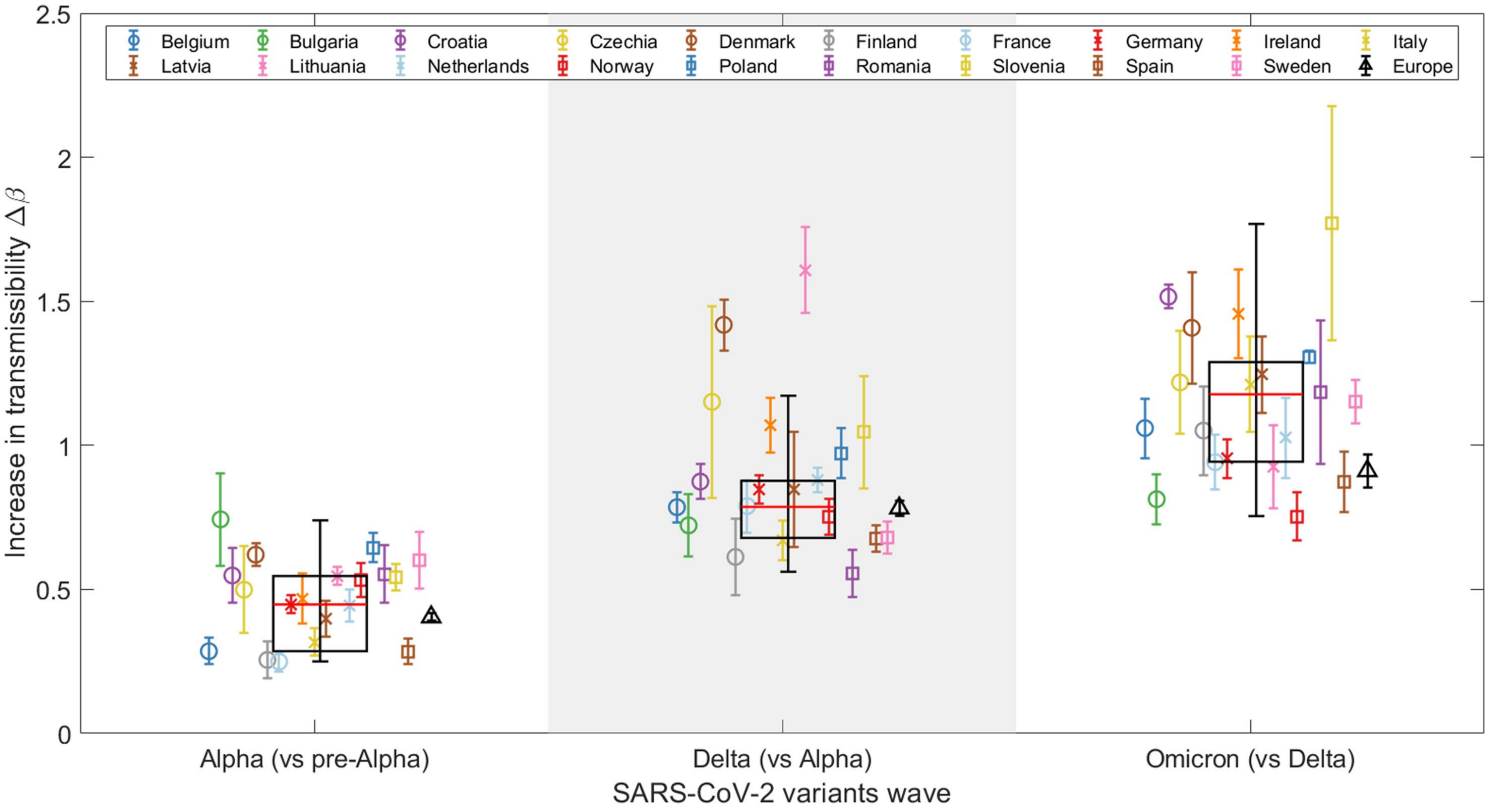
Representation of the increase in transmissibility (Δ*β*) for 19 European countries and Europe (see legend) as a combination of the studied countries, based on the results for the Alpha, Delta, and Omicron VOCs. Weighted boxplots (black boxes) are also included to provide statistical summary. The x-axis distinguishes the three SARS-CoV-2 VOC substitutions, while the y-axis displays the Δ*β* value for each substitution and country.

Figure 3 shows Δβ results for the 19 European countries and Europe as a whole (sum of variants for each week and country), as well as a boxplot of the fitted Δβ for all 19 countries. There is a consistent trend across countries: $\Delta \beta_{Alpha}<\Delta \beta_{Delta}<\Delta \beta_{Omicron}.$ This suggests that the Omicron variant spread more rapidly than both the Alpha and Delta variants. Note that an apparent increase in transmissibility of one variant to the previous one can be the result of intrinsic (virus-related) and/or extrinsic (population-related) factors. Figure 3 also shows weighted boxplots (black boxes) that account for the error associated with the Δβ of our nonlinear regression models. Each Δβ value has an associated error, and we use the inverse of that error as the weight. By assigning higher weights to Δβ parameters with smaller errors, we ensure that the parameters that we estimate with greater precision have a greater impact on our summary statistics. This is especially valuable when the data distribution might be skewed or outliers could disproportionately affect the results. The calculations were done without Europe as a whole, but with the 19 countries used in the analysis. Each box is centered on the median of the data (red line), with the edges of the box indicating the first and third quartiles. The whiskers extend vertically from the box to demonstrate the range of the data, indicating variability outside the upper and lower quartiles, thus providing a sense of the spread and skewness of the data. Points outside the vertical lines, can be considered outliers, thus only Denmark and Lithuania can be considered outliers in the Alpha-Delta substitution. This does not mean that both substitutions are wrong, but that in these two countries the emergence of the Delta variant did not follow the global trend of the others.

Note that in Figure 3, the global increase in transmissibility, when considering Europe as a single region (represented by a black triangle), consistently falls below the median of the boxplot (indicated by the red line) for all substitutions, although the values during the Alpha to Delta substitution are nearly equal, as detailed in Table 1. The disparity observed arises from the calculation method used to determine the global Δβ for Europe, which integrates the sequenced samples from every European country included in this study over the specified periods. This calculation is strongly affected by the asynchrony among substitutions, which we discuss further in Section 3.3. For example, in the last substitution, individual $\Delta \beta_{\text{Omicron}}$ values are high, indicating a fast transition for each country. When we merge all the variants to calculate the substitution for all of Europe, the transition becomes slower. This is because the Omicron substitutions in the 19 countries occur at different times, flattening the slope of the substitution curve and thus lowering the $\Delta \beta_{\text{Europe}}$ (represented by the black triangle). In contrast, the boxplot represents the distribution of individual fittings, which is not affected by the date of substitution onset.

**Table 1:** Results for the increase in transmissibility for Europe as a single region (black triangle in Figure 3) and the median across all countries (red line in Figure 3).

	Alpha	Delta	Omicron
Europe as a single region	0.4041	0.7821	0.9114
Median of European countries	0.4477	0.7863	1.177

#### 3.2.2 Relationship between Δβ and initial day of variant emergence

Variability in Δβ exists both between variants and between countries for the same variant. Factors like variant entry timing, country characteristics, vaccination rates, and current epidemiological conditions can influence these variations.

**Figure 4:**
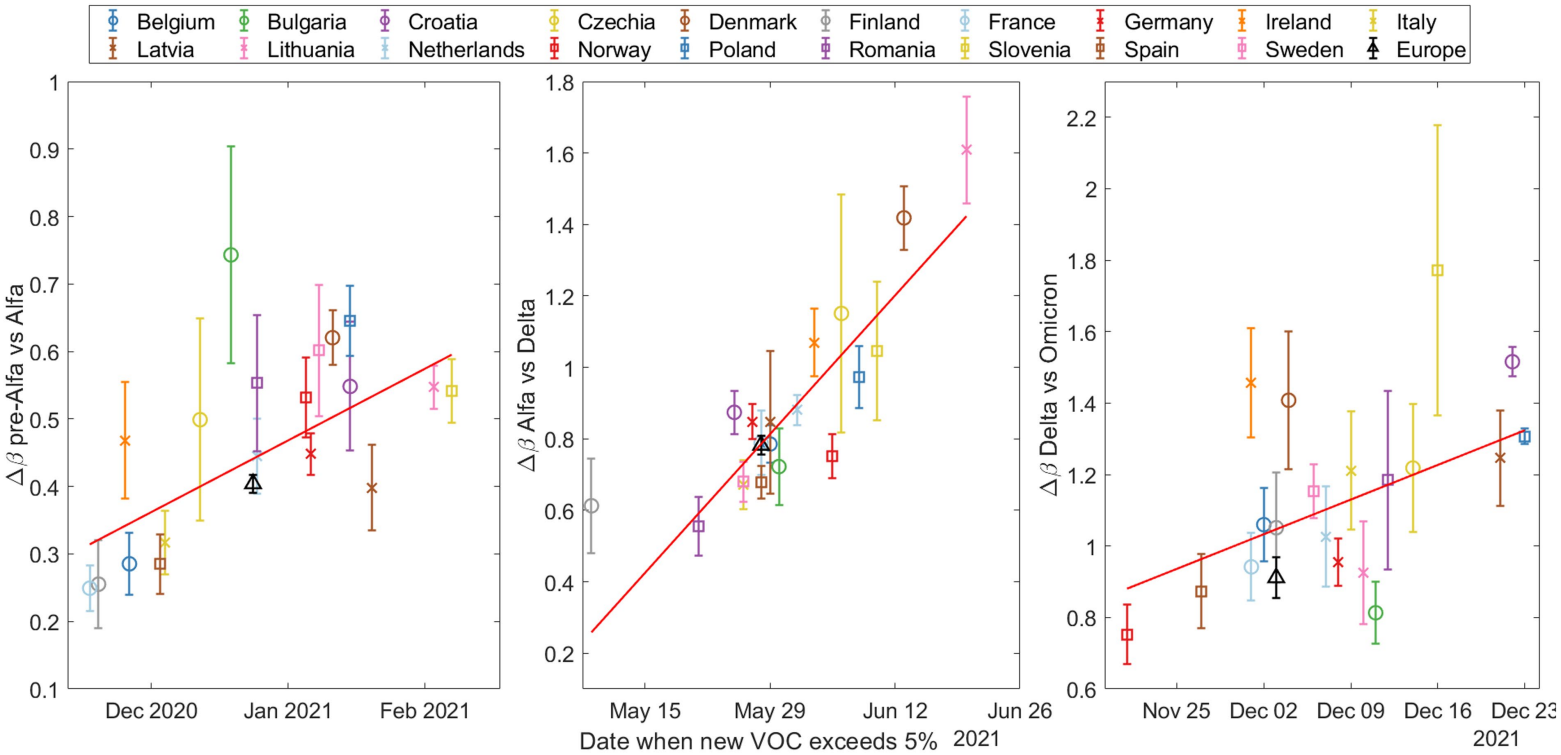
The increase in transmissibility (Δ*β*) based on the date the emerging variant (Alpha on top, Delta in the middle, and Omicron at the bottom) exceeded 5% according to our substitution model. Note that the y-axes are different and then the slopes cannot be compared between plots.

Figure 4 shows the Δβ values relative to the entry date of the dominant variant for each of the three waves of new VOCs. The entry date is defined as the day when the percentage of the new variant exceeded 5%, according to the fitted model. Here, a trend emerges: higher Δβ values are associated with later entry dates for the new variant in a given country. This could be explained by a higher probability of multiple simultaneous entries of the new variant as its prevalence increases in other countries. However, the trend is not perfectly linear, which is to be expected given the varied characteristics of the different countries.

To quantify these trends, we performed Spearman tests, and calculated the coefficient of determination $R^{2}$; see Supplementary material S10 for a more detailed explanation of these statistical tests. The results are shown in Table 2.

**Table 2:** Statistical results for trend analysis across three variants transitions: Alpha, Delta, and Omicron.

	Alpha	Delta	Omicron
$\rho_{5\%}$	0.609	0.804	0.523
*p*-value	0.005	<0.001	0.022
$R^{2}$	0.590	0.670	0.497

As is to be expected, the $R^{2}$ values are not very large, reflecting the complex interplay of real country-specific factors. However, the fact that all three *p*-values are below 0.05 allows us to reject the null hypothesis, thereby providing statistically significant evidence that the variables Δβ and entry date are not independent.

#### 3.2.3 Influence of country surface area on Δβ

We divide the countries of the study into two distinct clusters, in order to better address the role of the surface area: smaller and larger countries. These clusters are categorized based on a geographic area threshold of 200,000 km^2^. This criterion separates larger countries, referred to as cluster 2, including Romania, Italy, Poland, Finland, Germany, Norway, Sweden, Spain, and France (listed in increasing order of size), from the smaller countries (cluster 1) including Bulgaria, Czech Republic, Ireland, Lithuania, Latvia, Croatia, Denmark, Netherlands, Belgium, and Slovenia (listed in decreasing order of size).

**Figure 5:**
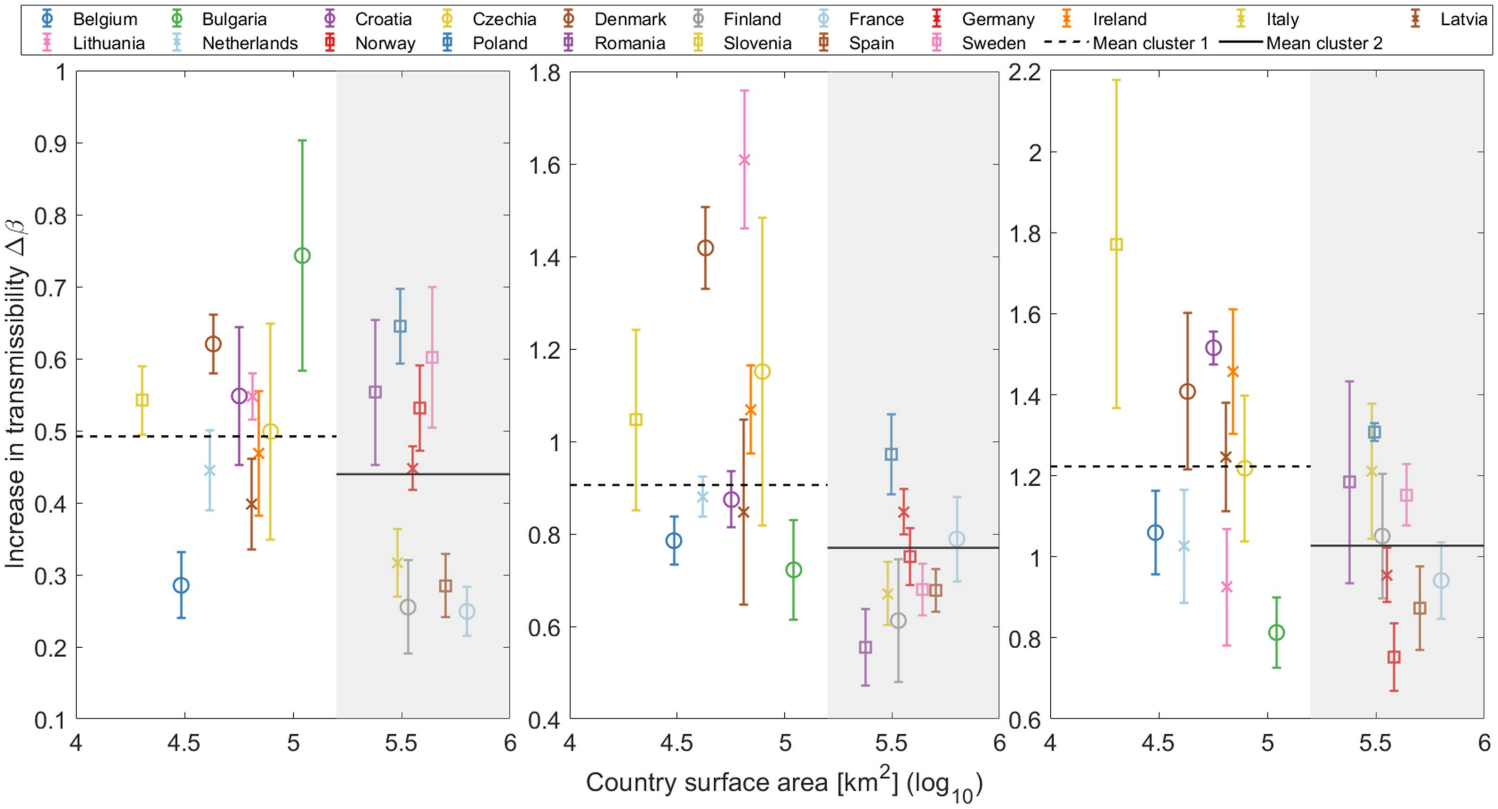
Increase in transmissibility (Δ*β*) plotted against the log of the country’s surface area, distinguishing two primary clusters: smaller countries (cluster 1) on a white background, and larger countries (cluster 2) on a gray background. Mean Δ*β* for both clusters are depicted in each substitution with horizontal lines (cluster 1: Dashed line; cluster 2: Solid line).

This analysis does not provide a clear statistical trend for any substitution or across countries. However, by separating the European countries into two clusters, a discernible pattern emerges as shown in Figure 5. For each VOC transition—Alpha, Delta, and Omicron— the median Δβ parameter is consistently lower for countries with a larger geographic area (cluster 2). This suggests that factors such as population (Supplementary material S9) and geographic area may influence the rate of global variant substitution. Specifically, in larger countries, an asynchronous emergence of the same variant across different regions could potentially affect the substitution process at the national level, leading to an apparent slower increase in transmissibility. This may also explain the consistently lower Δβ value for Europe compared to the median of individual countries, as discussed in Section 3.2.1. Additional insights into the influence of the country surface area on the transmission rate of national variant substitution are explored in Section 3.3.

#### 3.2.4 Δβ in relation to the percentage of fully vaccinated population

Vaccination is a key parameter in understanding the global dynamics of SARS-CoV-2. Vaccines should reduce disease severity, transmission rates, and variant susceptibility. However, the relationship between vaccine coverage and the transmissibility of new variants likely depends on several factors, making the relationship complex.

**Figure 6:**
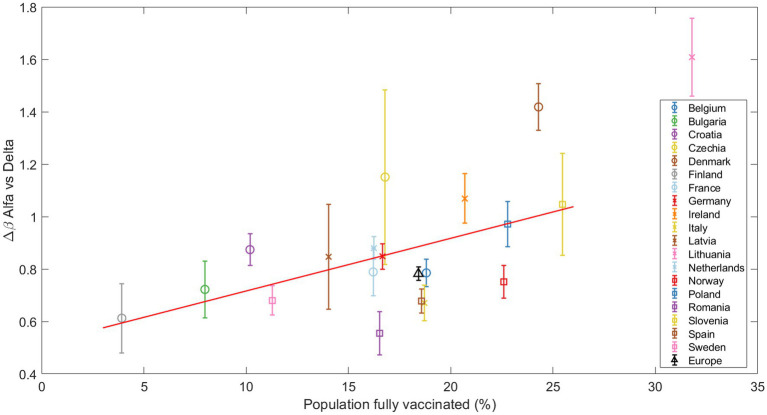
The increase in transmissibility Δ*β* is plotted against the percentage of fully vaccinated individuals at the beginning of the Alpha-Delta substitution (Delta>5%). The red line marks the linear regression, clearly indicating an increasing trend confirmed by the Spearman test. Other substitutions are presented in the Suppl. Mat. Text S9.

At the time of the Alpha variant emergence (December 2020 – January 2021), vaccination programs were not yet widely implemented in Europe. For the Delta variant, our analysis presents an interesting trend. As Figure 6 illustrates, countries with higher vaccination rates showed a greater Δβ of the Delta variant in relation to Alpha. In this analysis, consistent with our previous studies on this substitution, Denmark and Lithuania were excluded due to their outlier status. Statistical testing revealed a Spearman correlation coefficient of 0.5491 with a *p*-value of 0.0164, indicating a moderate positive correlation between the vaccination coverage and the Δβ of the Delta variant.

Finally, for the Omicron subvariants, no clear trend was observed with respect to the vaccination coverage, which is an expected result given the higher immunity level of the population. These results and those for the Alpha substitution can be found in Supplementary materials S9, S10.

#### 3.2.5 Trends in the effective reproduction number during variant substitutions

Finally, a key parameter in understanding the day-to-day progression of a pandemic is the effective reproduction number, or $R_{t}$.

**Figure 7:**
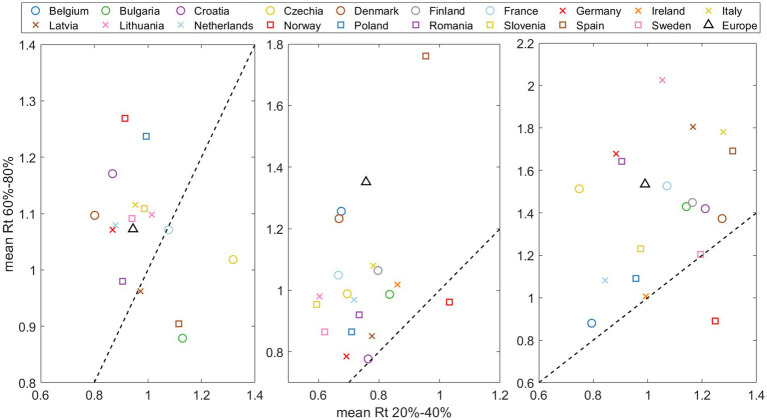
Effective reproduction number mean  $\left(\bar{R_{t}}\right)$  at the end of substitution (accounting for 60–80% of the emerging variant) versus $\bar{R_{t}}$ at the beginning of the substitution (20–40% of total). The dashed line indicates the Rt(after)/ Rt(before)=1/1. threshold, where points above signify an acceleration of the pandemic spreading at the end of substitution.

Figure 7 shows the relationship between the effective reproduction number at the beginning of the substitution (when the old variant dominates, x-axis) and the end (when the new variant dominates, y-axis) for each of the three VOCs substitutions. The data points were calculated by taking the days when the new variant was at a specific percentage (based on our daily substitution model) and calculating the average. These percentages were set at 20–40% for the start and 60–80% for the end of the substitution. We did not choose values lower than 20% or higher than 80% because in most countries, the Alpha variant never exceeded 90% of the total sampling. Thus, the ranges were symmetrical and consistent for each of the three substitutions. Note the left side of Figure 7 (*pre-Alpha* vs. Alpha substitution) is missing some data points because the substitution does not reach 80%. The diagonal dashed line in the figure represents the 1/1 boundary. We observe that most of the points fall above this line, i.e., suggesting that $R_{t}$ generally increases with the entry of a new variant.

### 3.3 The impact of different outbreaks within a single country: a case study of Spain

In the previous section, we observed that larger countries (Figure 5) generally have slower rates of increase in transmissibility Δβ. When we aggregate all sequenced samples and derive results for Europe (Figure 3), these also tend to fall below the median. In this section, we aim to explore how regional outbreaks within a large country, like Spain, can impact its overall viral landscape.

Spain is divided into 17 regions, known as Autonomous Communities (*Comunidades Autónomas* or CCAA in Spanish), and 2 autonomous cities. We used data obtained from the GISAID website, which provides comprehensive information about each sample: day, lineage, location, age… We focused on the Alpha vs. Delta substitution, as there were no major restrictions in the country, the number of weekly samples was high enough to apply the substitution model to individual CCAA, and the substitution was slow enough to observe the expected effect.

**Figure 8:**
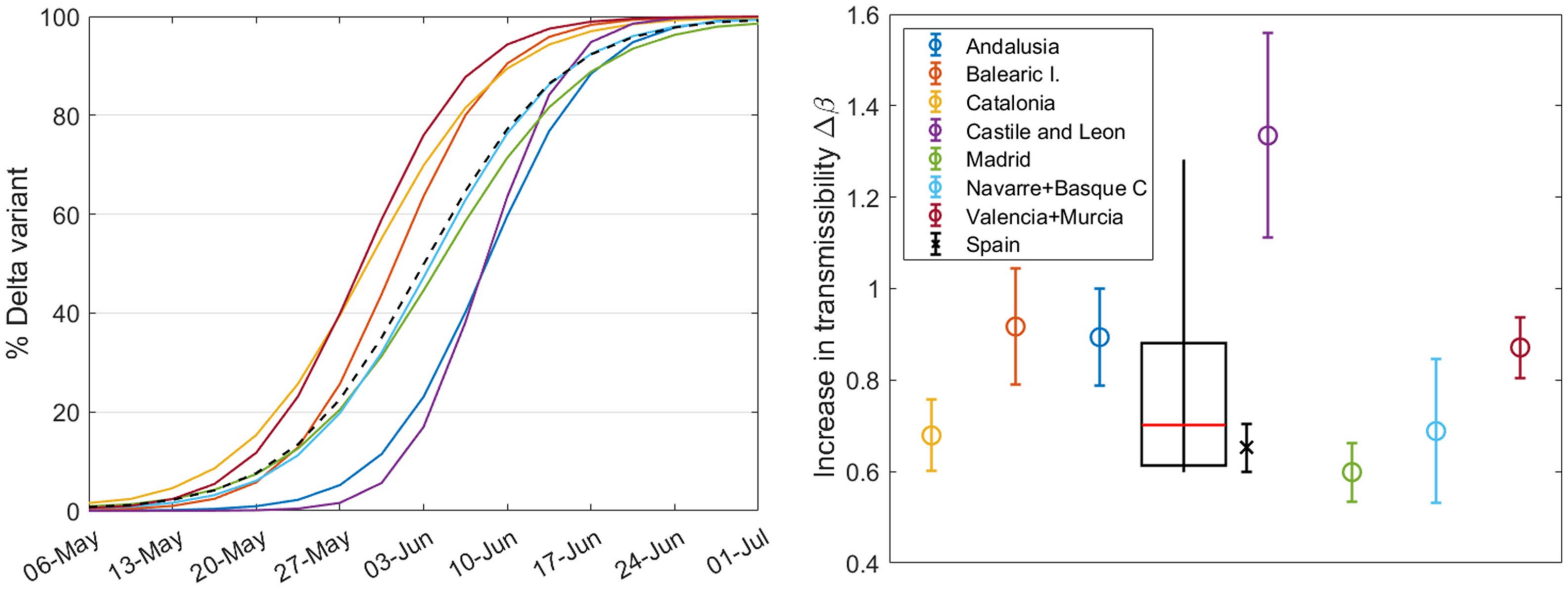
(Left) Daily percentage (without error margins for clarity) of the Delta variant during the substitution process, calculated for different CCAA with n_sample_ ≥1000 during these months. The dashed curve corresponds to the country, calculated solely from the weekly sample sums from the seven regions shown here. Right Increase in transmissibility calculated by the substitution model for each region (circles) and for Spain (cross), with a boxplot showing the median, first and third quartiles, and outliers.

We studied those CCAA that exceeded 1,000 sequences during the months of May and June and merged some medium-sized neighboring CCAA into a single region to reach this threshold. These CCAA are Andalusia (south), Balearic Islands (Mediterranean Sea), Castile and León (north central), Catalonia (northeast), Valencian Community + Murcia (along the Mediterranean coast on the east side), Madrid (center), and Navarre + Basque Country (north of Spain). The daily percentage calculated using the mathematical model from Section 2.3 for these seven groups are shown in different colors in Figure 8 (left). This figure reveals that regions in the northeast and east of Spain were the first to experience an increase in the Delta variant. It gradually spread to other parts of the country, resulting in a time gap of more than 2 weeks between the emergence of the same variant in different CCAA. Looking at the shape of the curve for the entire country, denoted by the dashed black line, we can predict that the calculated Δβ for Spain will be lower than the average for the CCAA. The percentage curve for Spain initially rises with Catalonia, the Valencian Community, and the Balearic Islands, but as the weeks pass, the curve of Spain relaxes and reaches its maximum at the same time as the last CCAA do. In summary, in large countries, the curve slows down from its initial increase driven by the first regions experiencing variant substitution as these regions reach complete substitution while the rest lag behind.

The results of Δβ from our model are shown in Figure 8 (right): the circles stand for CCAA and the cross represents Spain. Note that the result for Castile and León exceeds the interquartile range and will be considered an outlier. The weighted boxplot was calculated in the same way as in Section 3.1. As previously suggested, the result for Spain (black cross), as the sum of the sequenced samples taken weekly in each region, is lower than what we would obtain from the median of the different regions (red line in the boxplot). This supports the previous statements and reinforces the findings of the preceding sections. Note that the increase in transmissibility for Spain, $\Delta \beta_{\text{Spain}}=0.65\pm 0.05,$ does not correspond with that of the previous Section 3.2, $\Delta \beta_{\text{Spain},3.2}=0.68\pm 0.04,$ since we have not taken into account all the regions or CCAA.

### 3.4 Mathematical model with two Omicron subvariants strongly competing and plausible short to medium term scenarios

After the emergence and dominance of Omicron in Europe, the SARS-CoV-2 viral landscape has been characterized by frequent competition, typically involving more than two subvariants. But can we use our substitution model to determine which of the competing variants will ultimately dominate the viral landscape of a country? Our mathematical and computational model returns the parameters Δβ and $\xi_{0}$ based on initial and final conditions set for consistency across countries and substitutions (see Supplementary material S3). For Δβ, which may give us an idea of a short to medium subvariant dominance scenario, the time window is important.

Let us focus only on the last substitution of this study period: during the last quarter of 2022, Europe experienced a shift from the BA.5 variant to BQ.1 and, rapidly, to XBB subvariants (especially XBB.1.5). The Δβ values from our model (Figure 2) showed that BQ.1 and XBB are closely competing for dominance, see Table 3 (left columns). We see no other substitution or country with such closely matched figures (see Supplementary Table S9 to compare Δβ values for all countries and substitutions).

**Table 3:** Parameters for BQ.1 and XBB during substitution, taking into account their individual peak growth periods.

Country	$\Delta \beta \pm \varepsilon_{\Delta \beta }$			
	BA.5 *vs* BQ.1 substitution		BA.5 *vs* XBB substitution (extended weeks)	
	BQ.1	Others (incl. XBB)	BQ.1	XBB
Belgium	0.393 ± 0.023	0.368 ± 0.048	0.352 ± 0.032	0.827 ± 0.046
Croatia	0.257 ± 0.025	0.285 ± 0.038	0.241 ± 0.032	0.790 ± 0.083
Czechia	0.223 ± 0.048	0.318 ± 0.054	0.238 ± 0.048	0.693 ± 0.068
Denmark	0.364 ± 0.027	0.421 ± 0.040	0.281 ± 0.035	0.661 ± 0.044
Finland	0.281 ± 0.026	0.222 ± 0.052	0.231 ± 0.037	0.663 ± 0.052
France	0.348 ± 0.015	0.348 ± 0.050	0.322 ± 0.018	0.807±0.027
Germany	0.223 ± 0.016	0.303 ± 0.037	0.212 ± 0.014	0.594 ± 0.022
Ireland	0.371 ± 0.038	0.413 ± 0.061	0.381 ± 0.044	0.850 ± 0.050
Italy	0.283 ± 0.022	0.288 ± 0.057	0.260 ± 0.023	0.682 ± 0.040
Netherlands	0.312 ± 0.018	0.327 ± 0.030	0.307 ± 0.027	0.700 ± 0.033
Norway	0.311 ± 0.048	0.279 ± 0.077	0.271 ± 0.062	1.020 ± 0.207
Poland	0.400 ± 0.059	0.351 ± 0.076	0.389 ± 0.066	0.778 ± 0.075
Romania	0.418 ± 0.129	0.463 ± 0.158	0.355 ± 0.098	0.725 ± 0.118
Slovenia	0.340 ± 0.035	0.492 ± 0.059	0.328 ± 0.048	0.672 ± 0.059
Spain	0.514 ± 0.026	0.554 ± 0.055	0.484 ± 0.038	0.933 ± 0.044
Sweden	0.352 ± 0.023	0.454 ± 0.045	0.285 ± 0.037	0.662 ± 0.054

We repeated our analysis specifically for this substitution. Here, instead of grouping XBB within the “Others” category as in previous sections, we separated it into an independent group (samples sequenced and classified as both generic XBB and XBB.1.5). We also did the same with the BA.2-like (including BA.2 and BA.2.75) because it was strongly competitive. This new analysis was conducted over a larger time window to observe changes in Δβ: initially, we assumed that the final day of the simulation coincides roughly with the peak of the BQ.1, and now we assume that the final day of the time window corresponds to the peak of XBB. In this analysis, Bulgaria, Latvia, and Lithuania were excluded due to discontinuities in their reporting of sequenced samples. The results are shown in Table 3 (right columns). Here we can see how the parameter $\Delta \beta_{BQ.1}$ remains relatively stable with an average variation between the columns of about 9%. On the other hand, the $\Delta \beta_{XBB}$ parameter varies considerably, always increasing in the last column. The first double column (substitution of BA.5 for BQ.1) is somewhat truncated and does not adequately represent XBB; nevertheless, it shows us that the BQ.1 variant has a clear competitor. By setting the correct time windows, we obtain the real results for all variants in this last complex substitution.

## 4 Discussion and limitations

Our study presents a comprehensive analysis of SARS-CoV-2 variant substitution across 19 countries using publicly available data from the GISAID database. Although the model is limited by the available data, hence focusing on a specific number of countries, its flexibility allows for adaptation to different variant transition scenarios. We selected nonlinear regression for its speed and accuracy.

The results presented in Figures 1, 2 for Spain (and in Supplementary figures S2–S40 for the other European countries) demonstrate the robustness and adaptability of our mathematical approach in capturing the nuanced dynamics of SARS-CoV-2 variant substitutions. The model offers a close match to experimental data even when sequenced samples are limited and multiple variants are competing. Thus, our model can provide an accurate representation of real-world variant dynamics and thus serve as a valuable tool to complement variant sampling in genomic surveillance.

The temporal and spatial evolution of SARS-CoV-2 is evident through our analyses of the increase in transmissibility, Δβ, for the Alpha, Delta, and Omicron variants, which show a consistent trend across European countries, with the Omicron variant exhibiting significantly higher transmissibility (Figure 3) (11, 22). It is noteworthy that the results for the Omicron variant present a higher degree of dispersion compared to those for the Alpha and Delta variants. This may be attributed to the differences in relaxation of containment measures, varying degrees of travel restrictions, and disparities in vaccination coverage across the different countries. Such heterogeneity may have contributed to the observed variability in the Δβ for Omicron variants. Factors like the potential for immune escape and other variables studied here could also play a role. Additionally, the pattern is more heterogeneous across the different Omicron subvariants but always each newly dominant variant has been more transmissible than its predecessor (Supplementary material S10, Supplementary Figure S66).

Generally, our data suggest that later-entrant variants spread more rapidly (Figure 4). We hypothesize that this trend could mainly be attributed to a larger initial number of infections, possibly due to multiple entry points, facilitated by the prolonged circulation of the variant elsewhere. This broader initial presence in later new variant entrances may accelerate transmission within the country. Interestingly, different Omicron subvariants like BA.1 and BA.5 exhibit similar patterns, while BA.2 and BQ.1 seem to be insensitive to this factor (details can be found in Supplementary material S10). Nevertheless, note that the monitoring of the initial substitution dynamics may be strongly affected by the sequencing protocols of each country.

Countries of different sizes showed distinct substitution patterns, with larger countries experiencing systematically slower variant substitution (Figure 5). This could be attributed to multiple epidemiological curves at regional levels separated in time. Smaller countries tend to show faster variant substitution, which might be due to the lack of geographically isolated outbreaks. However, it is crucial to note that this geographical observation does not imply uniform behavior across countries, as other variables discussed in this study, as well as other factors, such as social networks and cultural factors, can also influence transmissibility. To corroborate the purely geographical effect, we studied Spain in detail, which is applicable to other large countries or even Europe as a whole. Section 3.3 underscores the importance of understanding regional differences in the spread of SARS-CoV-2 variants. Low Δβ in larger countries can be attributed to the sequential peaks of outbreaks in different cities or regions. The emergence of new variants in a country begins with initial outbreaks in some areas and ends when the last regions reach their peak, resulting in a smoother and more delayed curve on a country-wide scale, which subsequently leads to lower Δβ values. Furthermore, we demonstrate that the country-wide Δβ value is smaller than the $\Delta \beta_{\text{median}}$ value obtained from individual regions within the same country (Figure 8). The same results are obtained for Europe and for individual countries. Interestingly, among the Omicron subvariants, BA.1 and BA.5 follow the same trend, whereas BA.2 and BQ.1 deviate (see Supplementary material S10).

An important aspect to consider is the interplay between vaccination campaigns and the emergence of new variants (23–25). The introduction of a new variant with some degree of escapement to previous immunity and/or vaccination will encounter a larger population of susceptible individuals, achieving an apparent increase in transmissibility with regards to the previous variant, even if the viral replication rate remains unchanged. This does not mean that the absolute rate of spread of the different variants ($\beta_{i}$) may be lower in a country with a higher percentage of vaccinated population, but rather refers to a higher relative advantage between the two variants, and therefore, to our Δβ. The correlation we observed between vaccination rates and Δβ during the Alpha-Delta substitution (Figure 6) can be explained by this phenomenon (26), although other factors like natural immunity and non-pharmacological interventions could play an important role. This does not imply that $\beta_{\text{Alpha}}$ or $\beta_{\text{Delta}}$ are greater in countries with higher vaccination coverage, since recent vaccination also partially protected against transmission (26, 27), in addition to preventing more severe cases (28), but that the difference between both increased. In fact, some articles emphasize the impact of the Delta variant on unvaccinated individuals (especially younger people, since the vaccination schedules of European countries prioritized older or high-risk individuals first), and this could have contributed to an apparent increase in transmissibility of the new variant (29, 30). This correlation is not observed for any of the Omicron subvariants (Supplementary material S10; Supplementary Figures S80–S83). This could mean that Omicron can spread widely regardless of vaccination rates (31), or that higher levels of vaccination (and pre-existing immunity) mask potential differences in apparent increases in transmissibility. Indeed, current vaccination campaigns focus more on minimizing severe cases than on reducing viral spread.

Interestingly, our study consistently shows a higher effective reproduction number, $R_{t}$, toward the end of a variant substitution for most countries and substitutions (Figure 7). It seems that so far new variants retain the capacity to infect despite preexisting collective immunity. This observation aligns with findings in other papers (19, 32, 33). We have complemented these observations by comparing values at the beginning and end of the substitution process for emerging variants. The same results are observed for the Omicron subvariants (Supplementary material S10; Supplementary Figures S86–S88), suggesting a significant increase in cases accompanying the emergence of a new subvariant.

**Figure 9:**
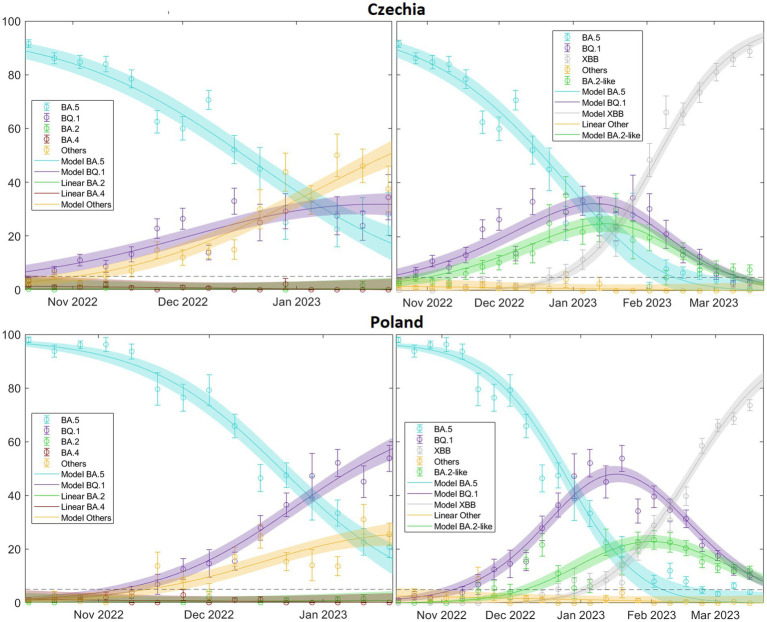
illustrates this for two extreme cases, Czechia, where it is immediately apparent that the “Others” category (including XBB) will quickly dominate  $(\Delta \beta_{BQ.1}=0.223\pm 0.048<\Delta \beta_{\text{Other}}=0.318\pm 0.054),$  and Poland, where the same conclusion may not be initially obvious $(\Delta \beta_{BQ.1}=0.400\pm 0.059>\Delta \beta_{\text{Other}}=0.351\pm 0.076),$ but eventually occurs. This is significant because by comparing the real-time increase in transmissibility, we can understand the current state of the viral landscape of a country. In fact, we could predict the prevalence of currently circulating variants in the short to medium term. For a comprehensive overview of the patterns related to the XBB variant across Europe, refer to (see Supplementary Table S13.

Finally, our model has proven to be reliable in scenarios involving multiple competing variants, as illustrated by our analysis of the BQ.1 and XBB Omicron subvariants, among others (Figure 9). It also underscores the importance of appropriately selecting time windows. In doing so, we can not only illustrate the complex dynamics underlying variant competition but also predict which variant is likely to become dominant, its expected peak time, and its probable prevalence.

This model can be adapted to data from any country or region, as long as the variants data are robust, representative and continuous in time. The reliability of predictions is limited in time and strongly depend on both the quality of data and the velocity of the substitution. The necessary codes to fit the model to data can be found in https://github.com/BIOCOM-SC/cloud-of-codes.

Despite its many significant contributions, this study has some limitations. Our analysis is limited to GISAID data provided by ECDC, which narrows our geographic scope to 19 countries and introduces unknown factors of variability in sequencing and sample collection from different regions and periods due to different health policies in each country. Moreover, the representativeness of variant surveillance may be compromised if the number of samples sequenced is too low. According to the ECDC, the number of samples to be sequenced varies depending on the size of the infected population and the desired level of new variant detection. However, as shown in Supplementary Table S10, except for Alpha substitution, where about half of the countries exceed the threshold number of samples sequenced, most countries have sufficient samples for the Delta and Omicron substitutions to meet the ECDC criteria. While the model has performed well under various scenarios, its performance with data from other sources (including non-European countries) or different databases remains to be evaluated. Additionally, the rapidly evolving nature of the virus requires constant updating; as such, long-term predictions are completely beyond the scope of our mathematical approach. Moreover, the relationship between the transmissibility of different variants and the levels of vaccination within a country is quite complex and strongly affected by other factors like the epidemiological context, as discussed above.

This study not only enriches our understanding of the evolutionary trajectories of SARS-CoV-2 variants but also provides insight into the uniformity with which the pandemic has unfolded across Europe. This contrasts with what other articles targeting a global study suggest (14), offering a unified response strategy for potential new waves of infection.

Future research could focus on incorporating data from a broader range of geographic locations and evaluating the performance of the model using larger, more diverse datasets. In addition, further research into how factors such as population immunity and public health interventions affect variant substitution, as well as exploring the complex interplay between vaccine dynamics and variant transmissibility, could improve our understanding of the evolutionary dynamics of SARS-CoV-2. As our understanding of the virus deepens, continued refinement of our model and others like it will remain critical to our collective efforts to combat the ongoing pandemic.

## 5 Conclusion

Our study offers valuable insights into the pattern of variant substitutions of SARS-CoV-2 across 19 countries. The main findings can be summarized as follows: (i) the continuous evolution of the virus has resulted in an increasing trend in its transmissibility from the Alpha variant to Delta and subsequently to the various Omicron variants; (ii) later-emerging variants tend to spread more rapidly upon their entry into a population, which can be explained by a higher chance of multiple entries; (iii) the spread pattern of COVID-19 variants displays significant variations across countries of differing sizes, with a slower substitution pattern in larger countries; (iv) a significant correlation has been identified between vaccination rates and the relative advantage of Delta with respect to Alpha, independently of their absolute propagation rate, but no relation has been found with the Omicron subvariants; (v) $R_{t}$ values tend to increase with a new variant substitution process, emphasizing the spread potential of new variants; (vi) the model shows robustness even in scenarios of multiple competing variants, accurately representing the underlying dynamics of variant competition.

All these results confirm the usefulness of this mathematical framework, which allows for the accurate and reliable estimation of daily case numbers, providing a valuable complement to variant sampling in SARS-CoV-2 genomic surveillance.

These findings have several implications for public health policy and practice. First, the continuous evolution of the virus underscores the need for ongoing genomic surveillance to identify new variants of concern, as the entry of new variants may alter the epidemic situation. Second, the ability of the model to predict the spread of new variants based on their initial entry may aid in proactive planning and response. Third, our findings on variant spread in countries of different sizes and entry timing can inform more nuanced, context-specific public health interventions.

## Data availability statement

The datasets presented in this study can be found in online repositories. The names of the repositories and accession number(s) can be found in the Supplementary material.

## Author contributions

VLdR: Conceptualization, Data curation, Formal analysis, Investigation, Methodology, Validation, Visualization, Writing – original draft, Writing – review & editing. AP-M: Data curation, Formal analysis, Investigation, Methodology, Validation, Writing – review & editing. SA: Investigation, Methodology, Validation, Writing – review & editing. CA: Investigation, Validation, Writing – review & editing. AA: Investigation, Validation, Writing – review & editing. AB: Investigation, Validation, Writing – review & editing. JC: Investigation, Validation, Writing – review & editing. P-JC: Investigation, Validation, Writing – review & editing. MC: Investigation, Methodology, Validation, Writing – review & editing. DL: Investigation, Methodology, Validation, Writing – review & editing. SM: Investigation, Validation, Writing – review & editing. EM: Investigation, Validation, Writing – review & editing. VS: Investigation, Validation, Writing – review & editing. CP: Funding acquisition, Conceptualization, Investigation, Methodology, Project administration, Supervision, Validation, Writing – review & editing. EA-L: Conceptualization, Funding acquisition, Investigation, Methodology, Project administration, Supervision, Validation, Writing – review & editing.

## Footnotes

^1^ https://www.who.int/activities/tracking-SARS-CoV-2-variants

^2^Lithuania has not reported any sequencing data from early May 2022 to late October 2022, thus it has been omitted from the Omicron variant analysis.

## Supplementary material

SUPPLEMENTARY MATERIAL TABLE 1a Suppl_Mat_Table_1a_GISAID_data.xlsx.

SUPPLEMENTARY MATERIAL TABLE 1b Suppl_Mat_Table_1b_TESSy_data.xlsx.

SUPPLEMENTARY MATERIAL TABLES 2 GISAID vs TESSy (ECDC).

SUPPLEMENTARY MATERIAL TABLE 3 Percentage GISAID per Country.

SUPPLEMENTARY MATERIAL TABLE 4 New cases & deaths.

SUPPLEMENTARY MATERIAL TABLE 5 Δβ Figure 1.

SUPPLEMENTARY MATERIAL TABLE 6 Δβ Figure 2.

SUPPLEMENTARY MATERIAL TABLE 7 Δβ Figure 3.

SUPPLEMENTARY MATERIAL TABLE 8 Different approaches.

SUPPLEMENTARY MATERIAL TABLE 9 All Δβ Figure 1.
