# Supplementary material for: Mathematical modeling of SARS-CoV-2 variant substitutions in European countries: transmission dynamics and epidemiological insights
Source: Front Public Health. 2024 May 15;12:1339267. doi: 10.3389/fpubh.2024.1339267 (PMC11160439; doi:10.3389/fpubh.2024.1339267)
Supplement: Supplementary file 4 [file Data_Sheet_1.PDF]

Supplementary Material

to de Rioja *et al.*, *Mathematical modeling of SARS-CoV-2 variant substitutions in European countries: Transmission dynamics and epidemiological insights*

Suppl. Material Text S1 – Data: GISAID, TESSy and WHO **2**

Suppl. Material Text S2 – Challenges encountered in data sets **4**

Suppl. Material Text S3 – Detailed procedure of the automated substitution model approach **7**

Suppl. Material Text S4 – Detailed of different fitting procedures in the substitution model **10**

Suppl. Material Text S5 – Main substitutions Alpha, Delta, and Omicron dynamics for each country with GISAID data source **11**

Suppl. Material Text S6 – Substitutions dynamics with Omicron lineages for each country with GISAID data source **22**

Suppl. Material Text S7 – Two-variant substitutions dynamics with Omicron lineages for each country with GISAID data source **32**

Suppl. Material Text S8 – Detail of each substitution process for Spain **43**

Suppl. Material Text S9 – Additional analyses of transmissibility in relation to population size, density, and vaccination status **45**

Suppl. Material Text S10 – Transmissibility differences among Omicron lineages using GISAID data source **48**

Suppl. Material Text S11 – Assessing independence of GISAID and TESSy through transmissibility differences **69**

Suppl. Material Text S12 – Transmissibility analysis in the context of two-variant problem **80**

Suppl. Material Text S13 – Extensive analysis of variant competitiveness (BQ.1 and XBB) across European countries **86**

Supplementary Tables **92**

Suppl. Material Text S1 – Data: GISAID, TESSy and WHO

In this Supplementary Material Text, we delve deeper into the data sources used in our study, namely the GISAID EpiCoV database and ECDC's TESSy database, as briefly mentioned in **Section 2.1 SARS-CoV-2 variants databases** of the main manuscript. Substantial variations exist in the quantity of samples, depending on the country, data source, and specific time frames. Some countries appear to exclusively report their sequenced samples to a single source, while others seem to submit identical data to both databases.

Given these issues, our main manuscript uses only the GISAID data due to its wide recognition in the literature and its provision of data that allows analysis of the first significant substitution (pre-Alpha vs Alpha).

To provide a better understanding, we have included the data for all weeks, all countries, and all variants in Supplementary Tables 1a and 1b. Additionally, Supplementary Table 2 contains the weekly recounting of the sequencing for each country (without considering the type of variant) for each data source. From these numbers, Supplementary Table 3 calculates the percentage of GISAID weekly reported samples versus the total number of reported sequences from the two sources, GISAID and TESSy.

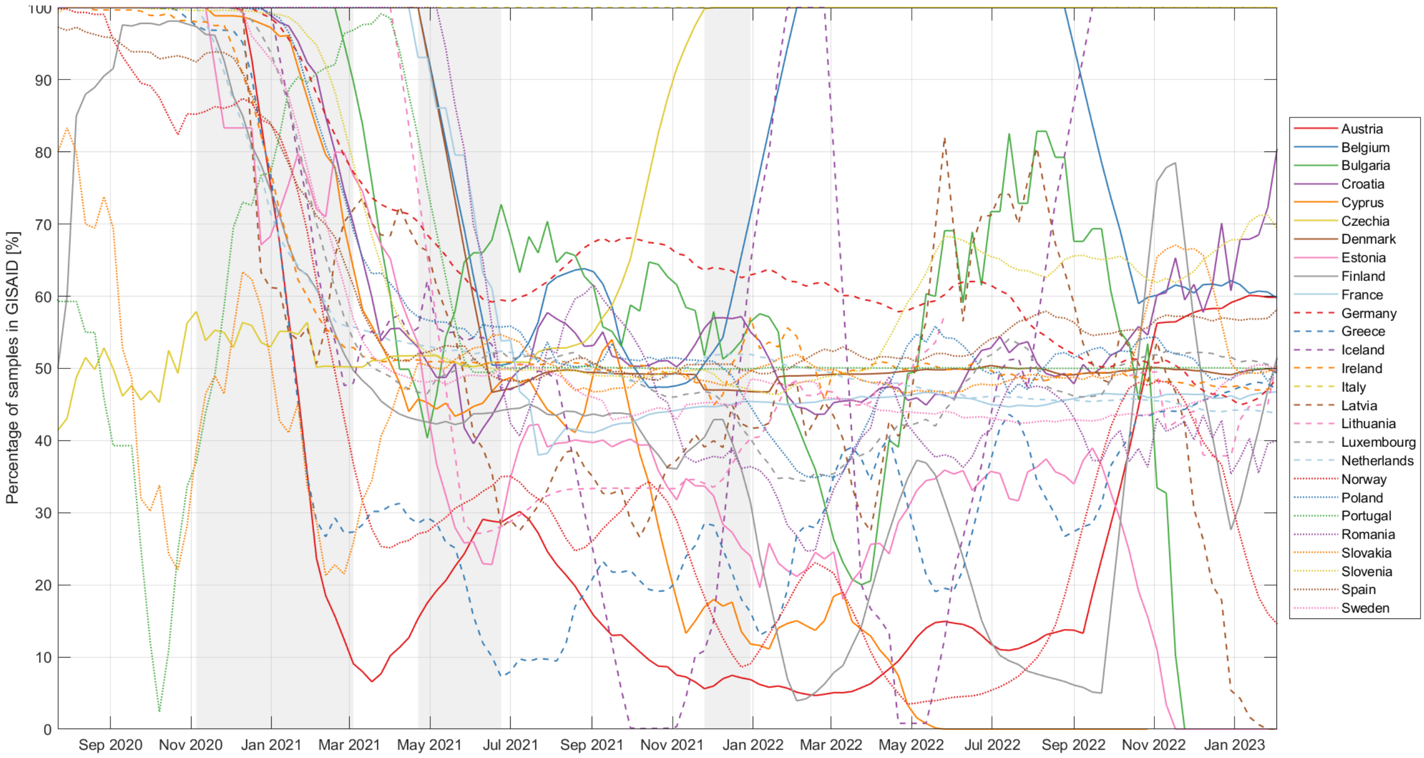

Figure S1 Percentage of total sequenced variants per week in GISAID in comparison to the total from both GISAID and TESSy.

Figure S1 represents the smooth weekly percentage of each country's samples reported to GISAID in relation to the total number of samples (GISAID+TESSy), facilitating a comparison between the two databases. This figure is a direct plot of the data found in Suppl. Table 3.

Further exploration of a combination of the two sources can be found in Suppl. Material Text S10

In addition to the GISAID and TESSy data, we have also utilized data on cases and deaths from the World Health Organization's database. This database is particularly useful because it provides data on a daily basis, whereas the former two sources provide data on a weekly basis.

The daily data on confirmed COVID-19 cases are fundamental in our estimation of the effective reproduction number, as detailed in **Section 2.4** of the main text.

Supplementary Table 4 contains the numbers extracted directly from the WHO's dedicated COVID-19 pandemic database, which can be accessed at https://covid19.who.int/data.

Suppl. Material Text S2 – Challenges encountered in data sets

As highlighted in **Section 2.1**, our study relies on two data sources, GISAID and TESSy, which offer variant sequencing information by country and week. However, these sources present variations in the quantity of data points across different countries and time frames. This suggests that at certain times, data may originate from independent sources, while at other times, the same data might be duplicated, leading to overlapping sources. We selected GISAID as our primary variant sequencing data source for the primary calculations and analysis in this article, mainly due to its extensive date range (early 2020 – present). GISAID's extensive database provides an exceptional resource for tracking the evolution and spread of SARS-CoV-2 variants worldwide. However, it is important to recognize that GISAID, like any large data repository, is not without its limitations and challenges. A major limitation is the absence of detailed reasoning for sequencing decisions and the lack of clinical characteristics of the patients associated with the sequences. In addition, the variability of sampling strategies across different countries and time periods introduces additional complexity. Also, sequencing the same number of samples in contexts where the total number of cases vastly differs (e.g., sequencing 100 samples when there are a thousand cases versus when there are ten thousand cases) can yield dramatically different insights into the prevalence and spread of the virus. A first insight into this problem is shown in Figure S2, which shows the different sampling sequence policies across countries.

To address these nuances and ensure a robust analysis, we examined the European Centre for Disease Prevention and Control (ECDC) methodology, which provides specific thresholds for the minimum number of samples that should be sequenced to detect variants of SARS-CoV-2 at a 95% confidence level in a given population size of confirmed COVID-19 cases. By examining the percentage of samples sequenced relative to the total number of cases reported, we have compiled a table to assess compliance with these guidelines in the emergence of the three main SARS-CoV-2 variants of the manuscript (Alpha, Delta, and Omicron), see **Supplementary Material Table S10**. The numbers show a heterogeneous compliance across countries: during the entry phase of the Alpha variant, where most countries did not meet the ECDC sequencing thresholds, with notable exceptions like Denmark and the Netherlands, and during the emergence of the Delta and Omicron variants, where almost all countries met the criteria.

GISAID provides data for 30 European countries: Austria, Belgium, Bulgaria, Croatia, Cyprus, Czechia, Denmark, Estonia, Finland, France, Germany, Greece, Hungary, Iceland, Ireland, Italy, Latvia, Liechtenstein, Lithuania, Luxembourg, Malta, Netherlands, Norway, Poland, Portugal, Romania, Slovakia, Slovenia, Spain, and Sweden.

Due to low sampling rates (with several weeks reporting zero samples) and significant variability in the data, three countries have been firstly excluded from our analysis: **Hungary**, **Liechtenstein**, and **Malta**.

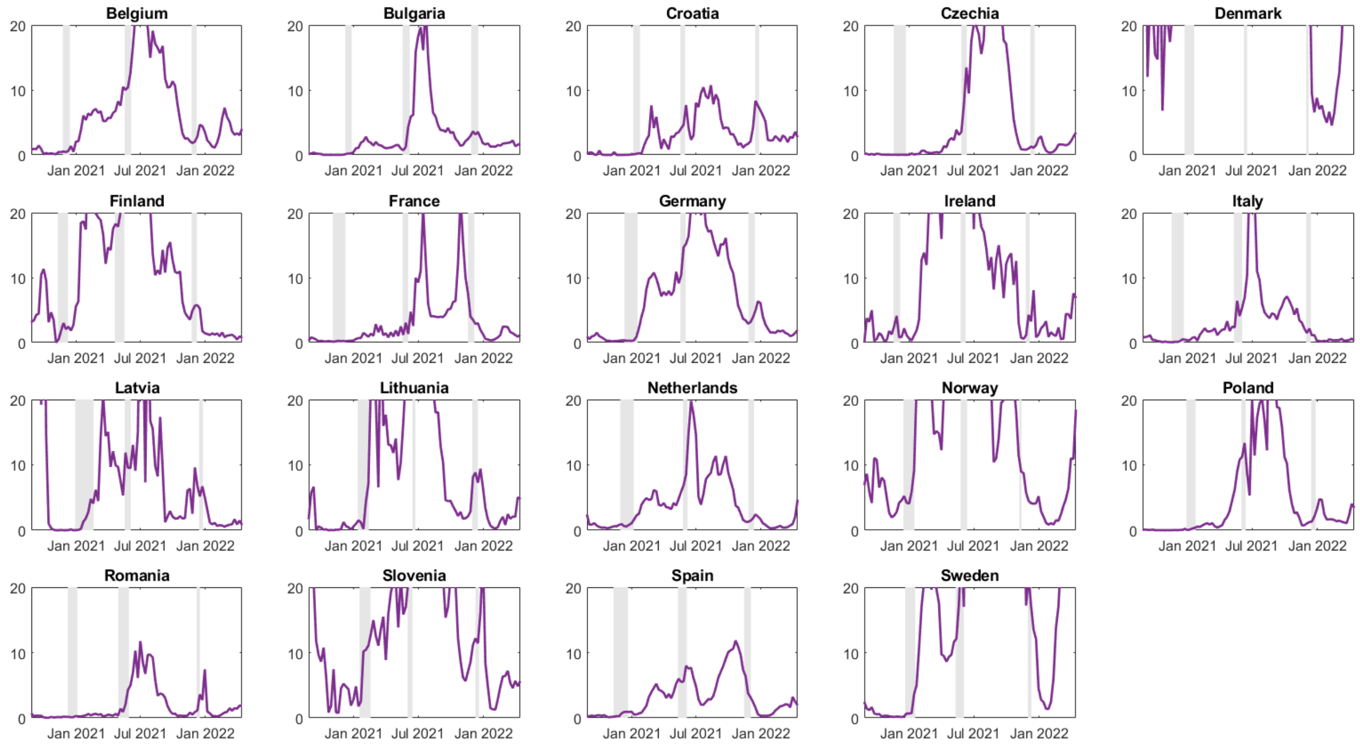

Figure S2 Nineteen images showing the percentage of sequenced samples relative to the total cases from the 19 countries studied in the main manuscript. Percentages are highlighted in purple, and grey boxes mark the dates at which new variants (in chronological order: Alpha, Delta, and Omicron) increase their presence in the country's viral landscape from 1% to 10%, as calculated from our mathematical model.

Out of the remaining 27 countries, we decided not to conduct the analysis with GISAID data for eight countries for various reasons:

The data set for **Austria** is markedly distinct from those of all other countries under consideration, with a considerable volume of variants categorized as “Unknown”. During the periods of dominance by the Alpha or Omicron lineages, these two VOCs are significantly eclipsed by the huge number of unidentified variants. Uniquely, when examining Austria's complete data set spanning over three years, there are 150,820 “Unknown” samples versus 250,448 total sequenced samples, amounting to over 60% of the total. The countries closest to this trend are France and Italy, but they only exhibit this issue to a much lesser extent, with a proportion of unidentified variants amounting to just 1.5% and 1.9% respectively. Due to this considerable discrepancy, we have opted to exclude Austria from the primary results in our study for the sake of clarity. For the remaining countries, the degree of error introduced by including the “Unknown” variants is relatively negligible, thus we have retained these within the “Others” category.

In **Cyprus**, aside from the low number of sequences (barely surpassing a hundred samples a couple of weeks), the switch from Delta to Omicron occurs from one week to another, jumping suddenly from 0% to 100%. Additionally, Cyprus ceased to take samples from April 2022.

Estonia presents a relatively regular database from the time of the Alpha variant's replacement. It has only a few weeks with very few samples, but this coincides with the time of the Alpha to Delta lineage switch (June 2021), and again, we see a jump from 0

to 100% in just one week. This, far removed from reality and purely due to the low number of sequences in these key weeks, means we must disregard it.

The data in **Greece** exhibits high volatility: the numbers can fluctuate from a few samples one week to over 200, then drop to a very low number within a month. This results in high percentages of variants and lineages classified as “Others” at times when we understand they should not be so high.

Iceland generally has a very low number of weekly samples (sometimes none) except for several consecutive weeks when over 500 sequences can be taken. These weeks with a higher sample number coincide with periods of variant dominance, making the substitutions unclear and less rigorous.

In **Luxembourg**, significant sequencing drops precisely at the time of variant substitutions, as in the rest of these cases. Furthermore, it appears to exhibit a stepped growth during the rise of Alpha and notable variability in the substitution of Alpha by Delta.

Portugal presents a large amount of data accumulated in notable weeks, and the rest of the weeks with a very limited number, up to June 2021. From this date, right at the Alpha to Delta substitution, its sample number is constant and very good, always around 500 and 600; but we had to discard it due to the initial data.

The first major substitution in **Slovakia** is very chaotic, continually presenting, for more than 2 months, jumps between the variants classified as “Other”, the B.1.1.7 and all the rest (grouped in the same pack).

In summary, the countries included in our study with data from ECDC corresponding to the GISAID source are Belgium, Bulgaria, Croatia, Czechia, Denmark, Finland, France, Germany, Ireland, Italy, Latvia, Lithuania, Netherlands, Norway, Poland, Romania, Slovenia, Spain, and Sweden.

Suppl. Material Text S3 – Detailed procedure of the automated substitution model approach

Mathematical substitution model

The simplest scenario for a substitution model involves only two variants: the initially dominant one (1) and the one that will ultimately prevail (2). Both variants evolve following an exponential dynamic equation:

$$N_i = N_{i,0}\, e^{\beta_i t}, \quad \text{Eq. 1}$$

where N_i are the number of cases and β_i are the exponents related with the transmissibility (therefore, to the effective reproduction number) of each variant. The effective reproduction number, R, of each variant was assessed from Eq. 1, assuming a fixed mean period τ between infection and maximum infectivity of individuals:

$$R(t) = \frac{N_i(t+\tau)}{N_i(t)} = e^{\beta_i \tau} \quad \text{Eq. 2}$$

Therefore, we could determine the values of the exponents from the effective reproduction numbers as:

$$\beta_i = \frac{\ln R_i}{\tau} \quad \text{Eq. 3}$$

If variant 2 presents a population-level increase in transmissibility of η with respect to variant 1, that is, $R_2 = \eta R_1$, the exponents can be related as $\beta_2 = \frac{\ln R_2}{\tau} = \Delta\beta + \beta_1$, where $\Delta\beta = \frac{\ln \eta}{\tau}$ is directly related to the increase in transmissibility of the emerging variant with regards to the previously dominant one. Although β_1 and β_2, can change in time there is a relation between them which remains constant $\Delta\beta = \beta_2 - \beta_1$, because τ is fixed.

In this case, we derive the following equation for the fraction of the emerging variant during the transition period:

$$p_2(t) = \frac{\xi_0 e^{\Delta\beta\, t}}{1 + \xi_0 e^{\Delta\beta\, t}}, \quad \text{Eq. 4}$$

where ξ_0 is the initial ratio between variant 2 (future dominant lineage) and variant 1 (previous dominant lineage). Therefore, as for the latter, which is on the decline, we find $p_1(t) = 1 - p_2(t)$.

These equations are useful when we aim to compare the increase in transmissibility between only two variants. However, they generally may not adequately capture the virus dynamics within a country, considering the presence of multiple circulating lineages concurrently. Results from this simplified approach are illustrated in **Suppl. Mat. Text S7**, while a comprehensive analysis of all $\Delta\beta$ values can be found in the **Suppl. Mat. Text S12**.

When three or more variants are involved, additional equations are incorporated to account for the increased complexity. Assume that N variants are competing to dominate the national viral landscape. For each of these aspiring variants, one can write the fraction of cases of a specific variant, i, among the total as:

$$p_i(t) = \frac{\xi_{0,i} e^{\Delta\beta_i t}}{1 + \sum_{j=1\ldots i\ldots N} \xi_{0,j} e^{\Delta\beta_j t}}, \qquad \text{Eq. 5}$$

where $\xi_{0,i}$ represents the initial ratio between variant i and all others, and $\Delta\beta_i$ is the parameter associated with the increase in transmissibility between the variant i and all the other lineages in the national viral scenario at that time. We can calculate the fraction corresponding to the descending variant as:

$$p_{desc}(t) = 1 - \sum_{j=1\ldots i\ldots N} p_j(t). \qquad \text{Eq. 6}$$

As previously explained, not all variants exhibit a peak behavior. Some variants present different trends: remain constant, increase linearly, decrease… In such cases, we can simulate each shape of the various lineages competing to dominate, as before, but excluding the one considered as residual. Consider N' variants behave as a peak or wave (those competing to dominate the national viral landscape) and M' lineages that can be considered as a residual (with low impact in the general landscape). The total variants at that point are $N' + M' + 1$ (to consider the one that would decline). Then, if variant k is one of least importance in the substitution, i.e., $k = 1, \ldots, M'$, the previous equations can be rewritten as:

$$\begin{aligned} p_i(t) &= \frac{\xi_{0,i} e^{\Delta\beta_i t}}{1 + \sum_{\substack{j=1\ldots i\ldots N \\ k\neq j}} \xi_{0,j} e^{\Delta\beta_j t}}, \\ p_k(t) &= \xi_{0,k} + \Delta\beta_k t, \\ p_{desc}(t) &= 1 - \sum_{\substack{j=1\ldots i\ldots N \\ k\neq j}} p_j(t). \end{aligned} \qquad \text{Eq. 7}$$

The model has been developed to emphasize the wave-substitution shape; hence the dropping lineage is constructed solely among these. So, in this case, normalization will be necessary to accomplish that:

$$p_{desc}(t) + \sum_{i=1}^{N'} p_i(t) + \sum_{k=1}^{M'} p_k(t) = 1, \qquad \text{Eq. 8}$$

for each instant (in our case, for each day).

Computational substitution model

Our computational approach involves a series of systematic steps, allowing us to understand and map the variant substitution for each country, considering their unique context. The methodology proceeds as follows:

1. Weekly samples of each variant are collected and classified into distinct groups, with VOCs considered individually and the rest combined into an "Others"

category. This step enables us to calculate the weekly percentages and the associated binomial variable errors.

2. We define the time window for each variant substitution. This interval begins when the primary ascending variant surpasses 1% of the total percentage of variants and rises above 3% in the subsequent week. The end is marked when this variant exceeds a specific threshold, which can range between 70% and 95%, depending on the variant substitution process, and when the variant's percentage starts to decline in the following week. Special considerations are factored in to prevent false starts due to the volatile nature of data in certain countries.

3. Our mathematical model is applied to discern if the substitution process involves two or more variants. Those lineages that do not meet a specific threshold or show a consistent linear rise or fall are categorized as "residual" variants and treated with linear models. This approach improves the fit for variables that display a substitution behavior.

4. We start by fitting the parameters in an ideal scenario, where all variants can be included as substitution models Eq. 5 and Eq. 6 in the main text. If this does not yield a satisfactory result for all variables, we proceed to less restrictive models, iteratively considering various lineages as linear or not until we find the best fit, Eq. 7.

5. This iterative process continues until the best model is found, which meets our established criteria. The code then automatically computes key parameters (ξ_0 and $\Delta\beta$) and estimates the daily percentages of each variant, the 95% confidence intervals (CIs), and the mean squared error. Additionally, we provide an array of significant metrics related to the substitution process, such as the timeline of a variant's rise and the total number of daily cases, helping to build a comprehensive understanding of the pandemic's progression across different countries.

This innovative approach provides a nuanced and dynamic understanding of the evolution and substitution of COVID-19 variants, delivering a robust framework for further research and future pandemic preparedness.

Suppl. Material Text S4 – Detailed of different fitting procedures in the substitution model

To address the challenges posed in obtaining the best parameters of the fit in the substitution model, we employ a variety of mathematical approaches, including non-linear fitting, weighted regressions, and Monte Carlo methods. More specifically, we have employed three different methods to estimate the parameters of the mathematical model and their corresponding uncertainties. Here, we present an overview of the three approaches, highlighting their key mathematical aspects. The first method is our primary approach; it is direct and computationally efficient. The other two methods parallel the primary one but require extensive simulations or additional weighting schemes.

Nonlinear regression: This method involves curve fitting to obtain initial parameters, followed by nonlinear regression to refine the estimates. The algorithm refines the initial parameter estimates by minimizing the sum of squared residuals (the differences between the observed data and the model predictions). Parameter uncertainties are determined using the covariance matrix (the standard errors of the parameter estimates can be ascertained by taking the square root of the matrix's diagonal elements), and 95% confidence intervals for the model's predictions are constructed, considering both the parameter estimates' uncertainty and the observed data's random variation.

Monte Carlo simulation: In this approach, we generate 1000 experiments or 1000 new sets of data that follow a binomial probability distribution based on the percentages of each variant for each substitution and country. For each "experiment," we calculate the parameters and the result using the same algorithm as in the first method. Then, we compute the average and the associated error for these 1000 results. This approach allows for a more robust estimation of parameter uncertainties by accounting for the observed data's random variations.

Weighted regression: Lastly, we repeat the process of the first method, this time assigning specific weight to each data point. The weight of each point is calculated as the inverse of the binomial error. With these weights, the nonlinear regression process is conducted, affording more importance to the data points with lower uncertainties.

These three methods yield comparable parameter estimates for the mathematical model, although the confidence intervals differ. This ensures that our results are robust and reliable, providing a solid basis for analyzing COVID-19 variant substitution. Results can be found in Suppl. Mat. Table 8.

Suppl. Material Text S5 – Main substitutions Alpha, Delta, and Omicron dynamics for each country with GISAID data source

Here we present an expansion of Figure 1 from the main manuscript, extending the analysis to the 19 countries and Europe, and covering the main lineage substitutions - Alpha, Delta, Omicron.

Numerical results can be found in Supplementary Material Table 5.

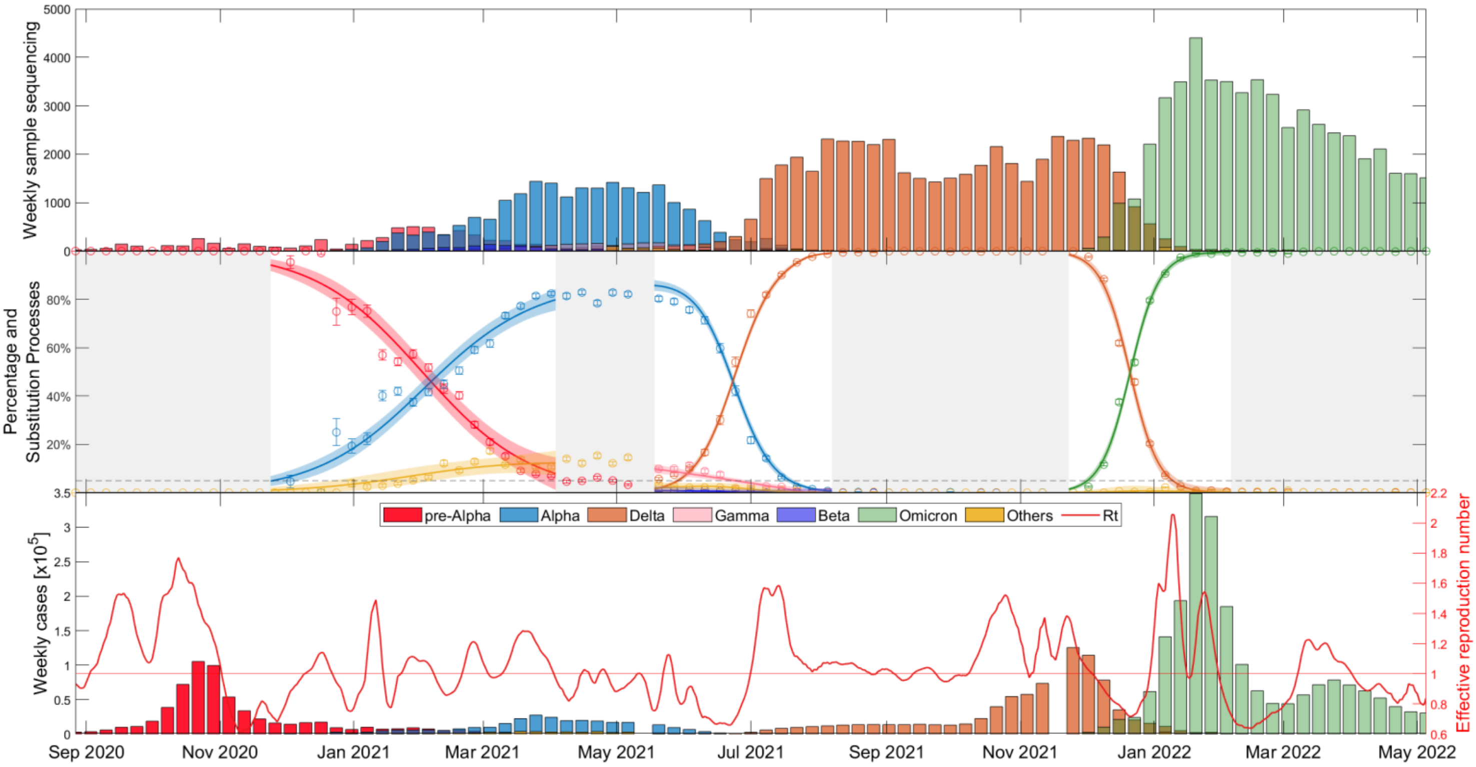

Figure S3 Dynamics evolution of COVID-19 main lineages substitutions (Alpha, Delta, and Omicron) in Belgium over time.

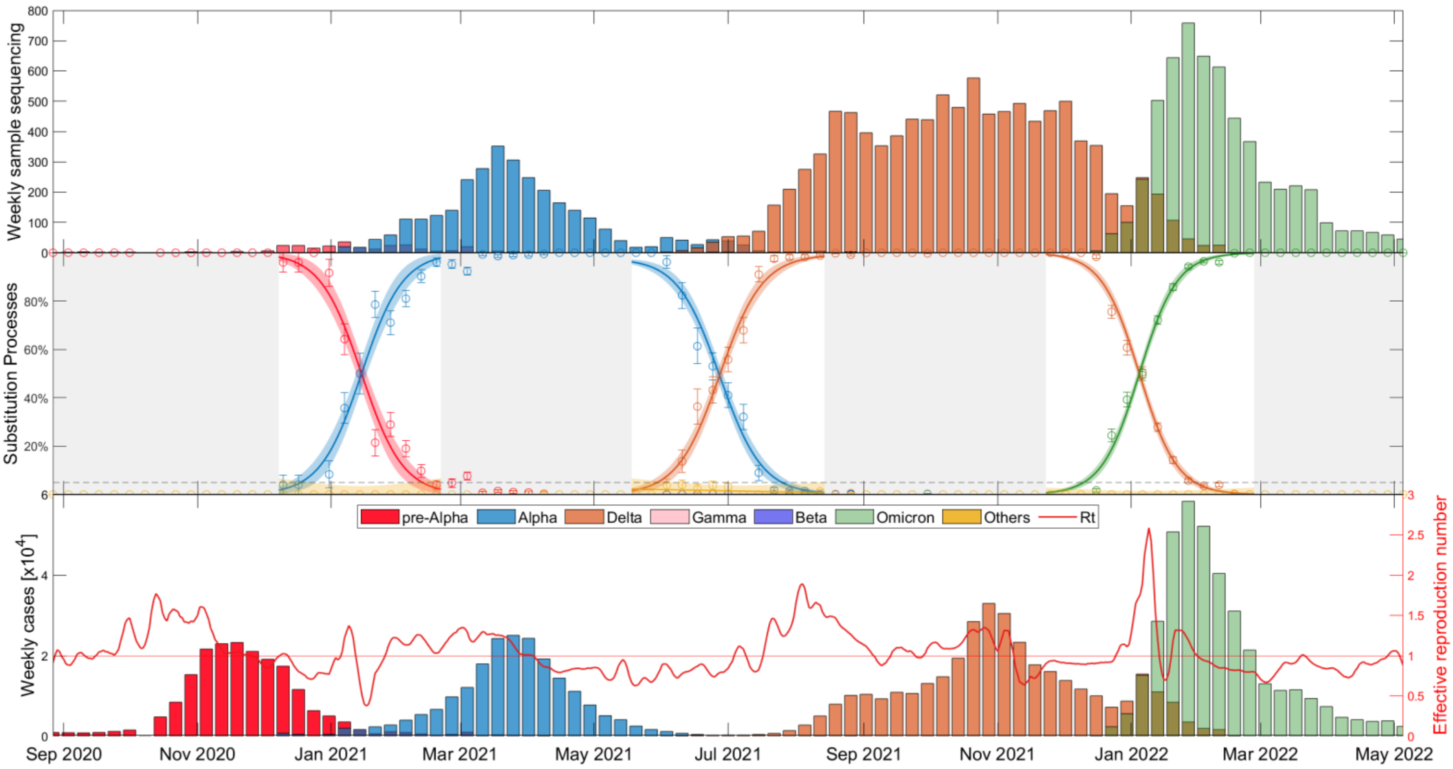

Figure S4 Dynamics evolution of COVID-19 main lineages substitutions (Alpha, Delta, and Omicron) in Bulgaria over time.

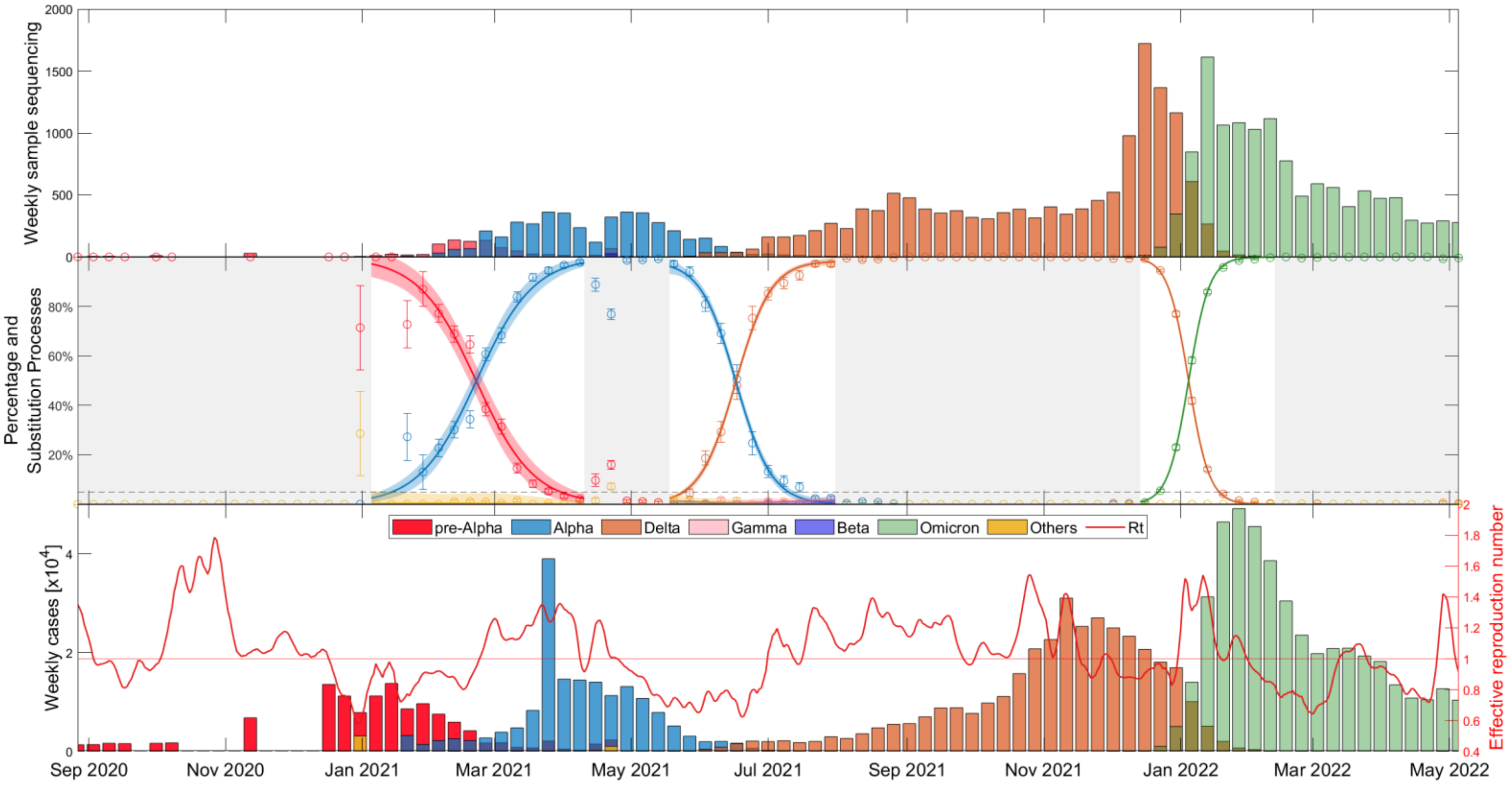

Figure S5 Dynamics evolution of COVID-19 main lineages substitutions (Alpha, Delta, and Omicron) in Croatia over time.

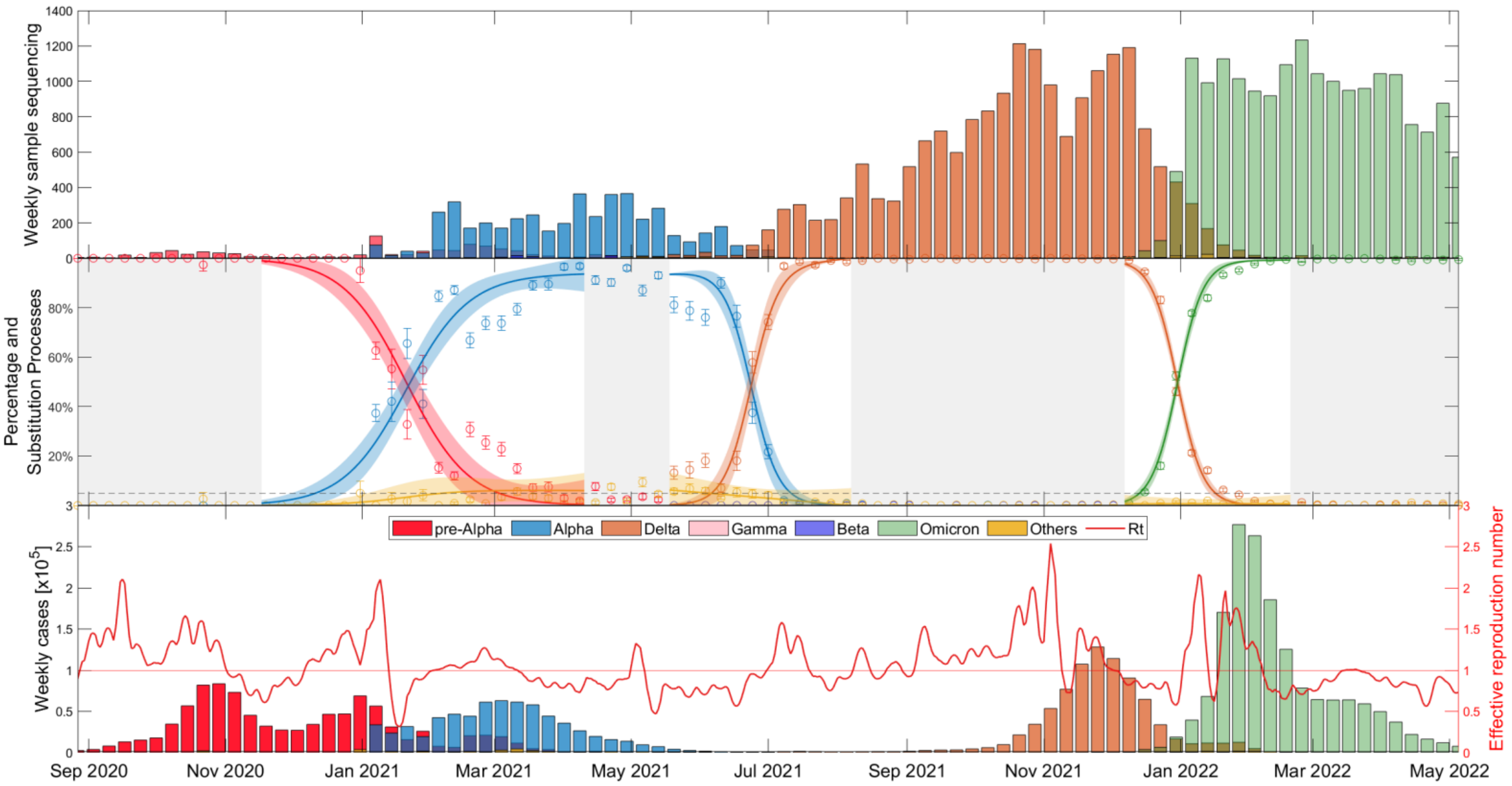

Figure S6 Dynamics evolution of COVID-19 main lineages substitutions (Alpha, Delta, and Omicron) in Czechia over time.

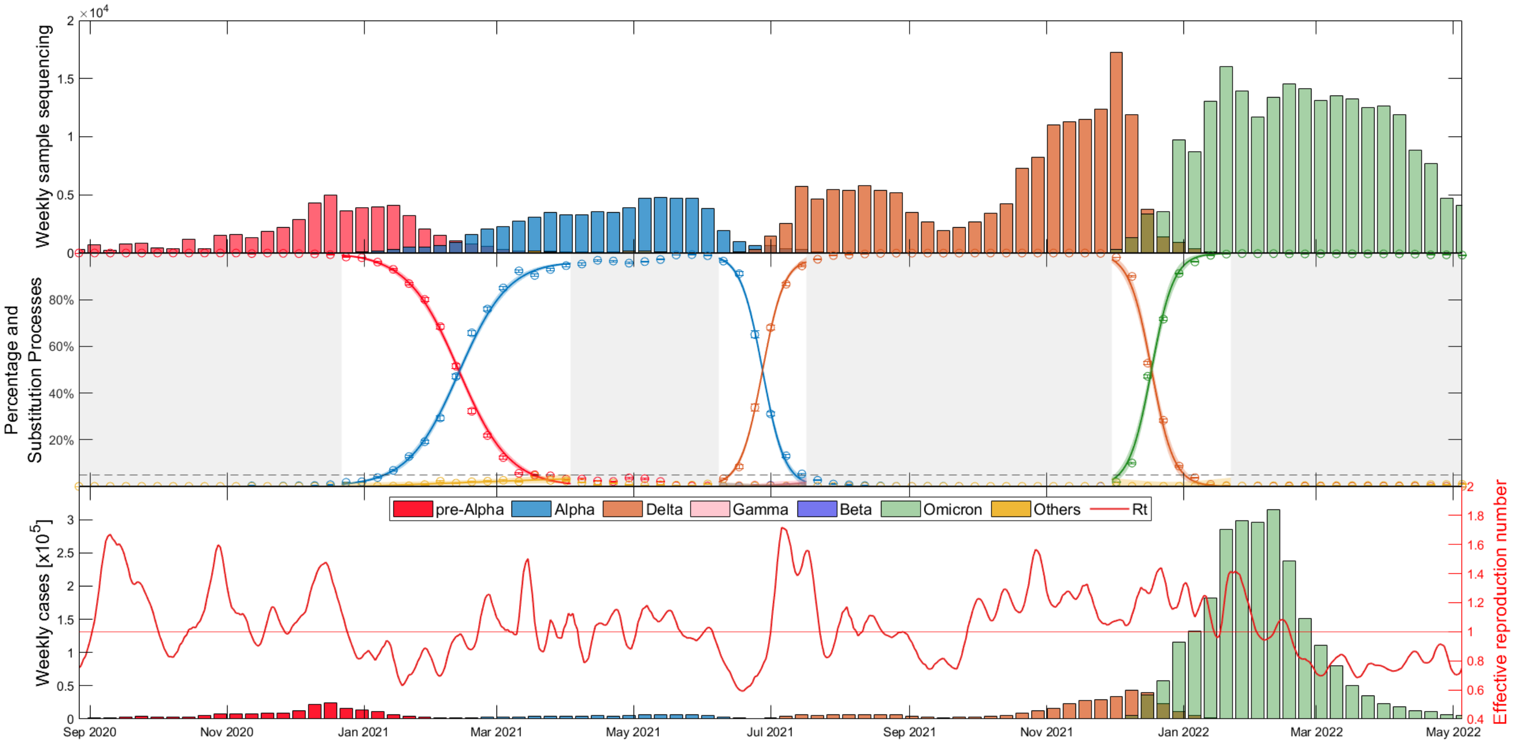

Figure S7 Dynamics evolution of COVID-19 main lineages substitutions (Alpha, Delta, and Omicron) in Denmark over time.

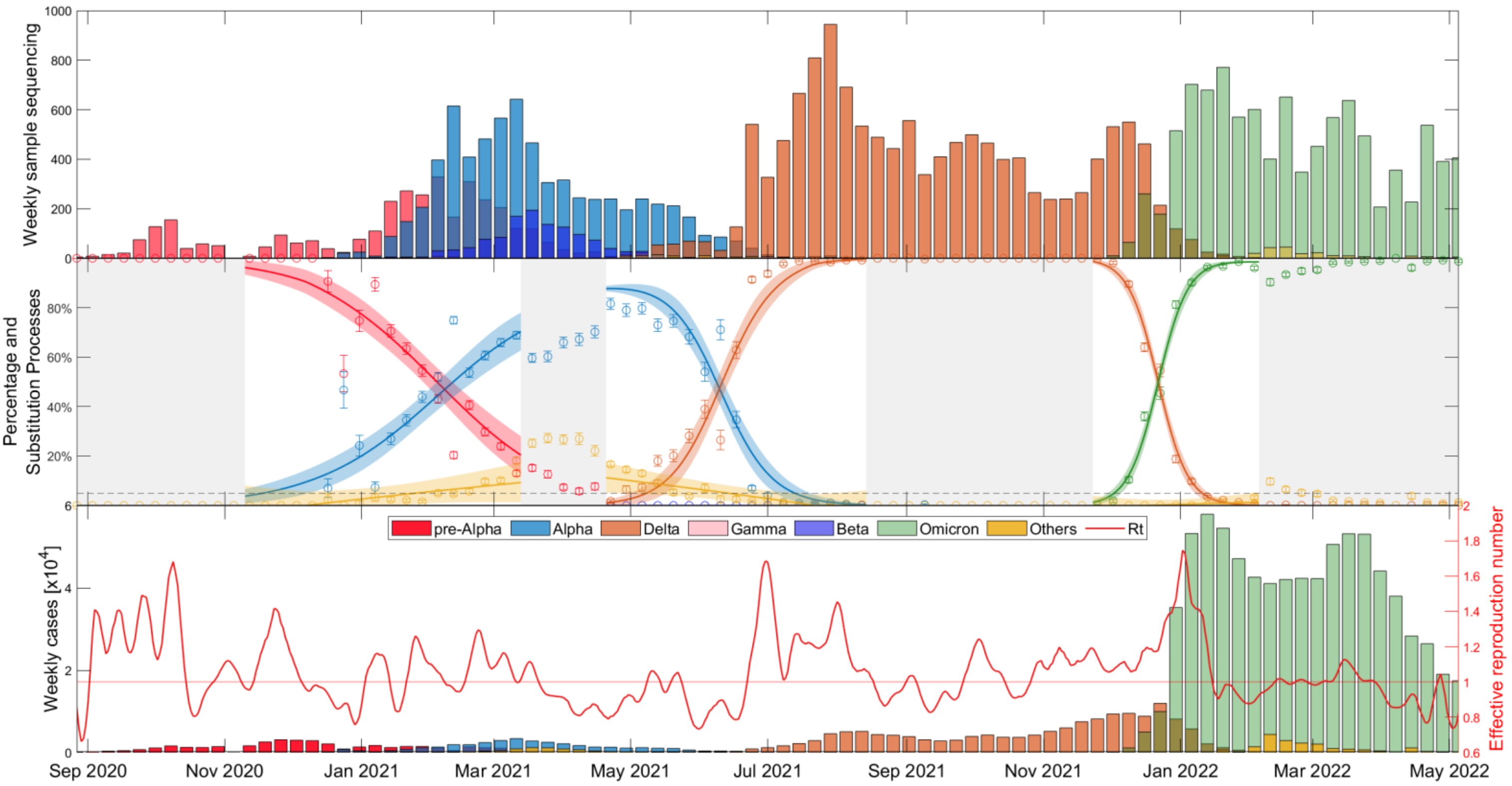

Figure S8 Dynamics evolution of COVID-19 main lineages substitutions (Alpha, Delta, and Omicron) in Finland over time.

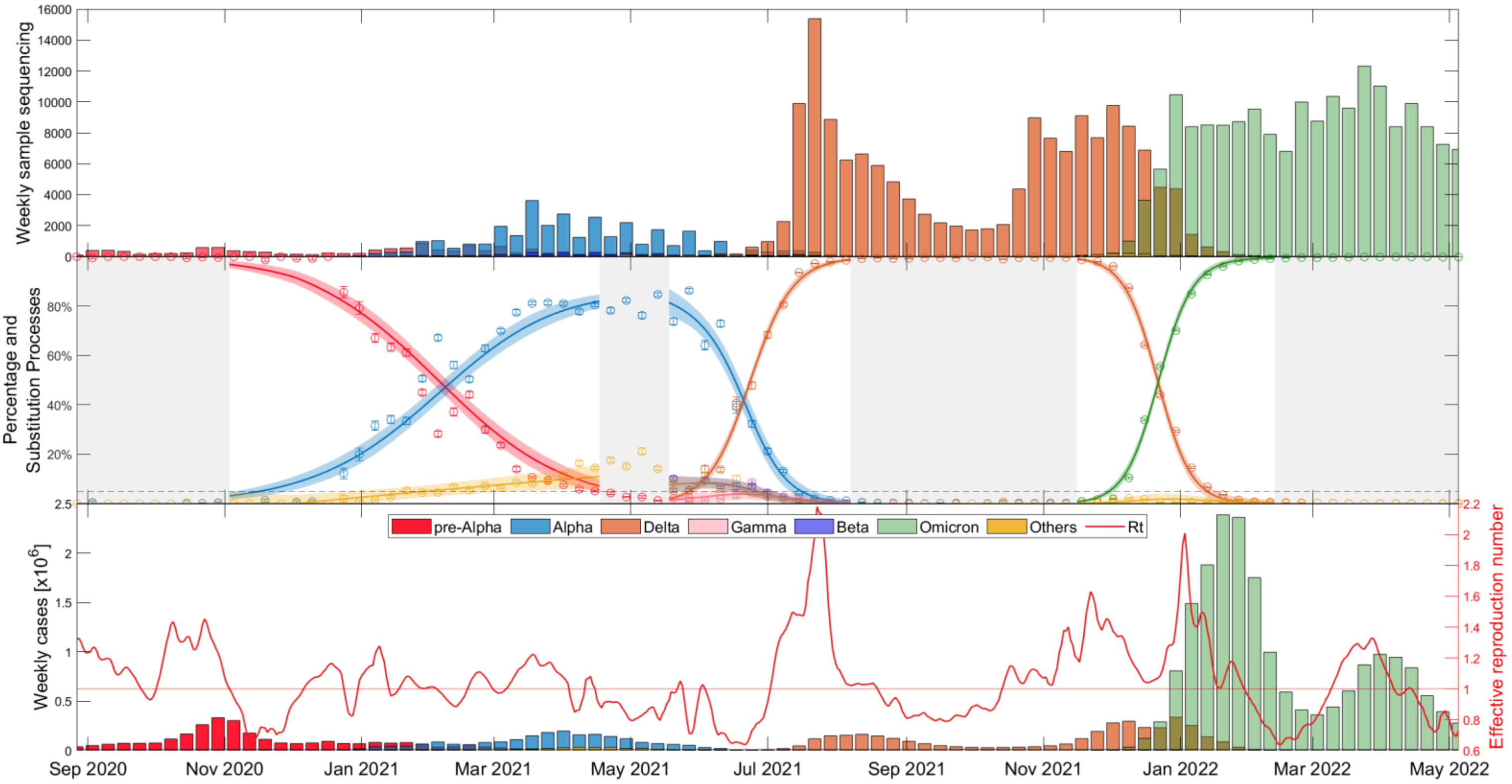

Figure S9 Dynamics evolution of COVID-19 main lineages substitutions (Alpha, Delta, and Omicron) in France over time.

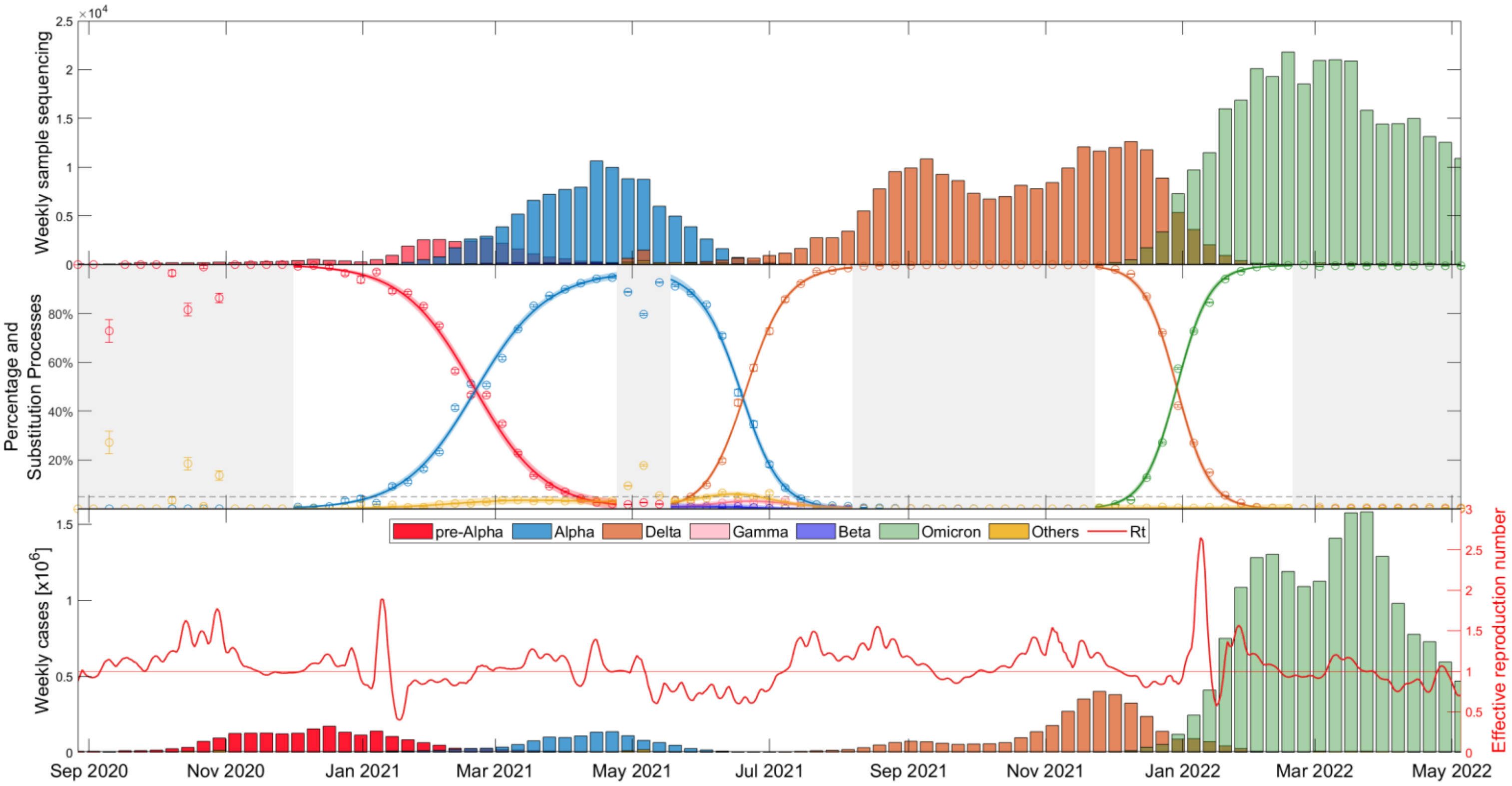

Figure S10 Dynamics evolution of COVID-19 main lineages substitutions (Alpha, Delta, and Omicron) in Germany over time.

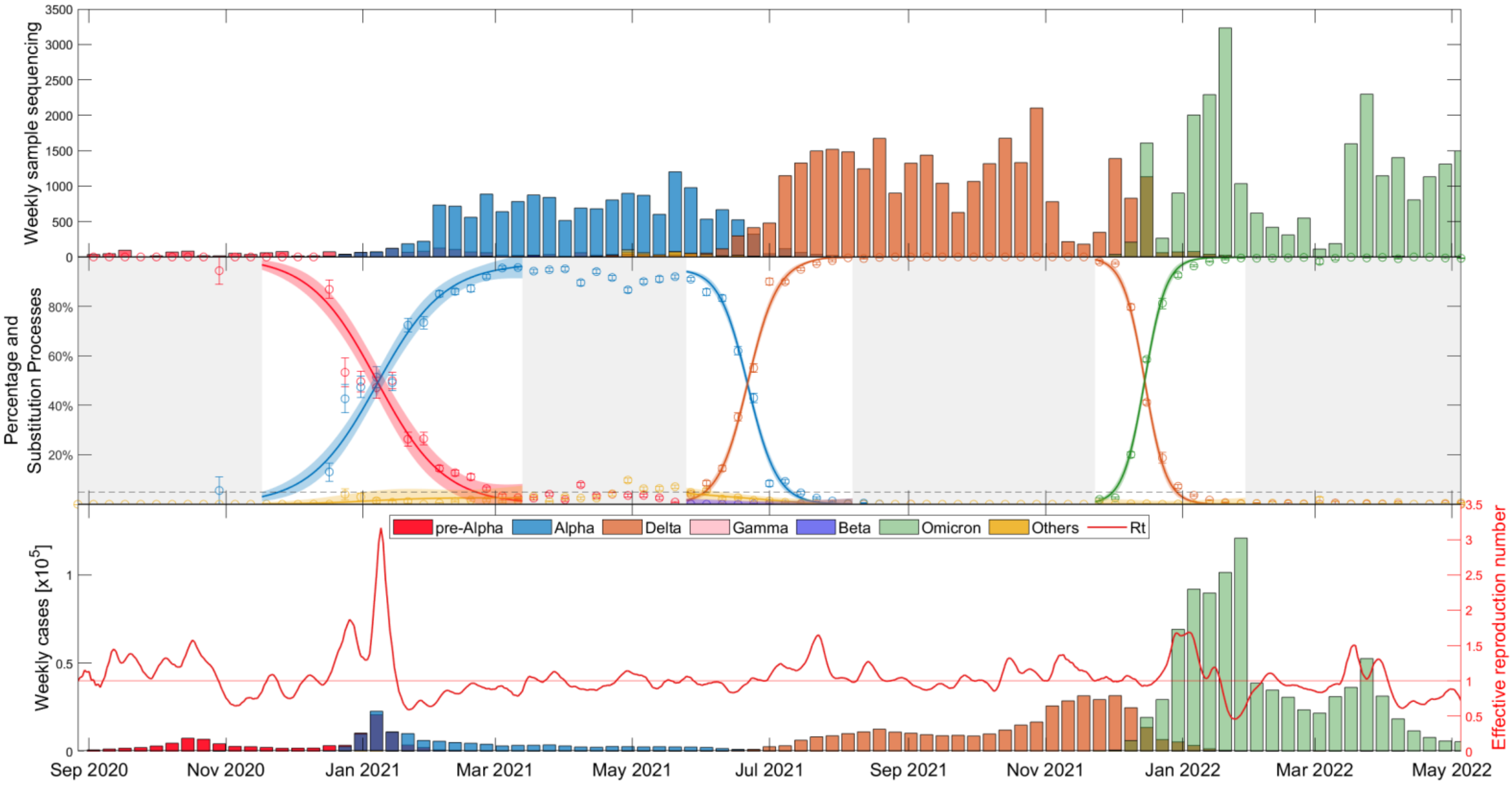

Figure S11 Dynamics evolution of COVID-19 main lineages substitutions (Alpha, Delta, and Omicron) in Ireland over time.

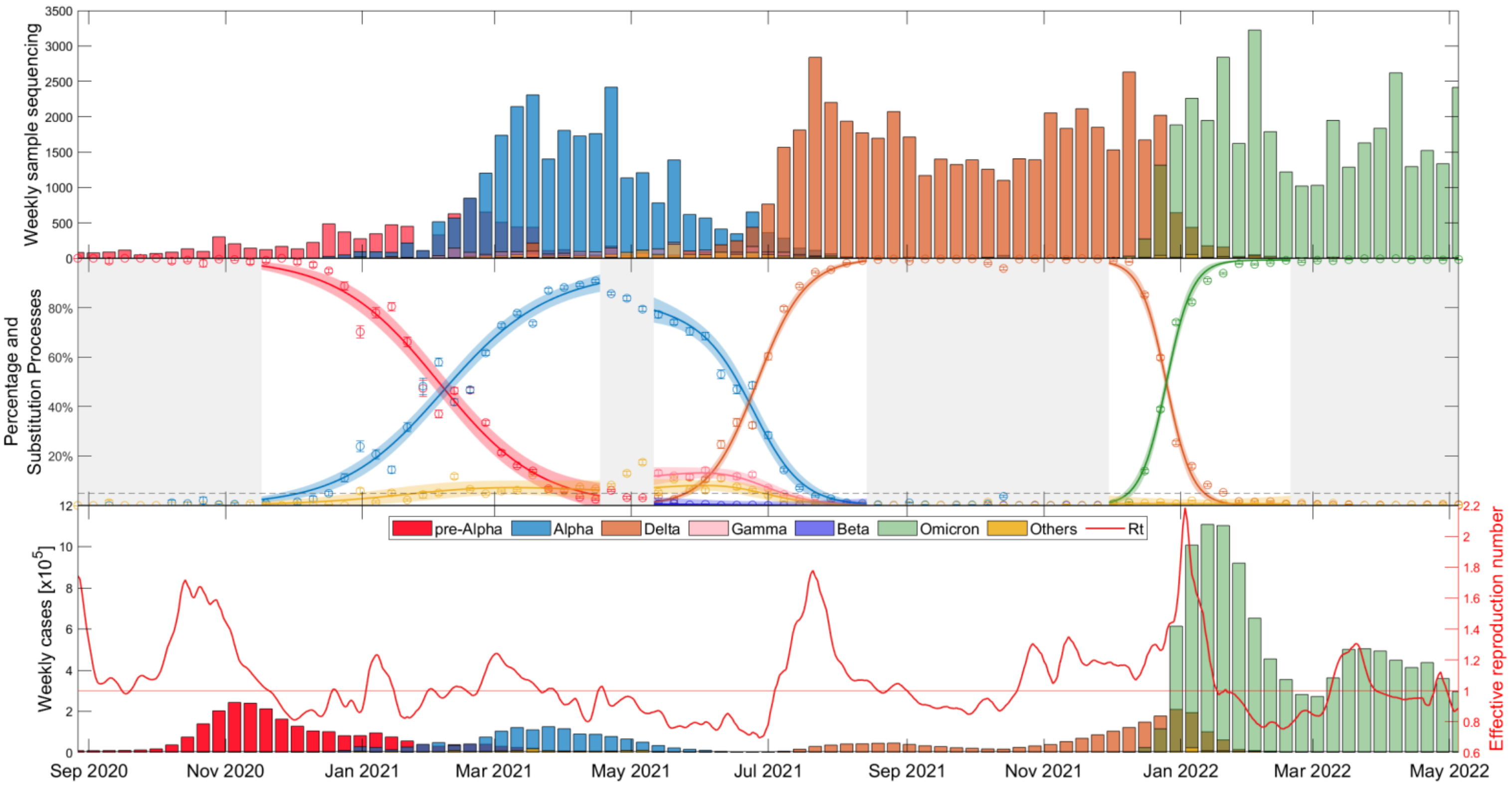

Figure S12 Dynamics evolution of COVID-19 main lineages substitutions (Alpha, Delta, and Omicron) in Italy over time.

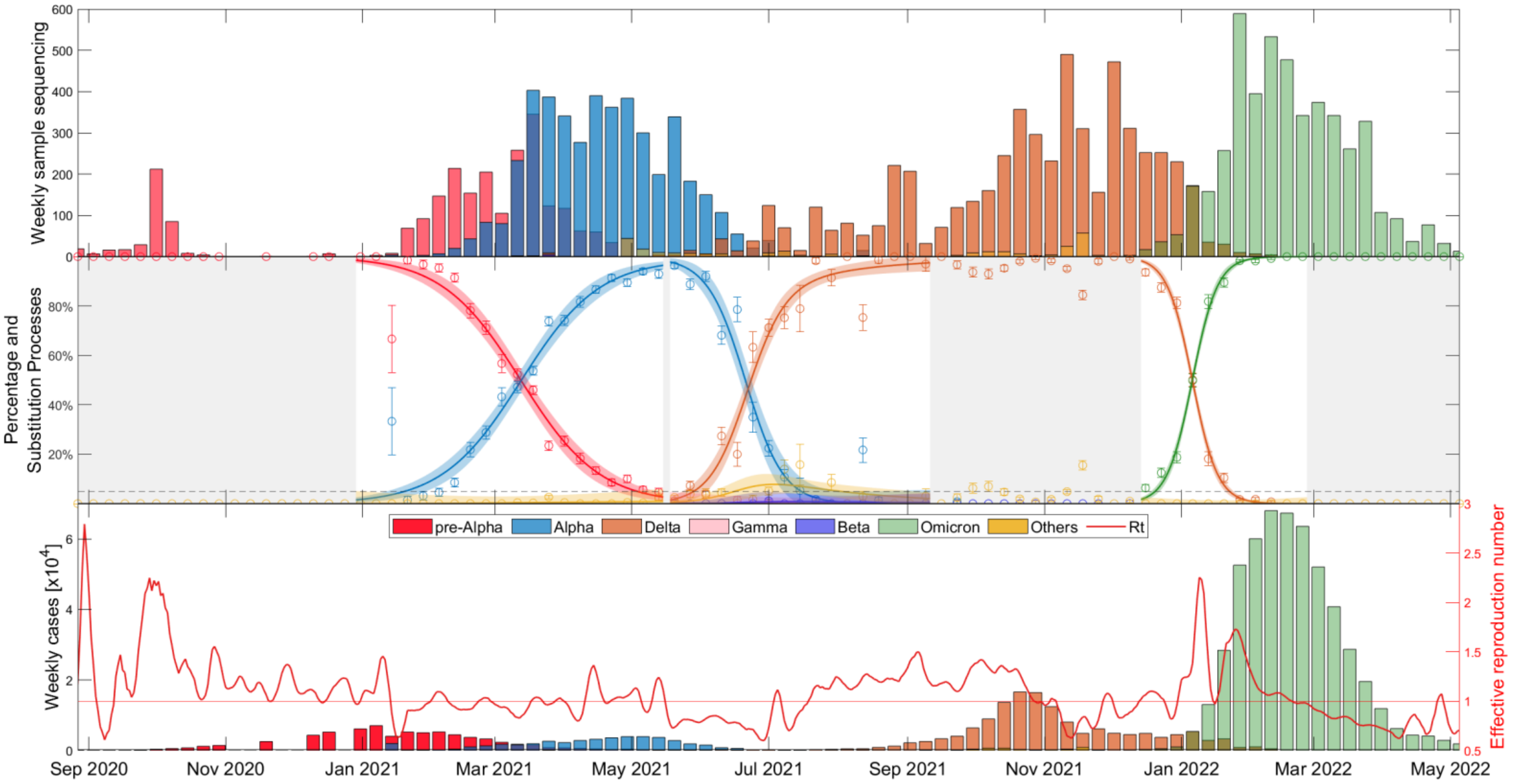

Figure S13 Dynamics evolution of COVID-19 main lineages substitutions (Alpha, Delta, and Omicron) in Latvia over time.

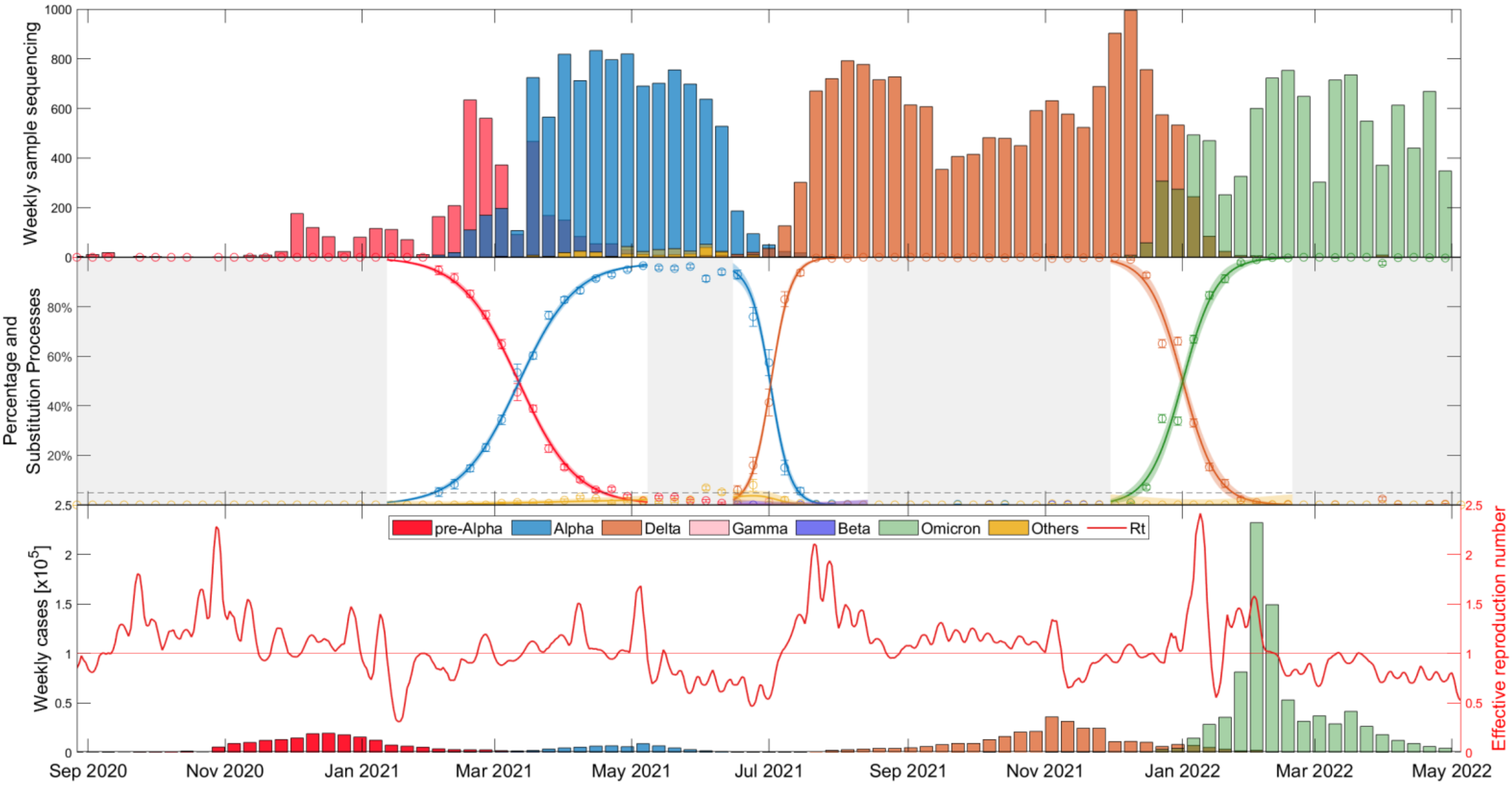

Figure S14 Dynamics evolution of COVID-19 main lineages substitutions (Alpha, Delta, and Omicron) in Lithuania over time.

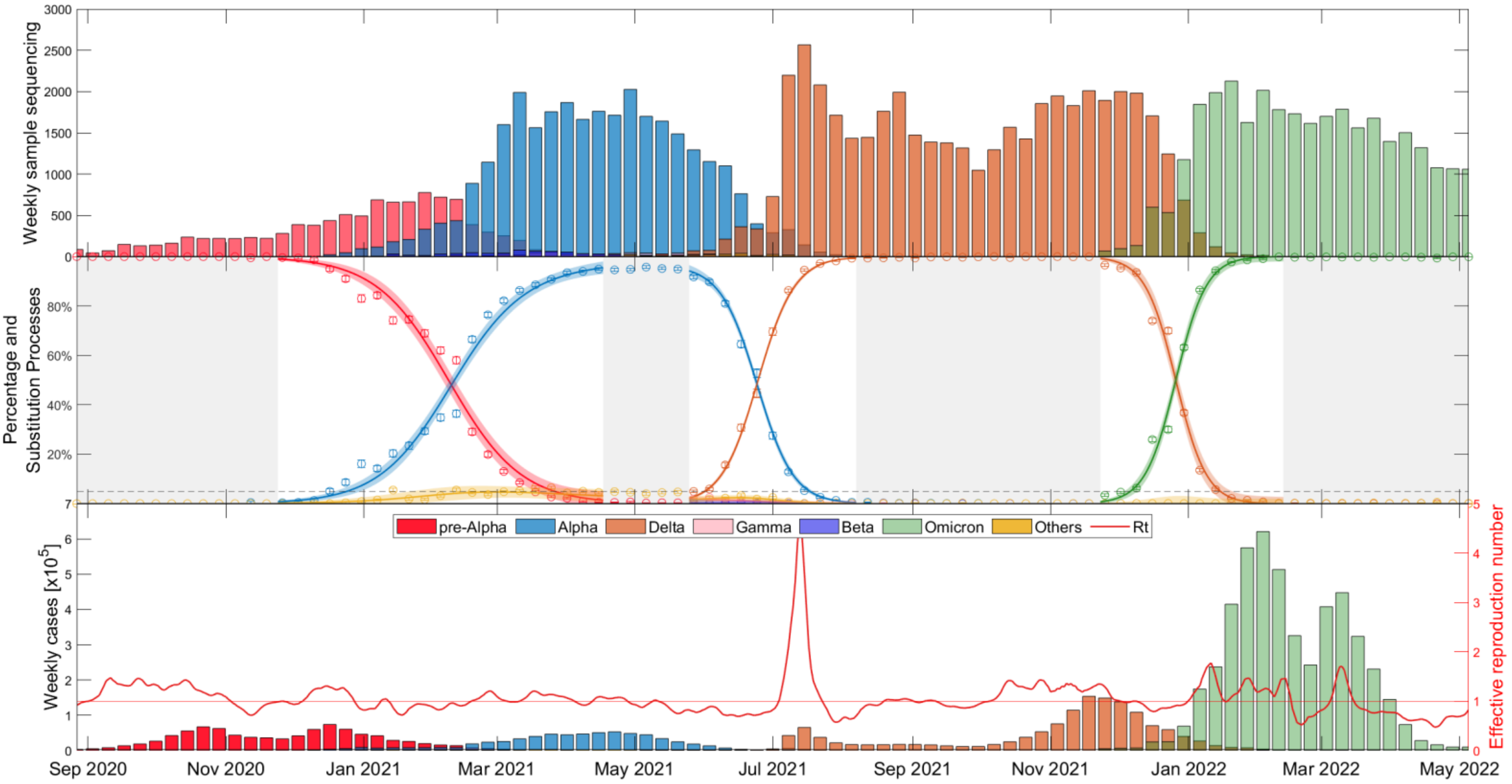

Figure S15 Dynamics evolution of COVID-19 main lineages substitutions (Alpha, Delta, and Omicron) in Netherlands over time.

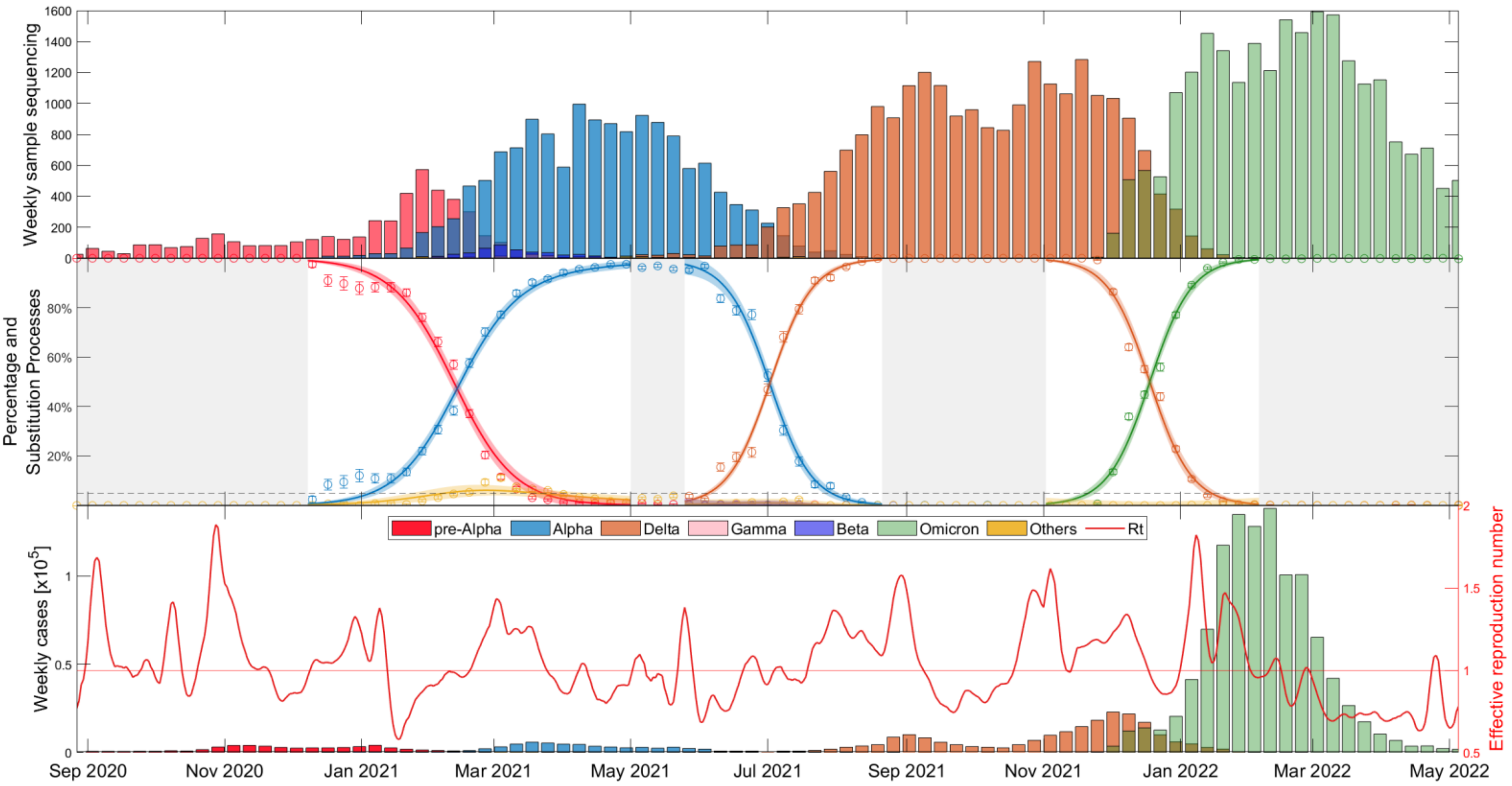

Figure S16 Dynamics evolution of COVID-19 main lineages substitutions (Alpha, Delta, and Omicron) in Norway over time.

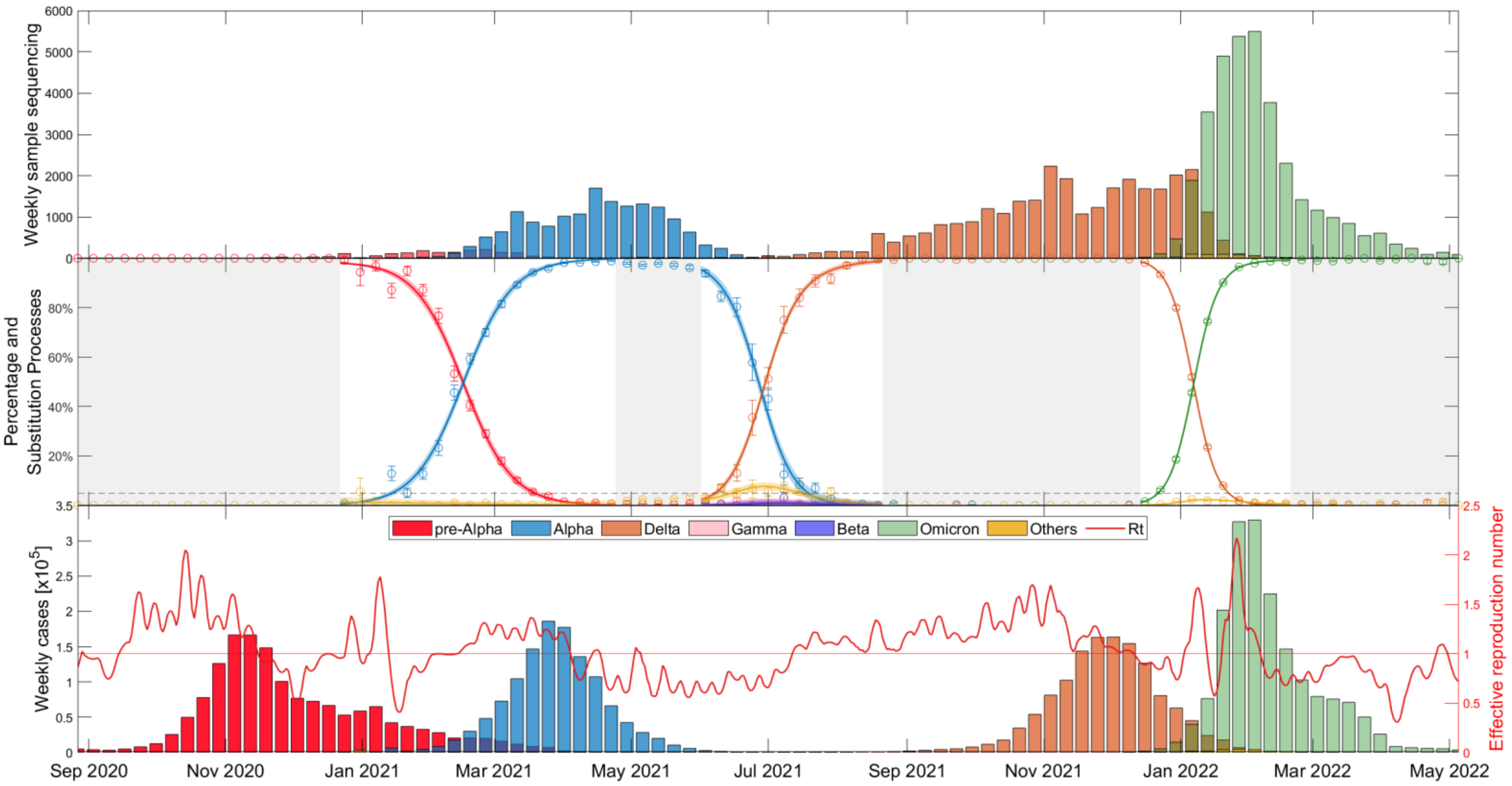

Figure S17 Dynamics evolution of COVID-19 main lineages substitutions (Alpha, Delta, and Omicron) in Poland over time.

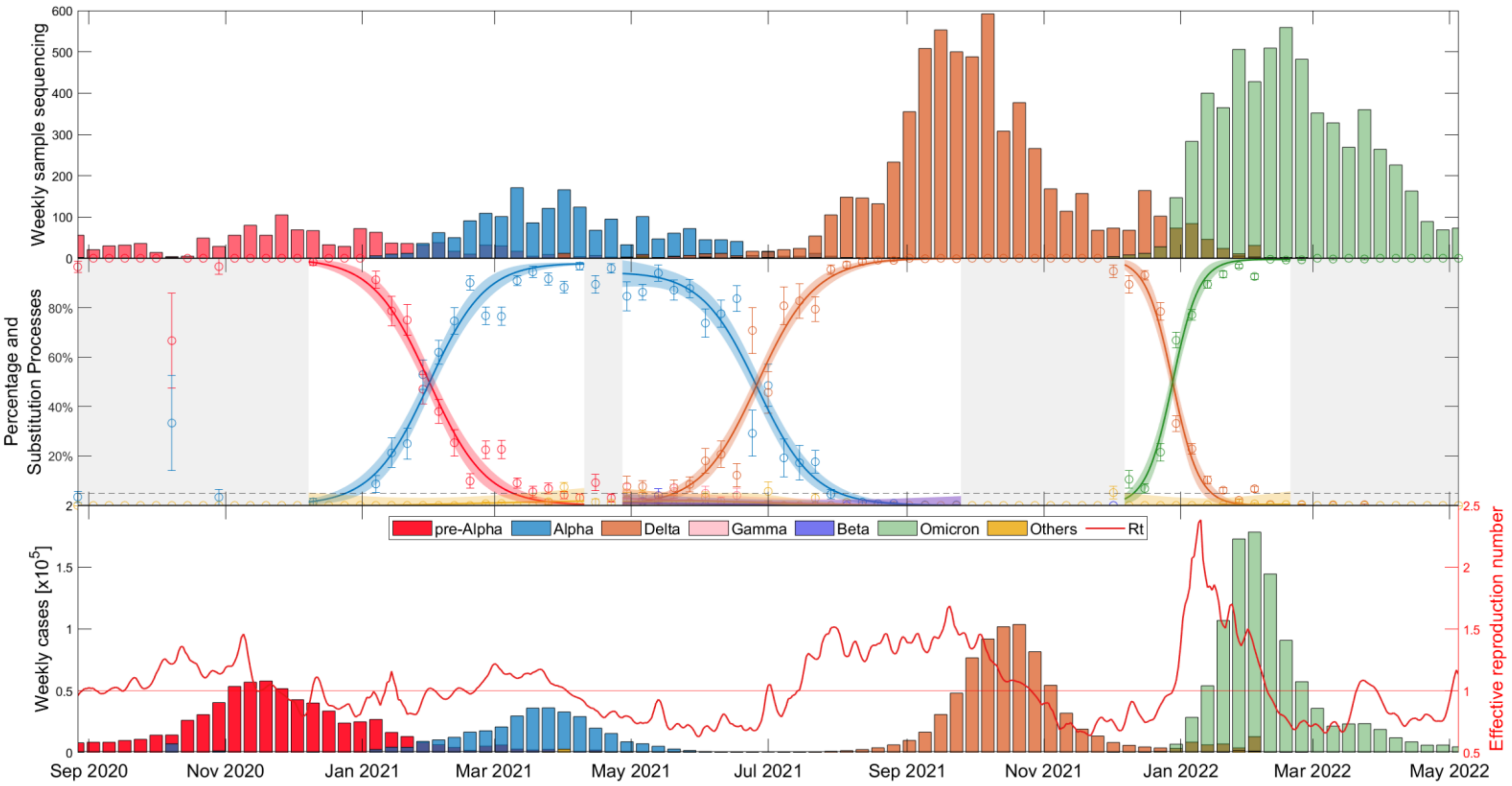

Figure S18 Dynamics evolution of COVID-19 main lineages substitutions (Alpha, Delta, and Omicron) in Romania over time.

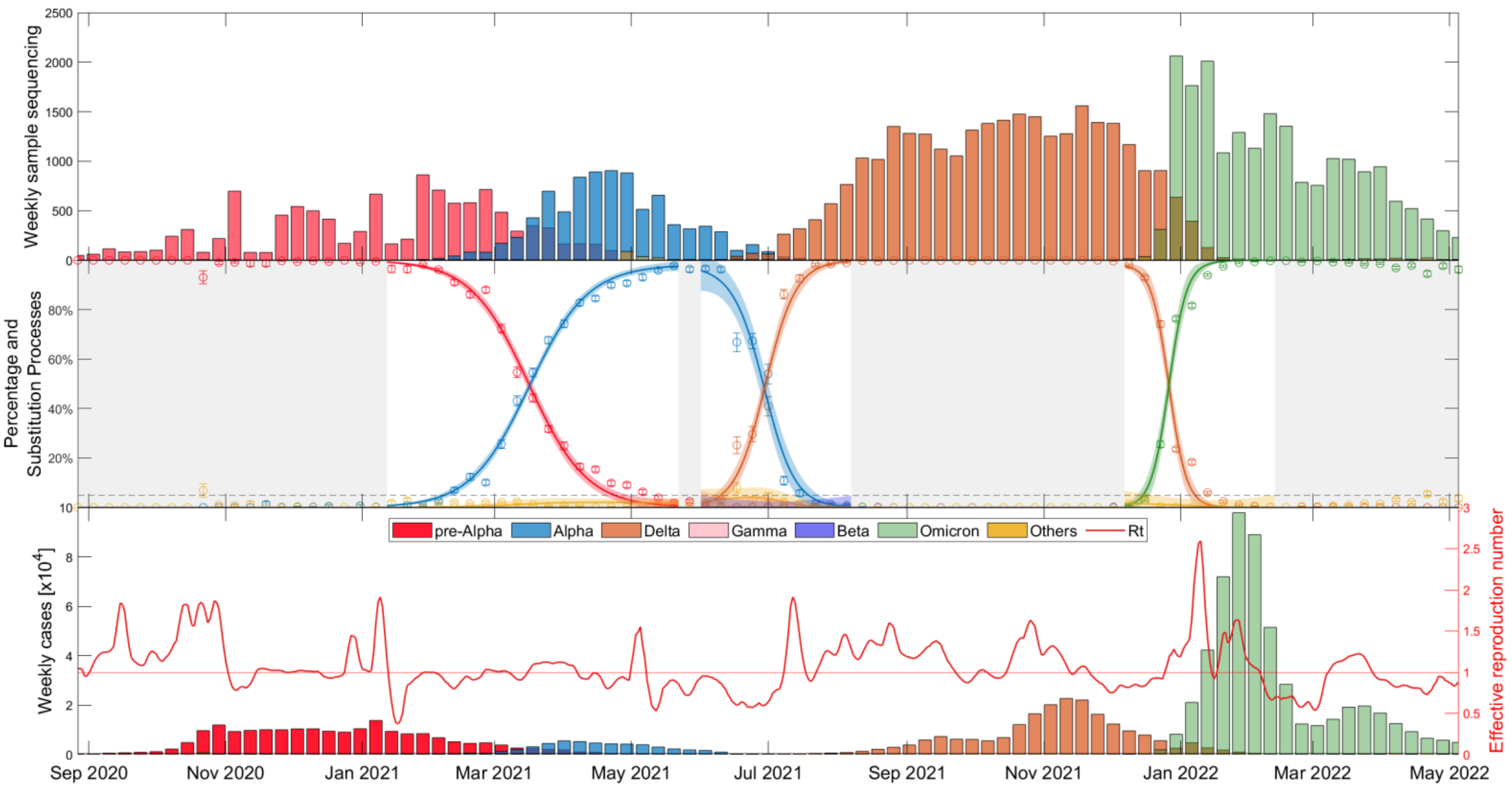

Figure S19 Dynamics evolution of COVID-19 main lineages substitutions (Alpha, Delta, and Omicron) in Slovenia over time.

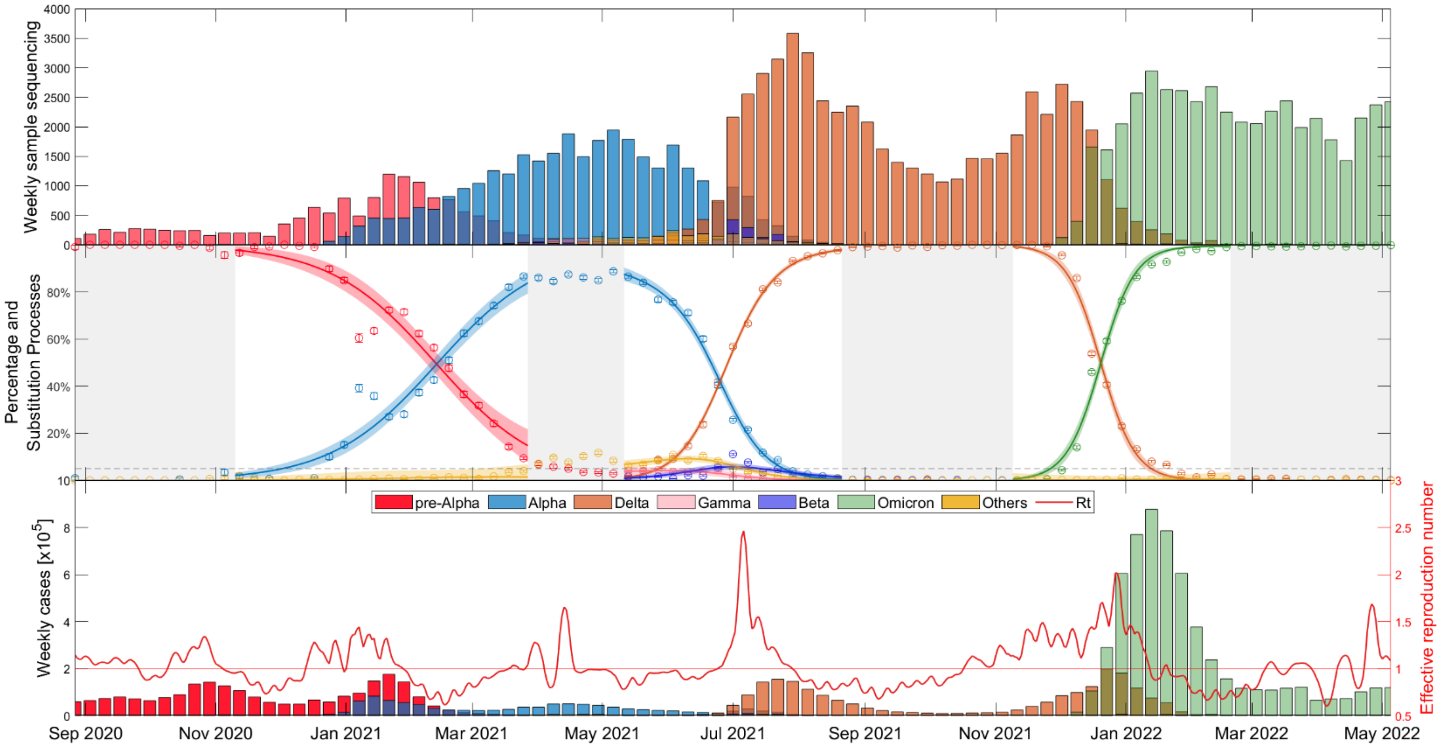

Figure S20 Dynamics evolution of COVID-19 main lineages substitutions (Alpha, Delta, and Omicron) in Spain over time. This is the same figure as Figure 1 in the main text.

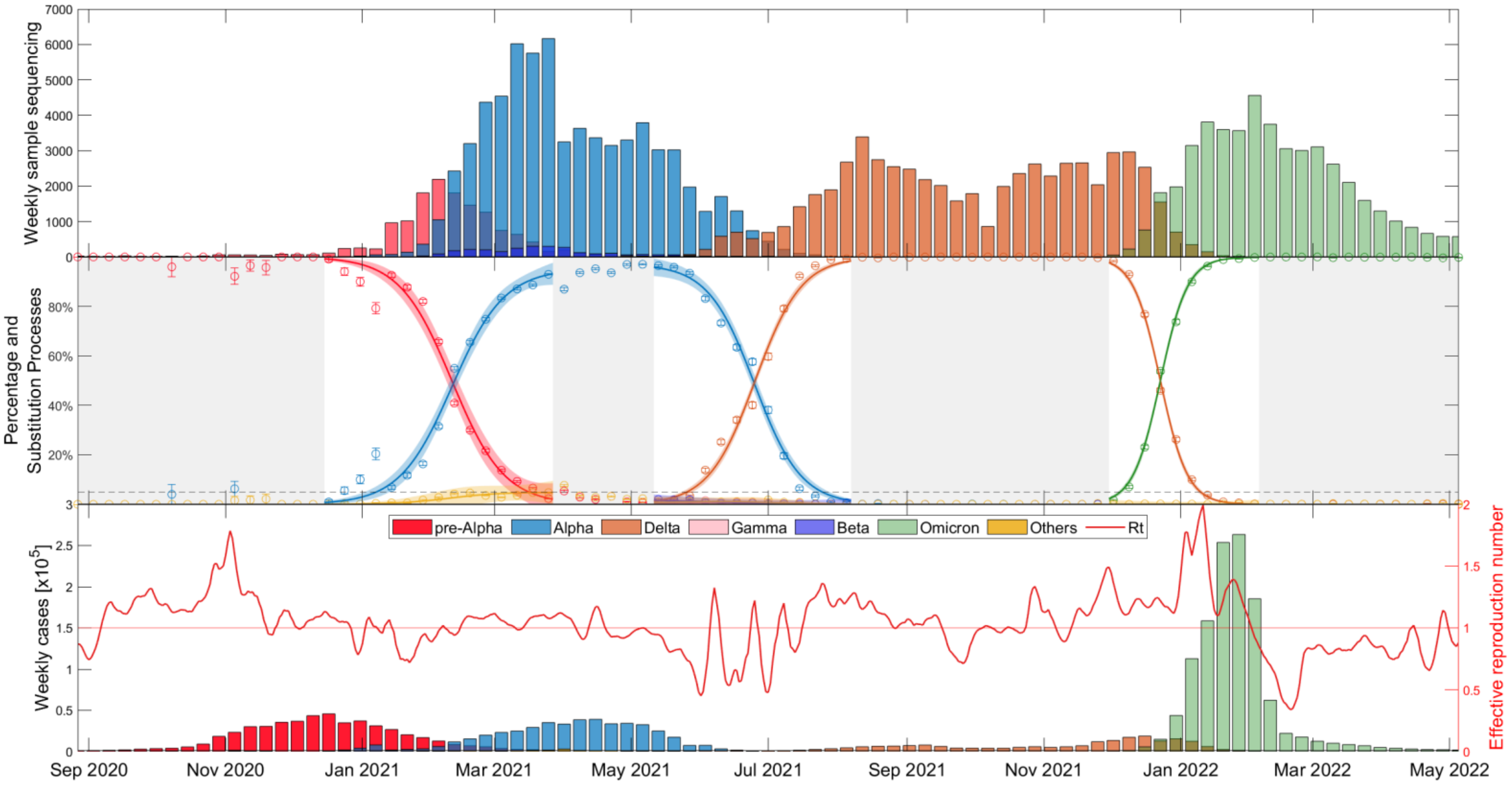

Figure S21 Dynamics evolution of COVID-19 main lineages substitutions (Alpha, Delta, and Omicron) in Sweden over time.

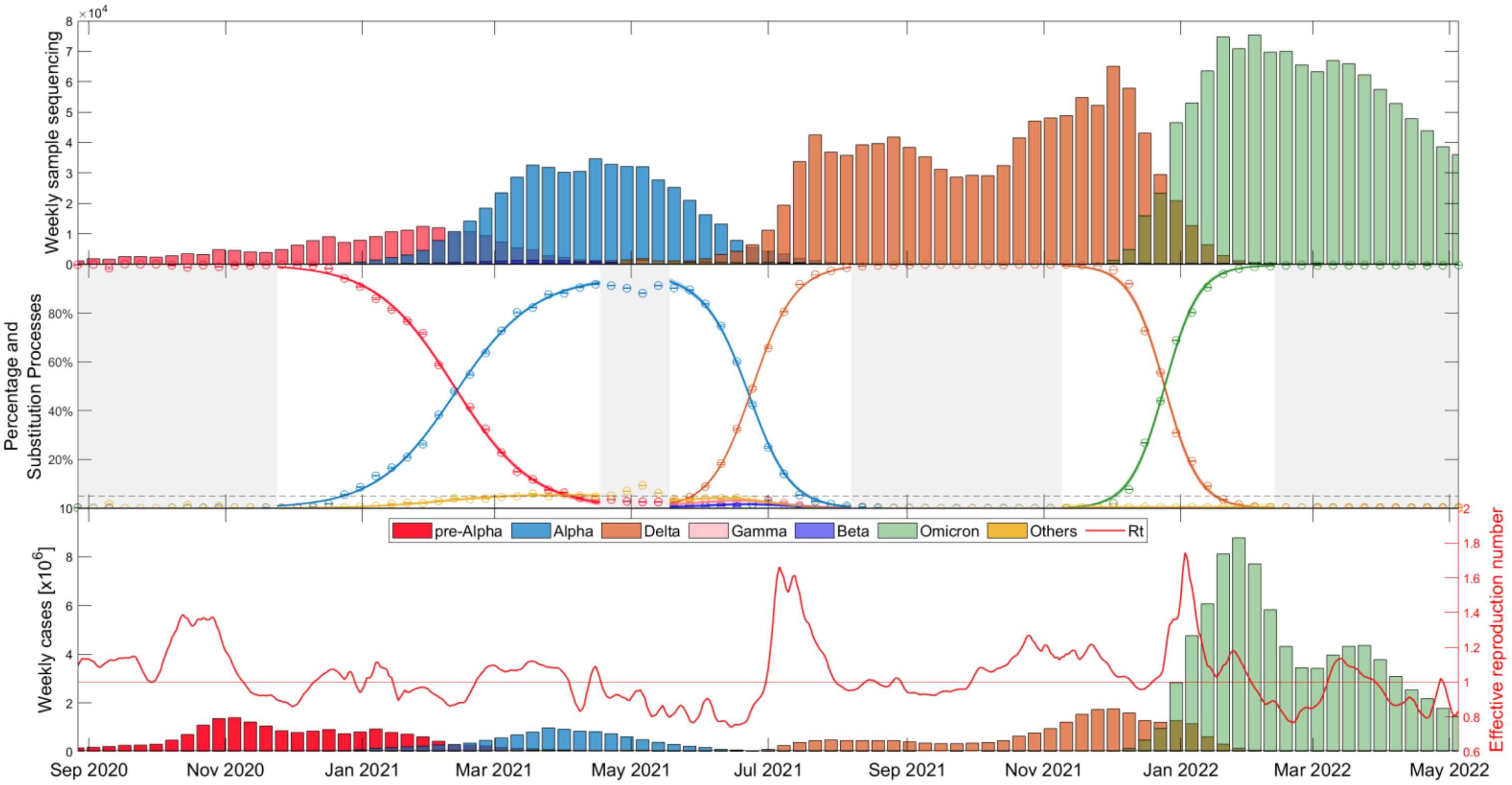

Figure S22 Dynamics evolution of COVID-19 main lineages substitutions (Alpha, Delta, and Omicron) in Europe over time.

Suppl. Material Text S6 – Substitutions dynamics with Omicron lineages for each country with GISAID data source

In this section, we extend the analysis presented in Figure 2 from the main manuscript to include the 18 countries and Europe. We examine the substitutions of the Alpha, Delta, and various Omicron lineages (BA.1, BA.2, BA.5, and BQ.1).

Please note that Lithuania is not represented due to the absence of data from May to November 2022, which encompasses the BA.2 to BA.5 and the BA.5 to BQ.1 substitution periods.

Further, Bulgaria stopped reporting data from November 2022 onward, meaning we could not account for the BA.5 to BQ.1 substitution in this country. Therefore, Figure S24 does not display any data for Bulgaria beyond this date.

Numerical results can be found in Supplementary Material Table 6.

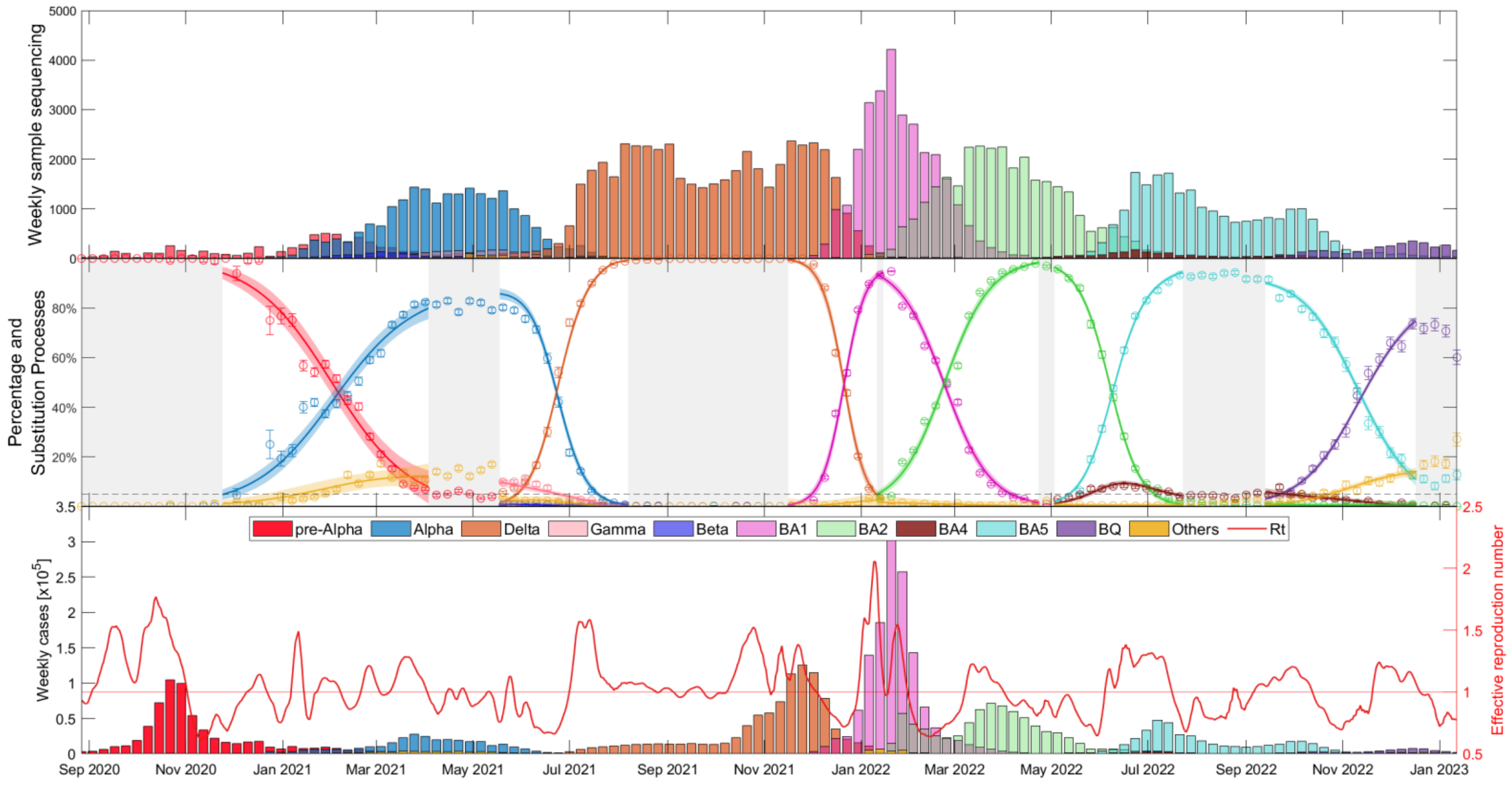

Figure S23 Evolutionary dynamics of Alpha, Delta, and Omicron lineages (BA.1, BA.2, BA.5, and BQ.1) substitutions in Belgium over time.

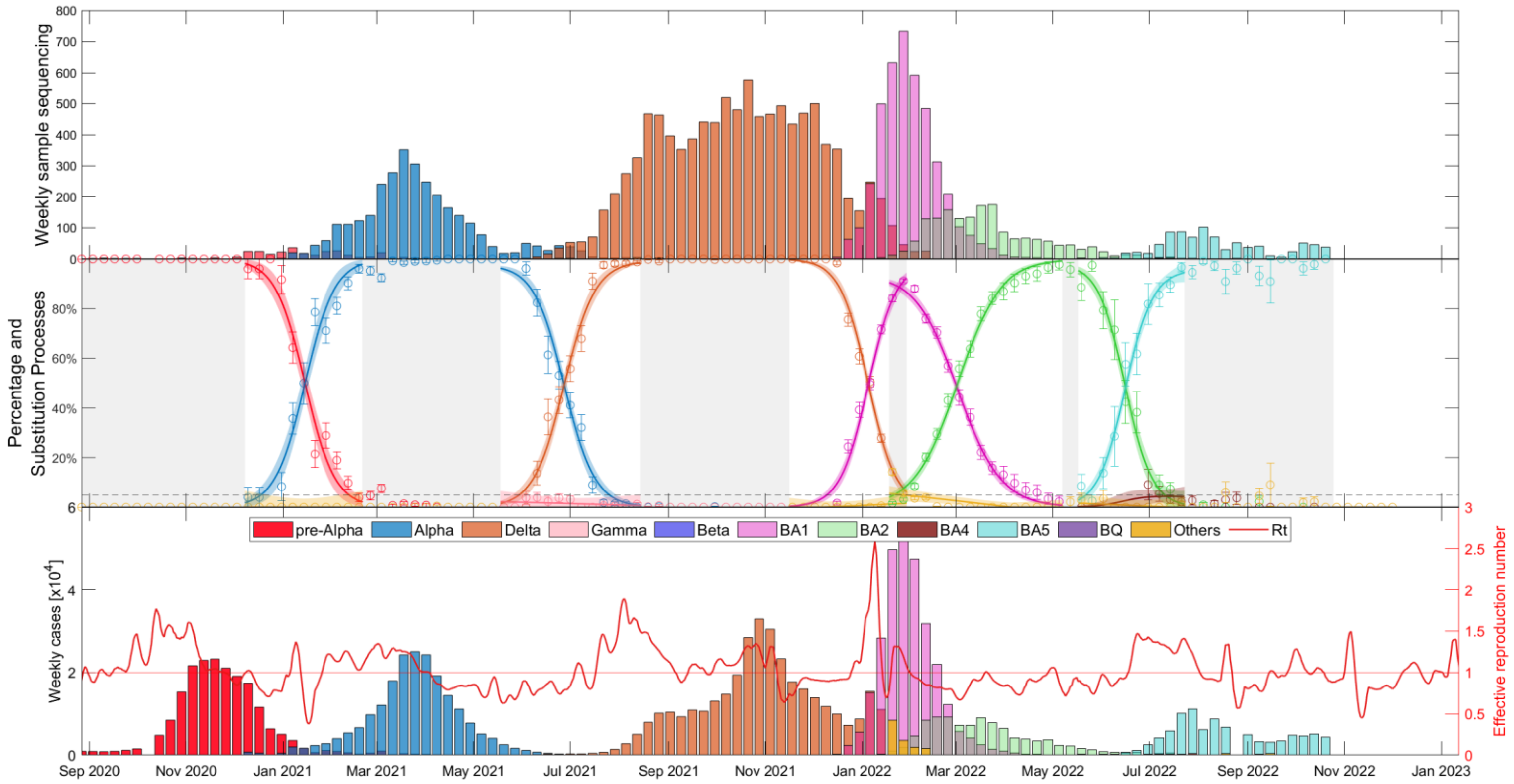

Figure S24 Evolutionary dynamics of Alpha, Delta, and Omicron lineages (BA.1, BA.2, BA.5, and BQ.1) substitutions in Bulgaria over time.

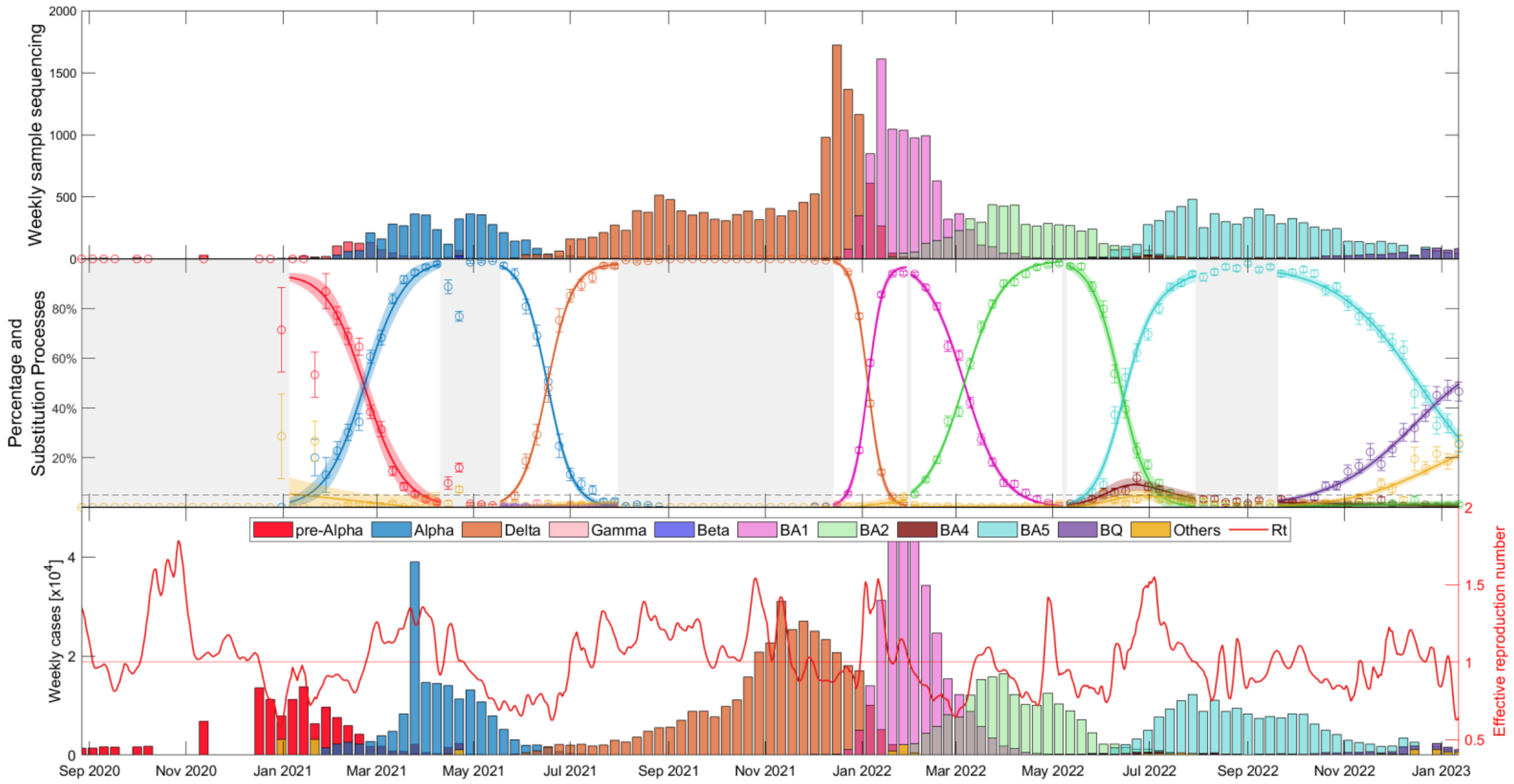

Figure S25 Evolutionary dynamics of Alpha, Delta, and Omicron lineages (BA.1, BA.2, BA.5, and BQ.1) substitutions in Croatia over time.

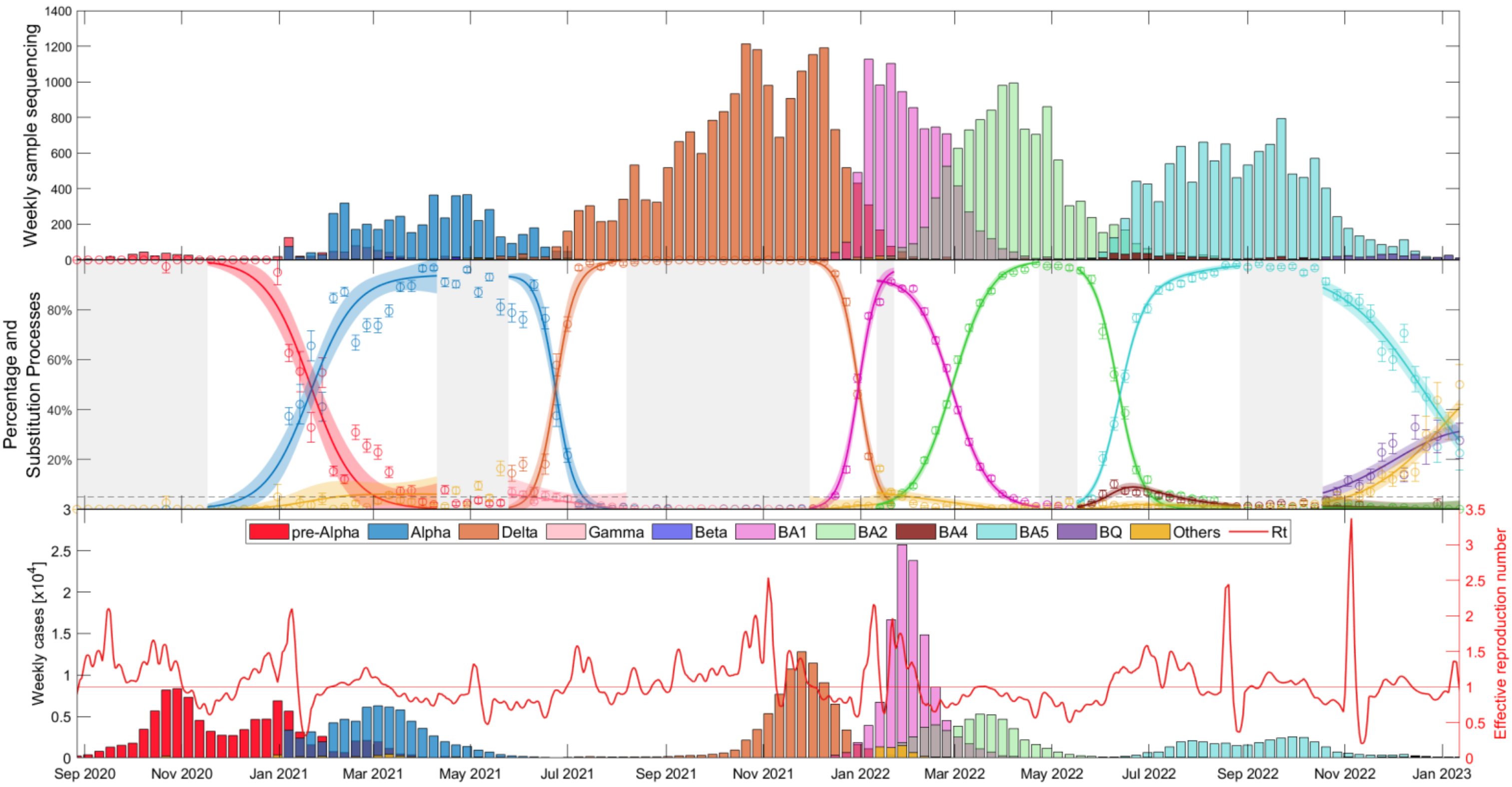

Figure S26 Evolutionary dynamics of Alpha, Delta, and Omicron lineages (BA.1, BA.2, BA.5, and BQ.1) substitutions in Czech Republic over time.

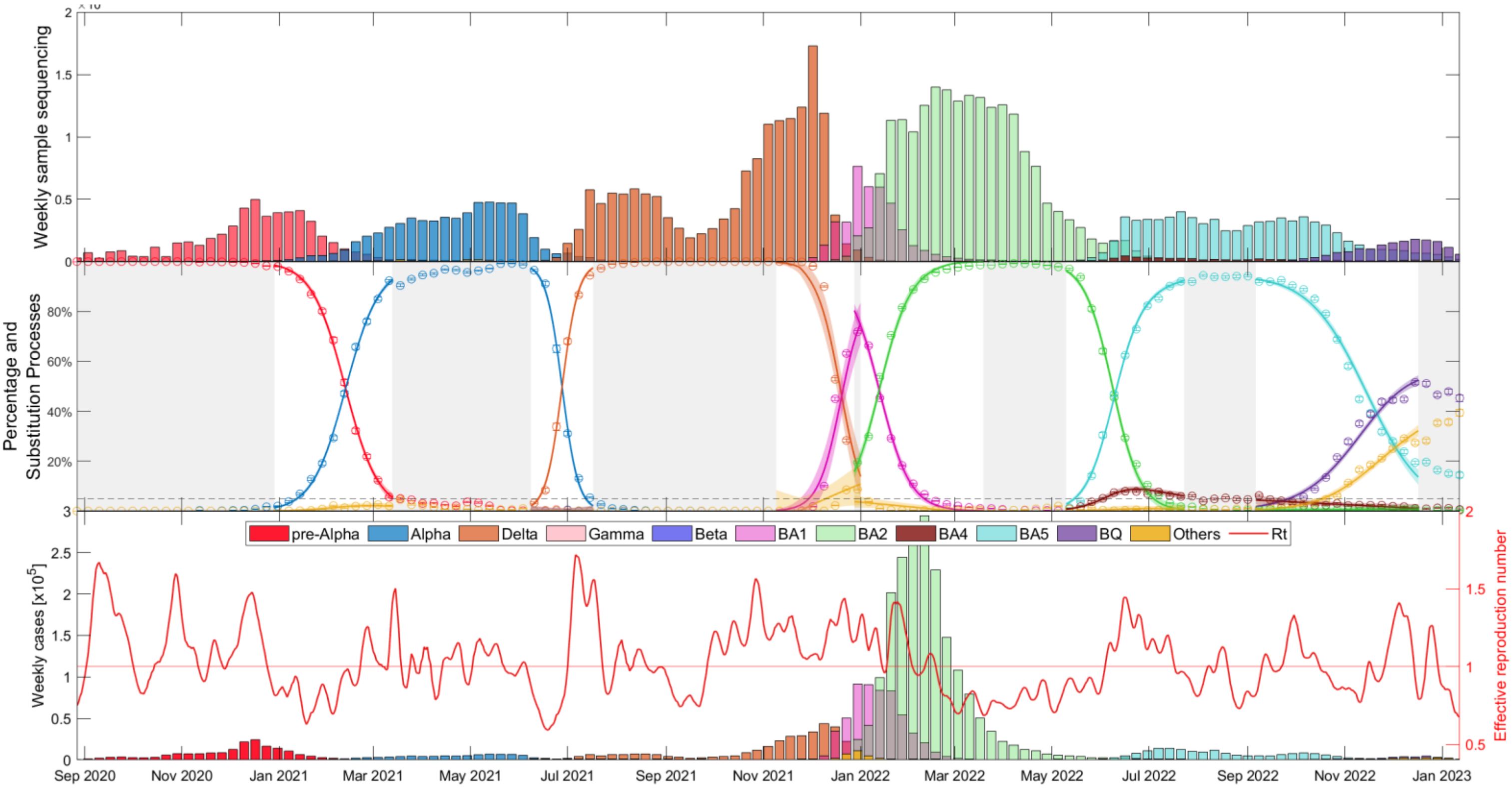

Figure S27 Evolutionary dynamics of Alpha, Delta, and Omicron lineages (BA.1, BA.2, BA.5, and BQ.1) substitutions in Denmark over time.

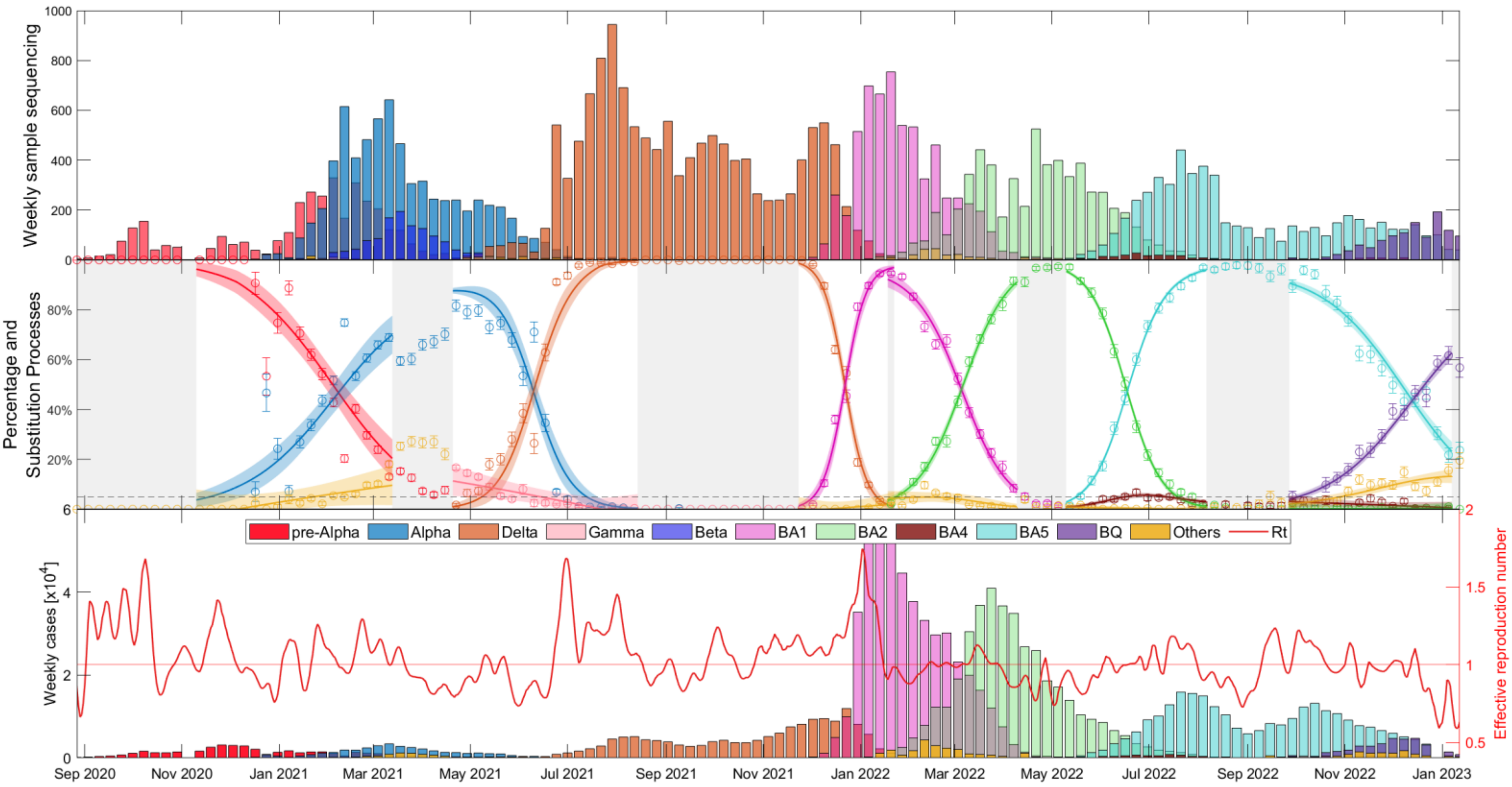

Figure S28 Evolutionary dynamics of Alpha, Delta, and Omicron lineages (BA.1, BA.2, BA.5, and BQ.1) substitutions in Finland over time.

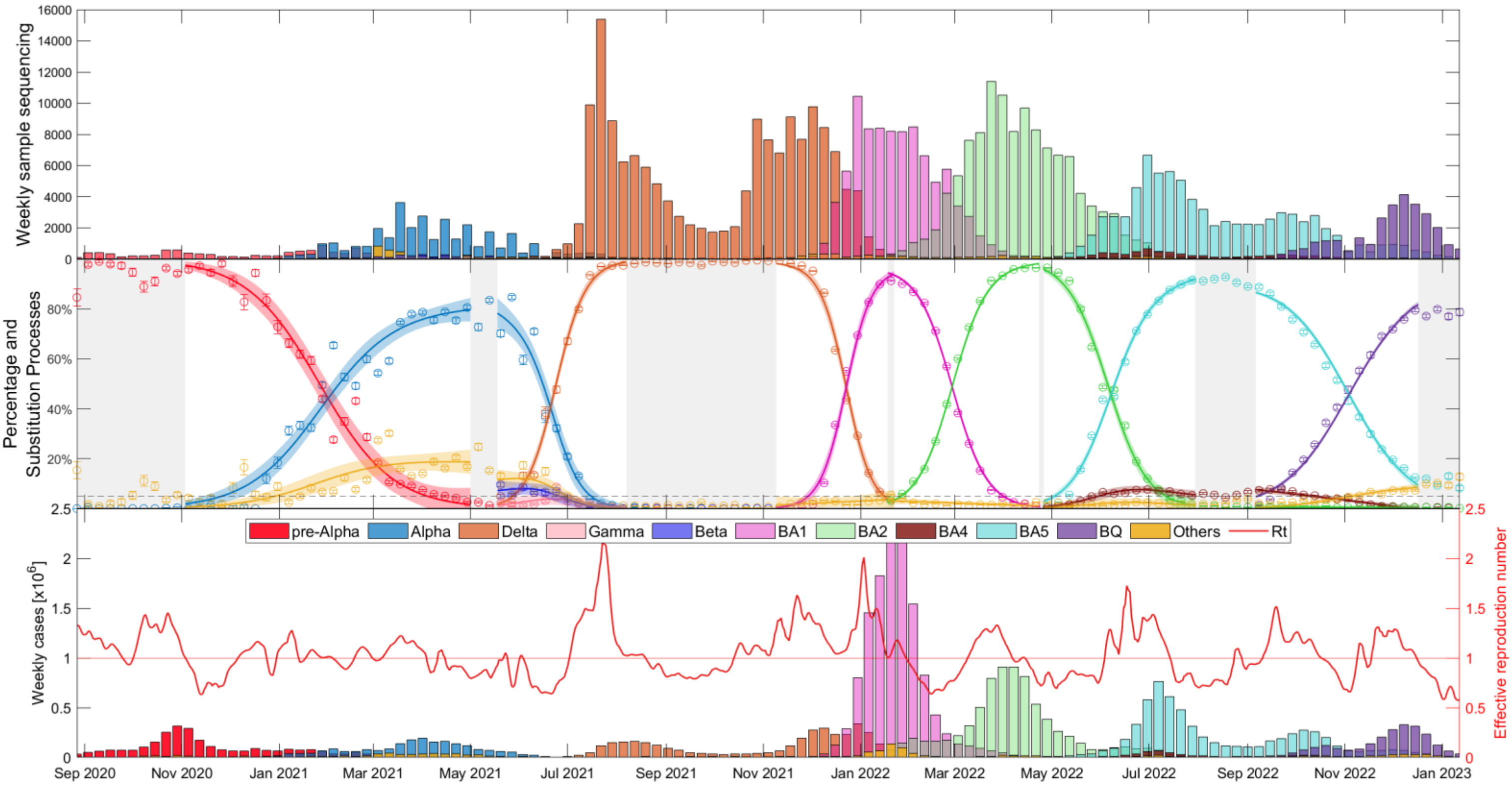

Figure S29 Evolutionary dynamics of Alpha, Delta, and Omicron lineages (BA.1, BA.2, BA.5, and BQ.1) substitutions in France over time.

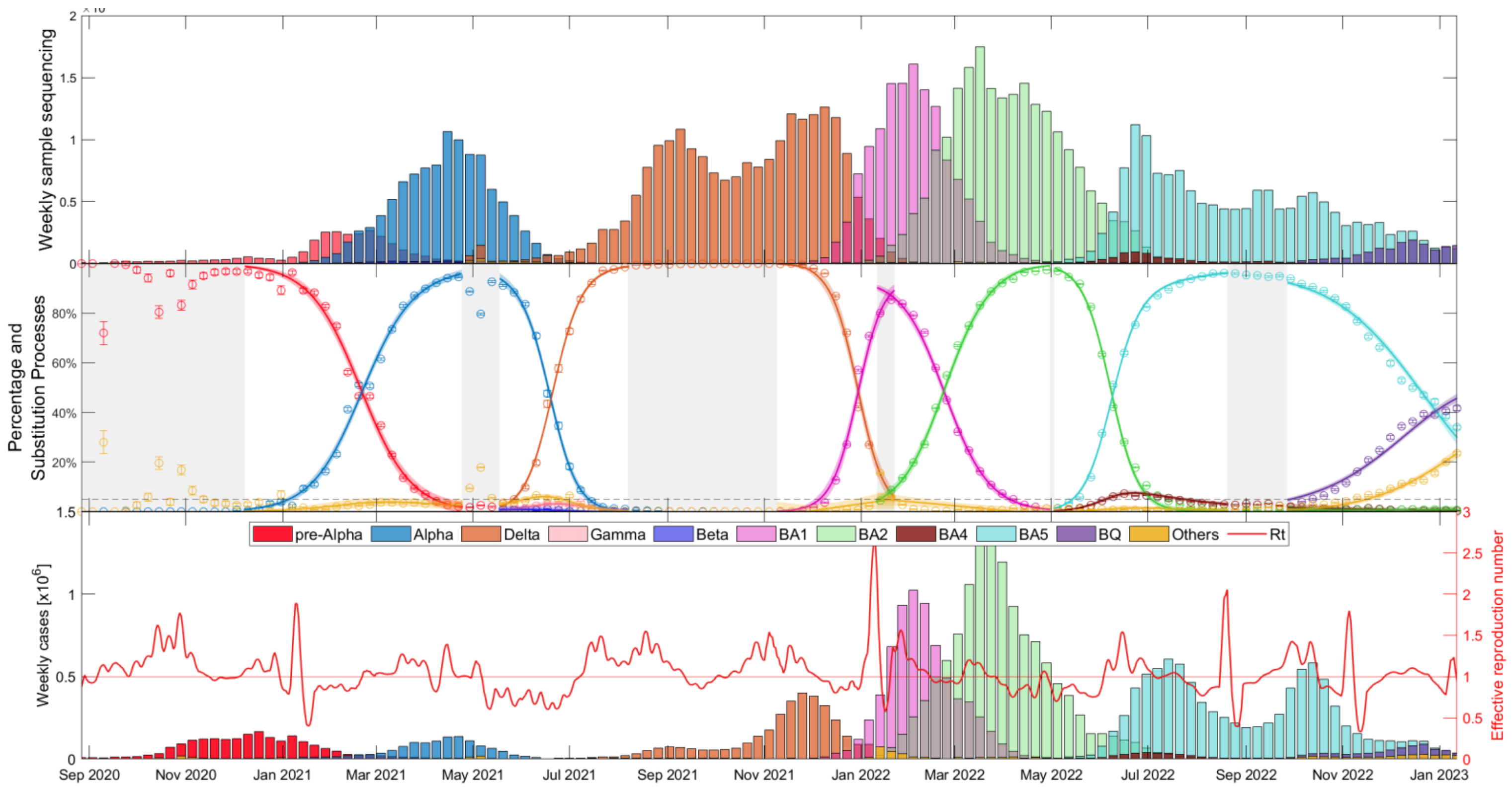

Figure S30 Evolutionary dynamics of Alpha, Delta, and Omicron lineages (BA.1, BA.2, BA.5, and BQ.1) substitutions in Germany over time.

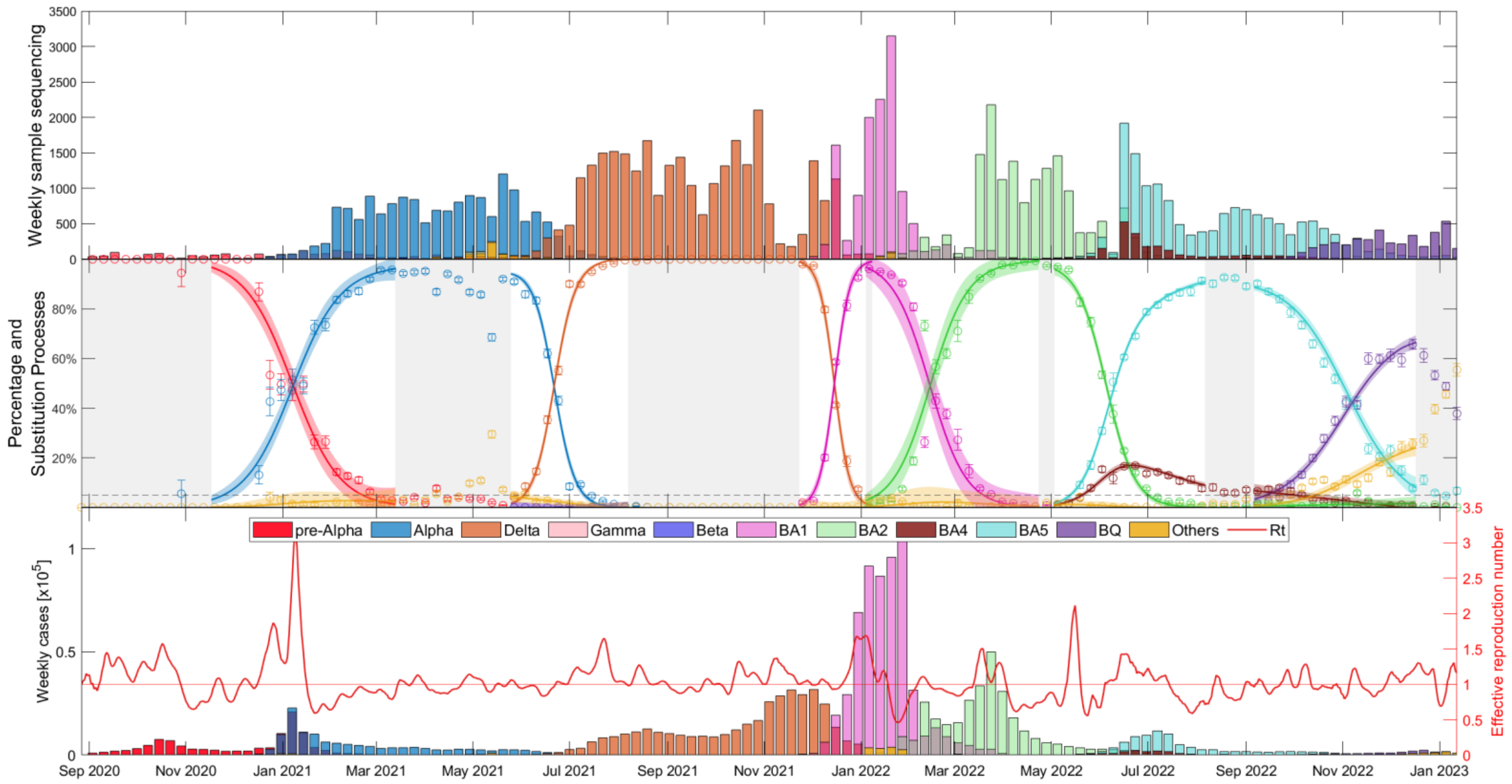

Figure S31 Evolutionary dynamics of Alpha, Delta, and Omicron lineages (BA.1, BA.2, BA.5, and BQ.1) substitutions in Ireland over time.

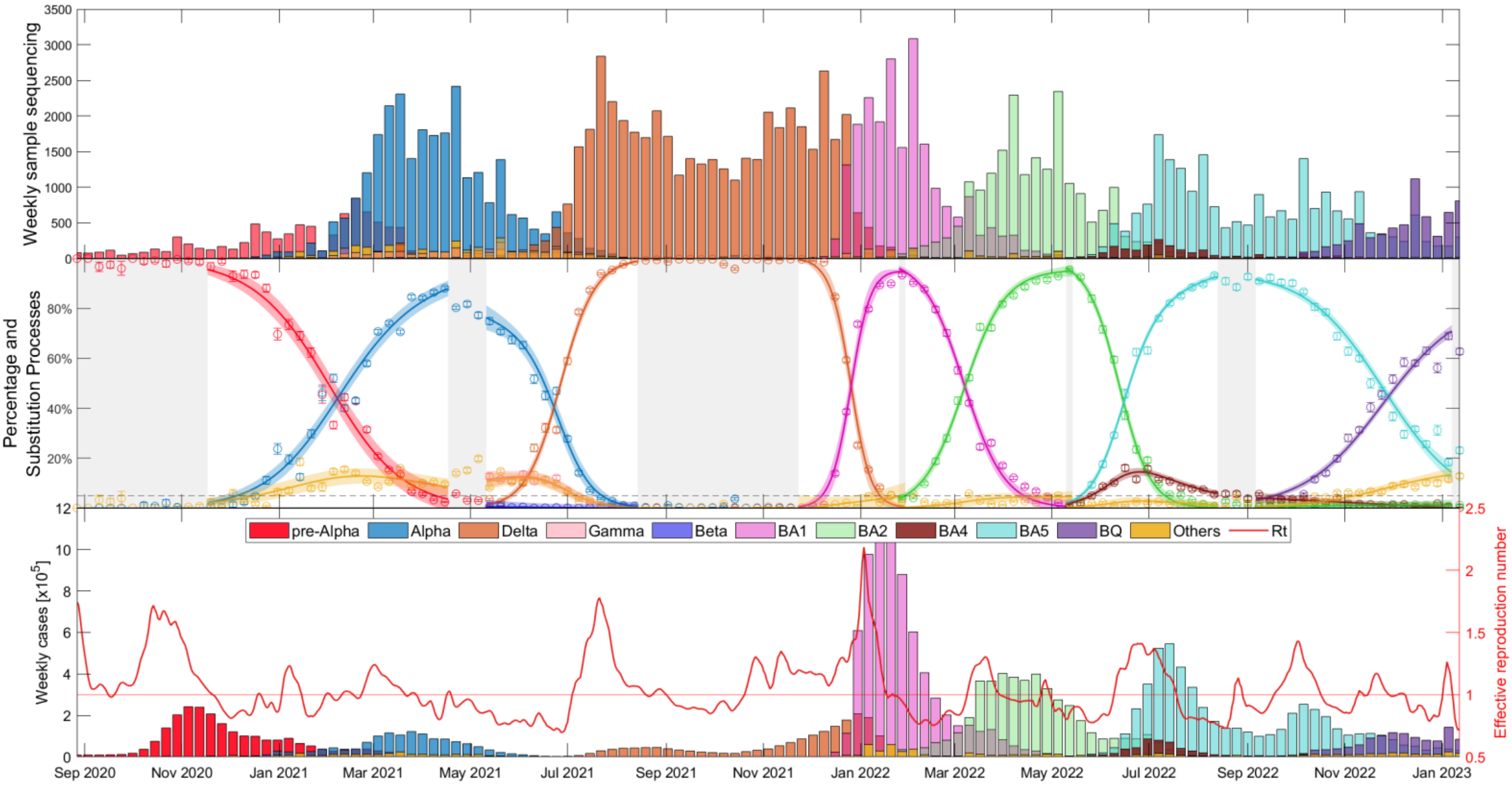

Figure S32 Evolutionary dynamics of Alpha, Delta, and Omicron lineages (BA.1, BA.2, BA.5, and BQ.1) substitutions in Italy over time.

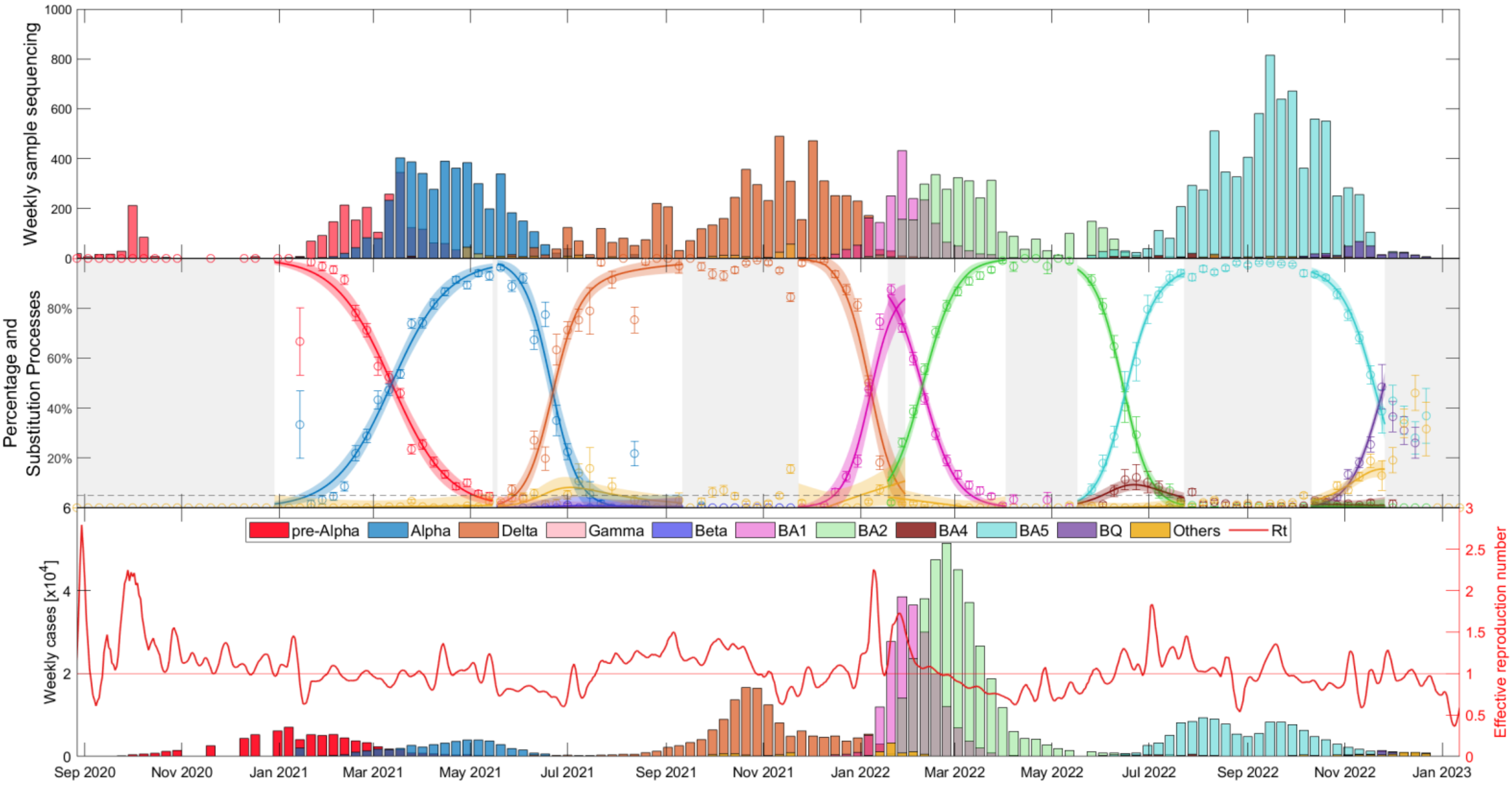

Figure S33 Evolutionary dynamics of Alpha, Delta, and Omicron lineages (BA.1, BA.2, BA.5, and BQ.1) substitutions in Latvia over time.

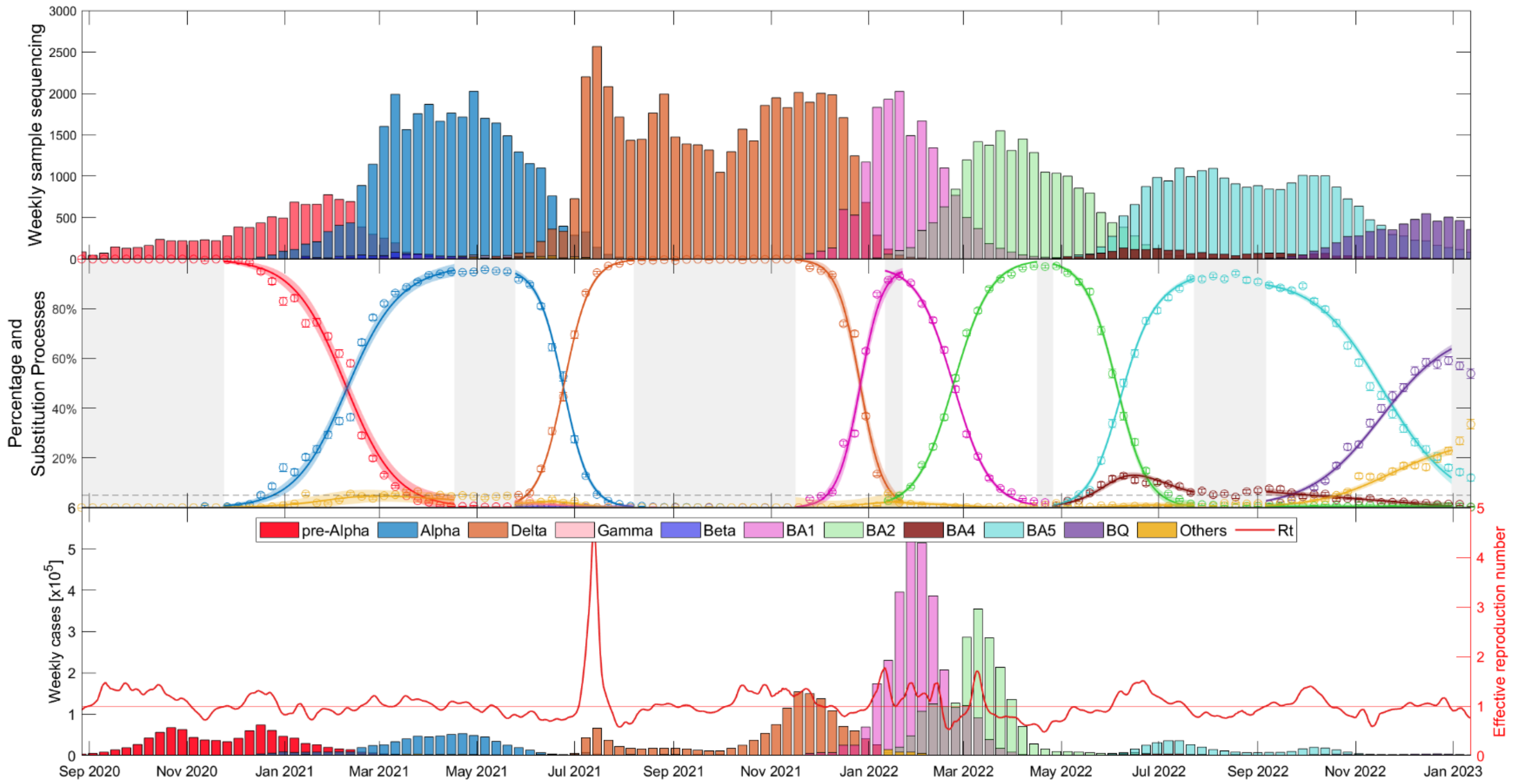

Figure S34 Evolutionary dynamics of Alpha, Delta, and Omicron lineages (BA.1, BA.2, BA.5, and BQ.1) substitutions in Netherlands over time.

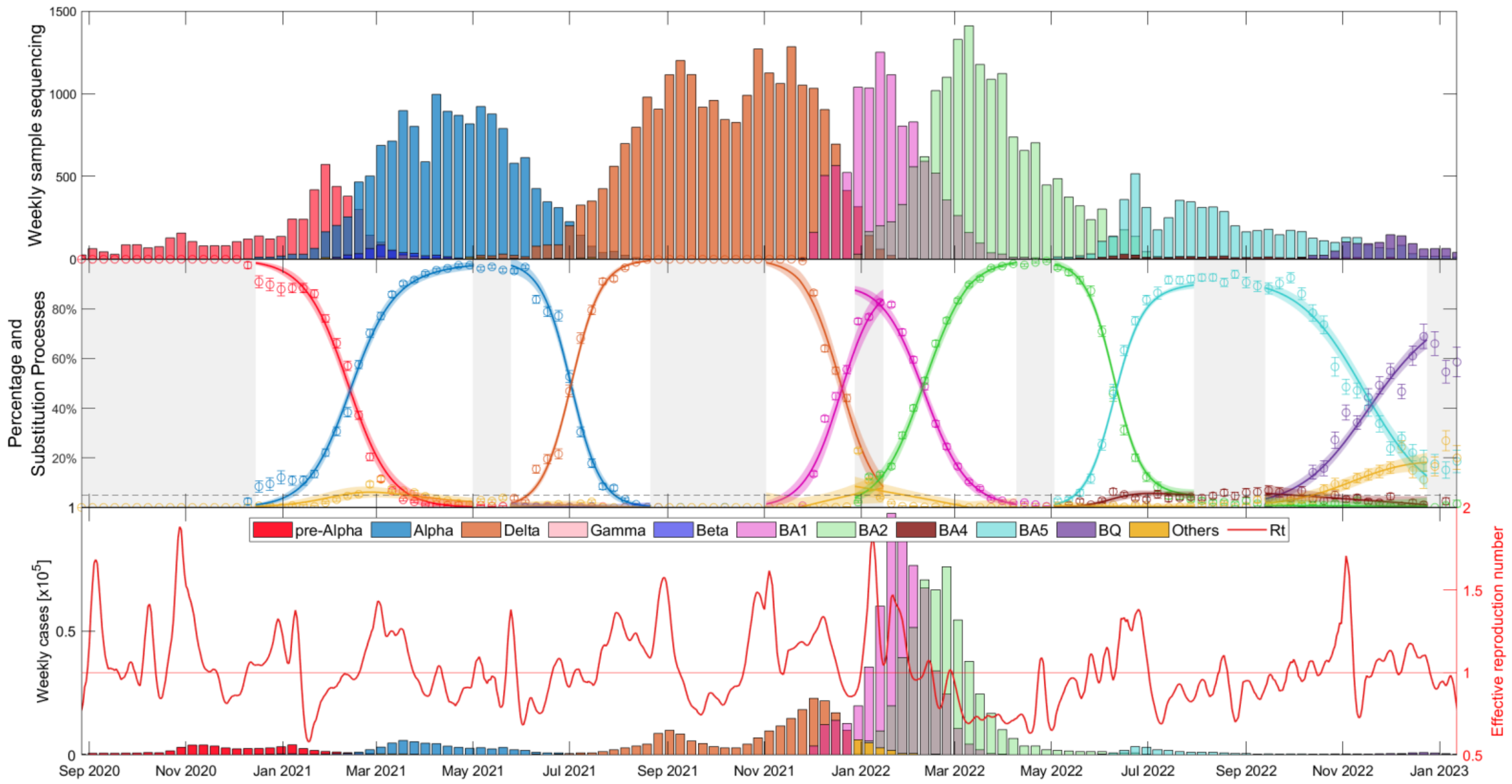

Figure S35 Evolutionary dynamics of Alpha, Delta, and Omicron lineages (BA.1, BA.2, BA.5, and BQ.1) substitutions in Norway over time.

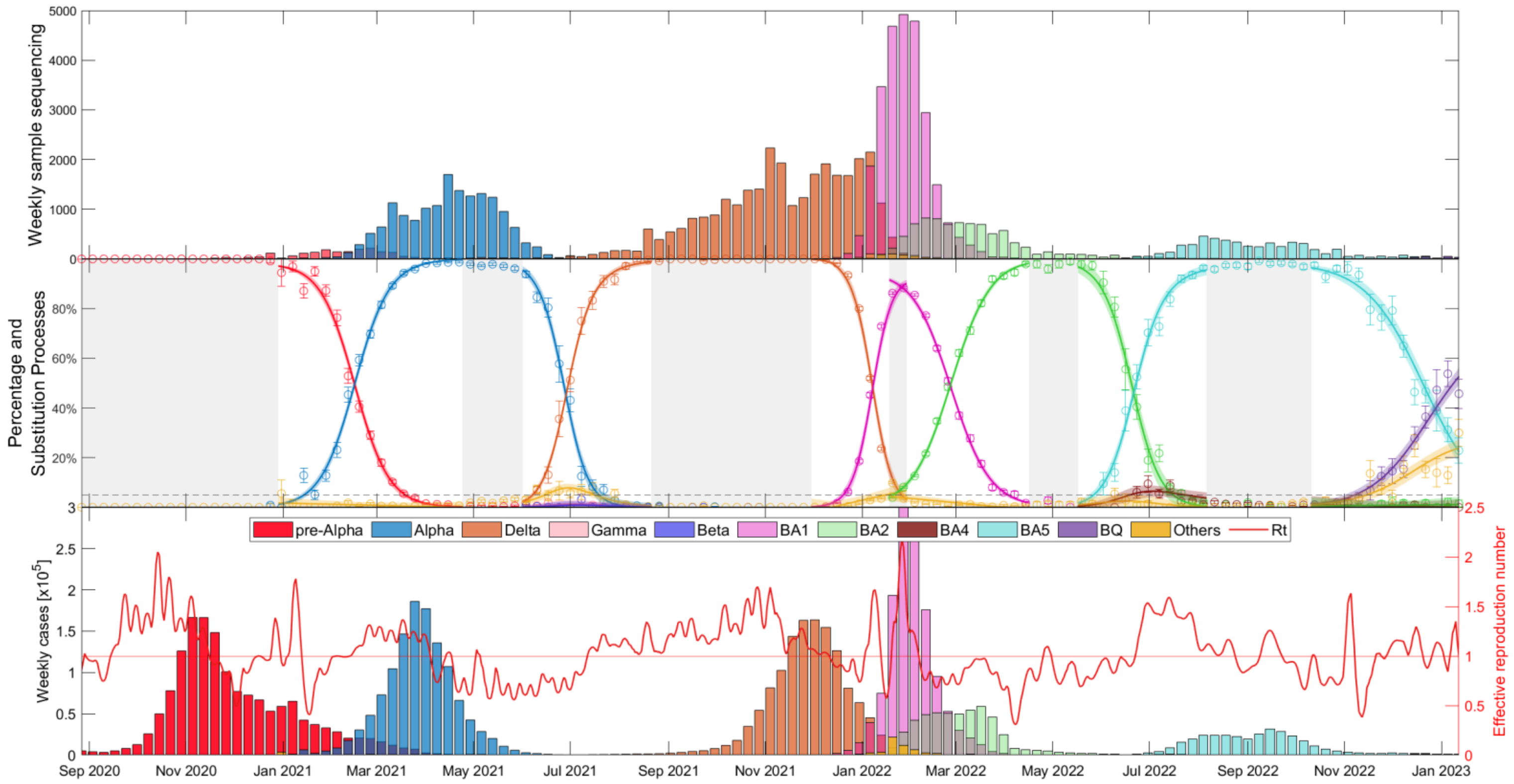

Figure S36 Evolutionary dynamics of Alpha, Delta, and Omicron lineages (BA.1, BA.2, BA.5, and BQ.1) substitutions in Poland over time.

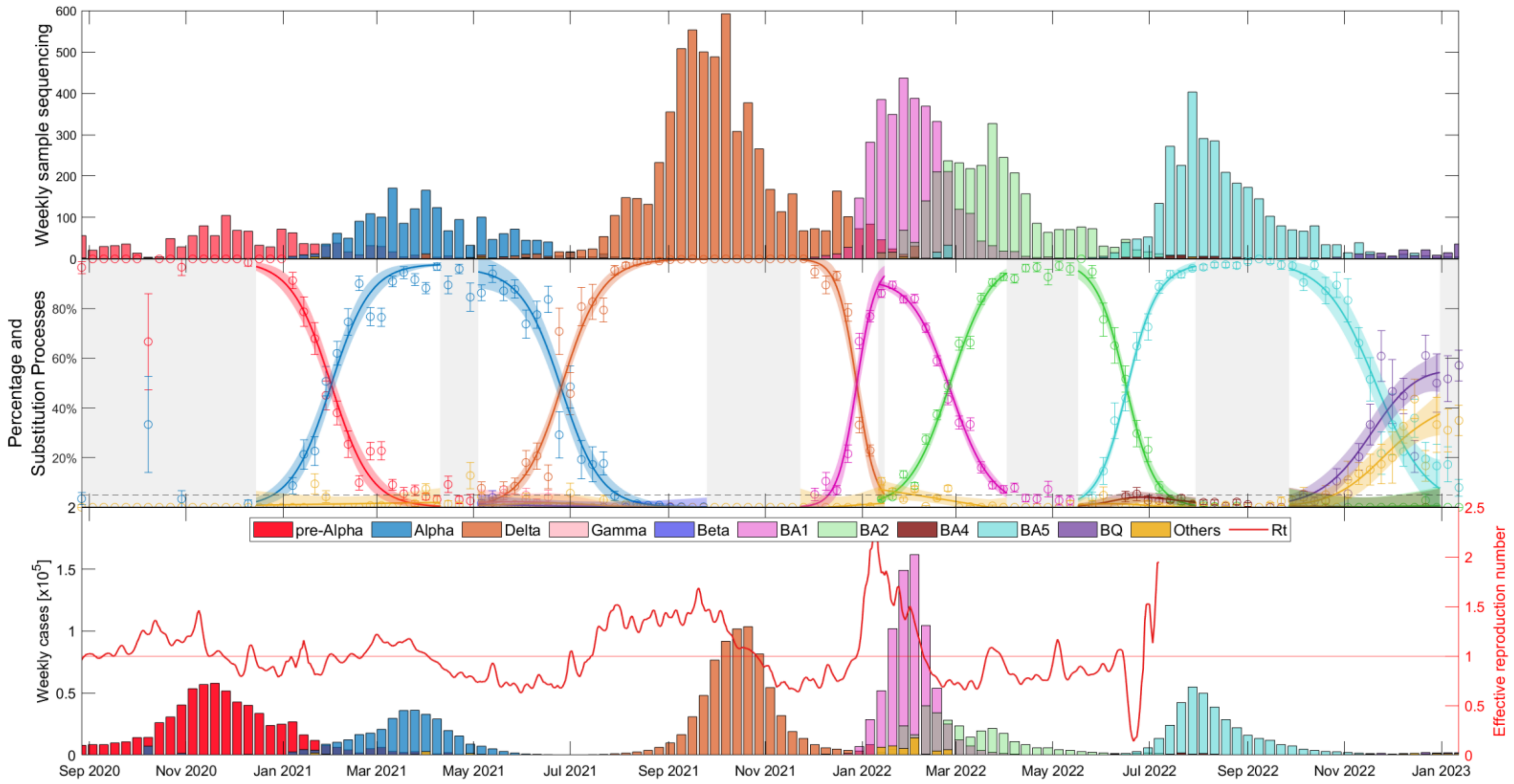

Figure S37 Evolutionary dynamics of Alpha, Delta, and Omicron lineages (BA.1, BA.2, BA.5, and BQ.1) substitutions in Romania over time.

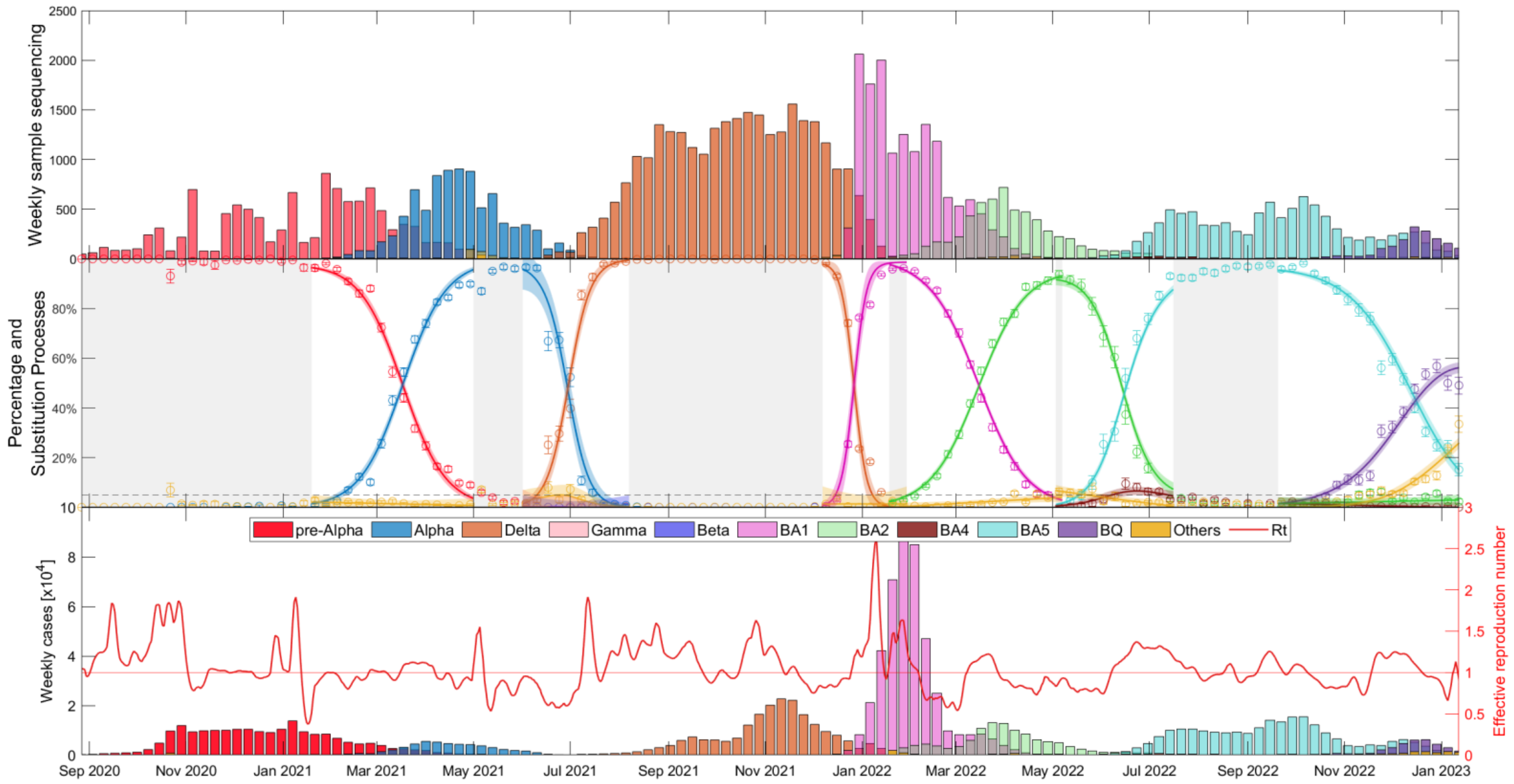

Figure S38 Evolutionary dynamics of Alpha, Delta, and Omicron lineages (BA.1, BA.2, BA.5, and BQ.1) substitutions in Slovenia over time.

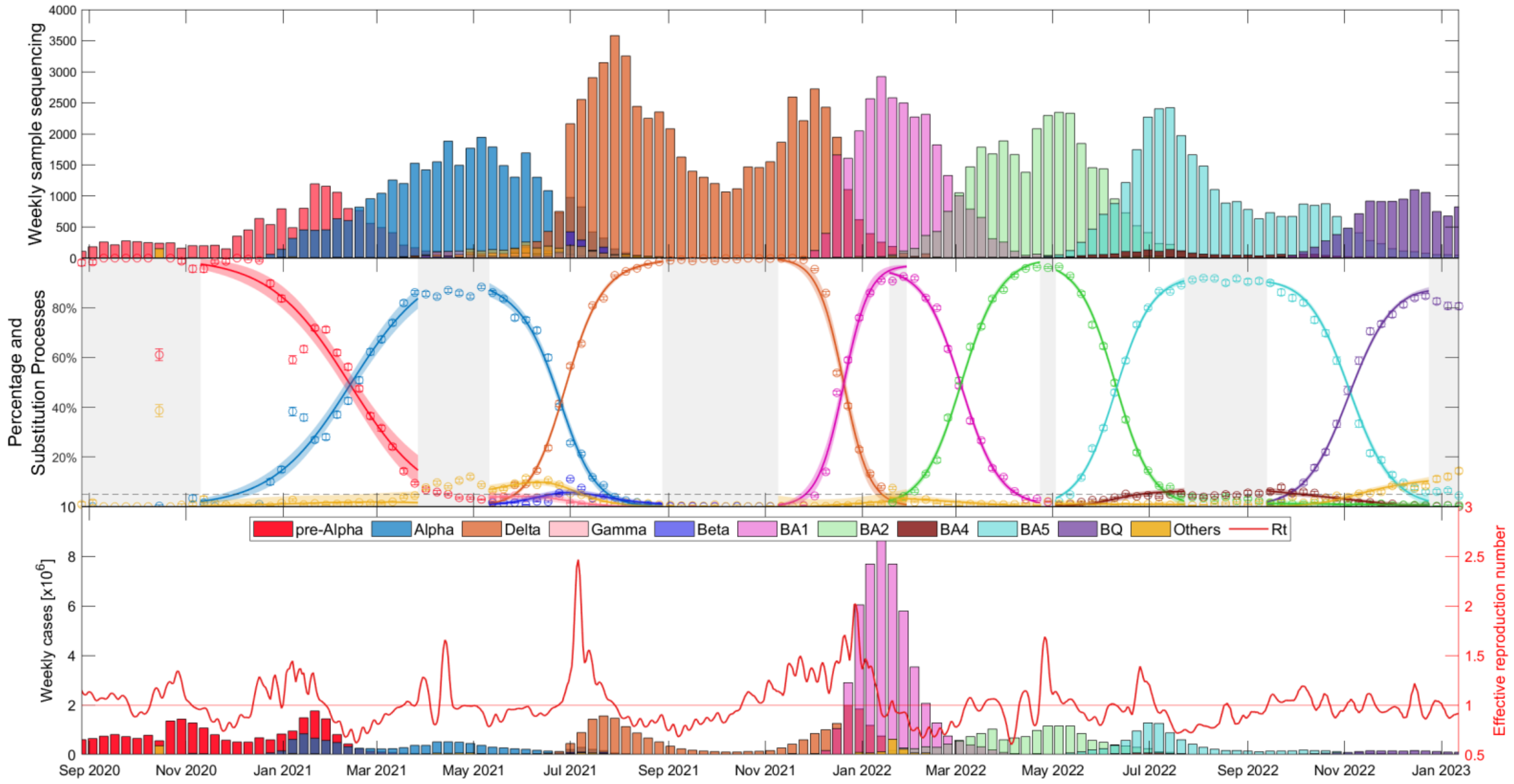

Figure S39 Evolutionary dynamics of Alpha, Delta, and Omicron lineages (BA.1, BA.2, BA.5, and BQ.1) substitutions in Spain over time.

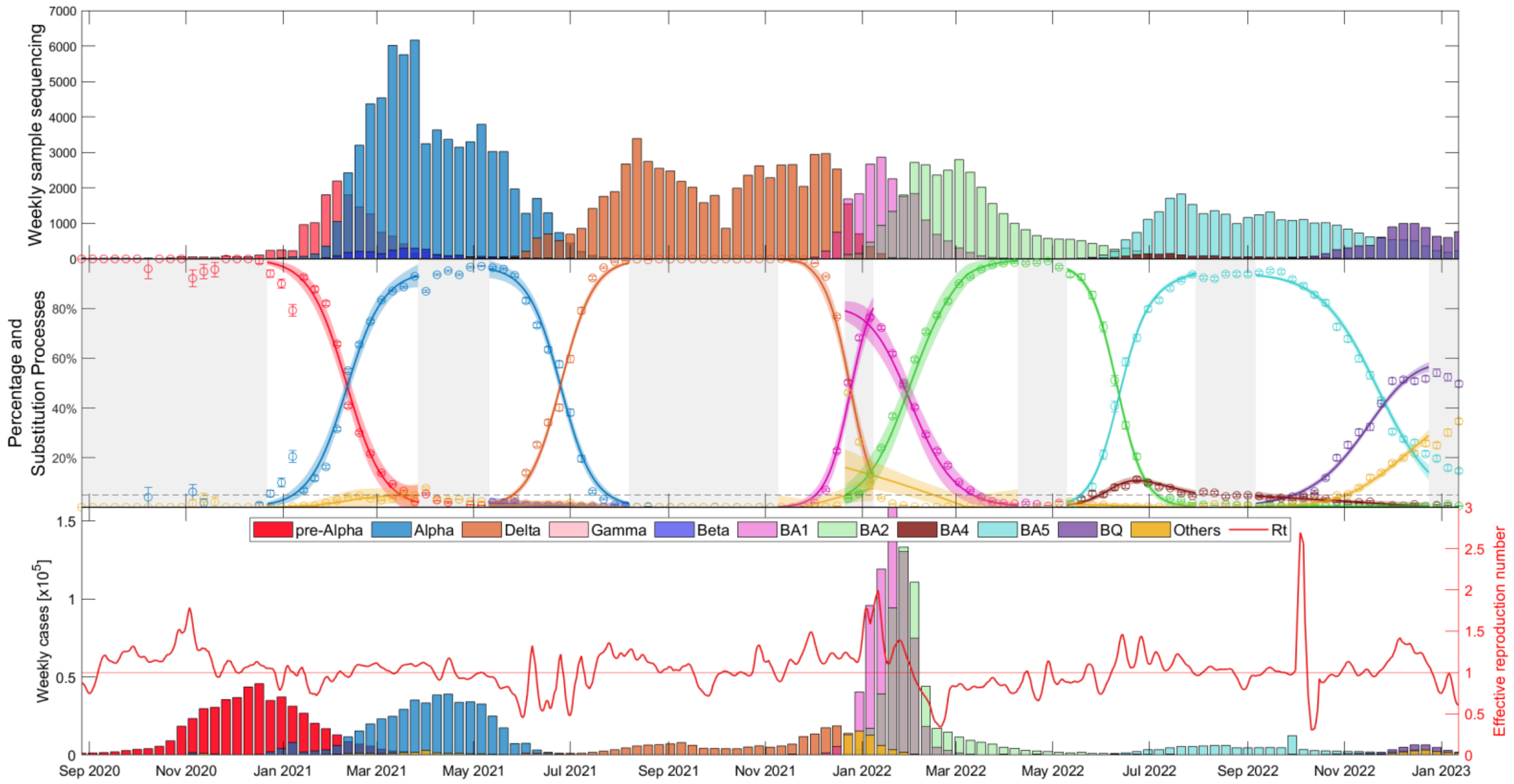

Figure S40 Evolutionary dynamics of Alpha, Delta, and Omicron lineages (BA.1, BA.2, BA.5, and BQ.1) substitutions in Sweden over time.

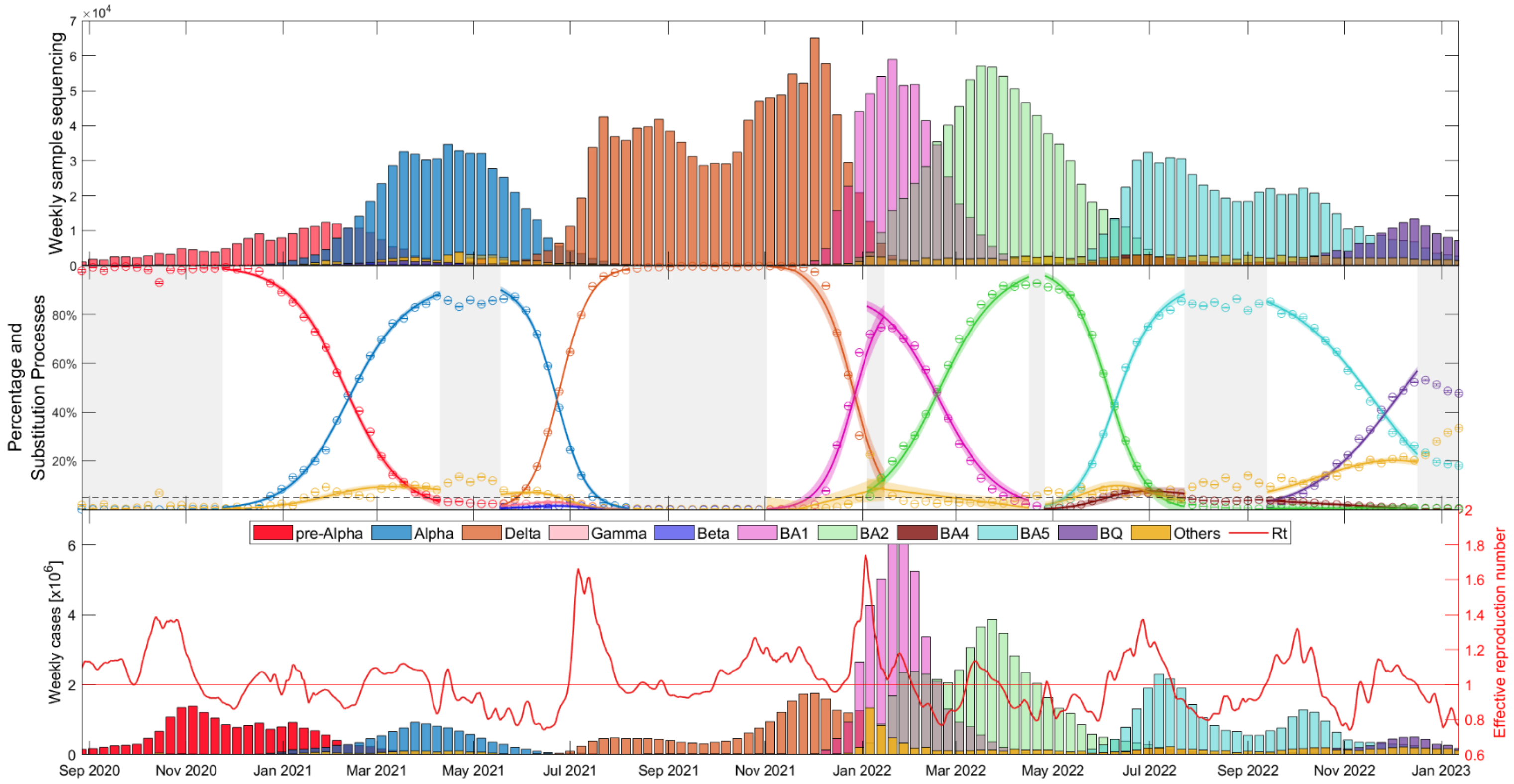

Figure S41 Evolutionary dynamics of Alpha, Delta, and Omicron lineages (BA.1, BA.2, BA.5, and BQ.1) substitutions in Europe over time.

Suppl. Material Text S7 – Two-variant substitutions dynamics with Omicron lineages for each country with GISAID data source

In Figures 1 and 2 in the main article and Figures S2-S40 in the Suppl. Material Text S5 and Suppl. Material Text S6 we presented the main substitution for all European countries included in this study. From this research, we derived the increase in transmissibility of one variant relative to all the simultaneous variants present in each country. However, to gain deeper insights into the variants that dominated specific periods in Europe—primarily the variants classified as VOC by the WHO—and to enable comparison of their transmissibility between them and across different regions, it might be more insightful to examine each substitution considering only the two variants: the one that dominated and the one that subsequently took over. These two variants are the same for each country and in each substitution. This means we compare pre-Alpha vs Alpha, Alpha vs Delta, Delta vs BA.1, BA.1 vs BA.2, BA.2 vs BA.5, and BA.5 vs BQ.1.

Adhering to the methodology employed in the main article, the subsequent figures in this Supplementary Material depict the substitution dynamics between the two dominant variants for all the countries encompassed in our study. This method focuses on a variant that was dominant and only the subsequent one that takes precedence, facilitating a practical comparison of their unique transmissibility across diverse geographical contexts. In this case, we cannot compute the daily percentage of each variant for the individual countries (given that we are excluding other circulating variants). However, the resulting $\Delta\beta$ in this context will give us an accurate estimation of the superiority of one variant compared to its predecessor.

It is important to note that that for Bulgaria, data pertaining to the BQ.1 substitution is absent (see Figure S43). Likewise, for Lithuania, data on the Omicron variant is missing due to the unavailability of reported information relevant to the substitutions of the BA.2, BA.5, and BQ.1 lineages (see Figure S53).

Consequently, these illustrations provide a comprehensive portrayal of the transmissibility transition from one variant to another across all countries investigated in our research. A more detailed analysis of these results is available in Suppl. Material Text S12 . These findings were not included in the main article because their conclusions align broadly with the primary results.

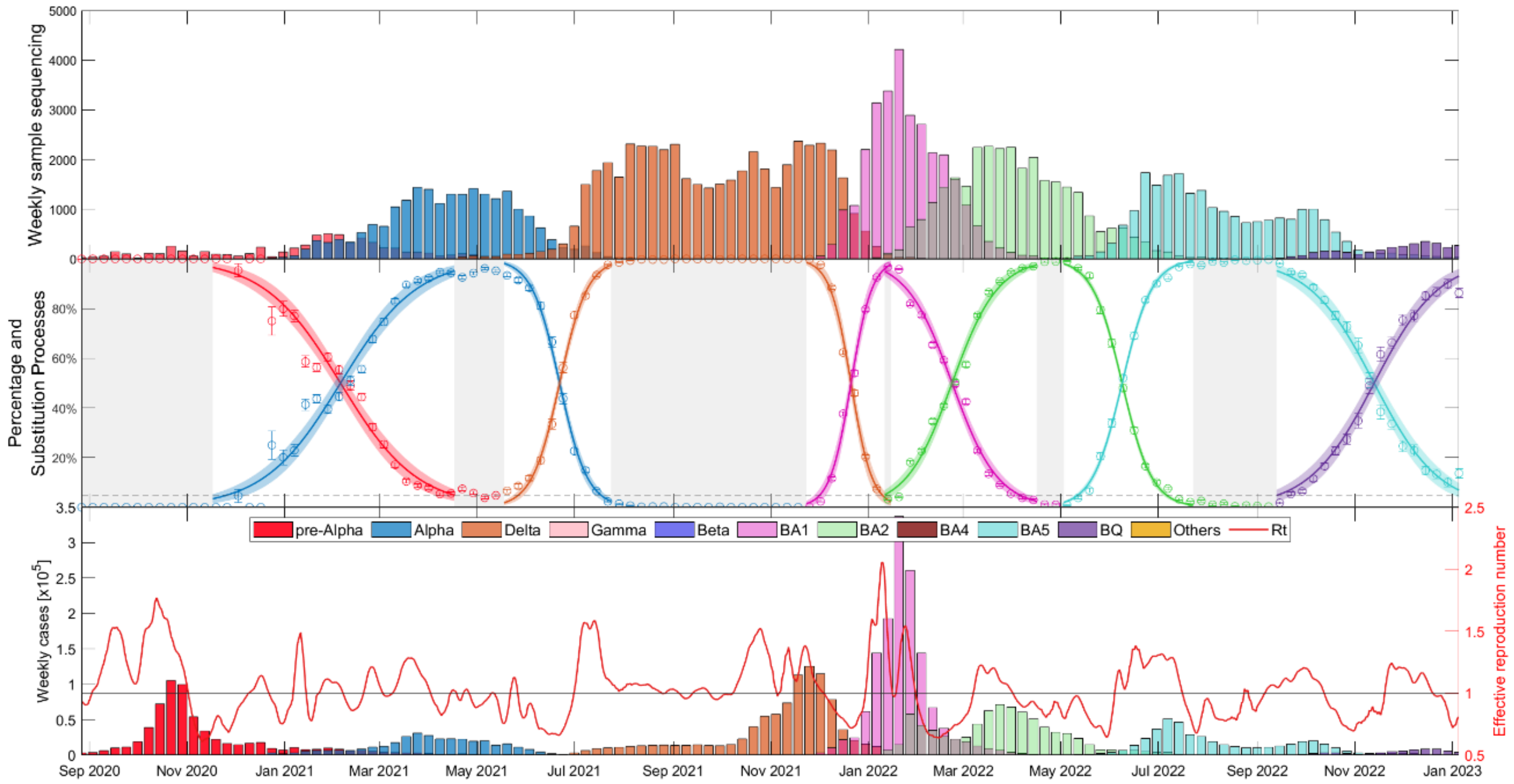

Figure S42 Temporal changes in the dominance of Alpha, Delta, and Omicron lineages (BA.1, BA.2, BA.5, and BQ.1) COVID-19 variants for two-variant substitution processes in Belgium over time.

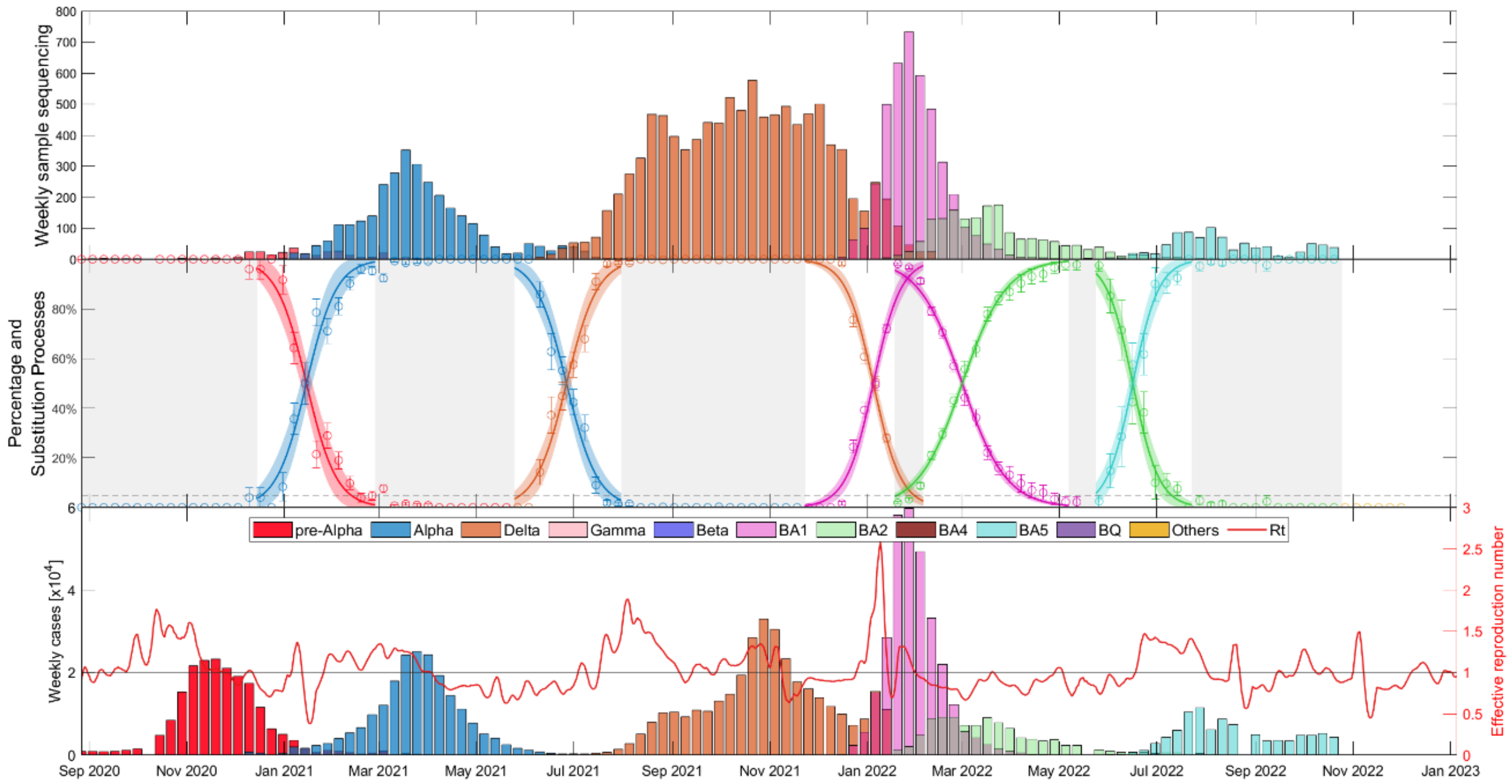

Figure S43 Temporal changes in the dominance of Alpha, Delta, and Omicron lineages (BA.1, BA.2, BA.5, and BQ.1) COVID-19 variants for two-variant substitution processes in Bulgaria over time.

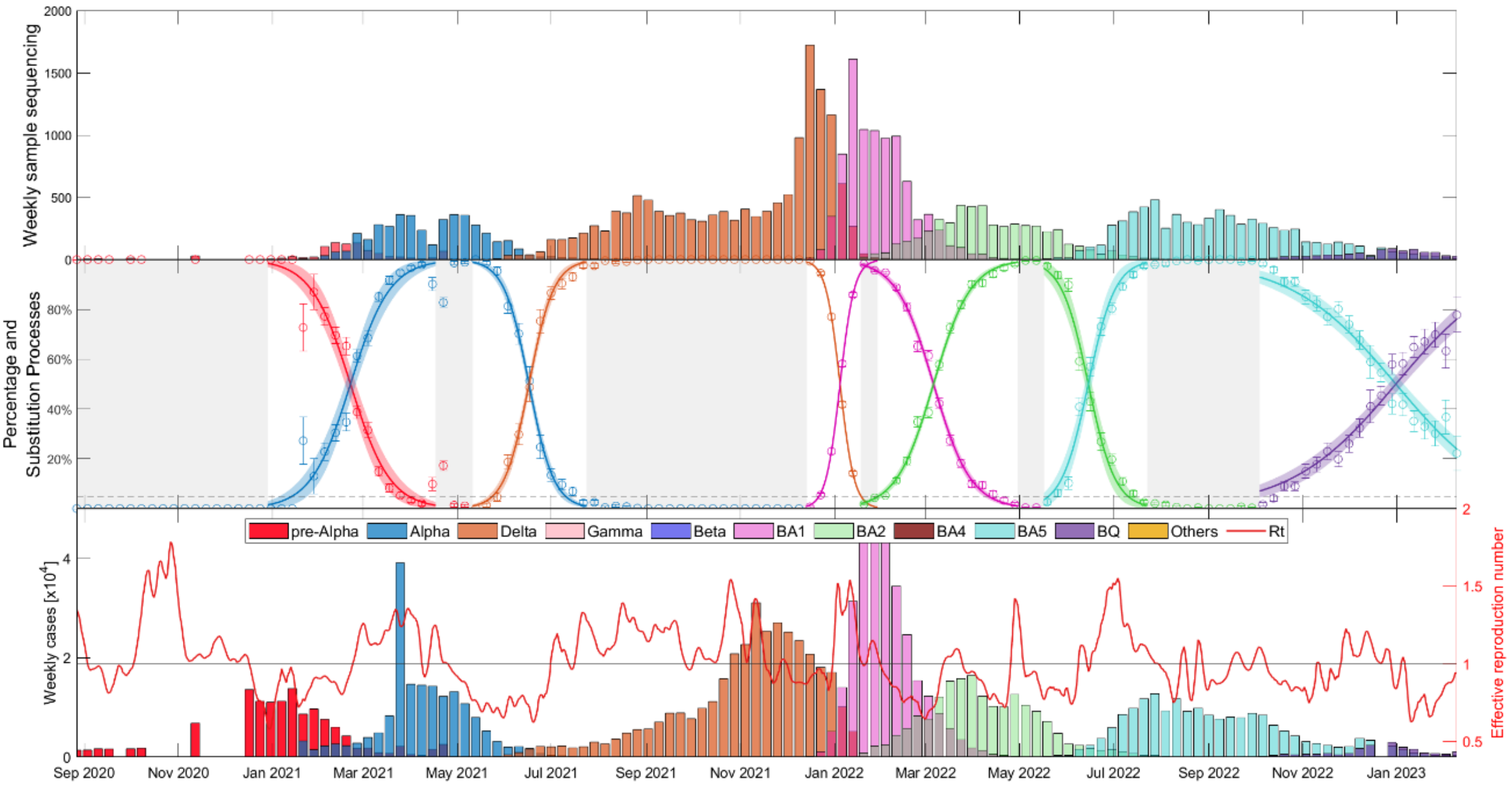

Figure S44 Temporal changes in the dominance of Alpha, Delta, and Omicron lineages (BA.1, BA.2, BA.5, and BQ.1) COVID-19 variants for two-variant substitution processes in Croatia over time.

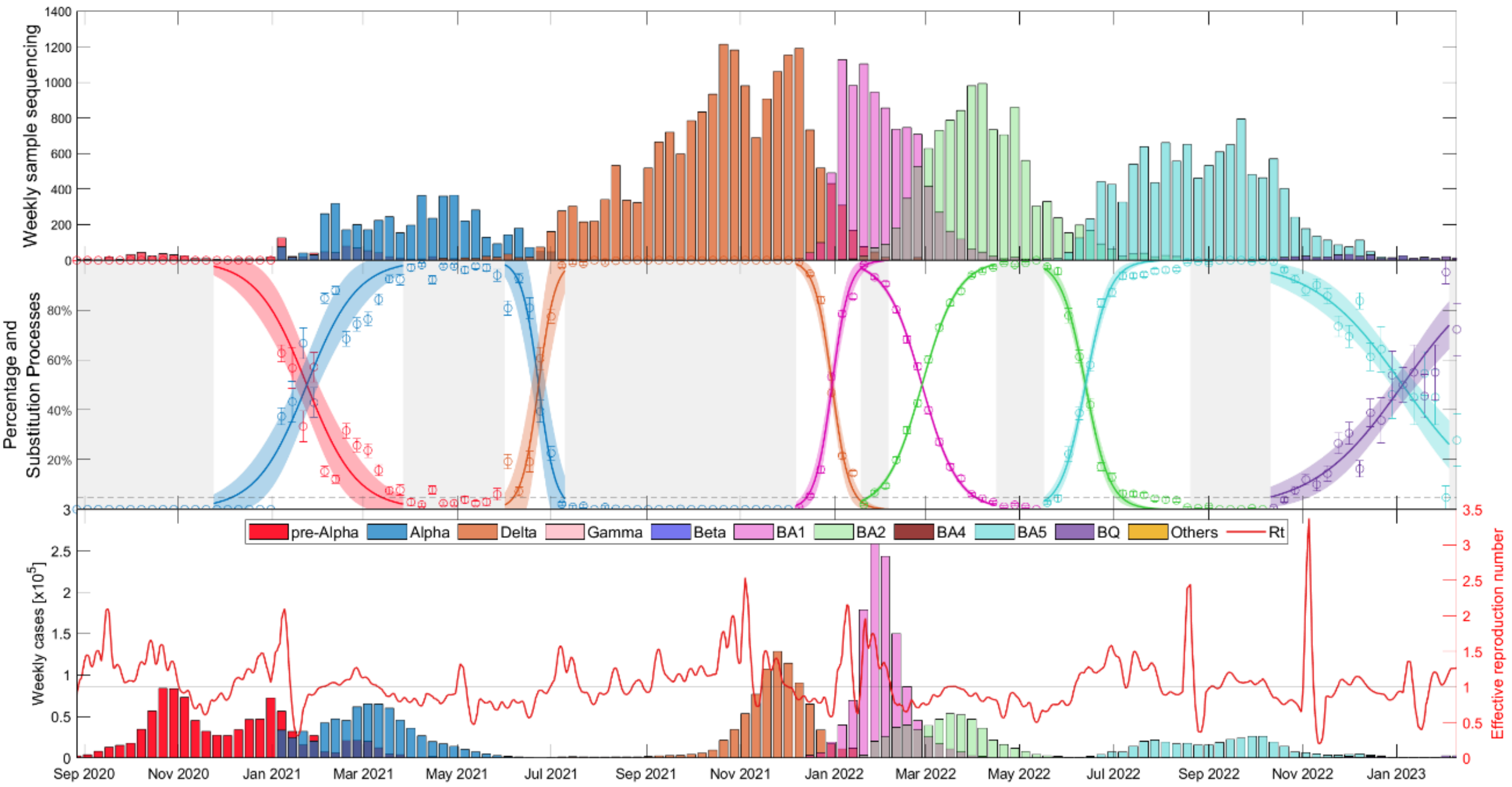

Figure S45 Temporal changes in the dominance of Alpha, Delta, and Omicron lineages (BA.1, BA.2, BA.5, and BQ.1) COVID-19 variants for two-variant substitution processes in Czechia over time.

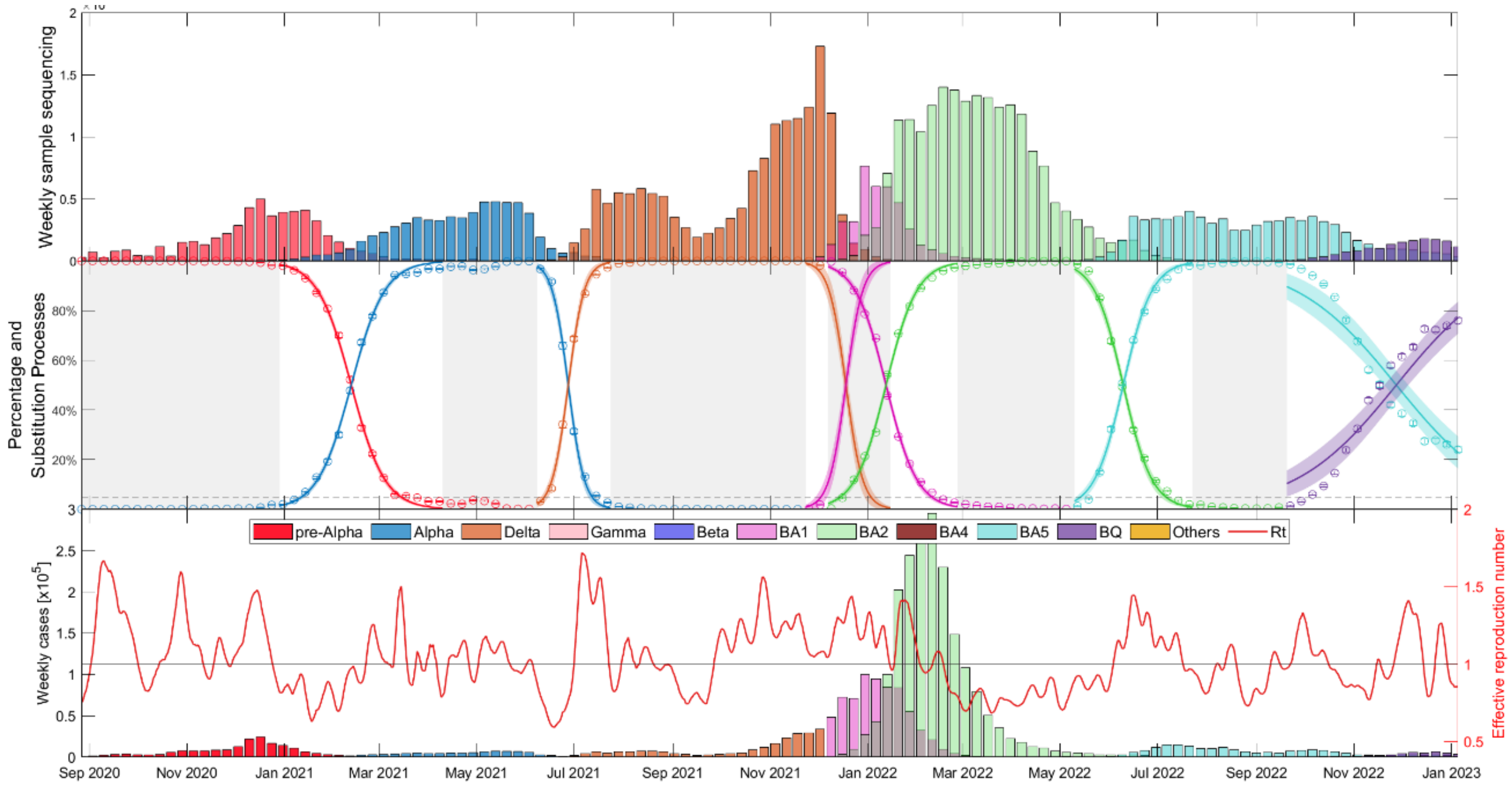

Figure S46 Temporal changes in the dominance of Alpha, Delta, and Omicron lineages (BA.1, BA.2, BA.5, and BQ.1) COVID-19 variants for two-variant substitution processes in Denmark over time.

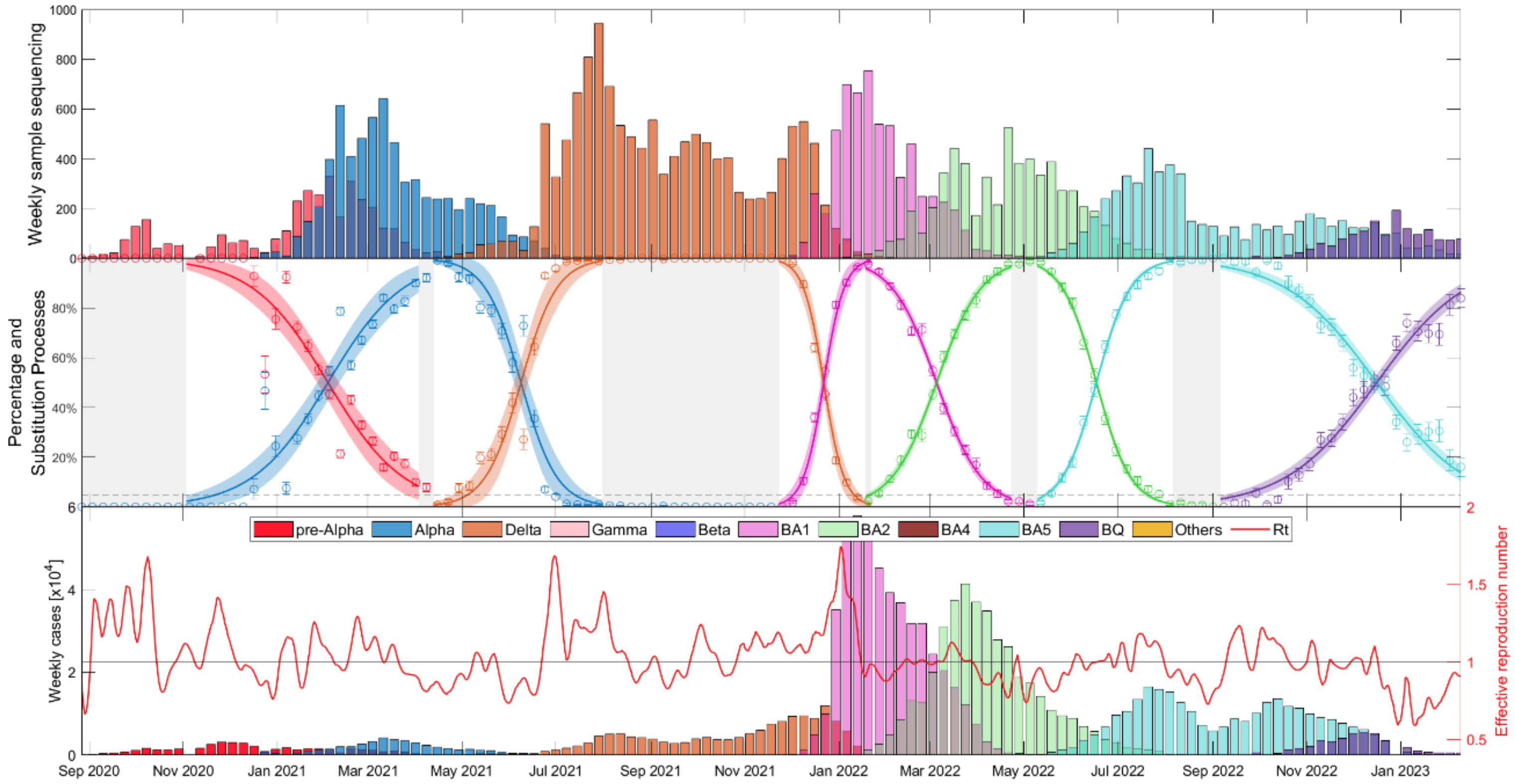

Figure S47 Temporal changes in the dominance of Alpha, Delta, and Omicron lineages (BA.1, BA.2, BA.5, and BQ.1) COVID-19 variants for two-variant substitution processes in Finland over time.

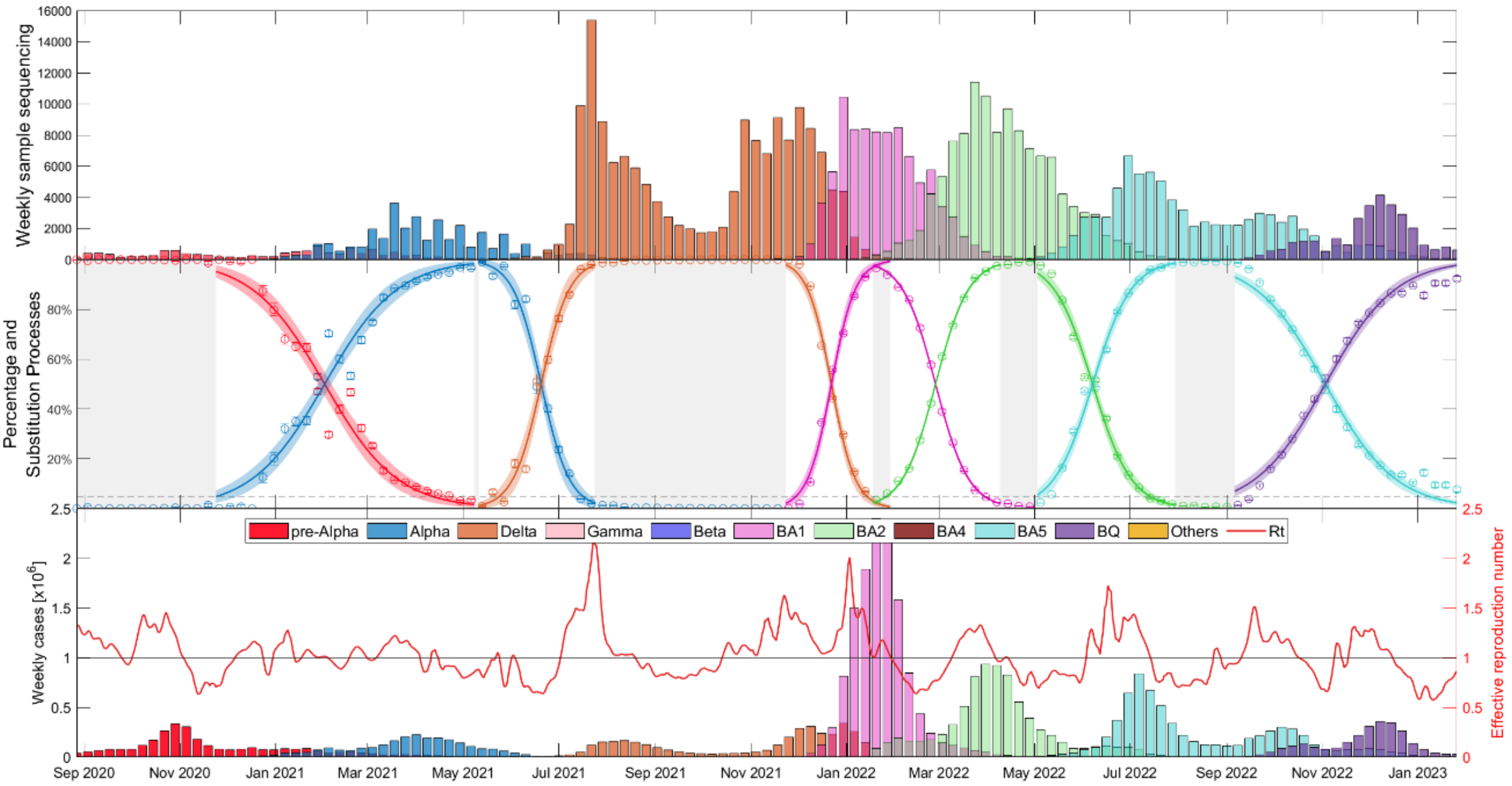

Figure S48 Temporal changes in the dominance of Alpha, Delta, and Omicron lineages (BA.1, BA.2, BA.5, and BQ.1) COVID-19 variants for two-variant substitution processes in France over time.

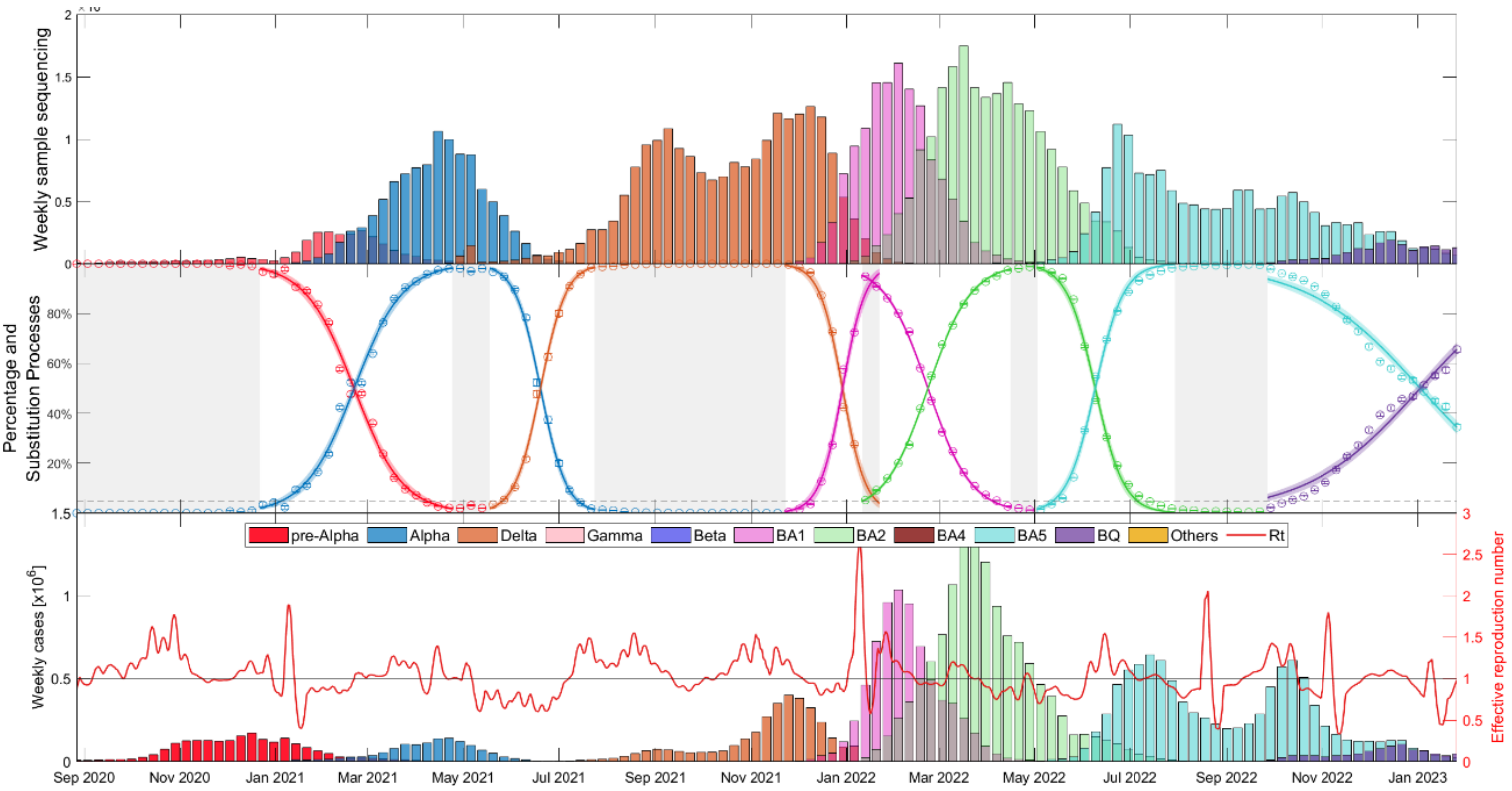

Figure S49 Temporal changes in the dominance of Alpha, Delta, and Omicron lineages (BA.1, BA.2, BA.5, and BQ.1) COVID-19 variants for two-variant substitution processes in Germany over time.

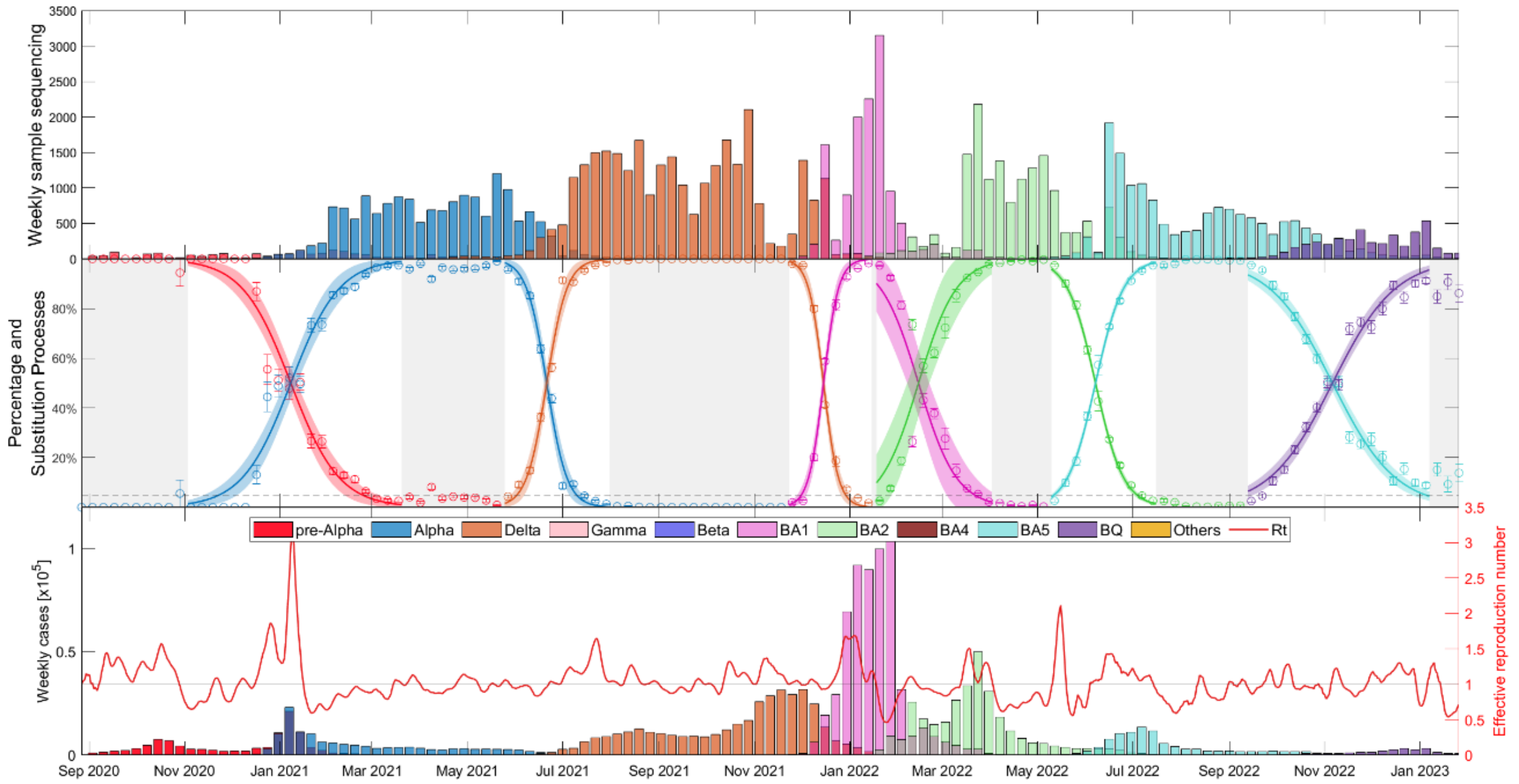

Figure S50 Temporal changes in the dominance of Alpha, Delta, and Omicron lineages (BA.1, BA.2, BA.5, and BQ.1) COVID-19 variants for two-variant substitution processes in Ireland over time.

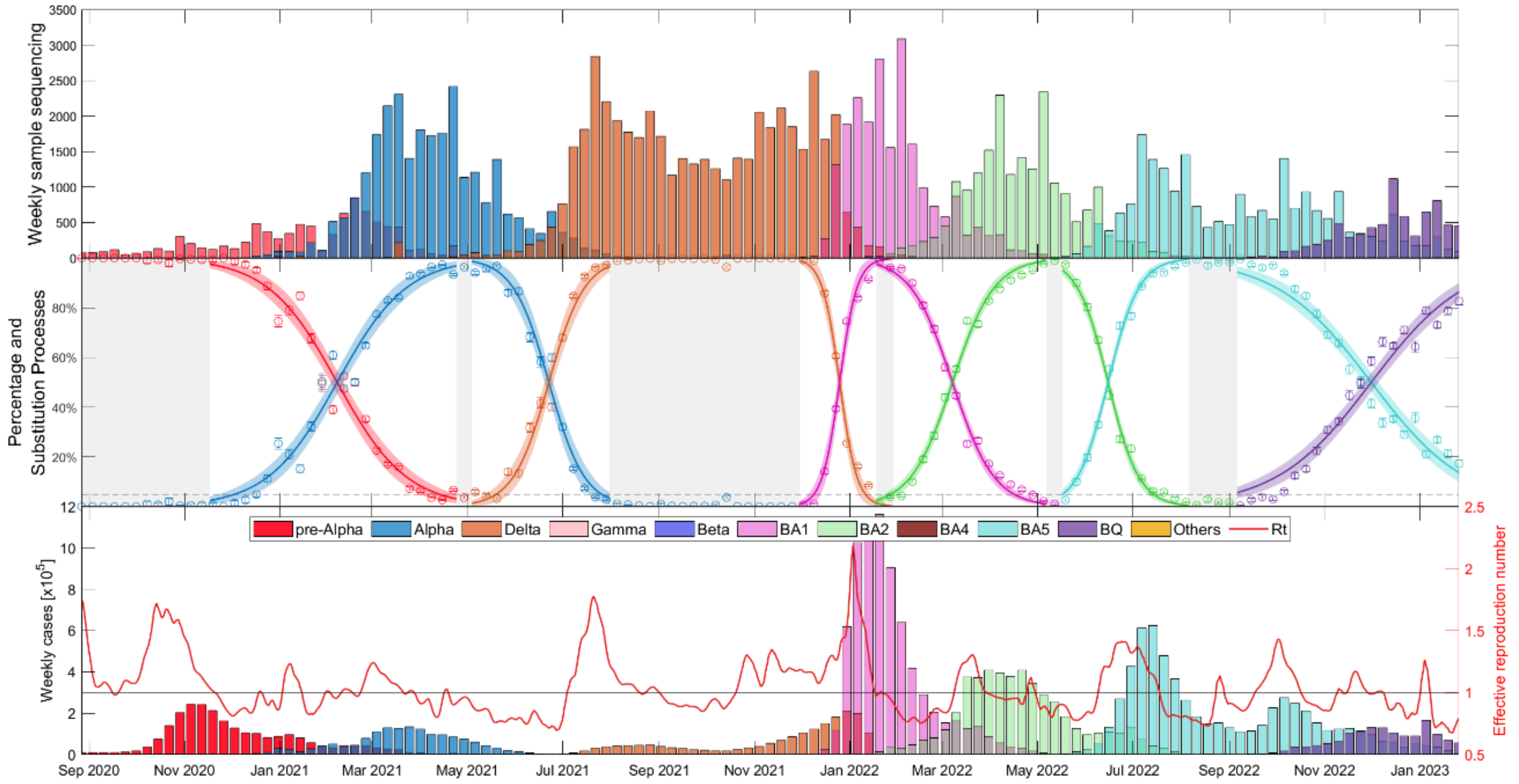

Figure S51 Temporal changes in the dominance of Alpha, Delta, and Omicron lineages (BA.1, BA.2, BA.5, and BQ.1) COVID-19 variants for two-variant substitution processes in Italy over time.

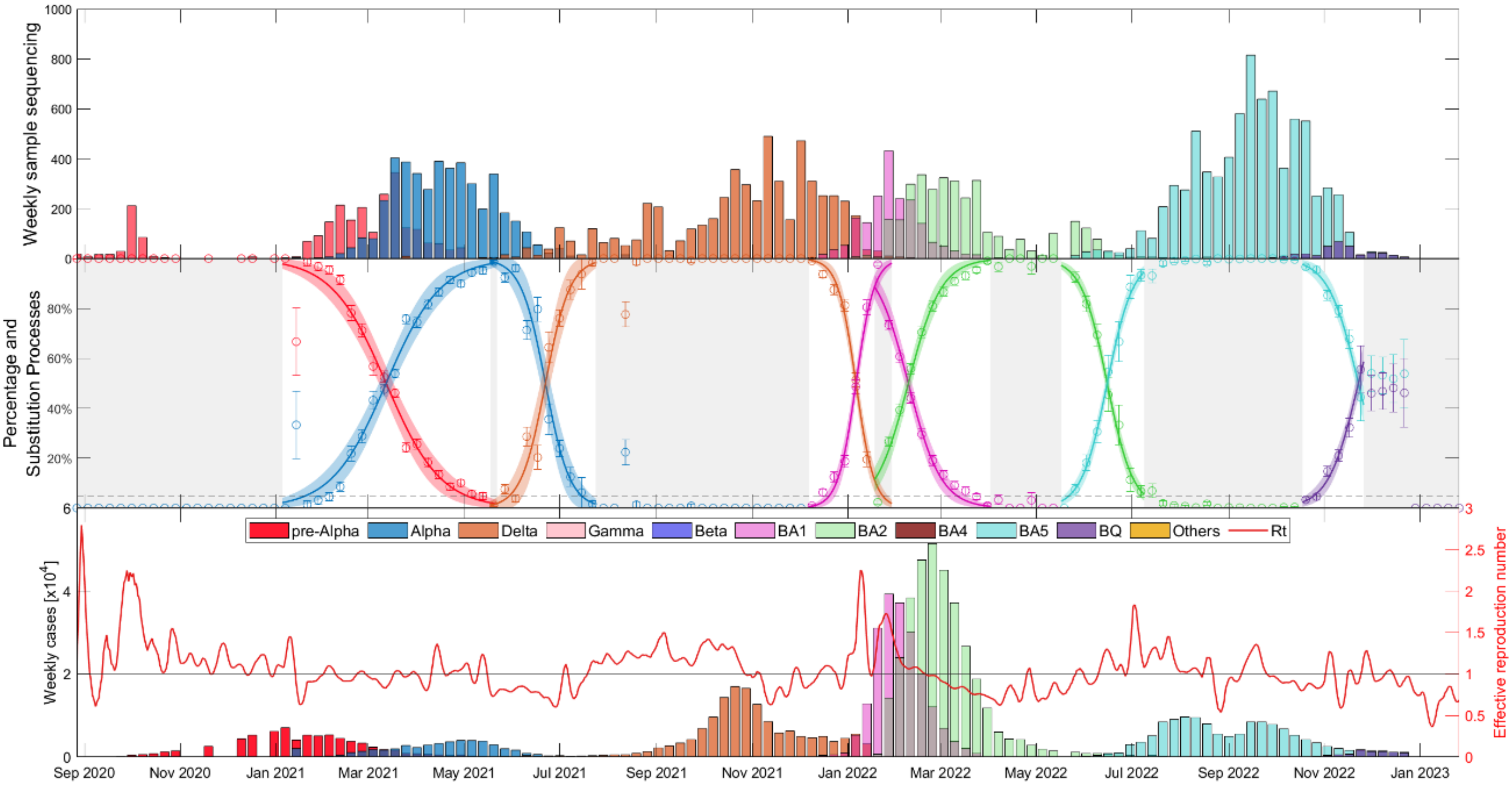

Figure S52 Temporal changes in the dominance of Alpha, Delta, and Omicron lineages (BA.1, BA.2, BA.5, and BQ.1) COVID-19 variants for two-variant substitution processes in Latvia over time.

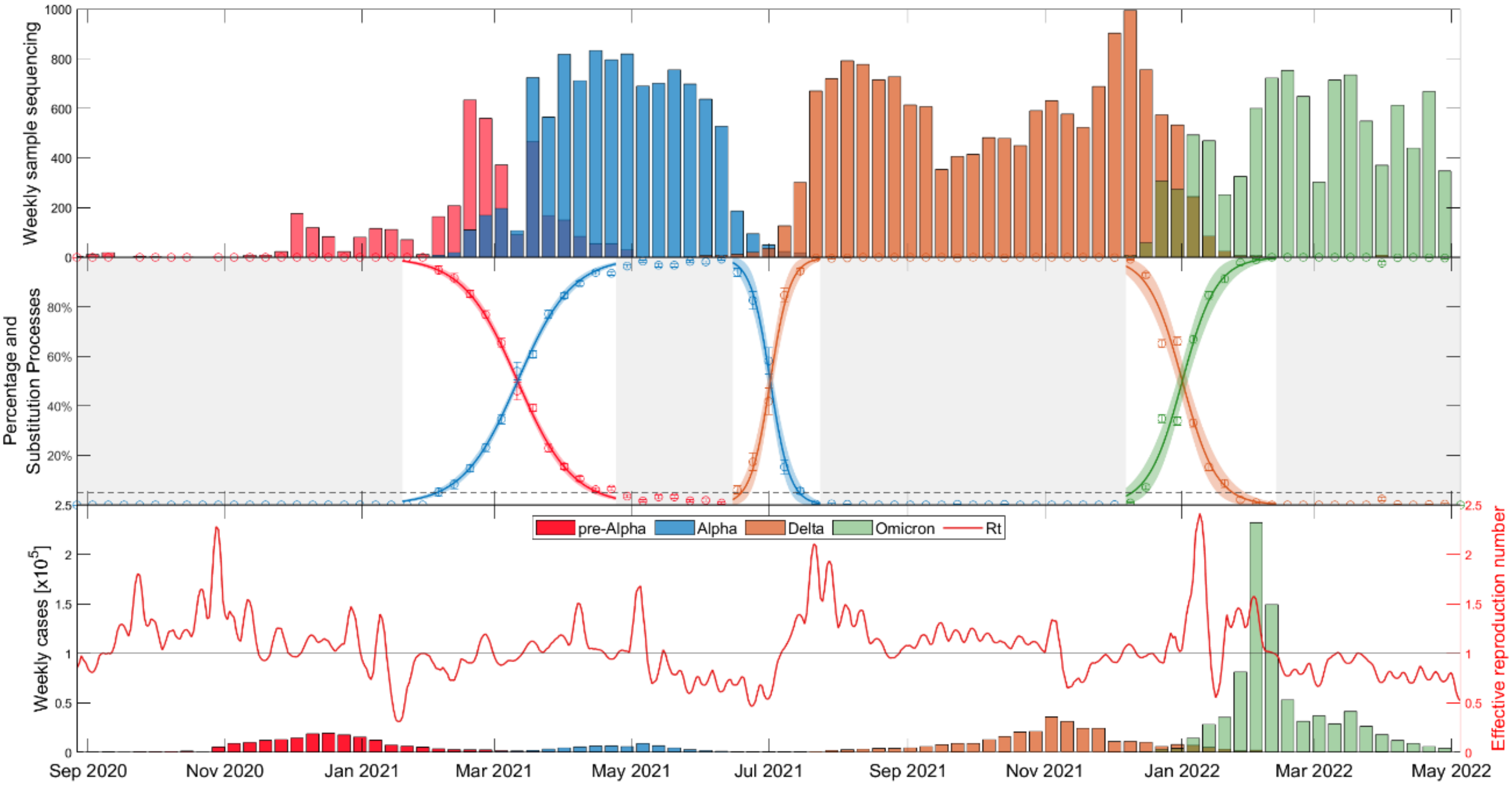

Figure S53 Temporal changes in the dominance of Alpha, Delta, and Omicron COVID-19 variants for two-variant substitution processes in Lithuania over time. Lithuania do not report sample sequencing from May 2022.

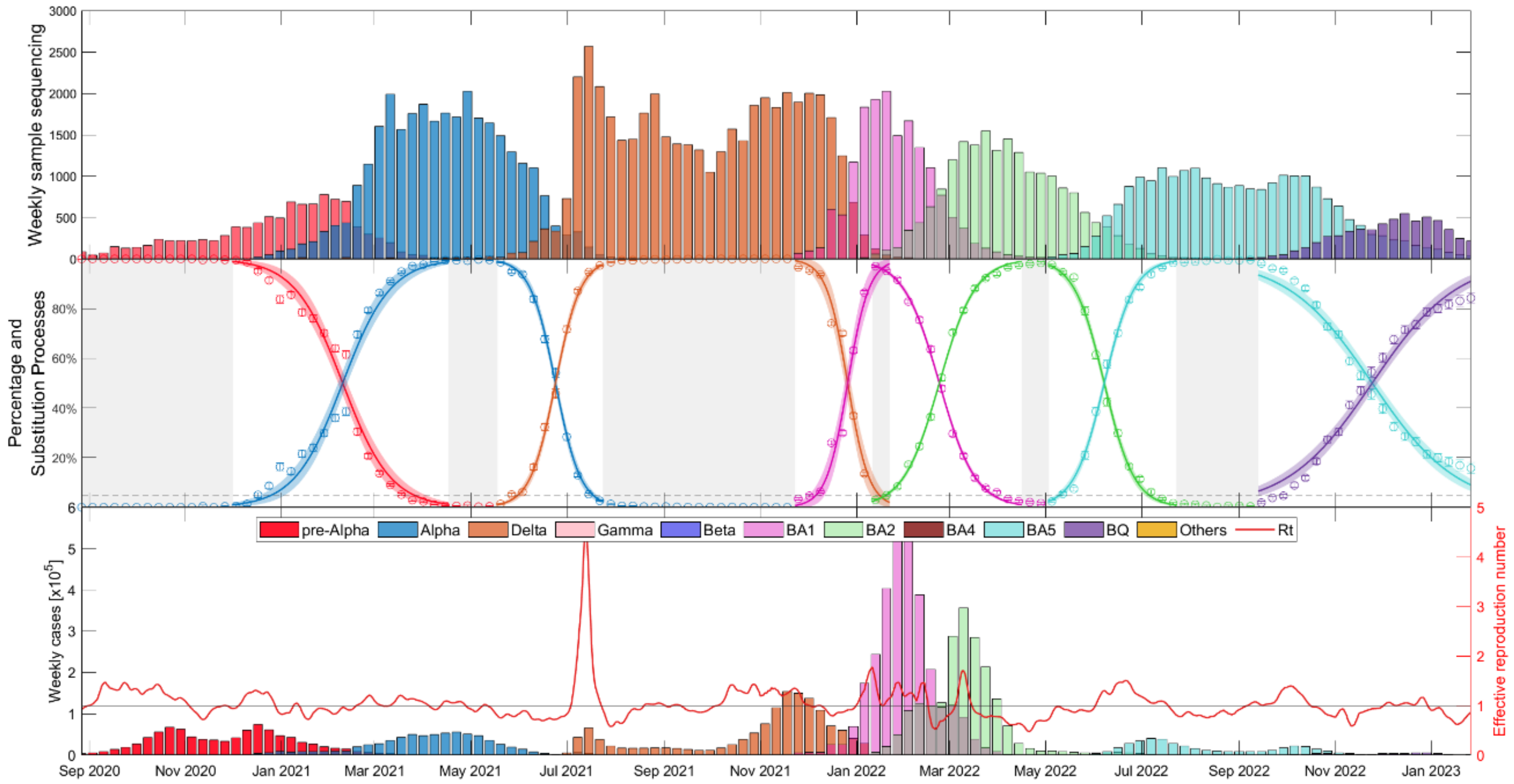

Figure S54 Temporal changes in the dominance of Alpha, Delta, and Omicron lineages (BA.1, BA.2, BA.5, and BQ.1) COVID-19 variants for two-variant substitution processes in Netherlands over time.

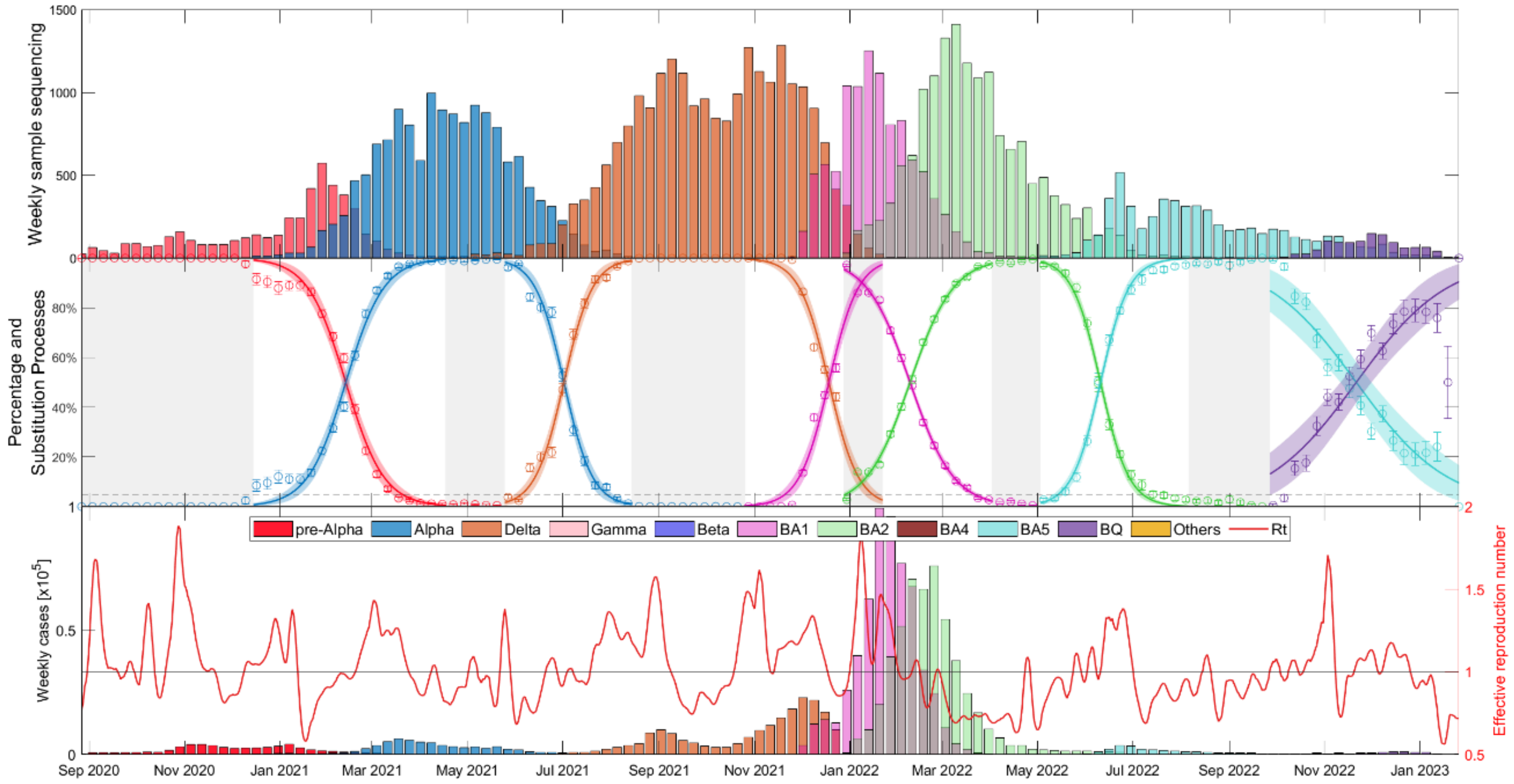

Figure S55 Temporal changes in the dominance of Alpha, Delta, and Omicron lineages (BA.1, BA.2, BA.5, and BQ.1) COVID-19 variants for two-variant substitution processes in Norway over time.

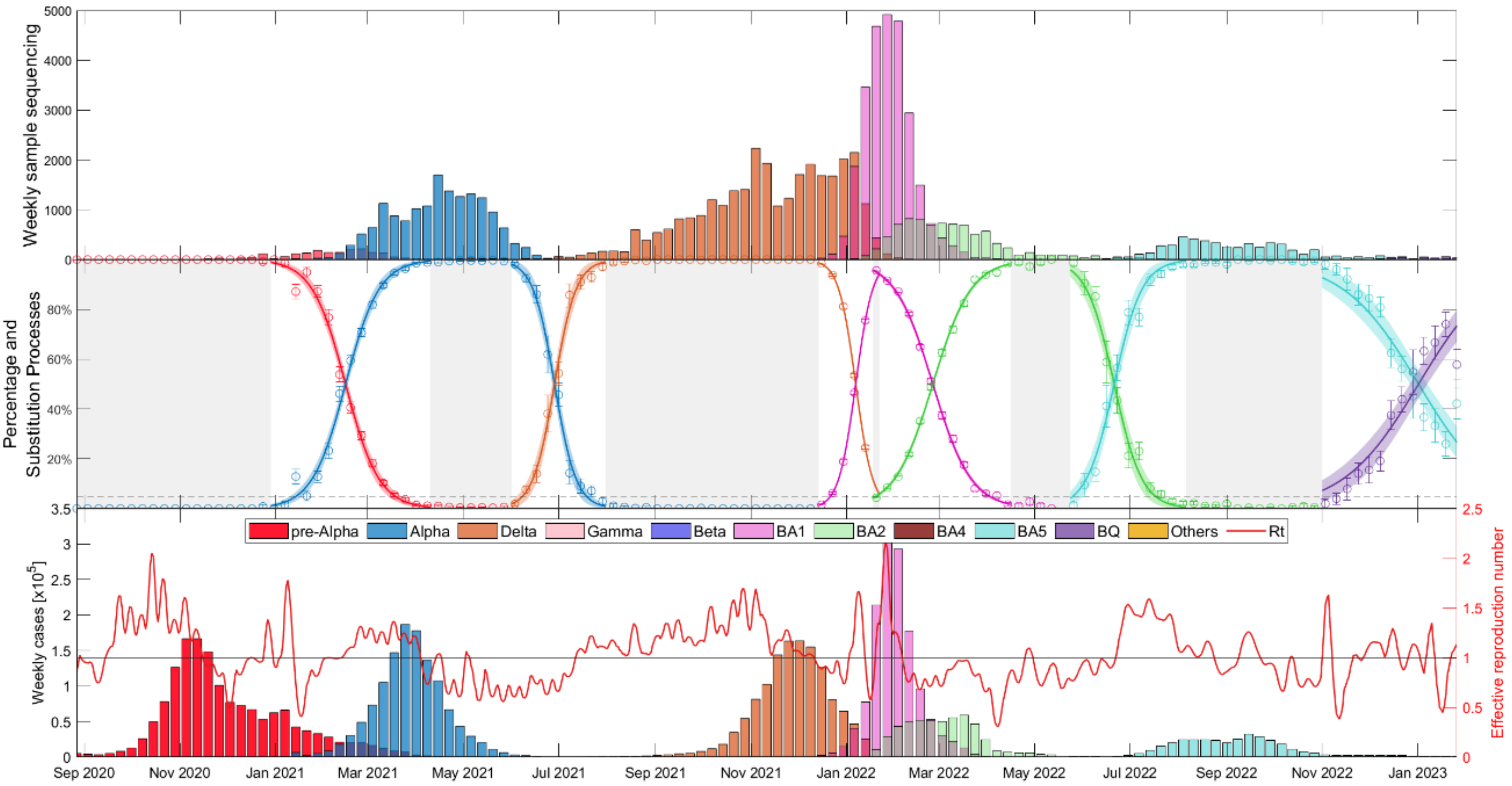

Figure S56 Temporal changes in the dominance of Alpha, Delta, and Omicron lineages (BA.1, BA.2, BA.5, and BQ.1) COVID-19 variants for two-variant substitution processes in Poland over time.

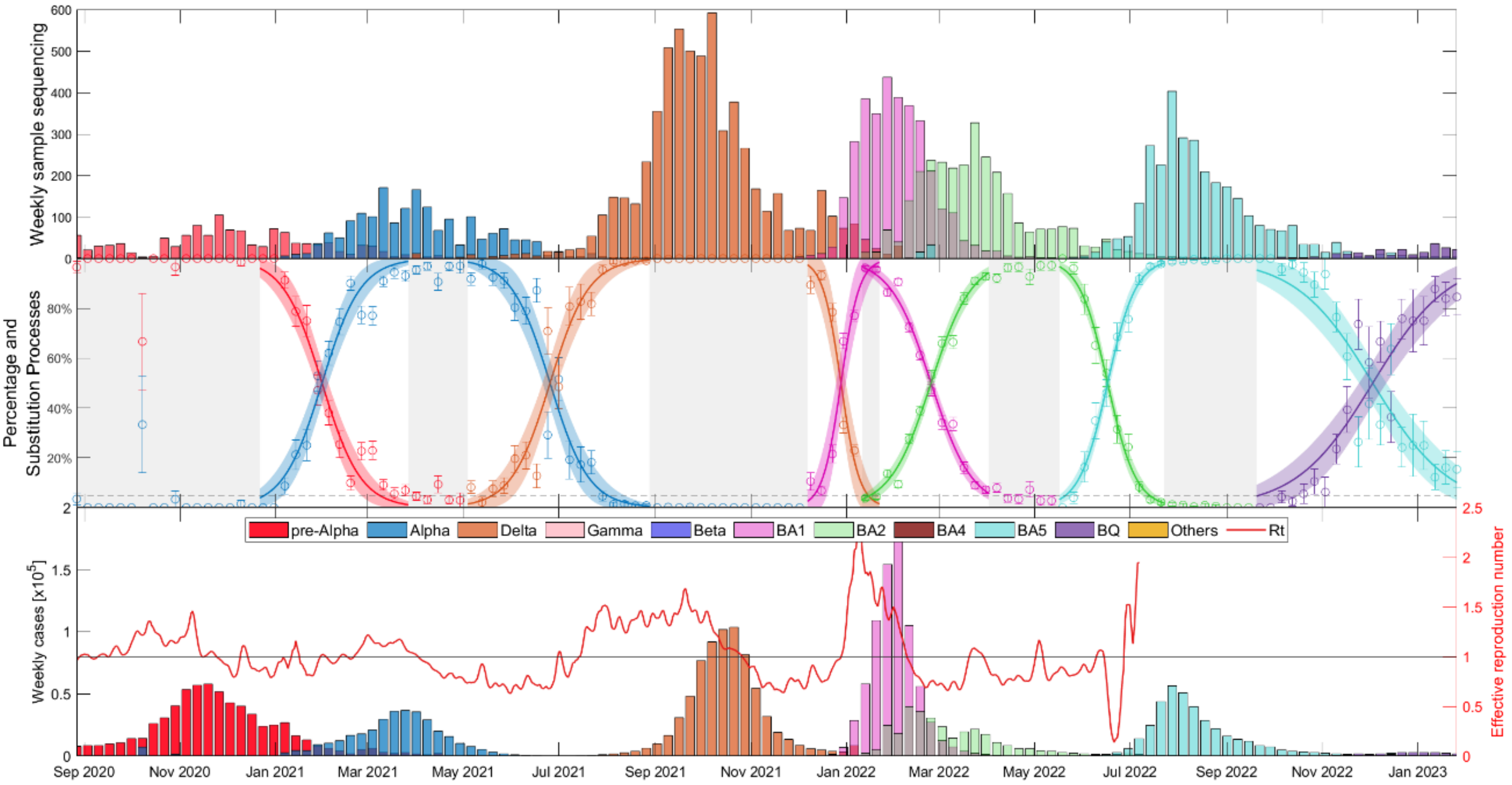

Figure S57 Temporal changes in the dominance of Alpha, Delta, and Omicron lineages (BA.1, BA.2, BA.5, and BQ.1) COVID-19 variants for two-variant substitution processes in Romania over time.

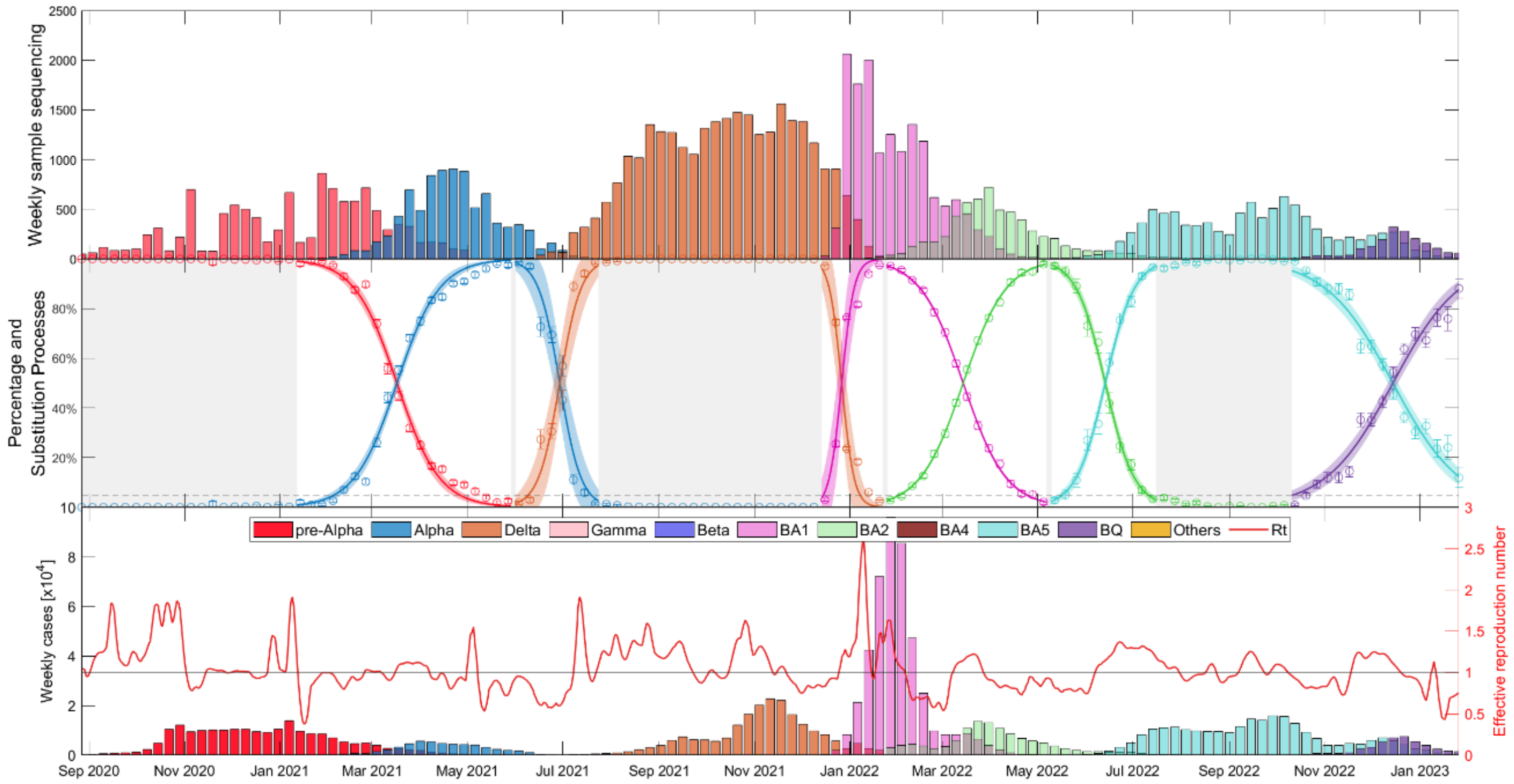

Figure S58 Temporal changes in the dominance of Alpha, Delta, and Omicron lineages (BA.1, BA.2, BA.5, and BQ.1) COVID-19 variants for two-variant substitution processes in Slovenia over time.

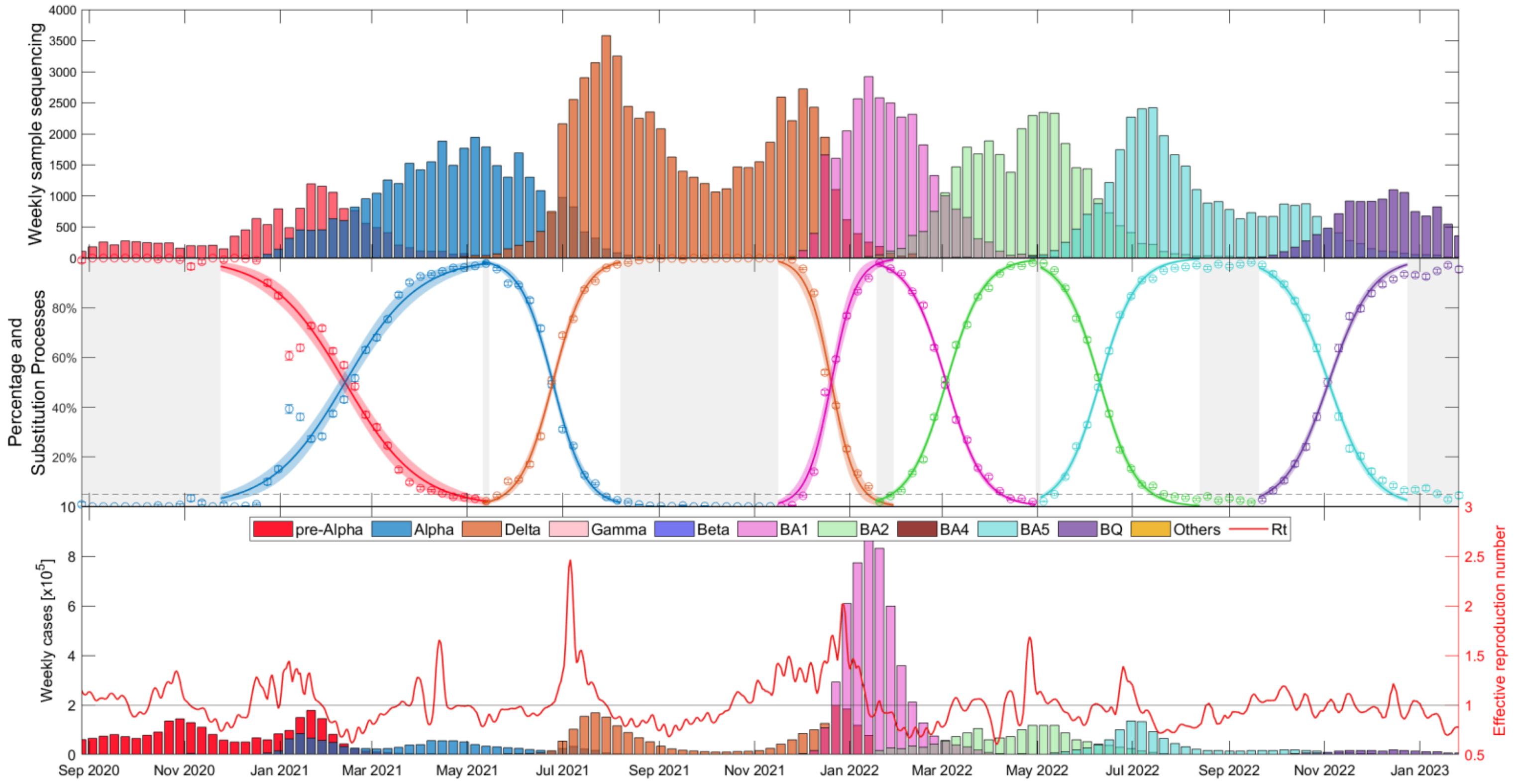

Figure S59 Temporal changes in the dominance of Alpha, Delta, and Omicron lineages (BA.1, BA.2, BA.5, and BQ.1) COVID-19 variants for two-variant substitution processes in Spain over time.

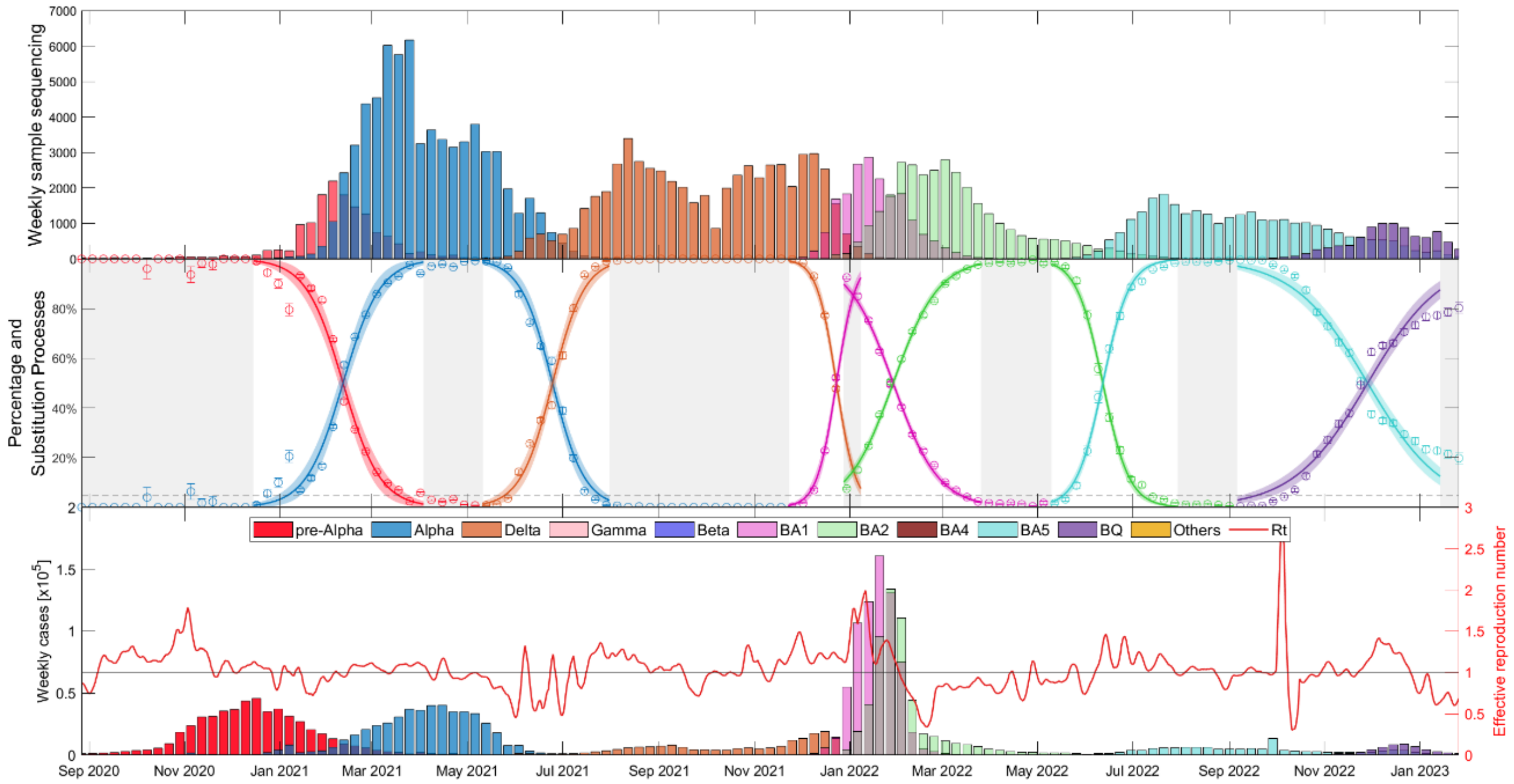

Figure S60 Temporal changes in the dominance of Alpha, Delta, and Omicron lineages (BA.1, BA.2, BA.5, and BQ.1) COVID-19 variants for two-variant substitution processes in Sweden over time.

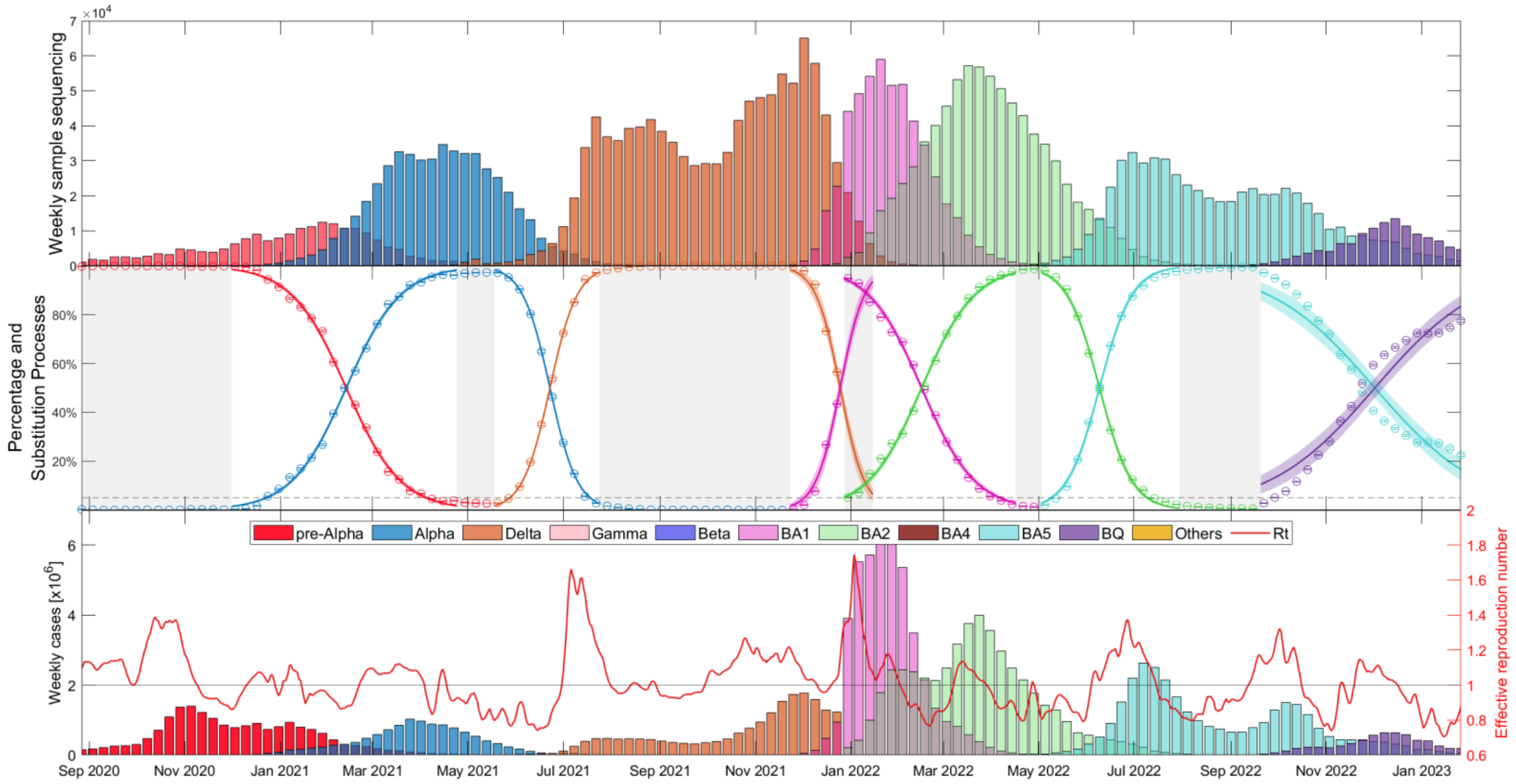

Figure S61 Temporal changes in the dominance of Alpha, Delta, and Omicron lineages (BA.1, BA.2, BA.5, and BQ.1) COVID-19 variants for two-variant substitution processes in Europe over time.

Suppl. Material Text S8 – Detail of each substitution process for Spain

The following six figures offer a detailed, individual analysis of each of the six substitutions (Alpha, Delta, BA.1, BA.2, BA.5, and BQ.1) for Spain. These visuals essentially elaborate on the images from Section S6, providing a breakdown of each substitution rather than a chronological sequence.

In addition, each figure outlines the selected model for each variant as per our coding system. For instance, in the second figure, all the involved variants are denoted as "Model", indicating that Eq. 2 is employed for these variants in our code, which results in the visible waveforms.

However, in the third and fourth figures, we can observe that the dominant Variants of Concern (Delta, BA.1, BA.2) are classified as "Model", while the "Other" package of variants is labeled as "Linear". This classification is based on the marginal importance of these latter variants' behavior, exhibiting either a slow and constant increase (as seen in the Delta VS BA.1 figure) or a slow and constant decrease (as in the BA.1 vs BA.2 figure).

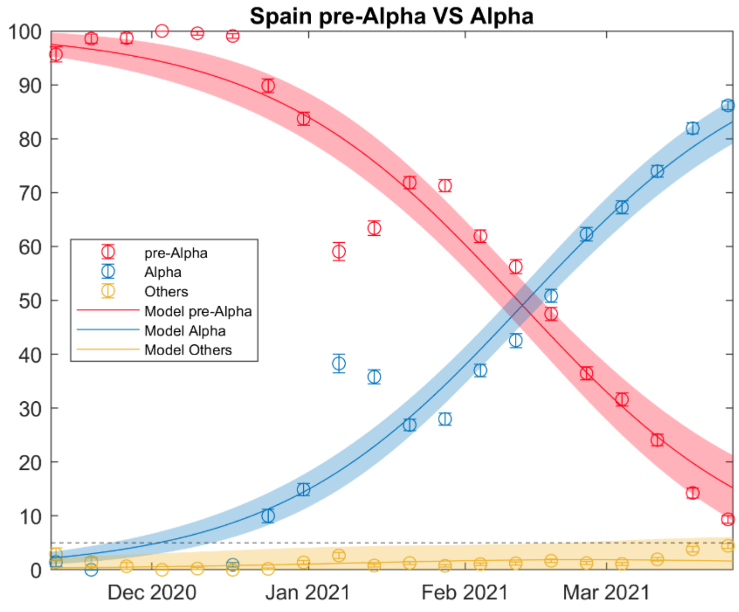

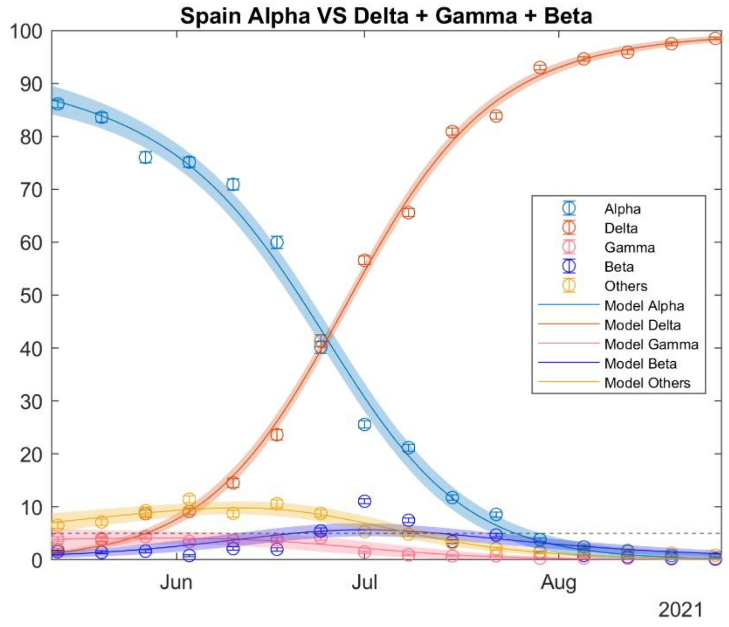

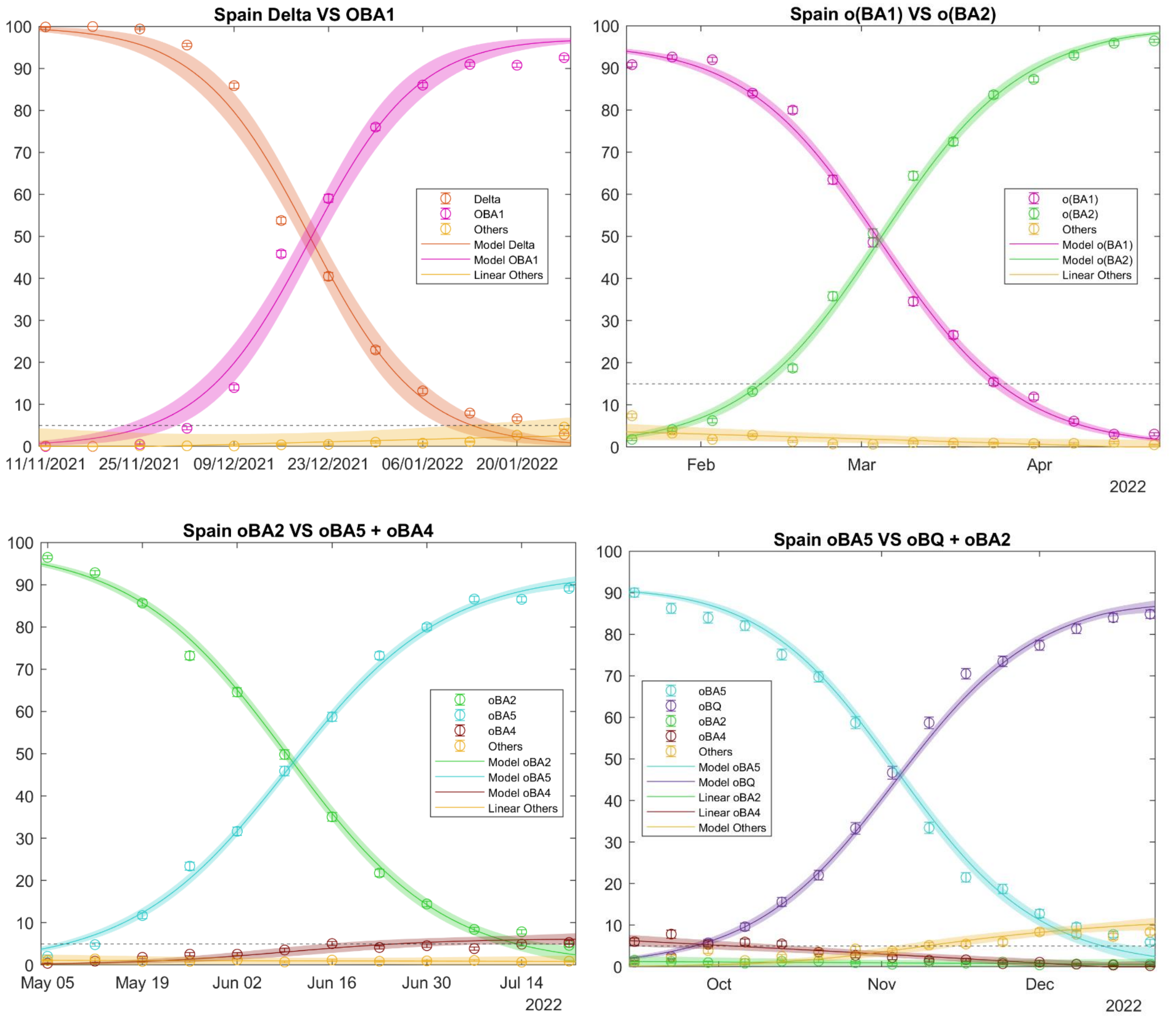

Figure S62 Detail of the different six substitutions processes of Spain. These images compound the substitution on Figure 2 in the main text or the Figure S39 in the Suppl. Material Text S6 .

Suppl. Material Text S9 – Additional analyses of transmissibility in relation to population size, density, and vaccination status

Building on Section 3.2.3 and Figures 5 and 6 of the main text, this supplementary analysis explores the relationship between the increase in transmissibility, Δβ and two other factors: population size and population density.

We begin by examining the relationship between transmissibility increases Δβ and the size of a country's population. The plots in Figure S63 of the supplementary materials mirror those in Figure 5 but relate to the logarithm of the countries' population. Once again, we've divided countries into two clusters: those with larger populations (cluster 2) and those with smaller ones (cluster 1), maintaining a similar relationship between these two groups as in the previous section. For this analysis, the two clusters are defined by countries with a population greater than 15 million. This categorization results in Germany, France, Italy, Spain, Poland, Romania, and the Netherlands (in decreasing order of population) being classified as more populated, with the remaining countries deemed less populated. The results are largely the same when the threshold is increased to 20 million, in which case Germany, France, Italy, Spain, and Poland are considered more populated. These findings echo those concerning the geographical area of the countries.

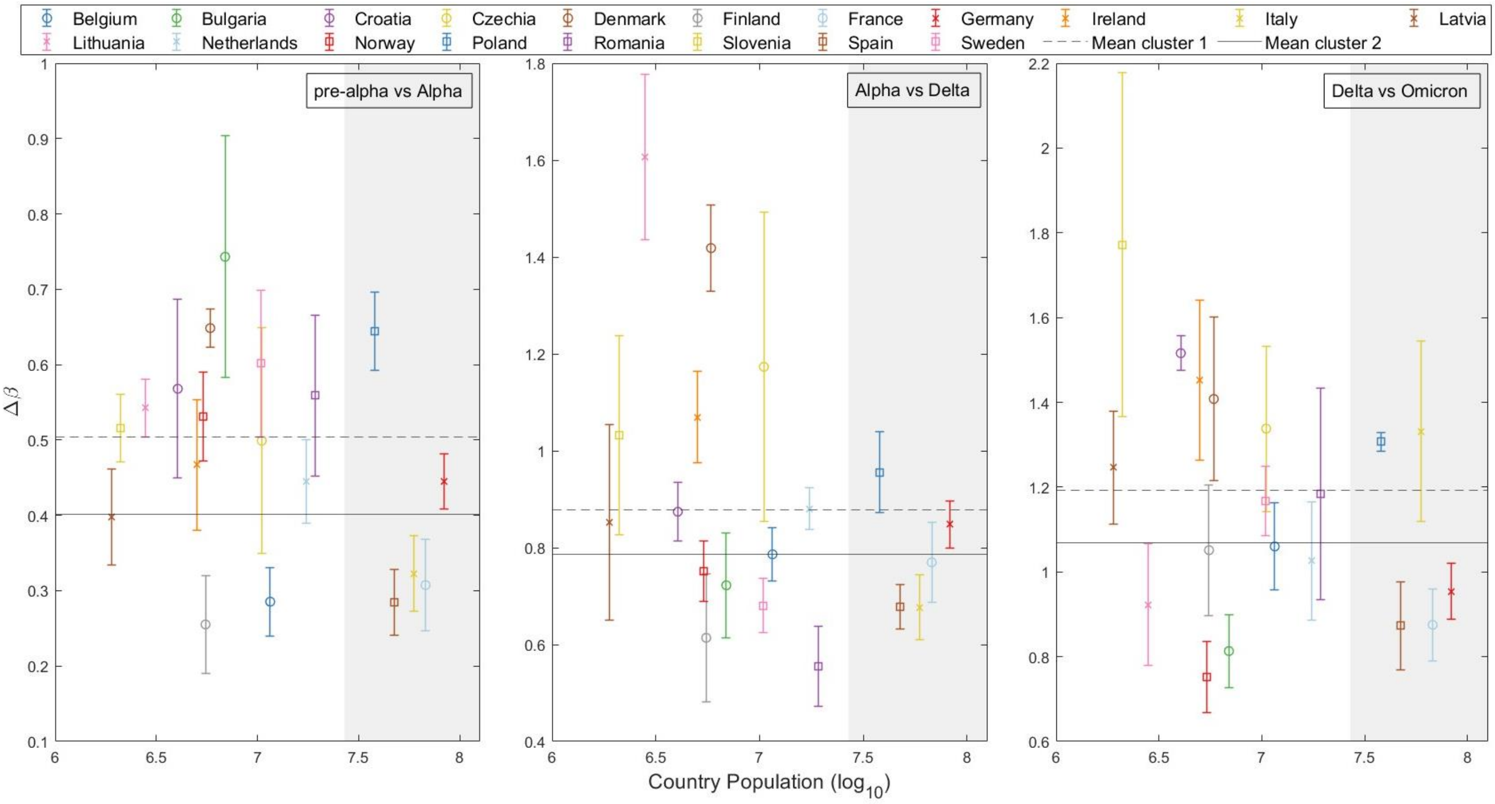

Figure S63 Increase in transmissibility ($\Delta\beta$) plotted against the log of the country's population, distinguishing two primary clusters: smaller populations (cluster 1) on a white background, and larger populations (cluster 2) on a gray background. Mean $\Delta\beta$ for both clusters are depicted in each substitution with horizontal lines (cluster 1: Dashed line; cluster 2: Solid line).

Next, we apply a similar approach to explore the potential correlation between transmissibility and population density. When we divide countries based on a density threshold of 110 inhabitants per km², the countries with the highest population densities

are the Netherlands, Belgium, Luxembourg, Germany, Italy, Denmark, Czechia, Poland, Portugal, and Slovakia (listed in descending order of density). However, in this case, any potential correlations seem to dissolve completely. Each variant substitution leads to a different relationship between the average transmission rates of more densely and less densely populated countries. Regardless of the substitution being considered, both the mean and median Δβ values exhibit considerable variability, suggesting no apparent connection between Δβ and population density.

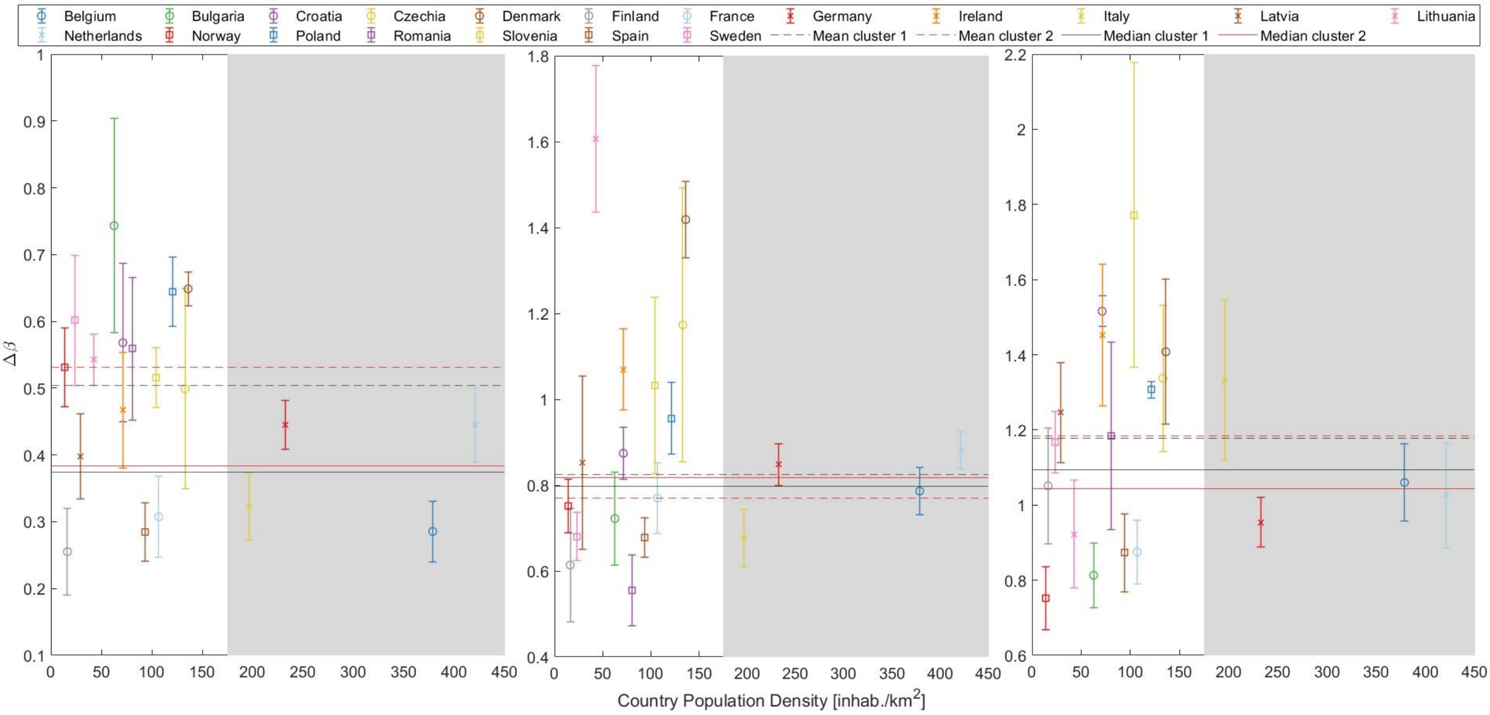

Figure S64 Increase in transmissibility ($\Delta\beta$) plotted against the country's population density, distinguishing two primary clusters: smaller densities (cluster 1) on a white background, and larger densities (cluster 2) on a gray background. Mean and median $\Delta\beta$ for both clusters are depicted in each substitution with horizontal lines (cluster 1: Dashed line; cluster 2: Solid line; mean in red and median in black).

Finally, paralleling Section 3.2.4 of the main text, we look at the relationship between Δβ and the percentage of the fully vaccinated population. The supplementary Figure S64 shows the pre-Alpha to Alpha substitution (left) and the Delta to Omicron substitution (right). For the former, no clear trend emerges, likely because most countries had not begun their vaccination campaigns at the time of this transition. However, the latter substitution shows a different picture. Given that the Omicron variant was seemingly unaffected by the level of vaccination, thus the number of susceptible individuals was effectively total, we find no correlation between Δβ and the percentage of the population fully vaccinated.

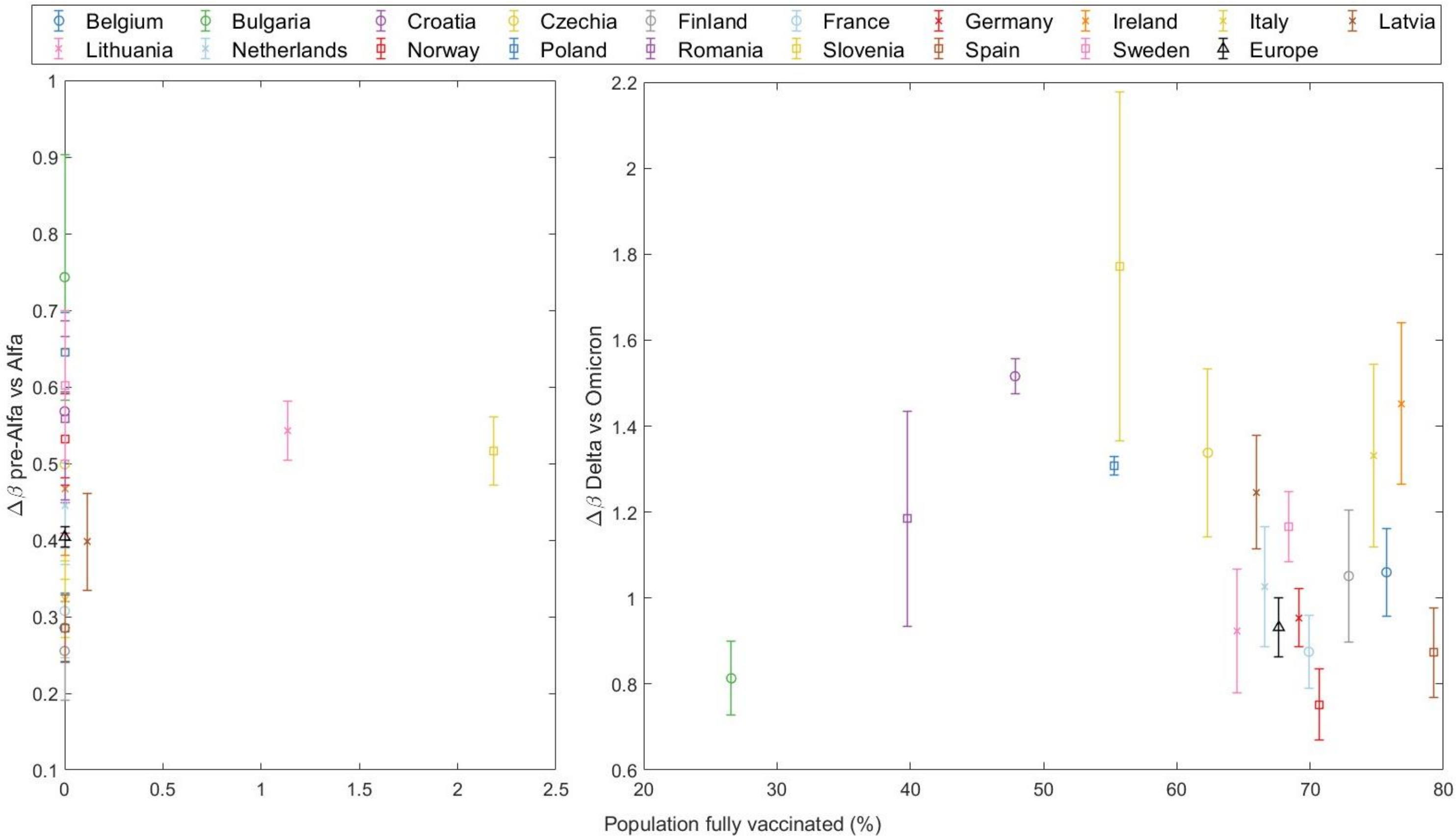

Figure S65 The increase in transmissibility $\Delta\beta$ is plotted against the percentage of fully vaccinated individuals at the beginning of the: (left) pre-Alpha-Alpha substitution (Alpha>5%) and (right) Delta-Omicron substitution (Omicron>5%). No extra line or marked is shown because no trend or relation between the two variables are found.

Suppl. Material Text S10 – Transmissibility differences among Omicron lineages using GISAID data source

In the main article, we were able to relate the different $\Delta\beta$ parameters of the model for each substitution and country to various characteristics such as the entry dates of the new variant, surface area, vaccination status of the population, and more. The main focus of the article is on the major substitutions of the Alpha, Delta, and Omicron variants, as detailed in the various subsections of Section 3.2.

In this part of the Supplementary Material, we present the same analyses as those mentioned above, but for all six substitutions (Alpha, Delta, BA.1, BA.2, BA.5, and BQ.1). We employ the same methods, such as weighted box plots, the Spearman Correlation Test, R^2 by error weights, and cluster distinction. These are the analyses associated with the entire study and all the figures of the different countries (and Europe) found in Suppl. Material Text S6 .

However, instead of having 19 countries as in the main article, here we have 18. As we have already mentioned in Suppl. Material Text S6 , Lithuania stopped reporting sequenced samples as of May 2022, and thus the substitution processes of BA.2, BA.5, and BQ.1 are missing for this country.

Next, we present the resulting figures from the $\Delta\beta$ analyses in relation to different characteristics. A brief commentary follows each figure, discussing the results and the similarities or differences with the three main substitutions discussed in the main article.

Relation between the different $\Delta\beta$ values

Figure S65, analogous to Figure 3 in the main article (section 3.2.1), presents the $\Delta\beta$ values along with their associated errors for 18 individual countries, as well as for Europe as a whole, and for the six substitutions (except for the last one in the case of Bulgaria, like we mentioned in Suppl. Material Text S6 , they did not report variant sequencing during these months). Additionally, for each substitution, we have included a box plot created from the contributions (by weight) of the 18 countries, with its median, and its maximum and minimum percentile values.

From the boxplot, we can infer that data from certain countries might be ineligible for subsequent statistical analysis: Denmark (Delta substitution), Croatia, Ireland, and Slovenia (BA.1 substitution), and Latvia and Spain (BQ.1 substitution).

As clearly evidenced and as stated in the article's conclusion, $\Delta\beta_{\mathrm{Alpha}} < \Delta\beta_{\mathrm{Delta}} < \Delta\beta_{\mathrm{BA.1}}$. However, this progression becomes less clear with the different lineages of the Omicron variant. In fact, we see that: $\Delta\beta_{\mathrm{BA.1}} > \Delta\beta_{\mathrm{BA.2}}$ and $\Delta\beta_{\mathrm{BA.5}} > \Delta\beta_{\mathrm{BQ.1}}$, which could be related to immunity and/or increased protection between variants BA.1 and BA.2. However, the BA.5 subvariants substantially escaped neutralizing antibodies induced by both vaccination and infection (of previous variants) [1]. It is important to remember that we are calculating an increase, so regardless of whether it's greater or lesser than previous waves of variant change, the increase is invariably positive. This means the new variant is always more transmissible than any of those present at the time of the increase, hence always $\Delta\beta > 0$.

It's also worth noting that in some countries, the last substitution to the BQ.1 variant is significantly influenced by the XBB.1.5 variant. This variant grows very quickly in conjunction with BQ.1, which may cause a decrease in $\Delta\beta_{BQ.1}$. It's worth noting that these variants are especially transmissible, with the highest immune escape to date. This new substitution with XBB1.5 should be studied in future studies.

Figure S66 also shows the six box plots for each substitution. As mentioned before, these are calculated from the Δβ values of the 18 countries and their errors. Two points about this figure stand out:

- Once the Omicron variant has arrived, the BA.2, BA.5, and BQ.1 lineages are extremely homogeneous across all European countries. Except for one data point that deviates due to being very fast (Latvia in the BQ.1 vs BA.5 substitution), the rest of the points group within the credible values of the box plot, with very low dispersion.

- The observation stated in Section 3.2.1 of the main article is reconfirmed: the increase in transmissibility values calculated for all of Europe, as a collective, are consistently lower than the average values calculated for each country individually, see Table S1. This supports the previous discussions regarding the interplay between geographical scale and transmissibility in Section 3.2 and 3.3. Essentially, when calculating the increase in transmissibility, the number and spatial-temporal distribution of outbreaks within a given region plays a crucial role, leading larger countries, or regions to exhibit lower Δβ values. The subsequent table presents the values of the six substitutions for Europe as a whole (black triangle), contrasted with the average for the 18 individual countries (red box plot line).

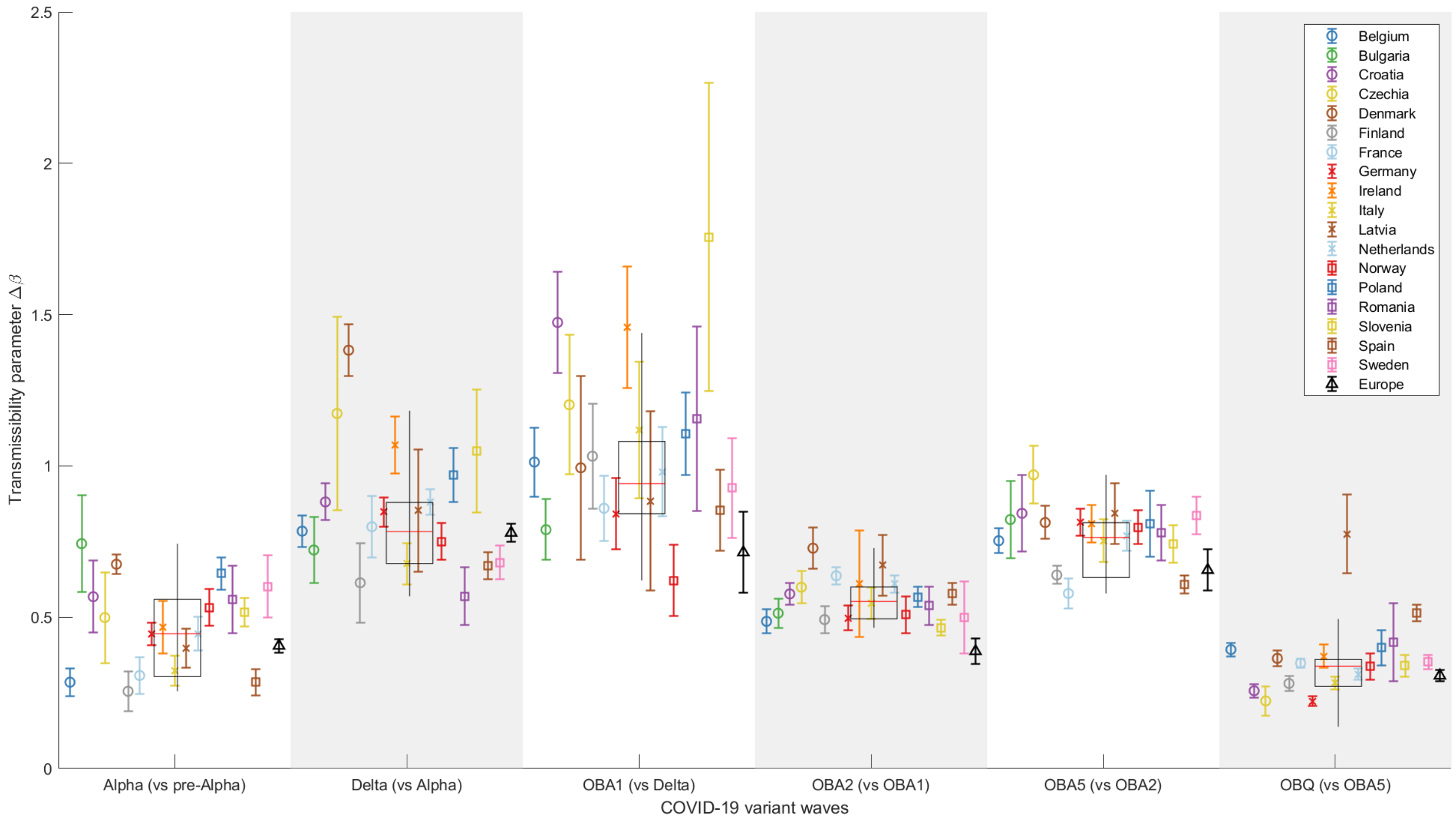

Figure S66 Representation of the increase in transmissibility parameter ($\Delta\beta$) for 18 European countries (circles, crosses, and squares) and Europe (black triangle) as a combination of the studied countries, based on the results for the Alpha, Delta, and Omicron lineages variants. The x-axis simply distinguishes the six COVID-19 variant substitutions, while the y-axis displays the increment on the transmissibility parameter for each country.

Table S1 Results for Europe and for the average of all countries. In all cases, it is verified that $\Delta\beta_{Europe} < \Delta\beta_{Average}$.

	Alpha	Delta	BA.1	BA.2	BA.5	BQ.1
**Europe**	0.406	0.779	0.714	0.388	0.656	0.307
**Average**	0.446	0.783	0.942	0.552	0.764	0.338

Relation between Δβ values and day of emergence

This section complements the 3.2.2 in the main article. The following Figure S67 to Figure S72 show Δβ parameters in relation to the entry day of the variant that will dominate. The first three figures are very similar to Figure 4 of the main article, as they involve the same waves, but they are slightly different because, in this case, Lithuania is not represented. Again, we can highlight the following:

There is a clear trend of Δβ increase the later a new variant enters a country for the Alpha, Delta, BA.1, and BA.5 lineages.

There is a trend, although not statistically significant, of constancy and homogeneity regardless of the day the variant enters, for BA.2 and BQ.1.

This may be due, once again, to what we have previously discussed about immunity and the differences or similarities between BA.1 and BA.2; and BA.5 and BQ.1. Again, it seems that the pattern between these two pairs of different lineages repeats itself.

For each of our cases we have calculated the Spearman correlation coefficient, ρ, and its p-value, besides the coefficient R^2.

The Spearman Correlation test is a non-parametric measure of the monotonic relationship between two variables and does not assume a normal distribution, which is very useful when the assumptions of normality and linearity cannot be met, as we believe is the case here. Its coefficient measures the monotonic relationship between two variables, that is, if the relationship between the variables is nonlinear, but follows a general trend. For each of our cases (and taking into account the previous outlier points) we have calculated the Spearman correlation coefficient, ρ, and its p-value. The R^2 test is calculated in a weighted manner, considering the weights of each point (as the inverse of the error, $w_i = 1/e_i$), i.e.:

$$R^2 = 1 - \frac{\sum_i w_i(\Delta\beta_i - f_i)^2}{\sum_i w_i\left(\Delta\beta_i - \overline{\Delta\beta}\right)^2}, \qquad \text{Eq. 9}$$

where $\Delta\beta_i$ are the transmissibility parameters for each country and substitution, $\overline{\Delta\beta}$ the average of all results for the same substitution and, f_i the value of the increase in transmissibility in the case that it corresponds with the linear regression (on the same day as the corresponding $\Delta\beta_i$).

The results are shown below:

Table S2 Statistical results for trend analysis across different lineage transitions: Alpha, Delta, and Omicron (BA.1, BA.2, BA.5, and BQ.1).

	**Alpha**	**Delta**	**BA.1**	**BA.2**	**BA.5**	**BQ.1**
$\boldsymbol{\rho_{5\%}}$	0.614	0.696	0.582	-0.032	0.646	0.088
p-value	0.008	0.002	0.018	0.899	0.003	0.735
$\mathbf{R^2}$	0.49	0.33	0.39	0.01	0.51	0.10

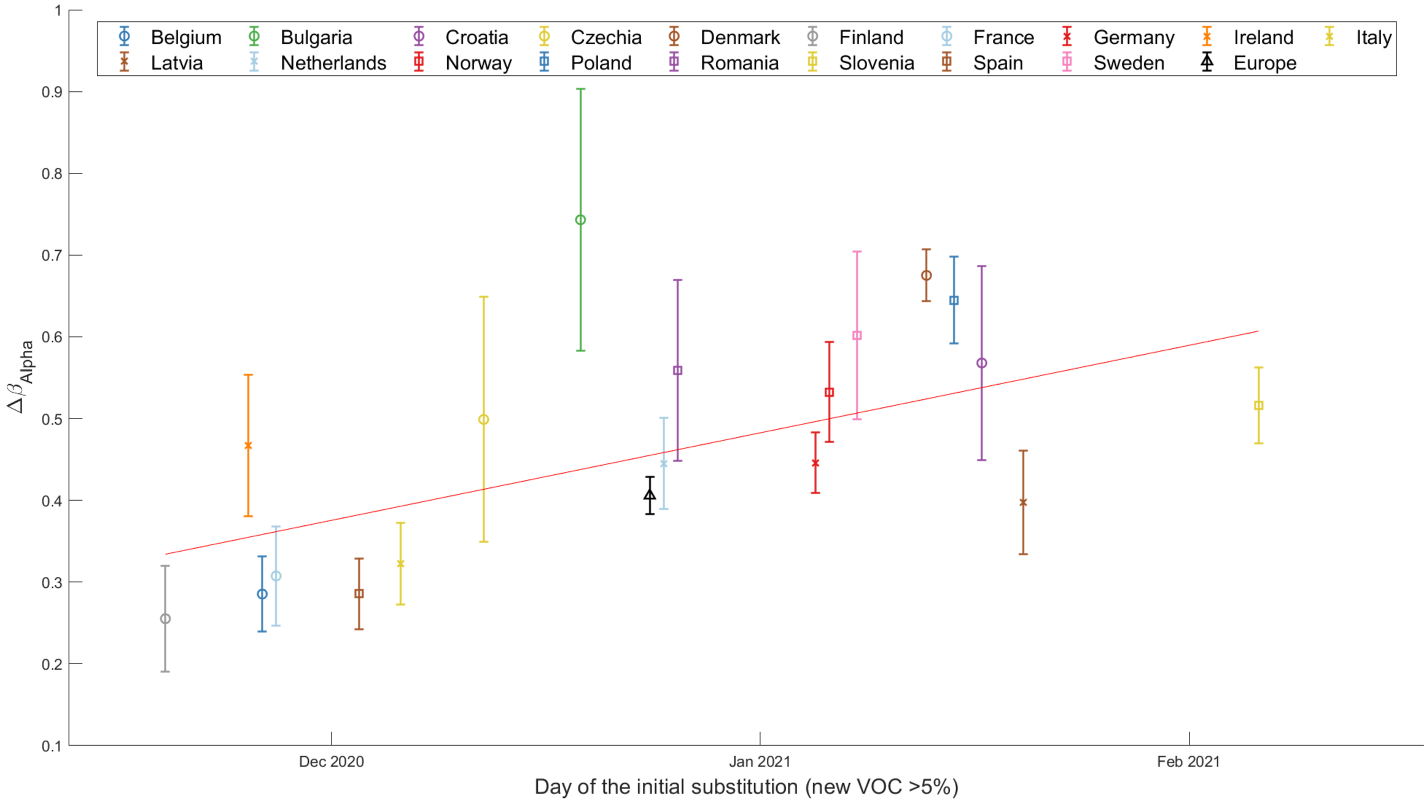

Figure S67 The increase in transmissibility ($\Delta\beta$) based on the day the emerging variant Alpha exceeded 5% according to our substitution model.

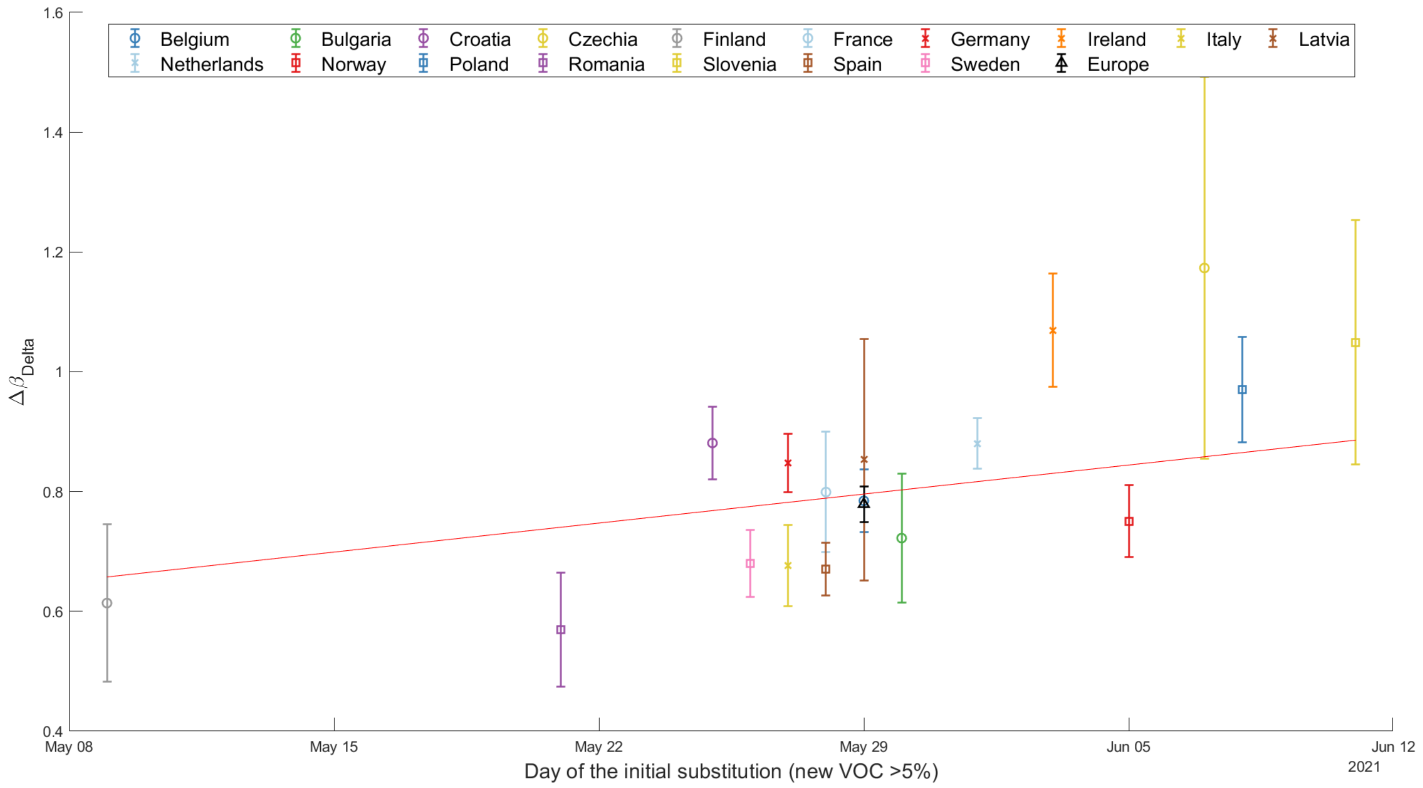

Figure S68 The increase in transmissibility ($\Delta\beta$) based on the day the emerging variant Delta exceeded 5% according to our substitution model. Denmark is not on the statistics.

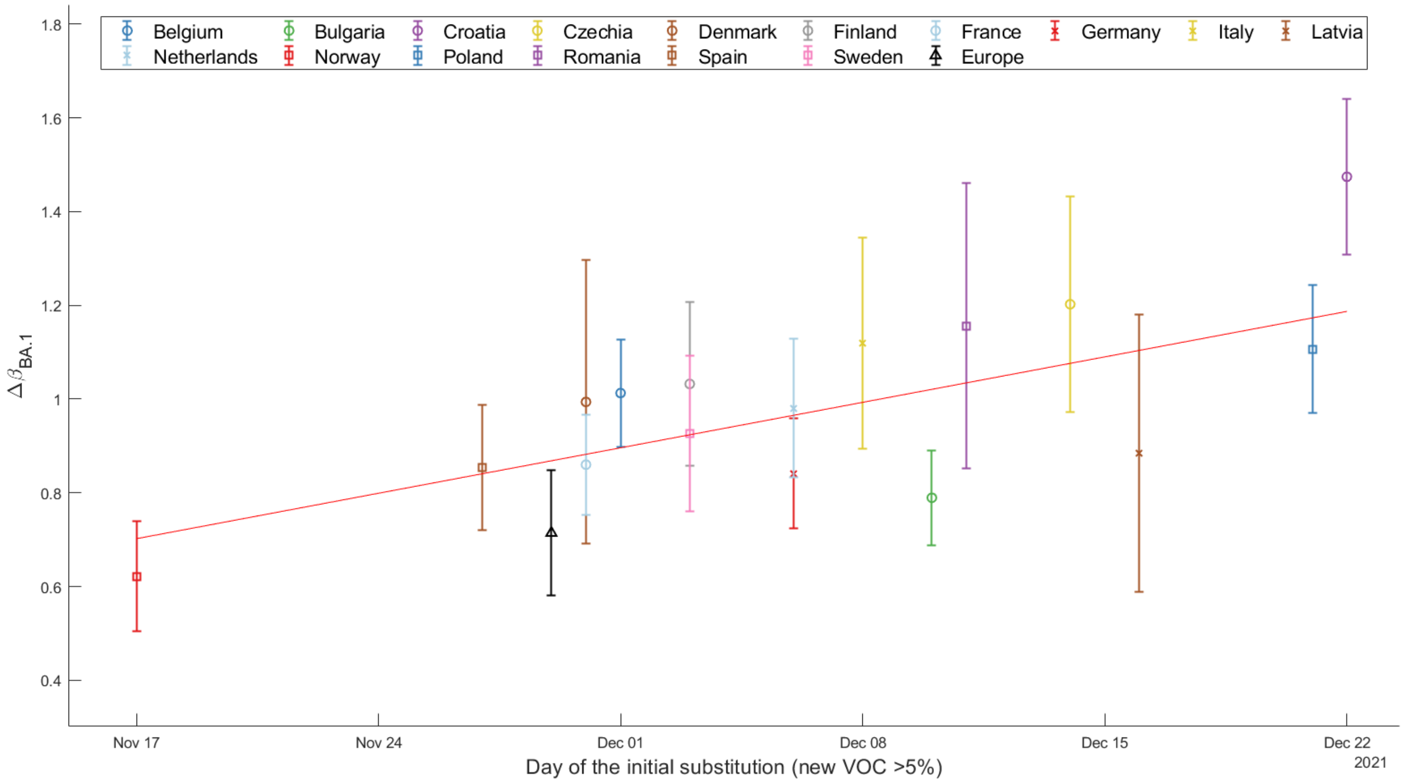

Figure S69 The increase in transmissibility ($\Delta\beta$) based on the day the emerging variant BA.1 exceeded 5% according to our substitution model. Ireland and Slovenia are not on the statistics.

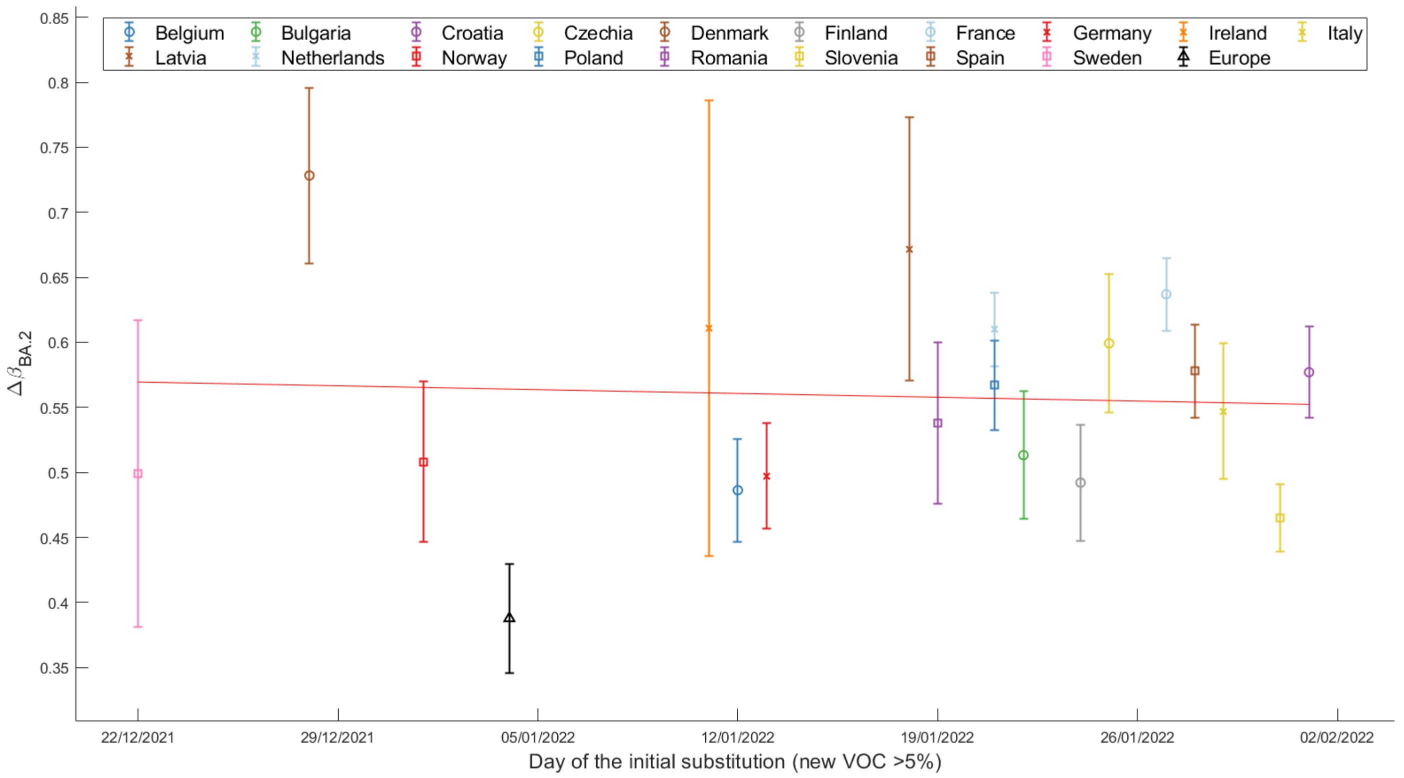

Figure S70 The increase in transmissibility ($\Delta\beta$) based on the day the emerging variant BA.2 exceeded 5% according to our substitution model.

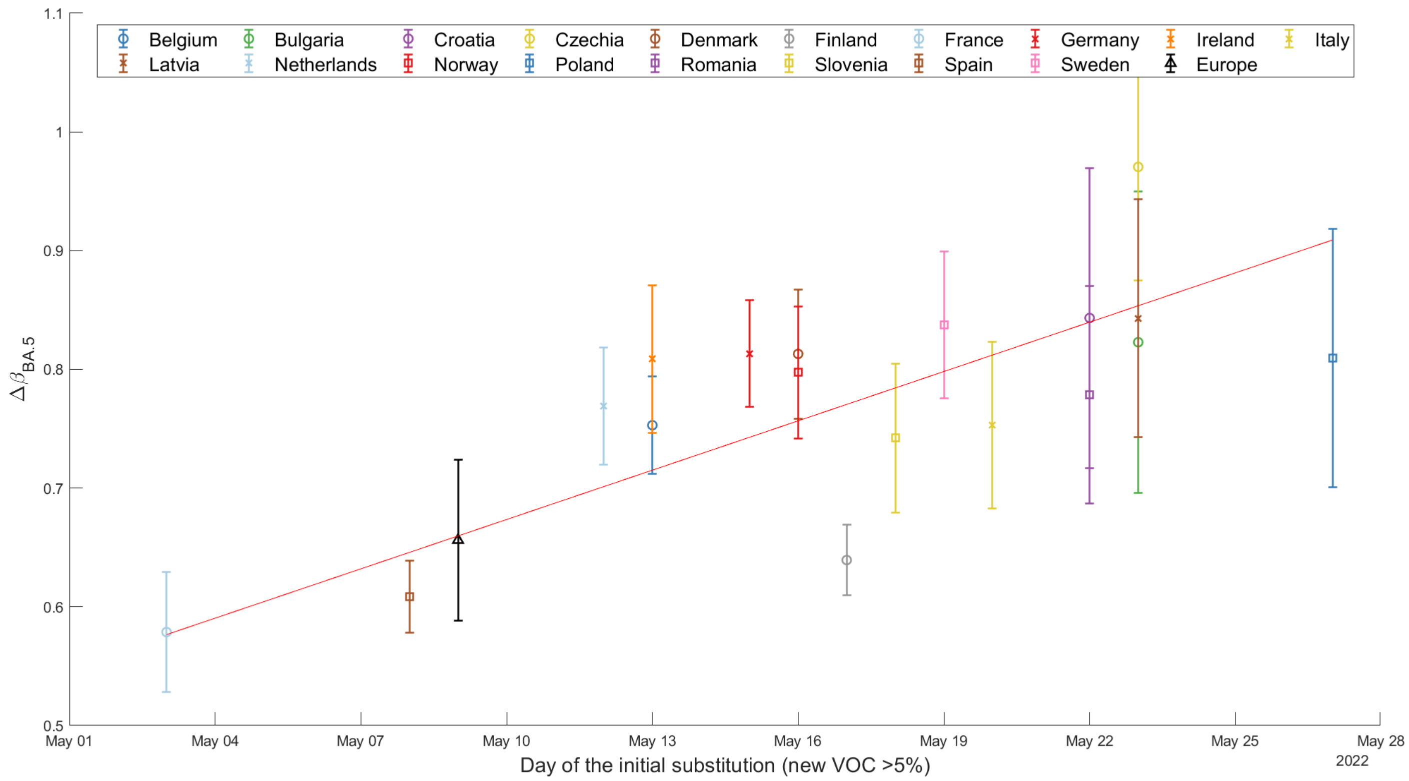

Figure S71 The increase in transmissibility ($\Delta\beta$) based on the day the emerging variant BA.5 exceeded 5% according to our substitution model.

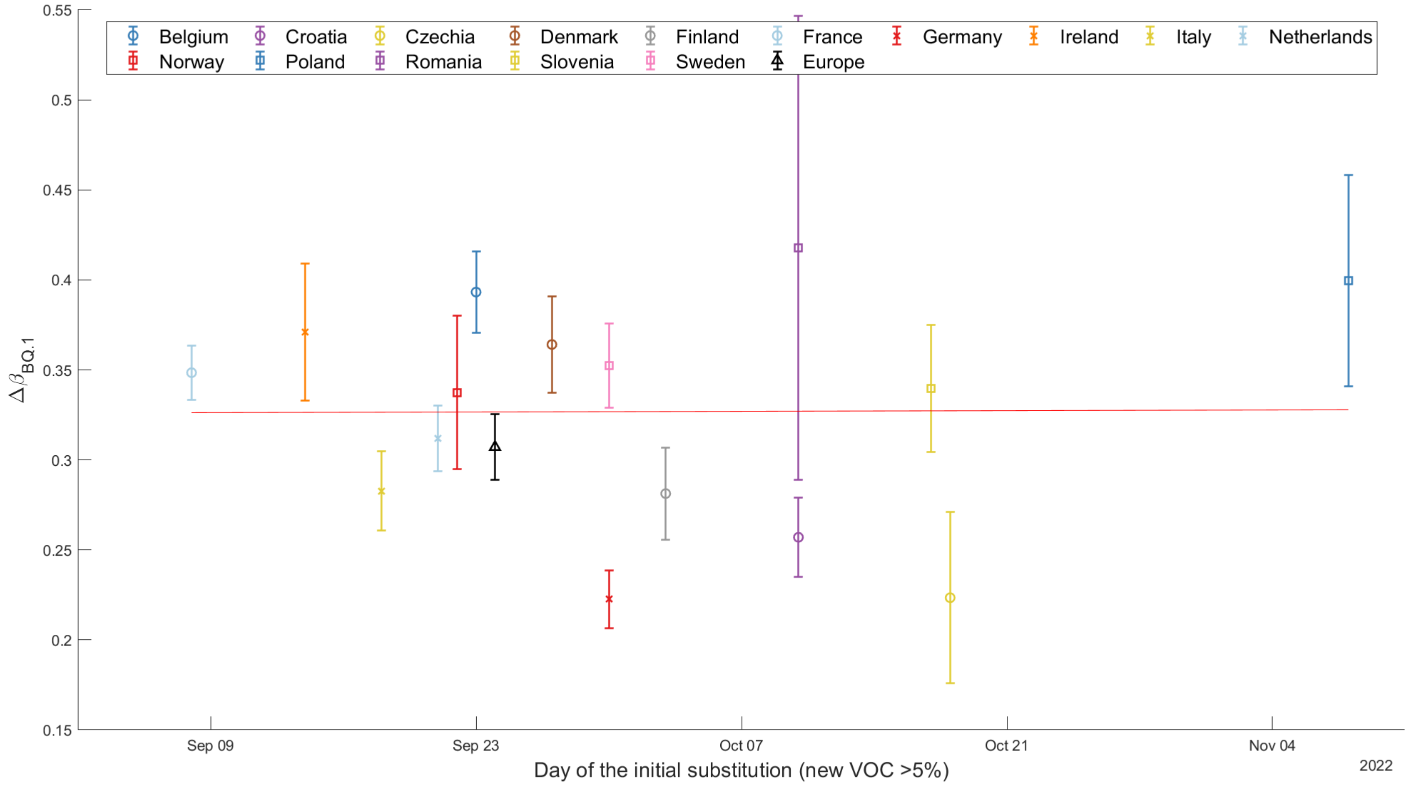

Figure S72 The increase in transmissibility ($\Delta\beta$) based on the day the emerging variant BQ.1 exceeded 5% according to our substitution model. Bulgaria is not represented because did not report variant sequencing during these months. Latvia and Spain are not on the statistics

Relation between Δβ values and country surface area or population

The following section showcases a set of six pairs of figures that present the increase in transmissibility parameter Δβ for various SARS-CoV-2 variants relative to population and surface area (both on a logarithmic scale) for each of the six substitutions. It pretends to be an extension for section 3.2.3 of the main article. These six figures, Figure S73-Figure S78, parallel Figure 5 of the main paper (Δβ with respect to the country's surface area) and Figure S63 of Suppl. Material Text S9 (Δβ with respect to the country's population). As previously noted, Lithuania is not represented due to the lack of sequenced samples from the entry of the BA.2 variant. Upon inspection and analysis of Figures S72-S77, we can highlight the following:

- As we have previously observed in the main paper (surface area) and in the prior section of the Supplementary Material (population), for the substitutions of Alpha, Delta, and Omicron (BA.1 in this case), larger and more populous countries consistently confirm a slightly lower Δβ.
- This trend now includes the BA.5 and BQ.1 variants (to a lesser extent).
- The outcome for the BA.2 variant does not present a clear difference between large/small countries or highly/lowly populated ones.

Once again, as has been recurrent throughout the study, the Alpha, Delta, and some Omicron variants (BA.1 and BA.5 in particular) bear striking resemblances to each other and the trends are always similar. As has happened before, the BA.2 lineage behaves distinctly differently, and in this case, while the BQ.1 also tends towards a lower Δβ for larger/highly populated countries, the difference is negligible.

The influence of previous variants BA.1 and BA.5 on BA.2 and BQ.1, respectively, could once again provide an explanation for understanding this phenomenon.

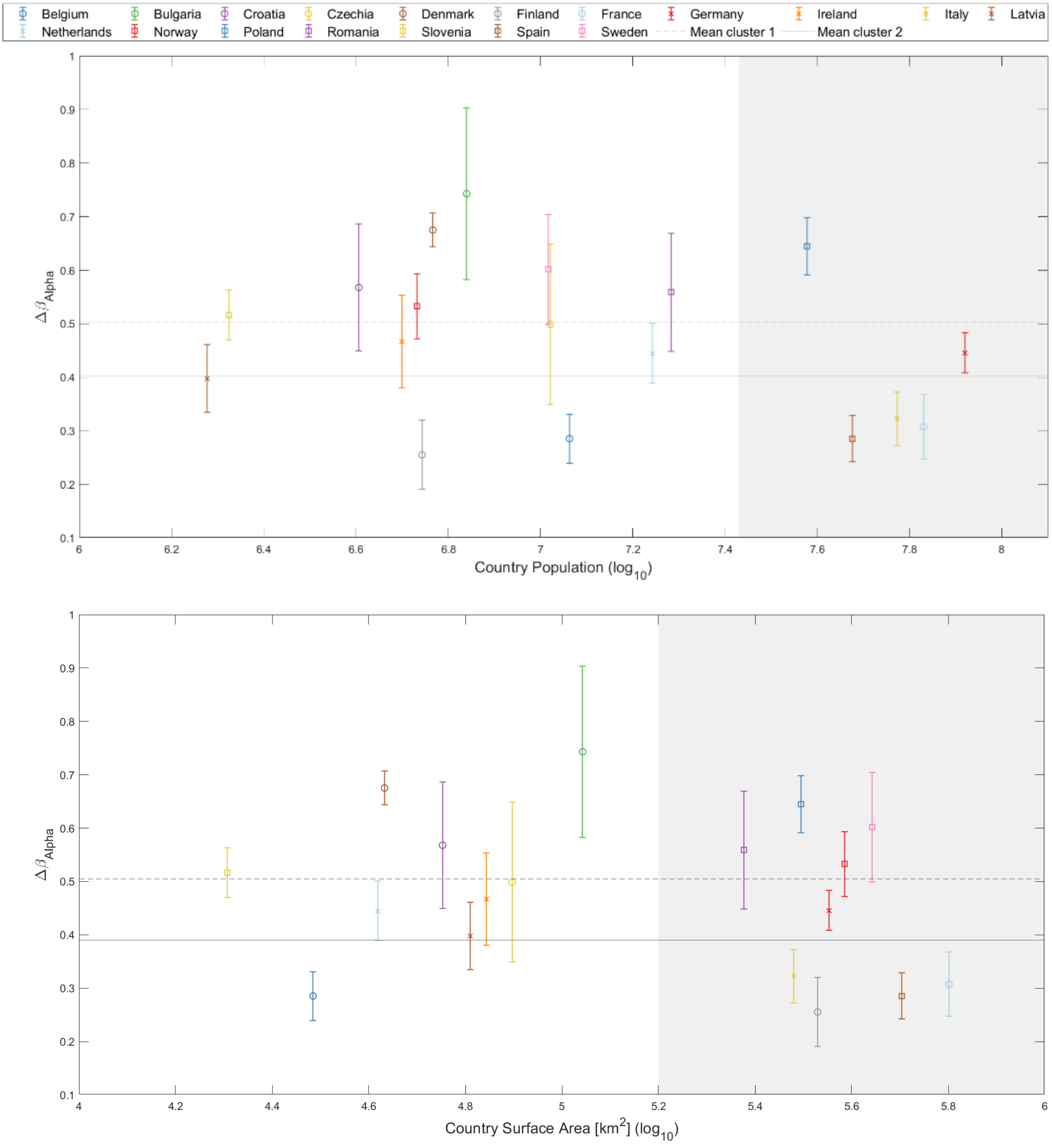

Figure S73 Increase in transmissibility ($\Delta\beta$) of the Alpha variant plotted against: (Top) the logarithm of the country's population and (Bottom) the logarithm of the country's surface area. Both images are divided into two distinct clusters: smaller populations and smaller countries (cluster 1) on a white background, and larger populations and larger countries (cluster 2) on a gray background. The mean $\Delta\beta$ values for both clusters are depicted with horizontal lines (cluster 1: Dashed line; cluster 2: Solid line).

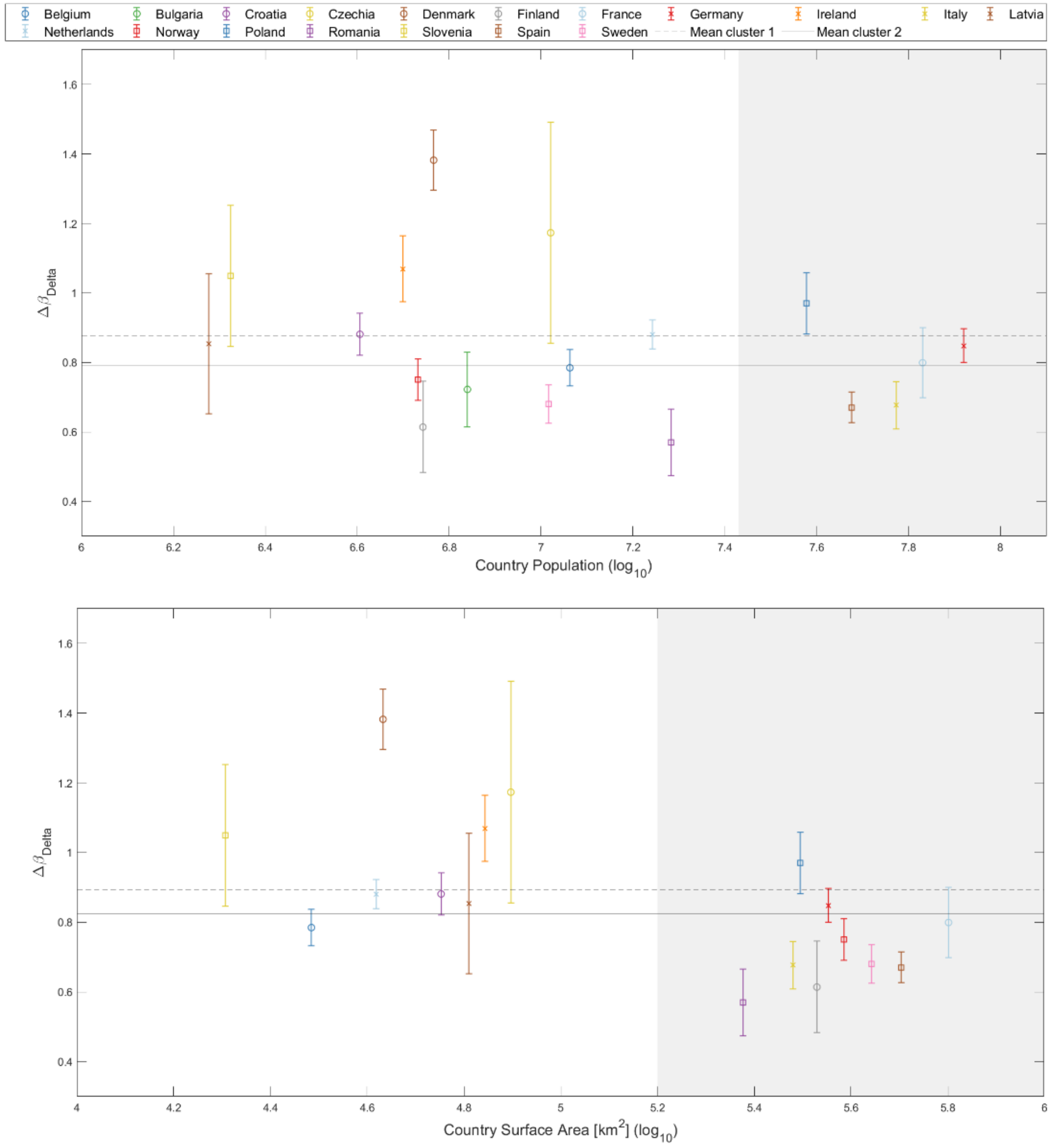

Figure S74 Increase in transmissibility ($\Delta\beta$) of the Delta variant plotted against: (Top) the logarithm of the country's population and (Bottom) the logarithm of the country's surface area. Both images are divided into two distinct clusters: smaller populations and smaller countries (cluster 1) on a white background, and larger populations and larger countries (cluster 2) on a gray background. The mean $\Delta\beta$ values for both clusters are depicted with horizontal lines (cluster 1: Dashed line; cluster 2: Solid line).

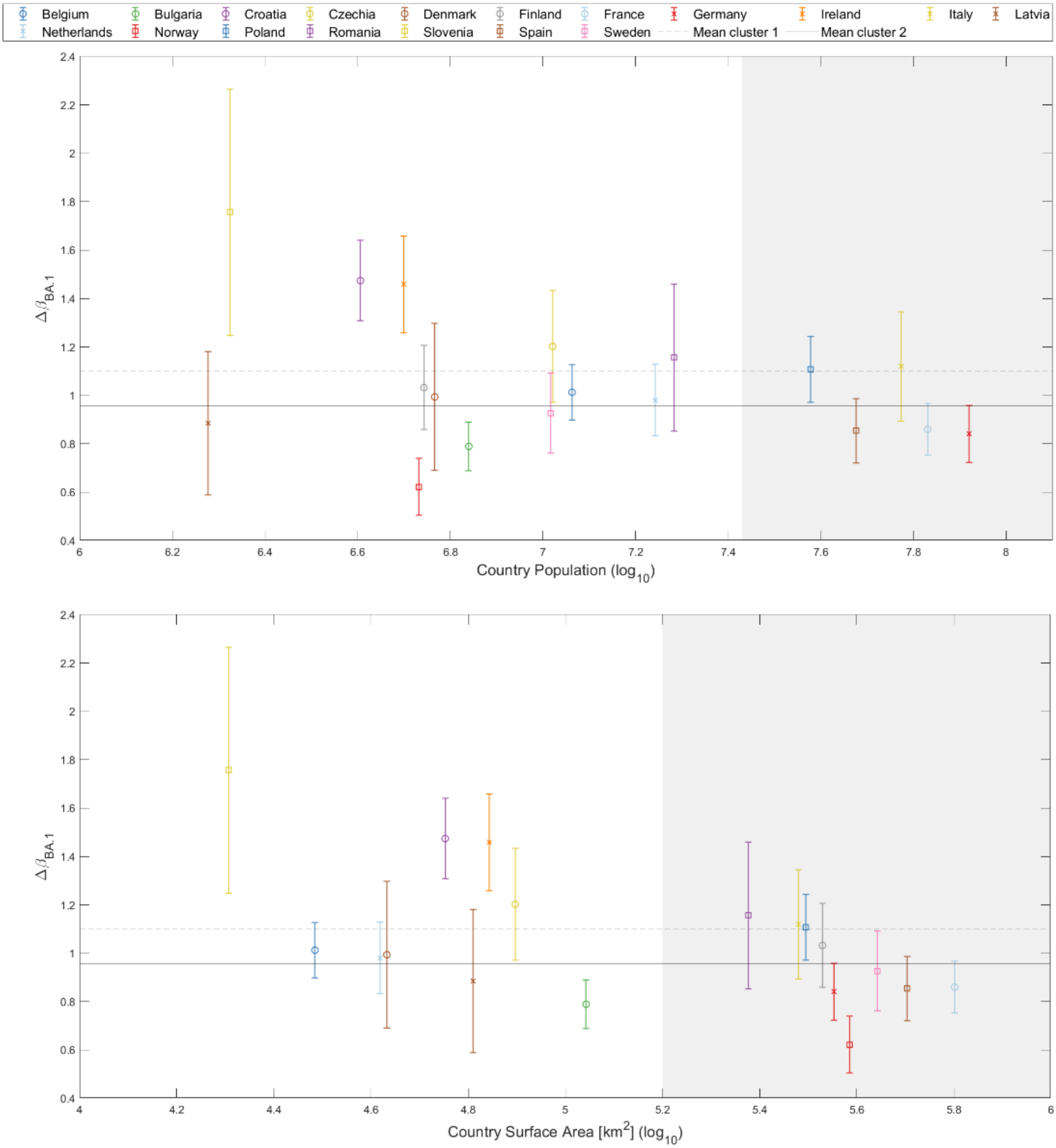

Figure S75 Increase in transmissibility ($\Delta\beta$) of the BA.1 variant plotted against: (Top) the logarithm of the country's population and (Bottom) the logarithm of the country's surface area. Both images are divided into two distinct clusters: smaller populations and smaller countries (cluster 1) on a white background, and larger populations and larger countries (cluster 2) on a gray background. The mean $\Delta\beta$ values for both clusters are depicted with horizontal lines (cluster 1: Dashed line; cluster 2: Solid line).

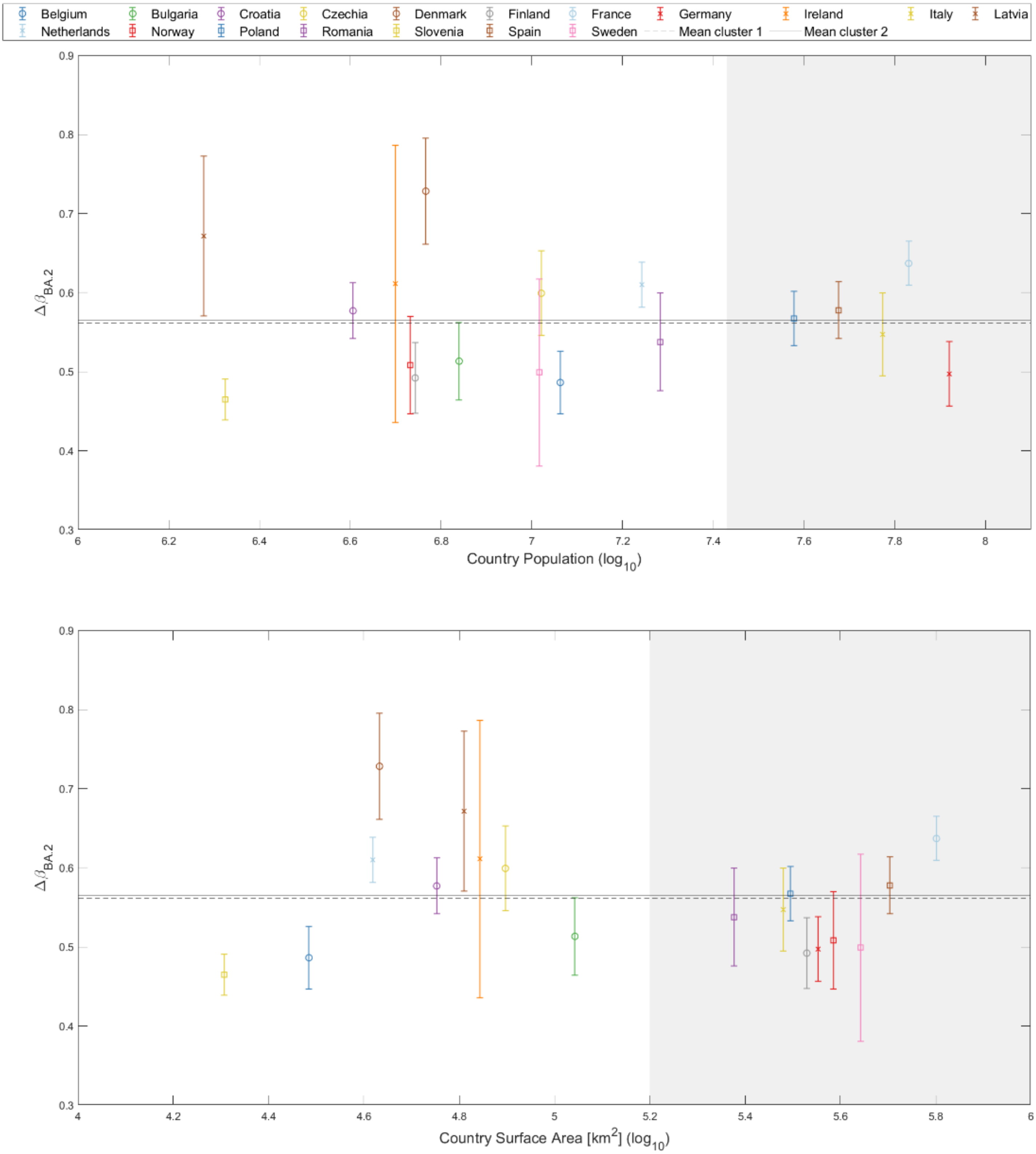

Figure S76 Increase in transmissibility ($\Delta\beta$) of the BA.2 variant plotted against: (Top) the logarithm of the country's population and (Bottom) the logarithm of the country's surface area. Both images are divided into two distinct clusters: smaller populations and smaller countries (cluster 1) on a white background, and larger populations and larger countries (cluster 2) on a gray background. The mean $\Delta\beta$ values for both clusters are depicted with horizontal lines (cluster 1: Dashed line; cluster 2: Solid line).

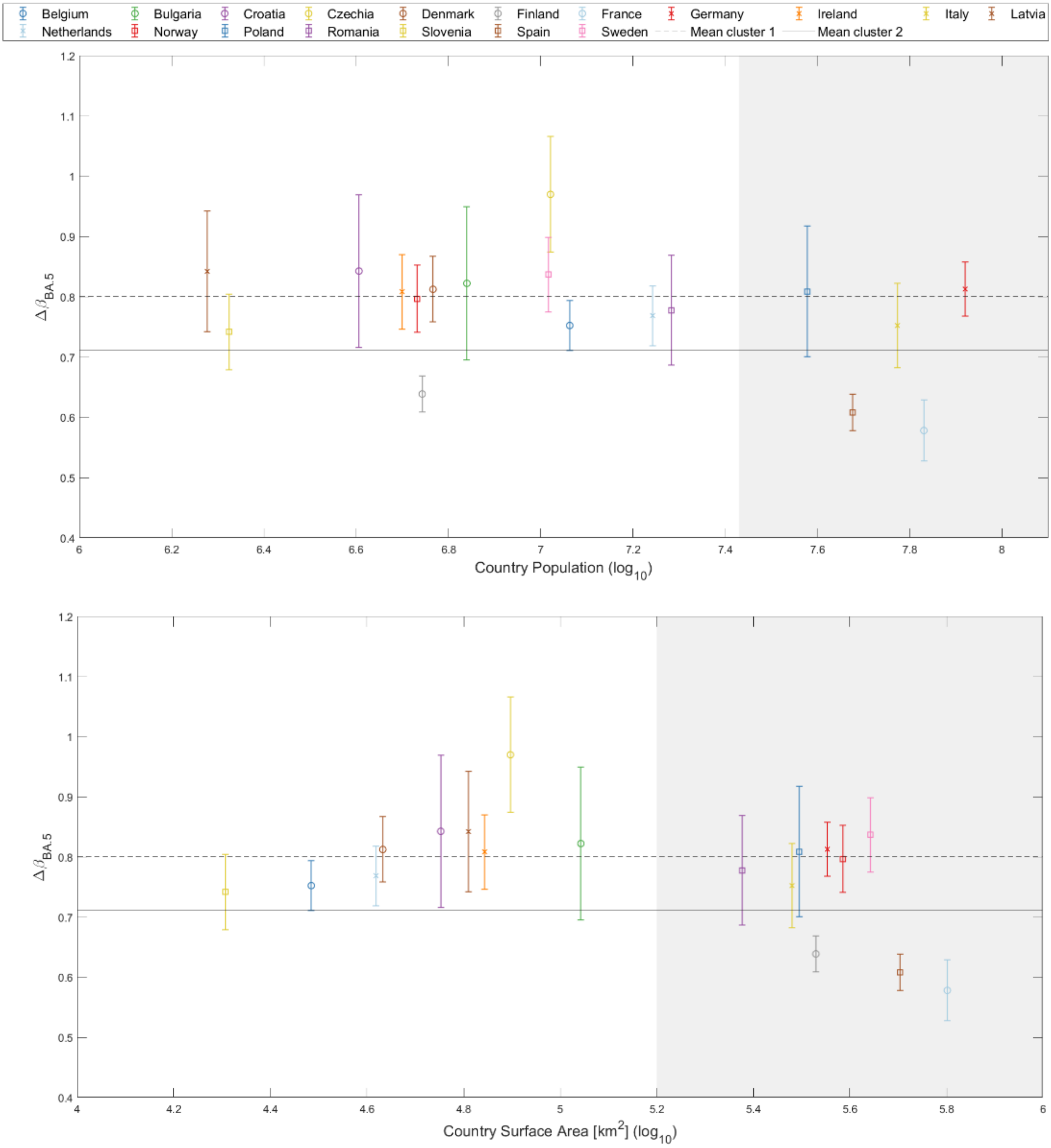

Figure S77 Increase in transmissibility ($\Delta\beta$) of the BA.5 variant plotted against: (Top) the logarithm of the country's population and (Bottom) the logarithm of the country's surface area. Both images are divided into two distinct clusters: smaller populations and smaller countries (cluster 1) on a white background, and larger populations and larger countries (cluster 2) on a gray background. The mean $\Delta\beta$ values for both clusters are depicted with horizontal lines (cluster 1: Dashed line; cluster 2: Solid line).

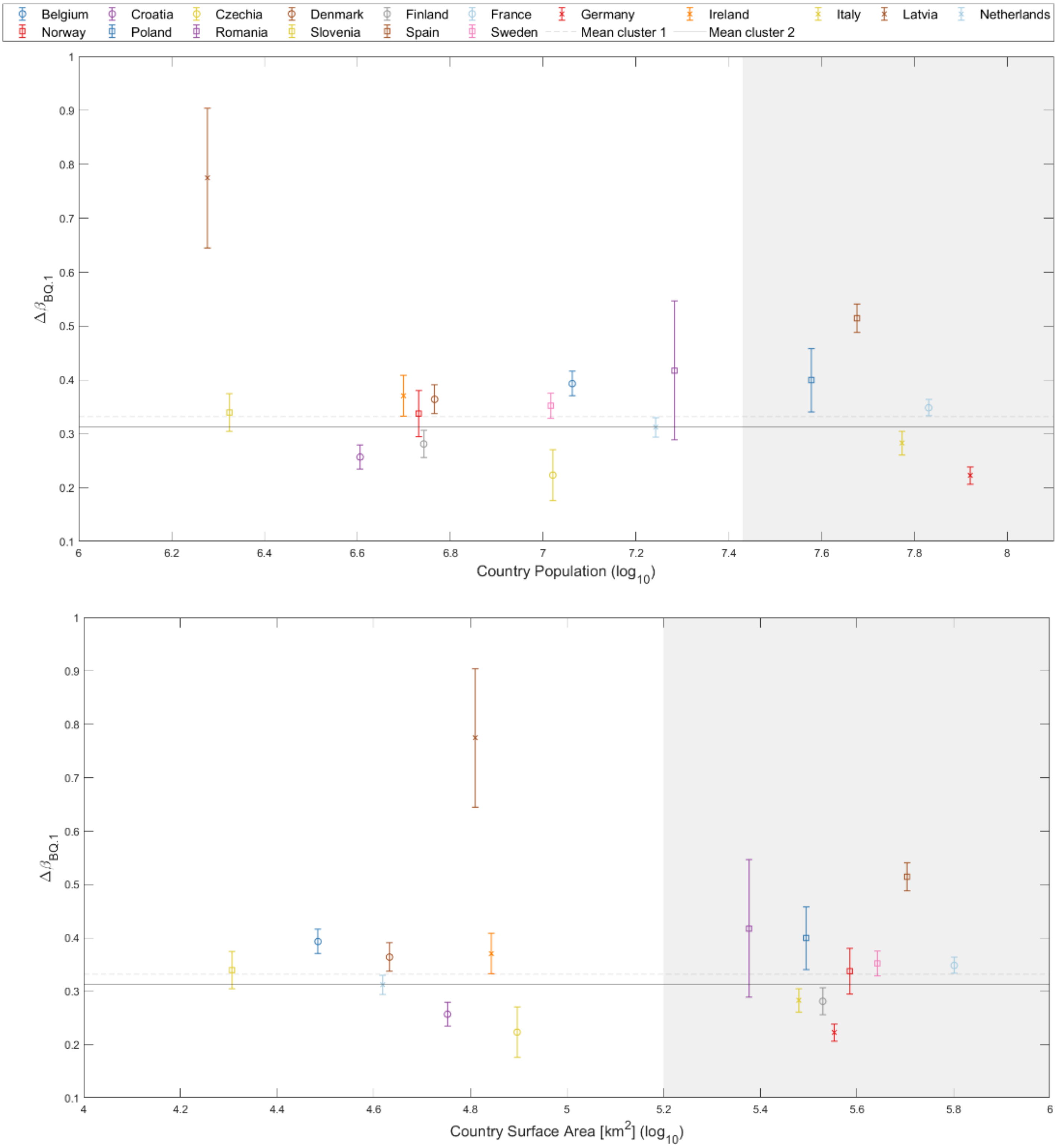

Figure S78 Increase in transmissibility ($\Delta\beta$) of the BQ.1 variant plotted against: (Top) the logarithm of the country's population and (Bottom) the logarithm of the country's surface area. Both images are divided into two distinct clusters: smaller populations and smaller countries (cluster 1) on a white background, and larger populations and larger countries (cluster 2) on a gray background. The mean $\Delta\beta$ values for both clusters are depicted with horizontal lines (cluster 1: Dashed line; cluster 2: Solid line).

Relation between Δβ values and population fully vaccinated

The following analysis involves studying the relationship between Δβ and the percentage of each country's population that is fully vaccinated, i.e., has completed the recommended vaccination regimen. This section serves to complement the findings presented in Section 3.2.4 of the main article, demonstrating, with the data from all six substitutions, the findings previously depicted in Figure 6 of the main article.

Figure S79 to Figure S84 present Δβ as a function of the percentage of each country's vaccinated population. Figure S80 is the same version presented in the main article, and Figures S78 and S80 are the same to Figure S64 (left and right, respectively).

One clear conclusion emerges from this analysis: only the arrival of the Delta variant showed any correlation with the population's vaccination level. More specifically, the higher the percentage of vaccinated individuals, the higher the transmissibility of this variant. As explained in Sections 3.2.4 and 4 (Discussion) of the main article, this arose because the vaccines restrained earlier variants, such as Alpha, but were less effective against newer lineages.

In the case of the Alpha variant's emergence, national vaccination policies were only just starting, making it impossible to draw any firm conclusions. As for the different Omicron variants, as can be observed in Figure S81 – Figure S84, the level of vaccination appears to have no discernible impact on the transmissibility of the variant.

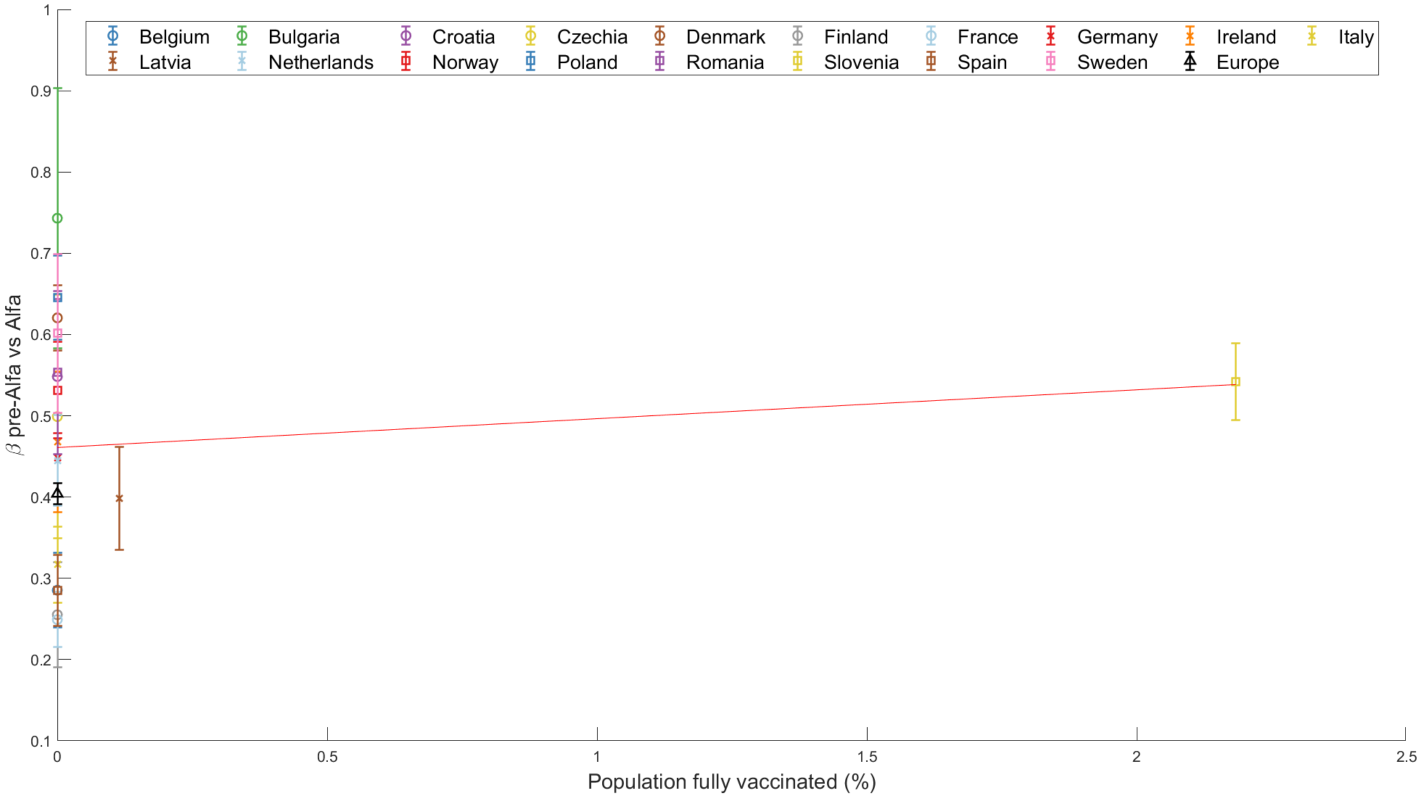

Figure S79 Relation between the increase in transmissibility ($\Delta\beta$) of SARS-CoV-2 Alpha variant and the percentage of fully vaccinated population in each country and Europe as a whole.

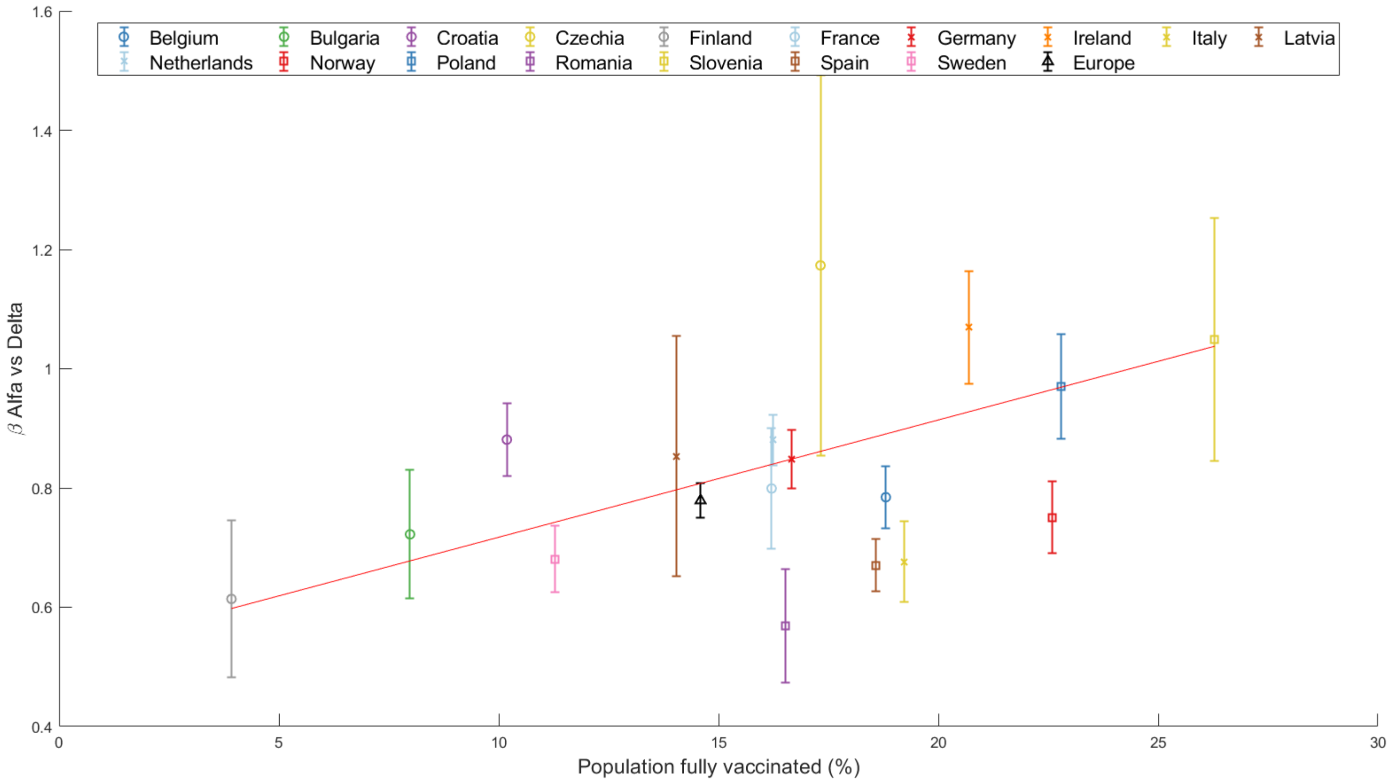

Figure S80 Relation between the increase in transmissibility ($\Delta\beta$) of SARS-CoV-2 Delta variant and the percentage of fully vaccinated population in each country and Europe as a whole. The data point for Denmark is excluded due to its significant deviation from the prior analysis, reducing its relevance.

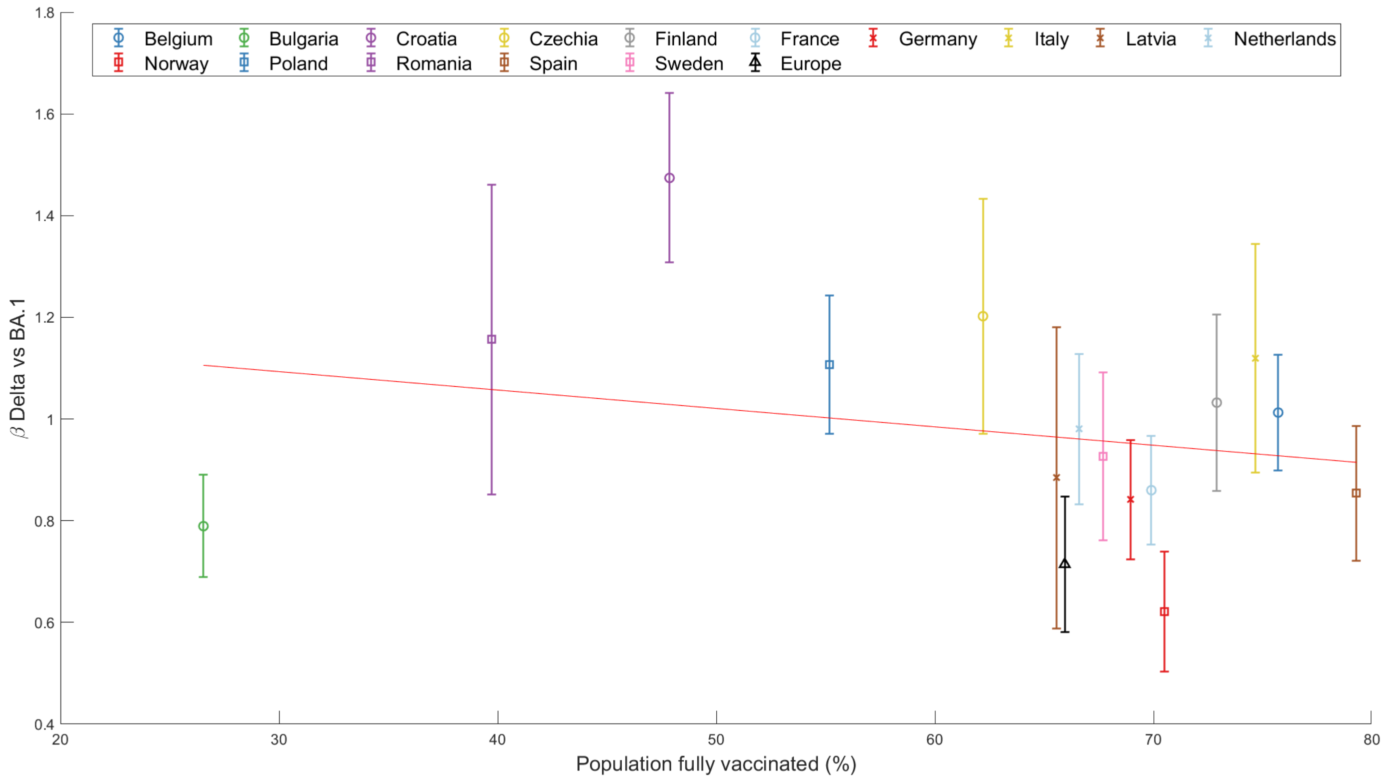

Figure S81 Relation between the increase in transmissibility ($\Delta\beta$) of SARS-CoV-2 BA.1 variant and the percentage of fully vaccinated population in each country and Europe as a whole. The data points for Ireland and Slovenia are excluded due to their significant deviation from the prior analysis.

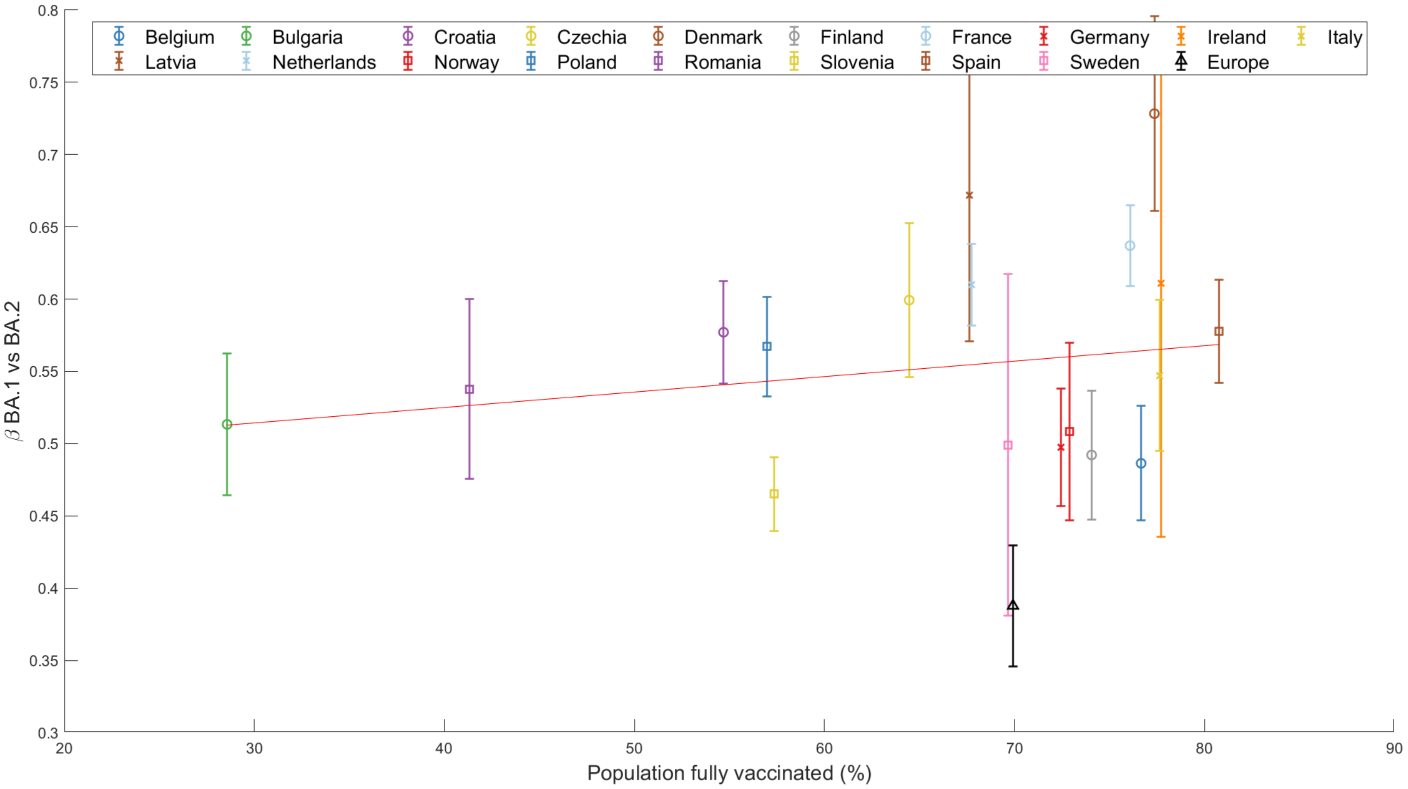

Figure S82 Relation between the increase in transmissibility ($\Delta\beta$) of SARS-CoV-2 BA.2 variant and the percentage of fully vaccinated population in each country and Europe as a whole.

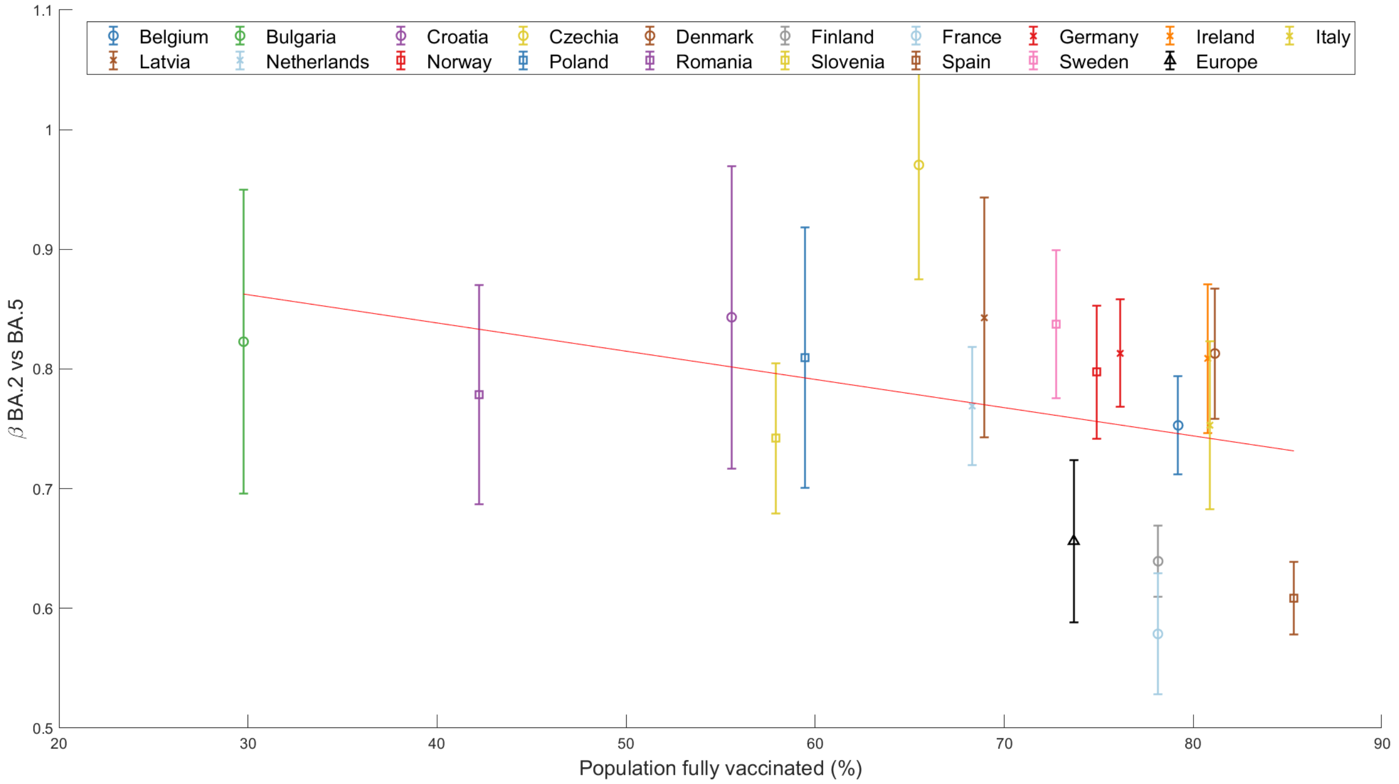

Figure S83 Relation between the increase in transmissibility ($\Delta\beta$) of SARS-CoV-2 BA.5 variant and the percentage of fully vaccinated population in each country and Europe as a whole.

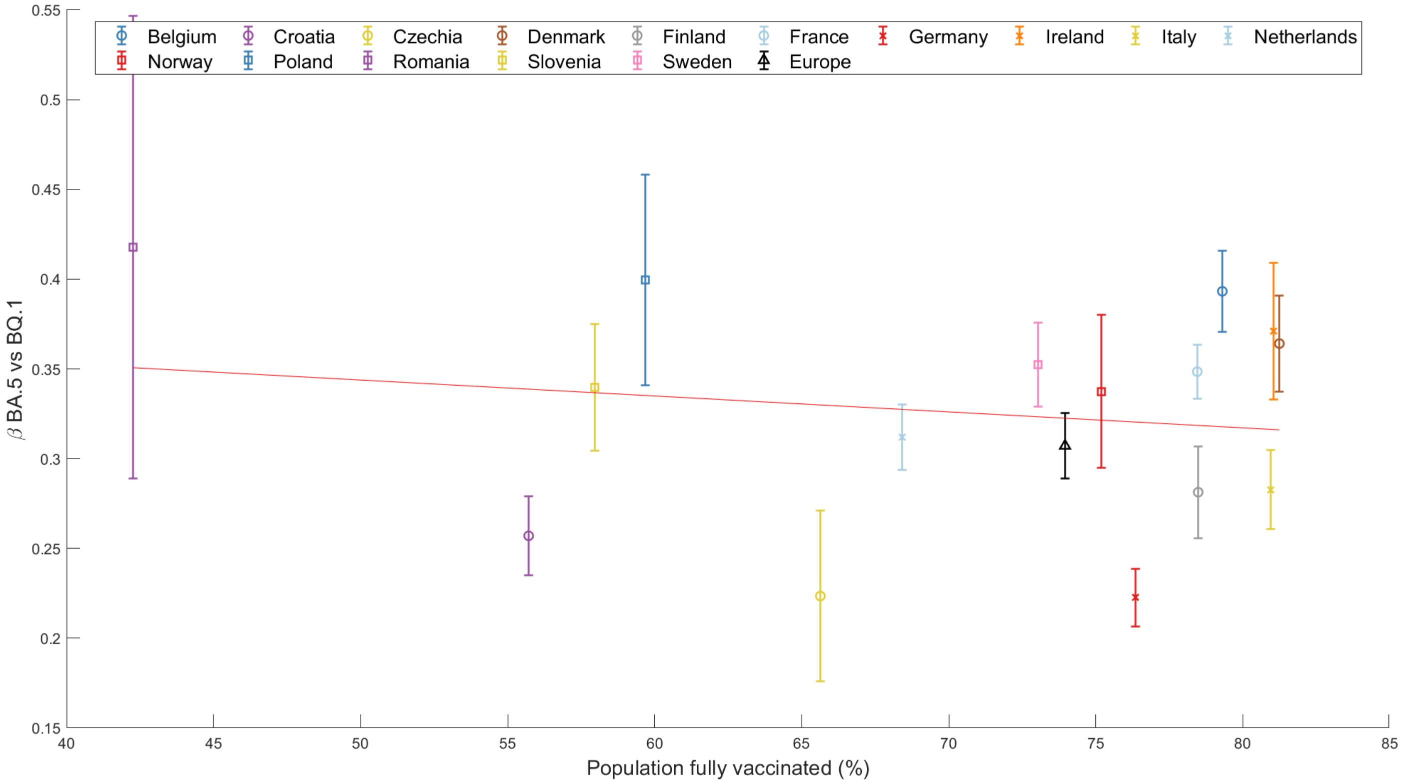

Figure S84 Relation between the increase in transmissibility ($\Delta\beta$) of SARS-CoV-2 BQ.1 variant and the percentage of fully vaccinated population in each country and Europe as a whole. The data points for Latvia and Spain are excluded due to their significant deviation from the prior analysis.

Evolution of Rt during variant substitutions

The effective reproduction number Rt is a critical metric for monitoring the progression of a pandemic, representing the average number of people that a single infected individual might infect at a particular point in time. The emergence of new variants could potentially trigger a surge in infections, given that the population could have developed immunity to the presently dominant variant, yet remain susceptible to the novel strain.

In the main paper's Section 3.2.5, we demonstrated the relationship between the Rt at the commencement and termination of each of the three major variant substitutions: pre-Alpha vs Alpha; Alpha vs Delta; and Delta vs Omicron (Figure 7)

In this supplementary material, we revisit the substitutions of the various Omicron variants. Unlike the main paper, we have not obtained a figure for the last substitution. Specifically, the prevalence of the BQ.1 variant often does not reach 60% of the total cases in many countries due to its intense competition with other variants, such as XBB.1.5 or the BA.2-like variants. Consequently, the following figures, Figures S84-S88, pertain only to the 18 countries (excluding Lithuania, as noted in this Suppl. Material Text S10) and five substitutions (Alpha, Delta, BA.1, BA.2, and BA.5).

In all the figures, the dashed diagonal line represents the 1/1 boundary, which differentiates whether one Rt value is larger than the other. As per our earlier discussion, we anticipate that most data points will fall above this line. The major findings are:

- Once again, the first substitution (pre-Alpha vs Alpha) has some points below the line (Figure S85). Recall that this substitution occurred during a period of low variant sampling and followed the 2020-2021 holiday season, which was marked by relatively light restrictions and high case numbers prior to the Alpha variant's emergence.
- The Delta, BA.1, and BA.5 substitutions align with expectations, with barely a single country per substitution falling below the line (Figure S86, Figure S87, and Figure S89).
- The BA.2 substitution is notable (Figure S88), with a number of countries below the line equal to that in the initial substitution. To understand this, we need only reference the country figures in the Suppl. Material Text S6 section. The wave of infections triggered by the emergence of the first major Omicron strain (BA.1) dwarfed all others in case numbers. The BA.2 variant appeared weeks to a few months later, depending on the country. Hence, it is not unusual for countries with a short interval between BA.1 and BA.2 to have a higher initial number of cases than at the end of the BA.1 vs BA.2 substitution, thus leading to an Rt < 1.
-

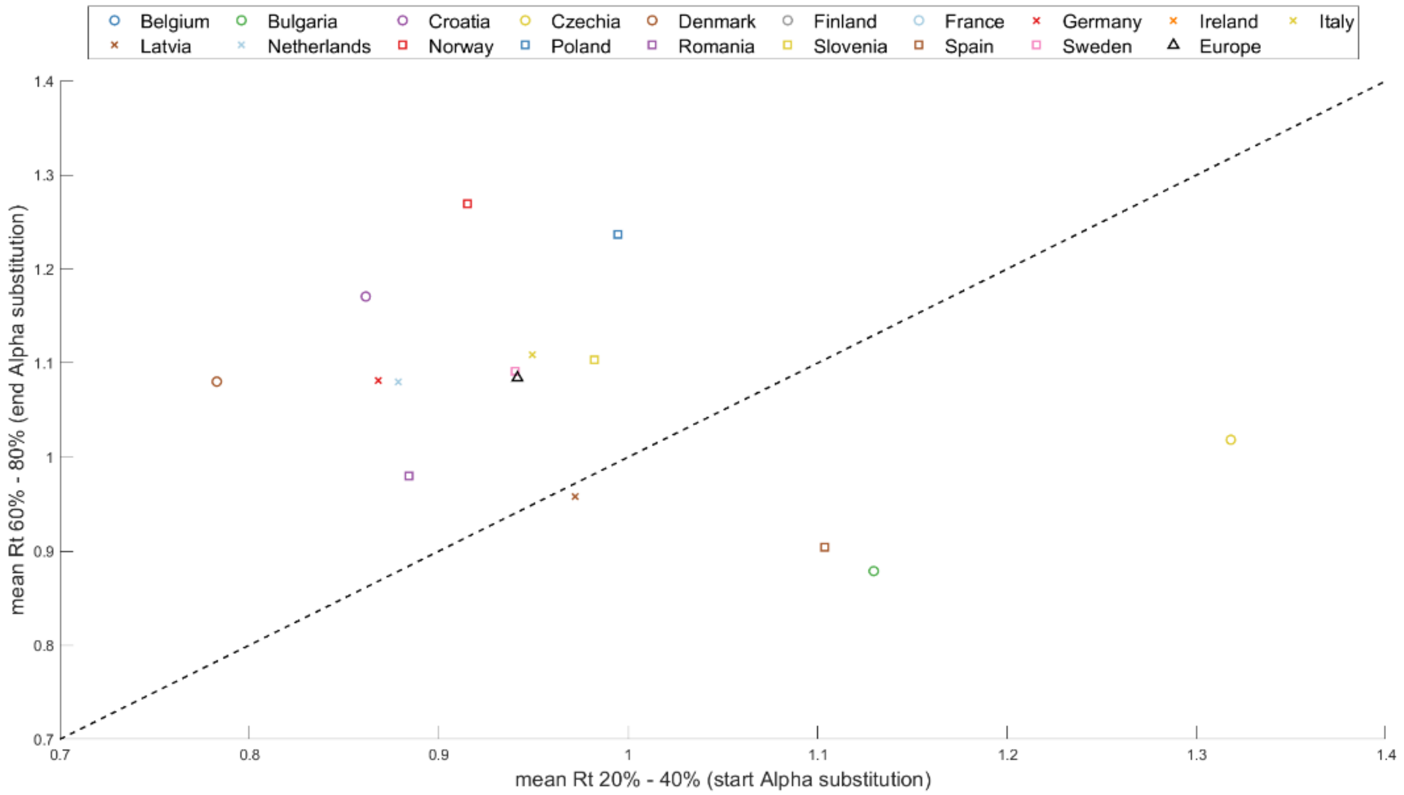

Figure S85 Mean effective reproduction number (Rt) during the pre-Alpha to Alpha substitution. The plot compares Rt at the beginning and end of substitution (corresponding to when Alpha makes up 20%-40% and 60%-80% of total cases, respectively).

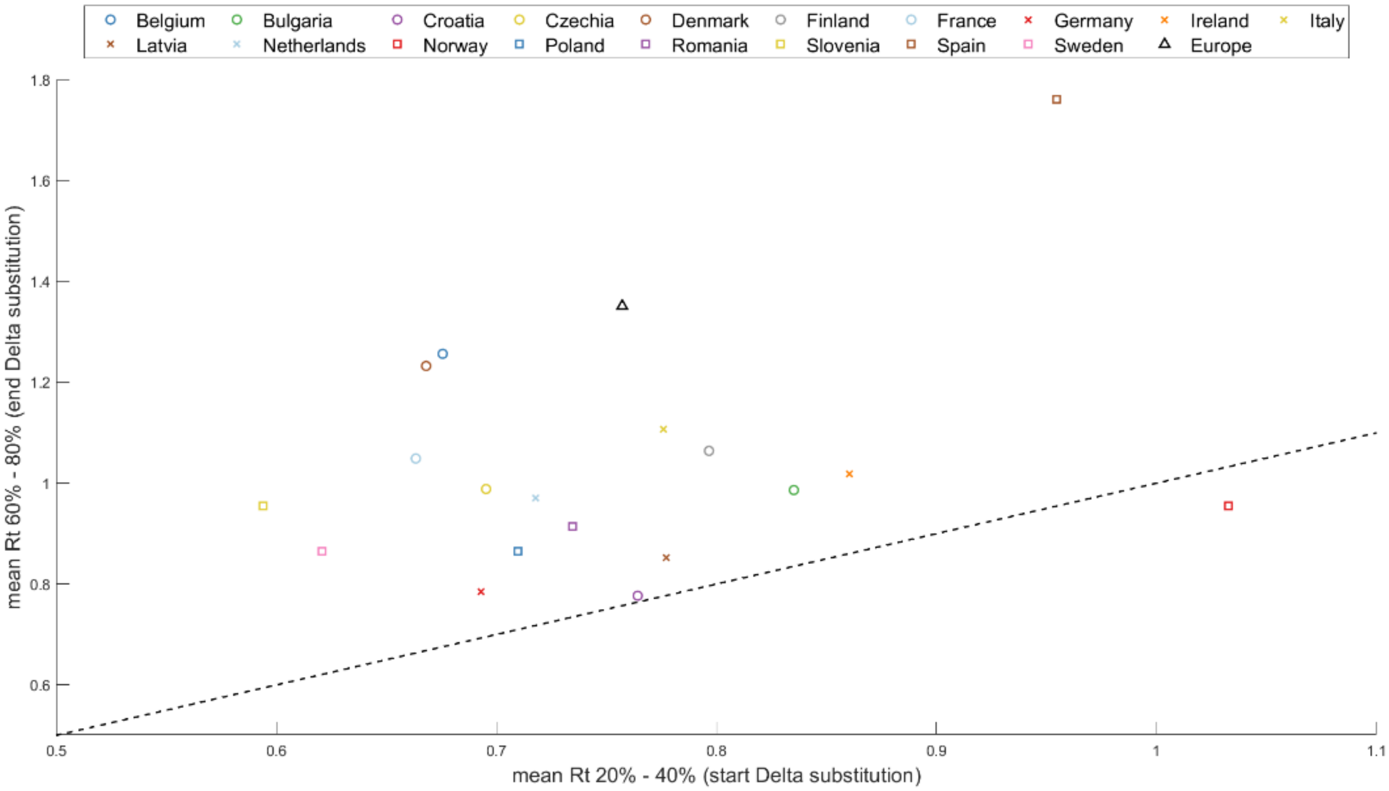

Figure S86 Mean effective reproduction number (Rt) during the Alpha to Delta substitution. The Rt values are shown at the beginning and end of substitution (representing when Delta forms 20%-40% and 60%-80% of total cases, respectively).

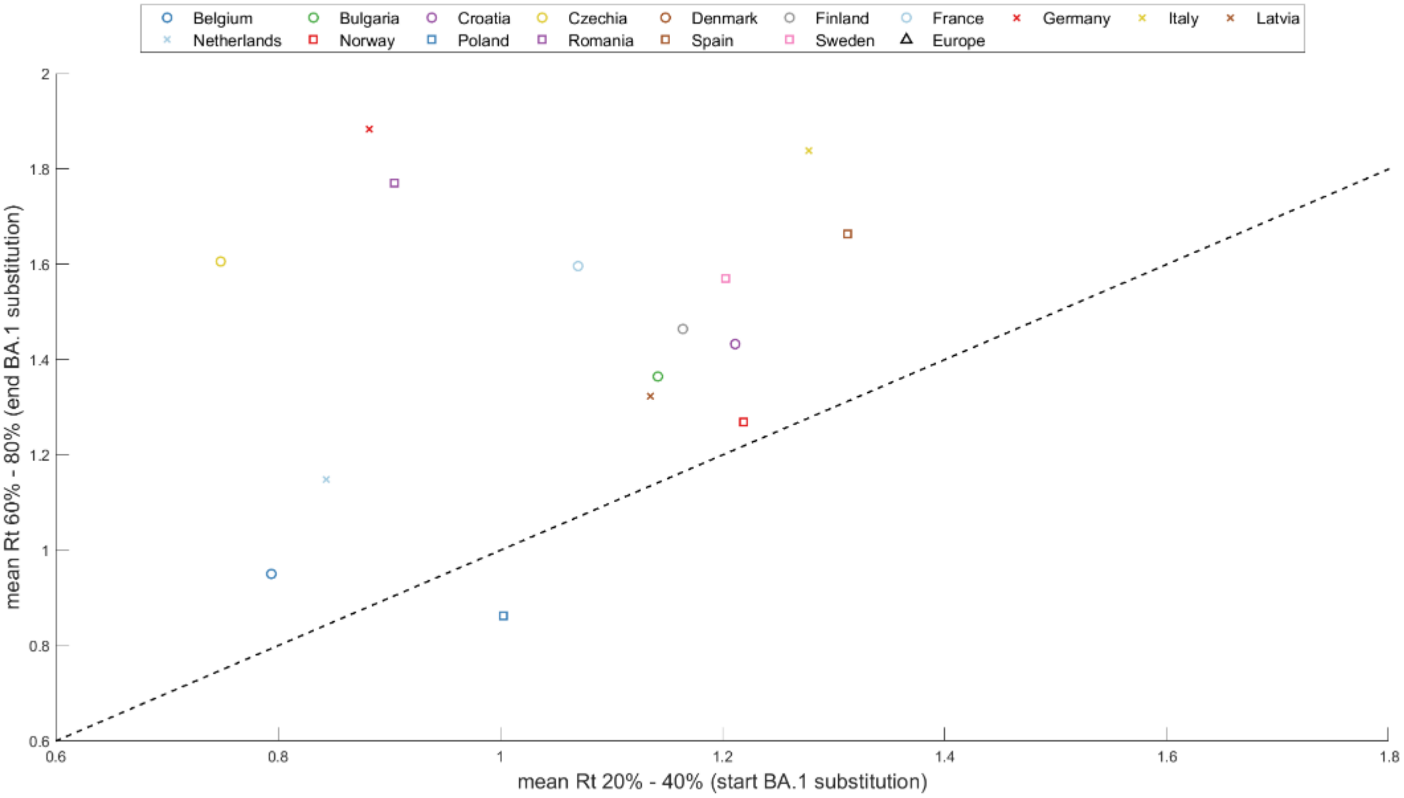

Figure S87 Mean effective reproduction number (Rt) during the Delta to BA.1 substitution. The plot contrasts Rt at the start and conclusion of substitution (corresponding to when BA.1 accounts for 20%-40% and 60%-80% of total cases, respectively).

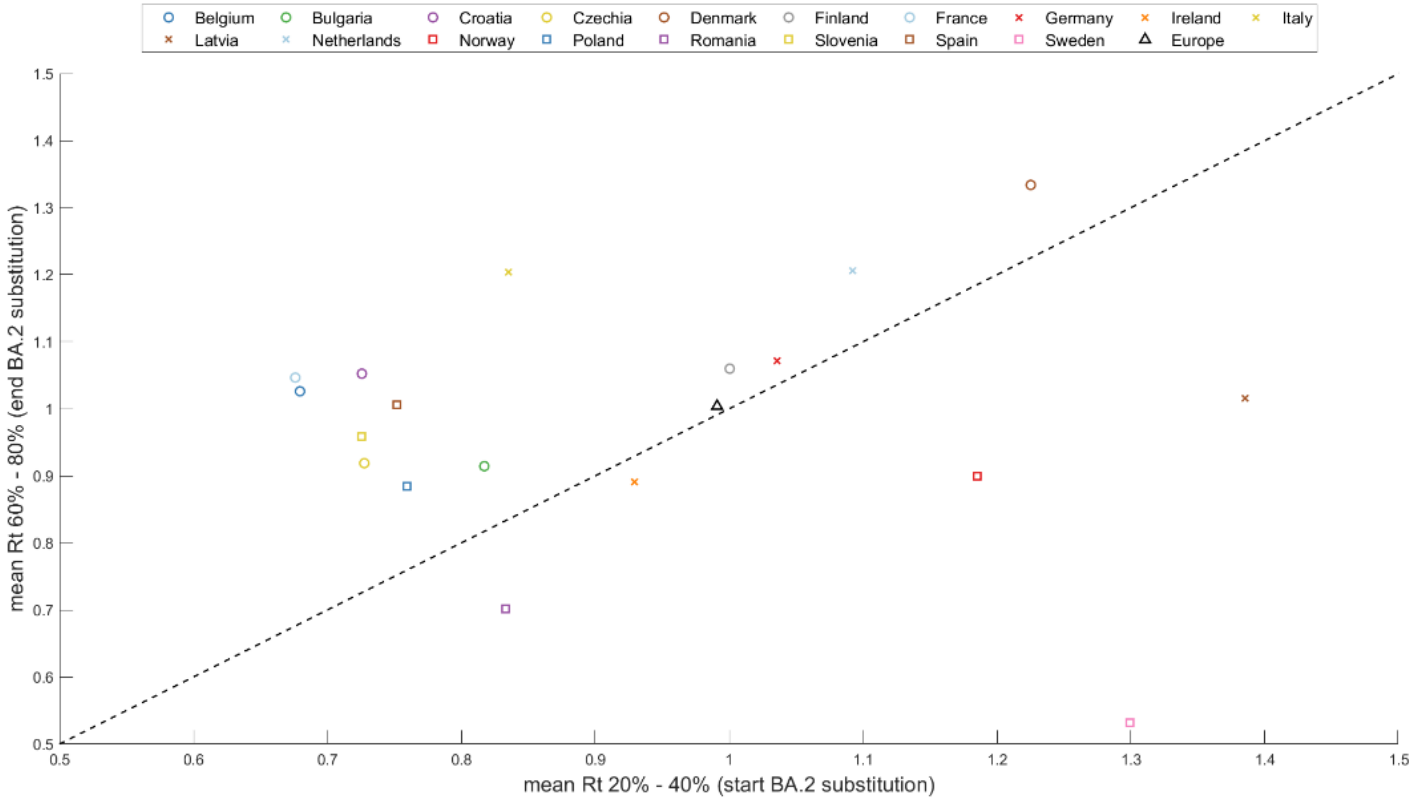

Figure S88 Mean effective reproduction number (Rt) during the BA.1 to BA.2 substitution. The plot compares Rt at the beginning and end of substitution (corresponding to when BA.2 makes up 20%-40% and 60%-80% of total cases, respectively).

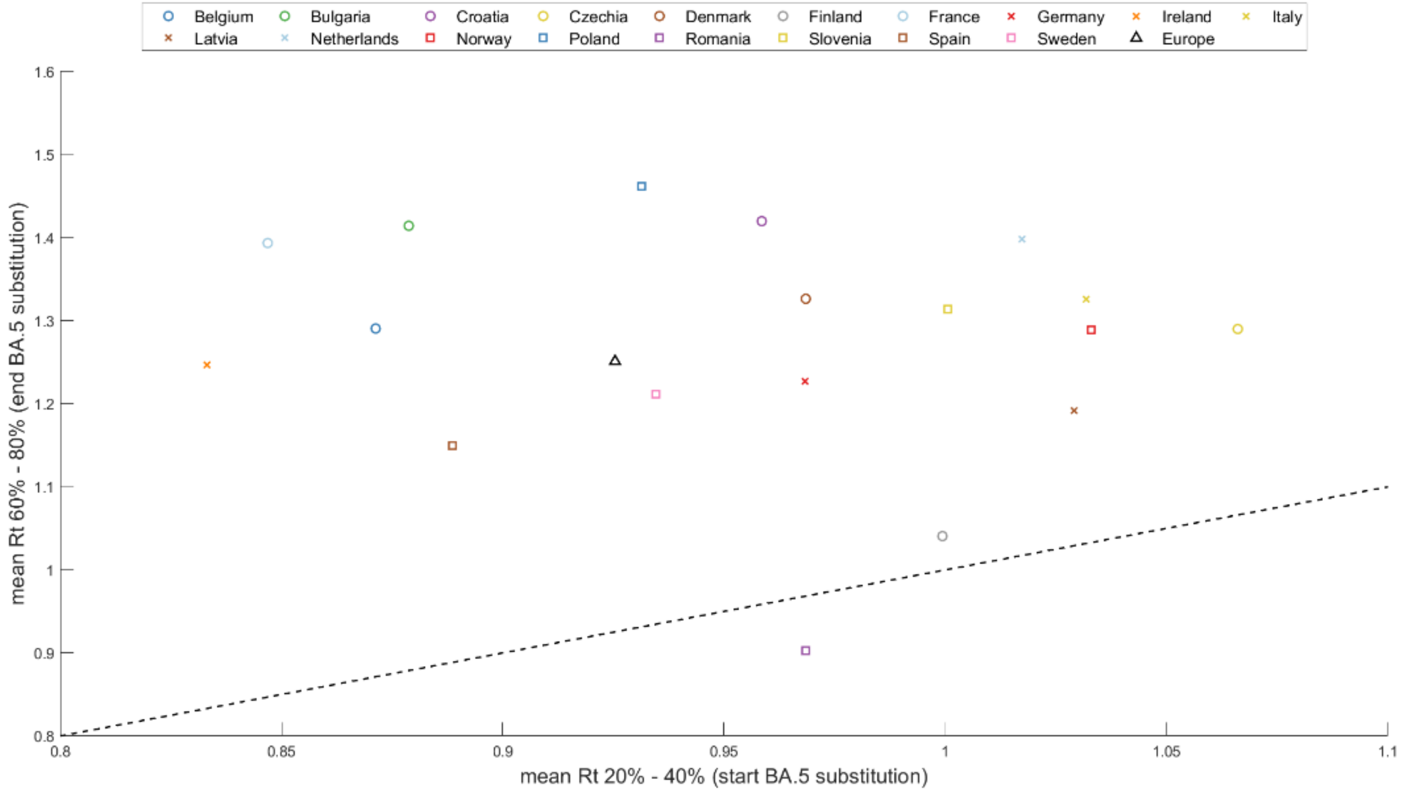

Figure S89 Mean effective reproduction number (Rt) during the BA.2 to BA.5 substitution. The Rt values are shown at the beginning and end of substitution (representing when BA.5 forms 20%-40% and 60%-80% of total cases, respectively).

Suppl. Material Text S11 – Assessing independence of GISAID and TESSy through transmissibility differences

The results of the main article present, besides the figures of Spain with the number of cases sequenced per week, the estimated and calculated percentages of each variant and their respective incidences in the number of cases, various plots comparing the increment in transmissibility Δβ with respect to different countries, different variant substitutions and different factors that could have caused this parameter to vary from one place to another. Figures 3 – 7 in the results section 3.2 show these different studies for the three major substitutions (Alpha, Delta, and Omicron). All these results have been calculated from the data in the GISAID database which, as extensively discussed in section 2.1 of the article and in the Suppl. Mat. Text S1 and S2, does not contain a homogeneous number of samples and varies greatly from country to country. This is due, in part, to another database: TESSy. TESSy contains the same type of information and, unfortunately, with the same variability. As we have noted, it is possible that in some countries and at sometimes these two databases overlap, but as seen in Suppl. Material Text S1, it is very likely that in some countries and at other times this is not the case.

In this section, we want to study what would have happened if these two databases, GISAID and TESSy, were totally independent. To do this, we will proceed exactly as we have done in the article, with the same codes, the same approaches, and the same studies of Δβ with respect to different factors.

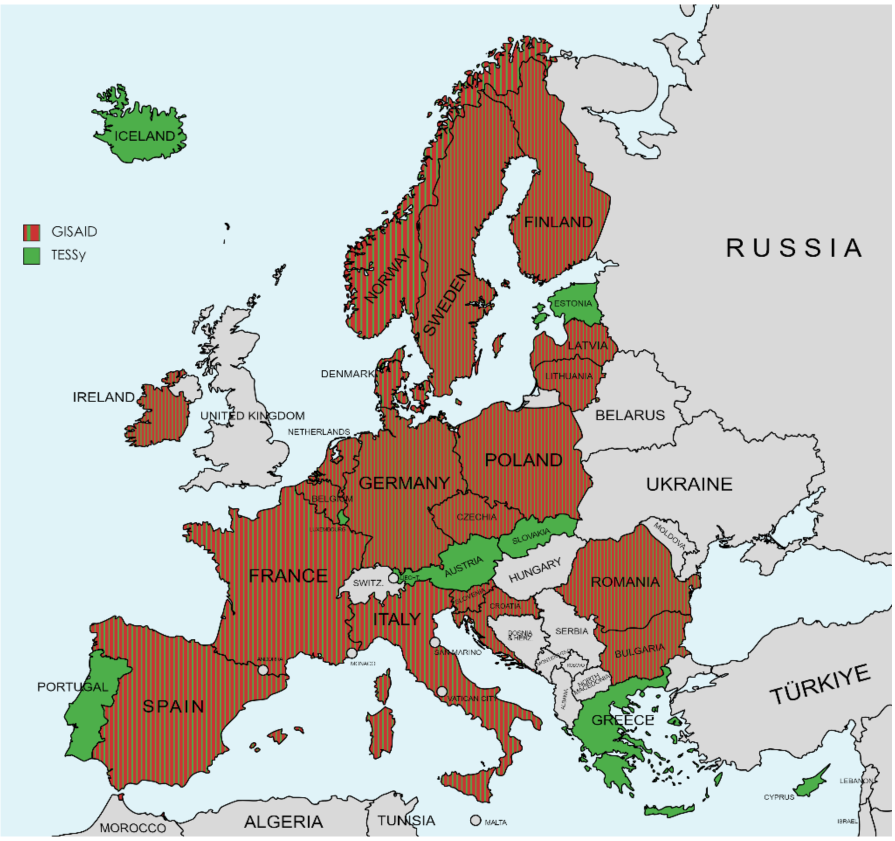

Figure S90 Europe map highlighting countries by database: red for GISAID, green for GISAID+TESSy.

Using the two databases together allows us to study a larger set of countries. If before, with GISAID, the report of variants and cases allowed us to study 19 countries in the three major substitutions (Alpha, Delta, and Omicron), now we can do the same with up to 27 countries. Of the 19 countries we had studied in the main article: Belgium, Bulgaria, Croatia, Czechia, Denmark, Finland, France, Germany, Ireland, Italy, Latvia, Lithuania, Netherlands, Norway, Poland, Romania, Slovenia, Spain, and Sweden; now added are: Austria, Cyprus, Estonia, Greece, Iceland, Luxembourg, Portugal, and Slovakia. Figure S89 shows in red the countries studied in the main article and in all previous supplementary materials and, in green, those intended to be studied in this last section considering GISAID and TESSy as independent.

Studying the temporal evolution of the different virus variants with these two databases results in very similar outcomes as in the figures of the article. Obviously, by merging the databases, the number of weekly sequences will be higher and, as it changes, the weekly percentage of each variant changes, and therefore also the mathematical model of substitution. Figure S90 shows the case of Spain, as we have done in the main article (Figure 2), to see that, although the number of samples increases (the plot at the top), the percentage and the result of the model are very similar, only slightly varying. The only clear difference we can observe between Figure S90, and Figure 2 is that, on the sample scale, which in this figure (GISAID+TESSy) reaches 7000 samples/week and in Figure 2 (GISAID) barely reaches 4000 samples/week.

This general image already shows us that the results for $\Delta\beta$ and its different analyses according to different factors will also be very similar to those already shown in the article.

Once the simulations for all substitutions and countries have been performed, we proceed to calculate and display the same results obtained in the main article, but with the current 27 countries and Europe.

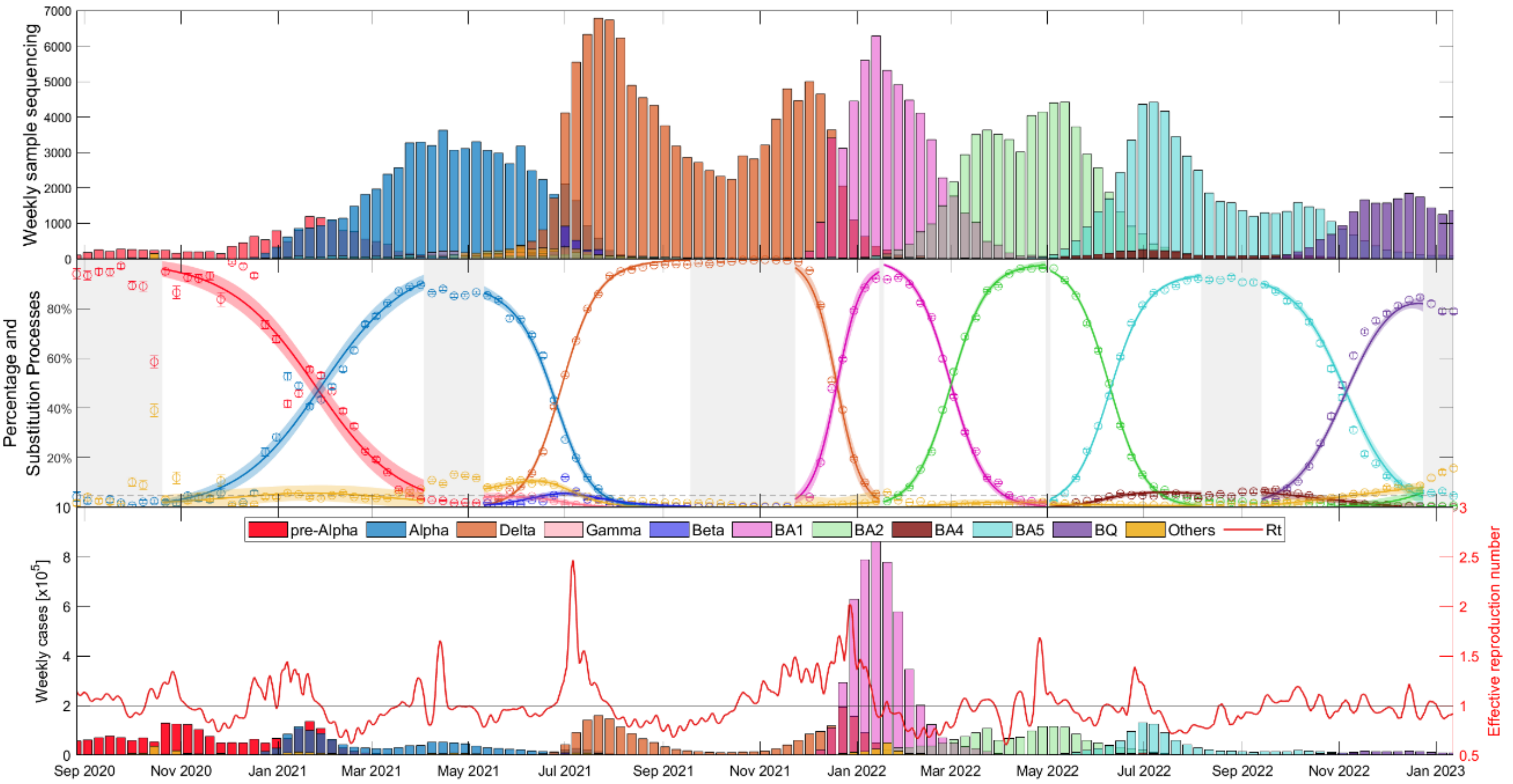

Figure S91 Evolutionary dynamics of Alpha, Delta, and Omicron lineages (BA.1, BA.2, BA.5, and BQ.1) substitutions in Spain over time for GISAID+TESSy database sources. The figure is a direct comparison with Figure 2, calculated only with GISAID source.

Figure S91, analogous to Figure 3 of the article, represents the increase in transmissibility parameter in the three major substitutions for the countries analyzed with both databases. Again, even when adding more countries, the trend is exactly the same: $\Delta\beta_{\mathrm{Alpha}} < \Delta\beta_{\mathrm{Delta}} < \Delta\beta_{\mathrm{Omicron}}$.

Thanks to the boxplots calculated with the 27 countries with their corresponding weights (from their error bars), we can extract the average (horizontal red line within the rectangle) and see which countries are outside of what is statistically acceptable. In this case, as in the main article, Denmark and Lithuania have values that are too high for $\Delta\beta$ for the second substitution shown, and, in this case, Iceland has a slightly higher value of $\Delta\beta$ in the last substitution (in addition to huge error bars).

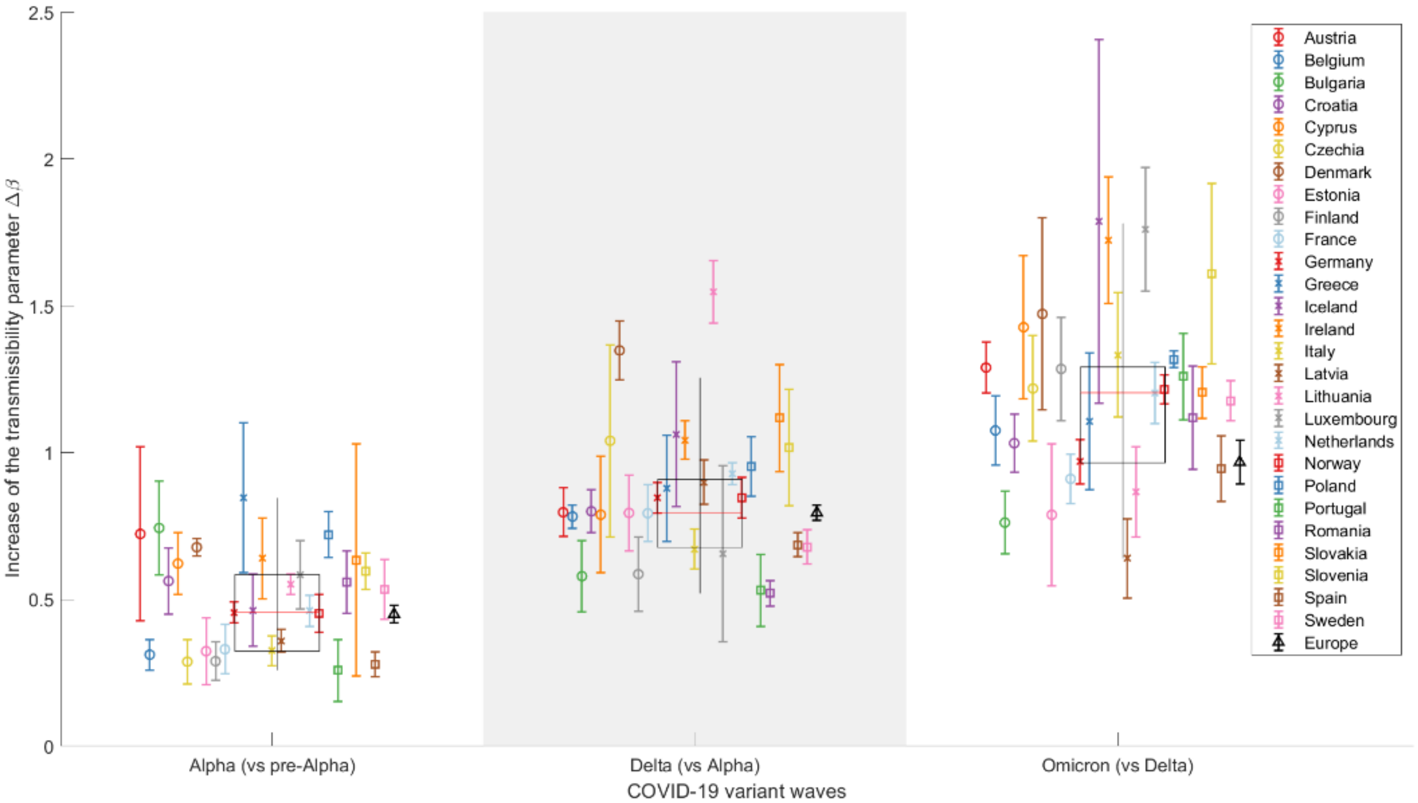

Figure S92 Representation of the increase in transmissibility ($\Delta\beta$) for 27 European countries (circles, crosses, and squares) and Europe (black triangle) as a combination of the studied countries, based on the results for the Alpha, Delta, and Omicron variants with GISAID and TESSy database sources. The x-axis simply distinguishes the three COVID-19 variant substitutions, while the y-axis displays the increment on the transmissibility parameter for each country.

As can be seen in the figure, we again achieve the same trend of $\Delta\beta_{Europe} < \Delta\beta_{Average}$, just as we saw in the article and as happened when we considered all six substitutions, see Suppl. Mat. Text S10 (Figure S65 and Table S1). Table S3 shows the results achieved in the main article with the GISAID database and the values achieved with the union of the two databases. In both cases it is fulfilled that Δβ tends to decrease when the country (or in this case, the continent) is too large and, therefore, the global computation of new variants over time broadens, because the different outbreaks are occurring in very different spaces and times.

Table S3 Results for Europe and for the average of all countries. Las dos primeras filas se corresponden con la Tabla 1 del artículo. Las dos últimas filas son los valores que podemos observar en la Figura S91.

	**Alpha**	**Delta**	**Omicron**
**Europe (GISAID)**	0.4041	0.7821	0.9114
**Average (GISAID)**	0.4477	0.7863	1.177
**Europe (GISAID+TESSy)**	0.4492	0.7952	0.9675
**Average (GISAID+TESSy)**	0.4551	0.7957	1.204

Figure S93 to Figure S95 present Δβ as a function of the day of entry of the new variant in each country, similar to Figure 4 of the main article. The first two substitutions (pre-

Alpha vs Alpha and Alpha vs Delta) display similar trends and values, indicating that the later the new variant is introduced, the greater the increase in transmissibility. For the last depicted substitution (Omicron), the trend qualitatively persists, although it loses statistical significance (see Table S4). Nonetheless, the conclusions remain unchanged: transmissibility tends to be higher the later the new variant is introduced, suggesting that initial outbreaks are more localized.

Table S4 Statistical results for trend analysis across three lineage transitions for GISAID (Table 2) and GISAID+TESSy data sources.

	**Alpha**	**Delta**	**Omicron**
$\rho_{5\%}$ **(GISAID)**	0.609	0.804	0.523
p-value **(GISAID)**	0.005	$\sim 10^{-5}$	0.022
R^2 **(GISAID)**	0.6	0.67	0.5
$\rho_{5\%}$ **(GISAID+TESSy)**	0.431	0.734	0.155
p-value **(GISAID+TESSy)**	0.022	$\sim 10^{-5}$	0.459
R^2 **(GISAID+TESSy)**	0.4	0.3	0.08

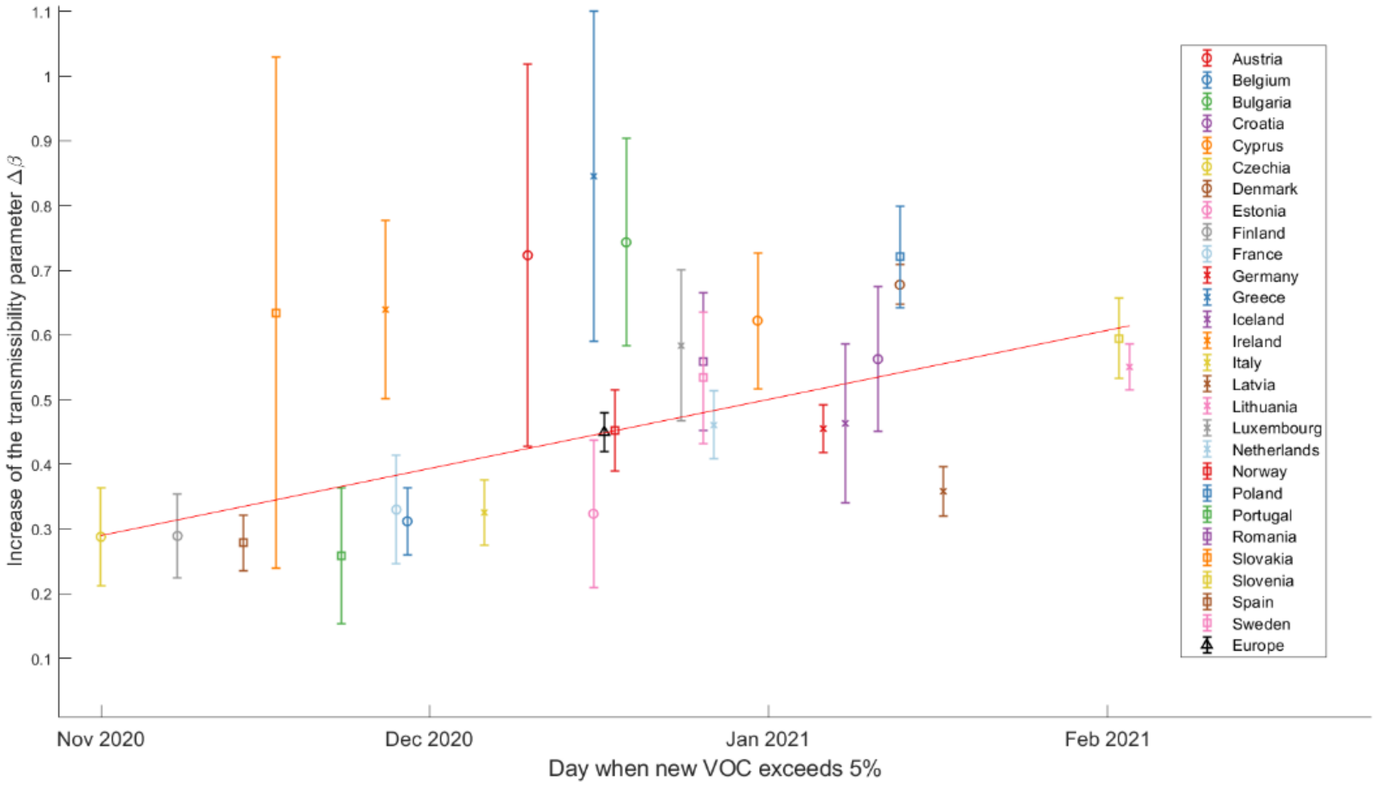

Figure S93 The increase in transmissibility ($\Delta\beta$) based on the day the emerging variant Alpha exceeded 5% and GISAID+TESSy databases sources according to our substitution model.

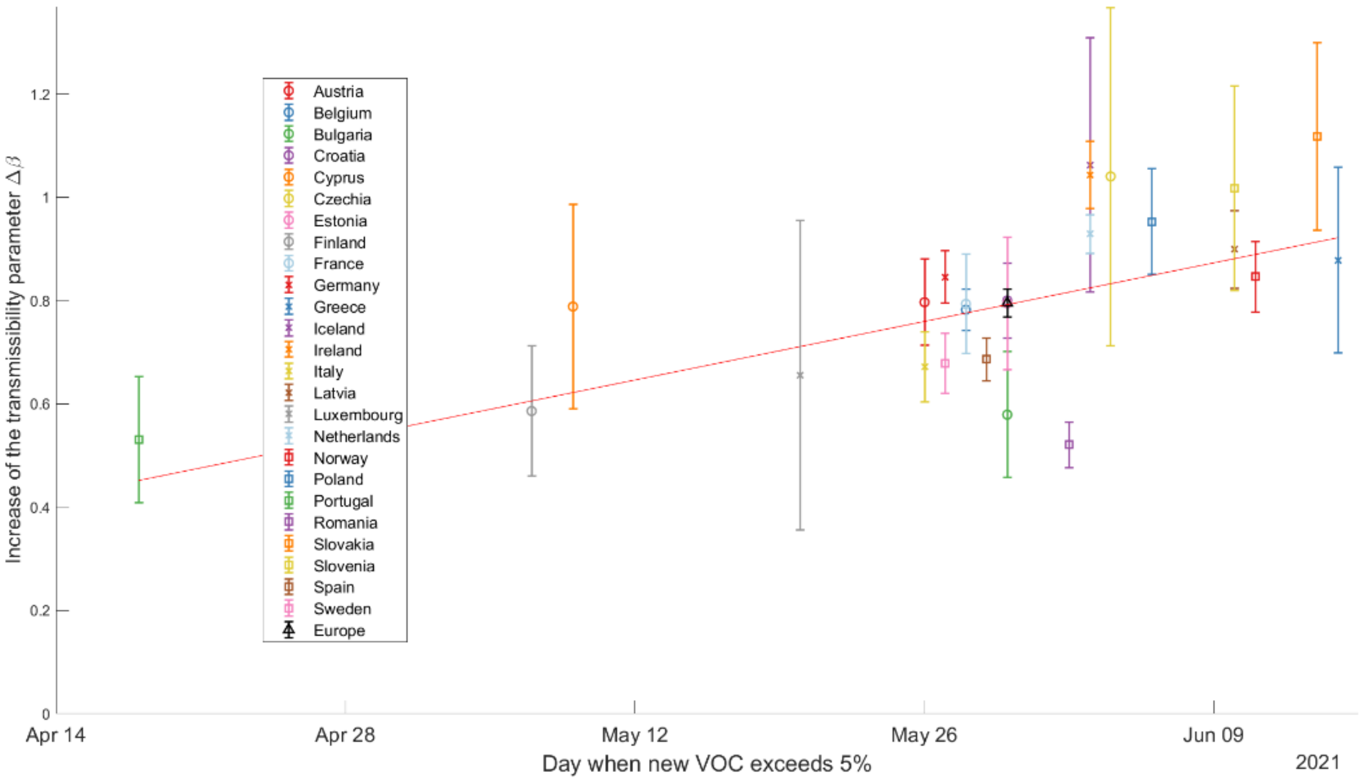

Figure S94 The increase in transmissibility ($\Delta\beta$) based on the day the emerging variant Delta exceeded 5% and GISAID+TESSy database sources according to our substitution model.

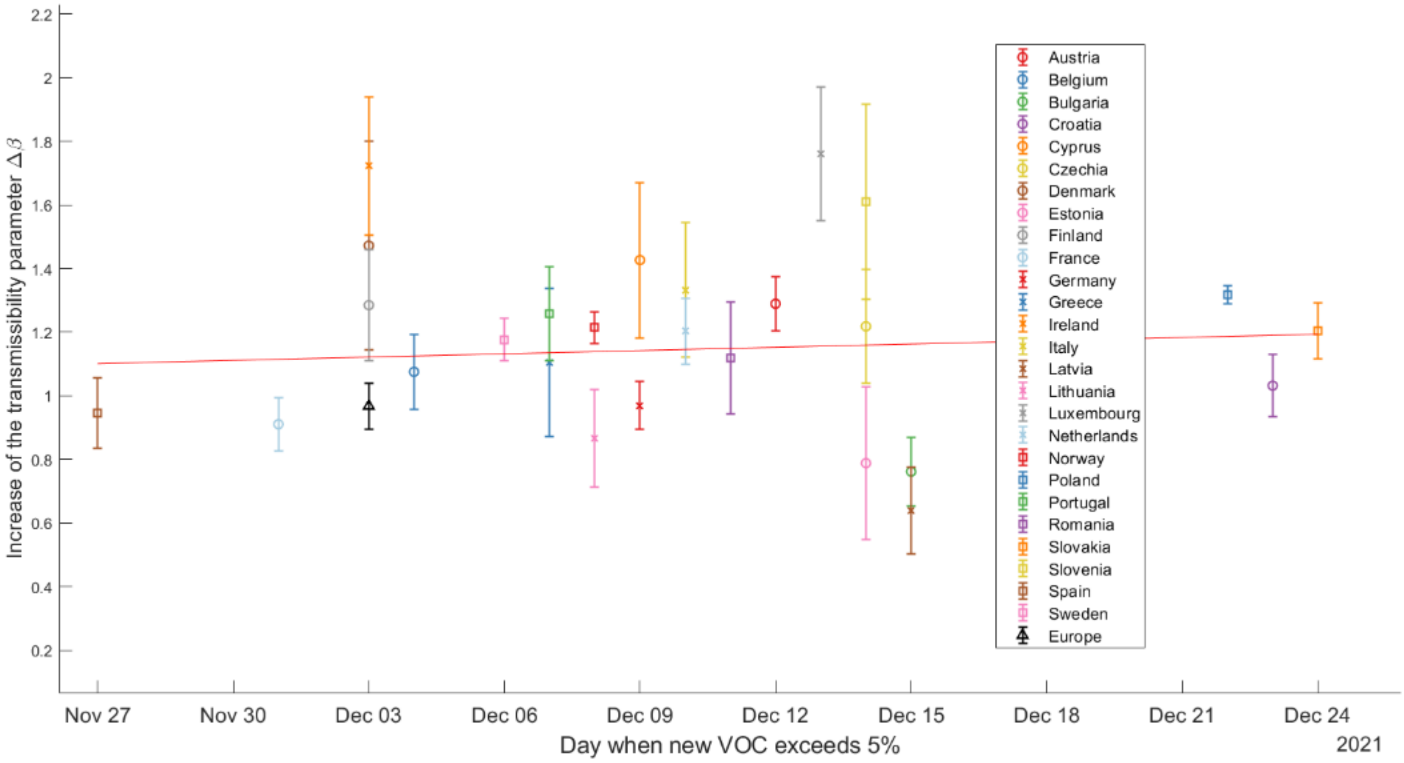

Figure S95 The increase in transmissibility ($\Delta\beta$) based on the day the emerging variant Omicron exceeded 5% and GISAID+TESSy database sources according to our substitution model.

Subsequently, Figure S96 to Figure S98 illustrate the relationship between Δβ and country size, analogous to Figure 5 of the main article. These figures split the countries into two major clusters:

- Cluster 2, larger countries: Romania, Italy, Poland, Finland, Germany, Norway, Sweden, Spain, and France.
- Cluster 1, smaller countries: Greece, Bulgaria, Iceland, Portugal, Austria, Czech Republic, Ireland, Lithuania, Latvia, Croatia, Slovakia, Estonia, Denmark, Netherlands, Belgium, Slovenia, Cyprus, and Luxembourg.

The two horizontal lines represent the mean, with a solid line for larger countries (Cluster 2) and a dashed line for smaller countries (Cluster 1). Again, it is found that larger countries tend to have a smaller increase in the transmissibility of new variants.

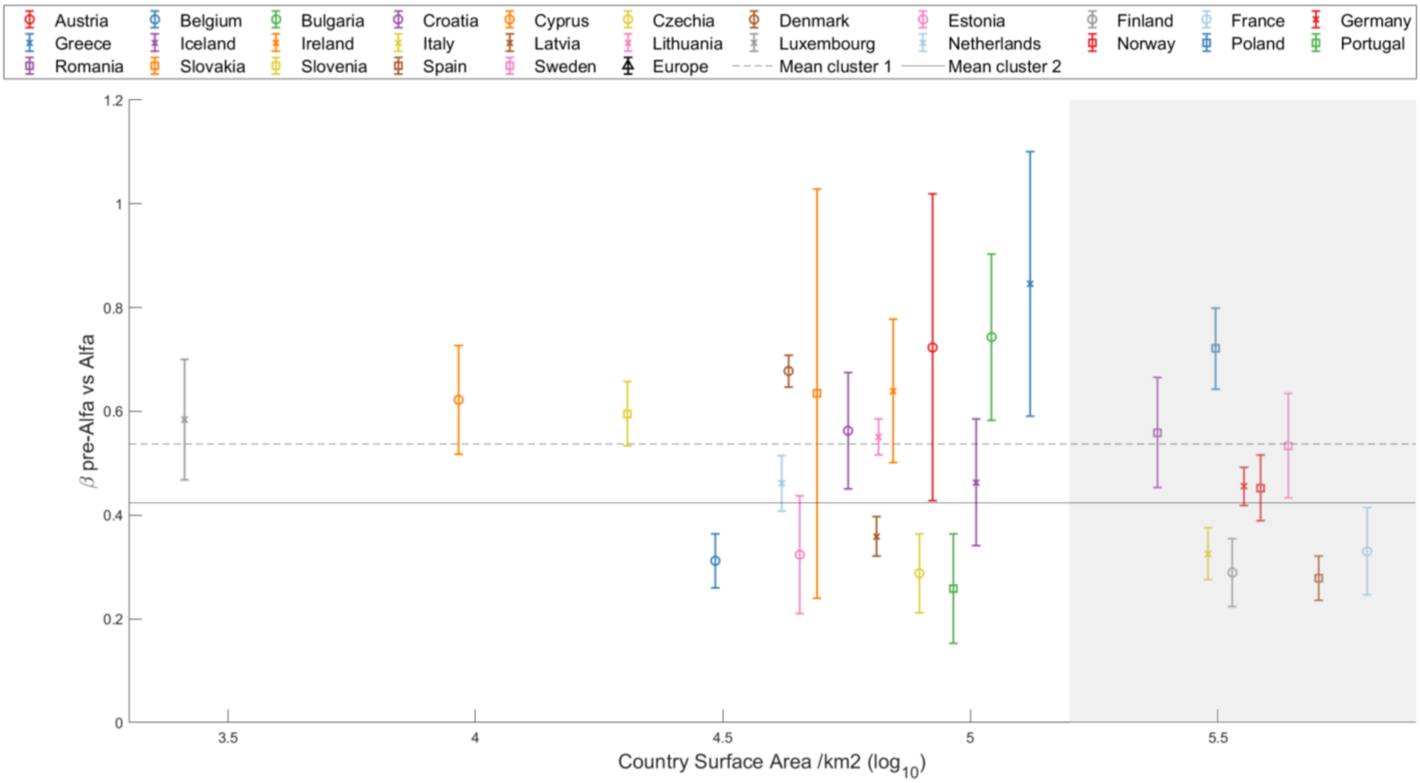

Figure S96 Increase of transmissibility for the Alpha substitution ($\Delta\beta$) plotted against the logarithm of the country's surface area, distinguishing two primary clusters: smaller countries (cluster 1) on a white background, and larger countries (cluster 2) on a gray background. Mean $\Delta\beta$ for both clusters are depicted in each substitution with horizontal lines (cluster 1: Dashed line; cluster 2: Solid line).

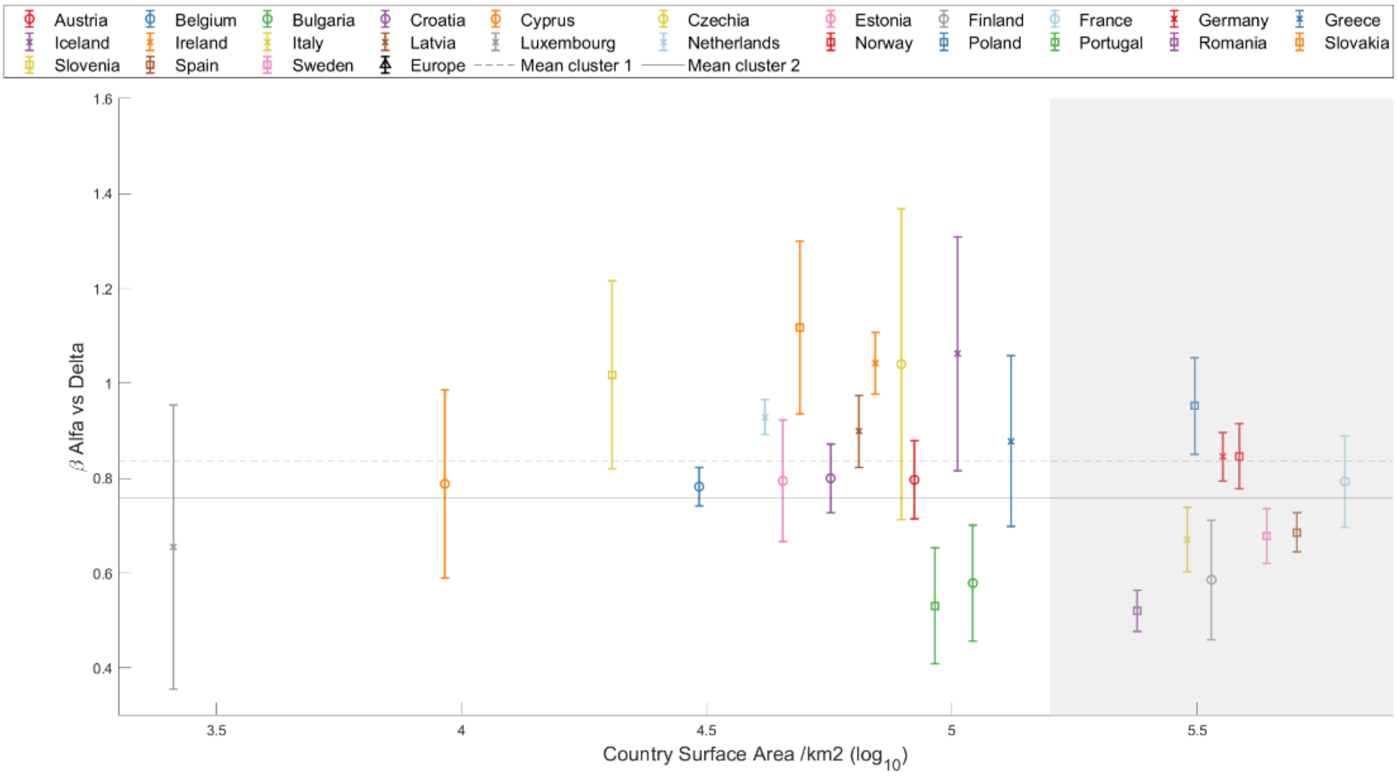

Figure S97 Increase of transmissibility for the Delta substitution (Δβ) plotted against the logarithm of the country's surface area, distinguishing two primary clusters: smaller countries (cluster 1) on a white background, and larger countries (cluster 2) on a gray background. Mean Δβ for both clusters are depicted in each substitution with horizontal lines (cluster 1: Dashed line; cluster 2: Solid line).

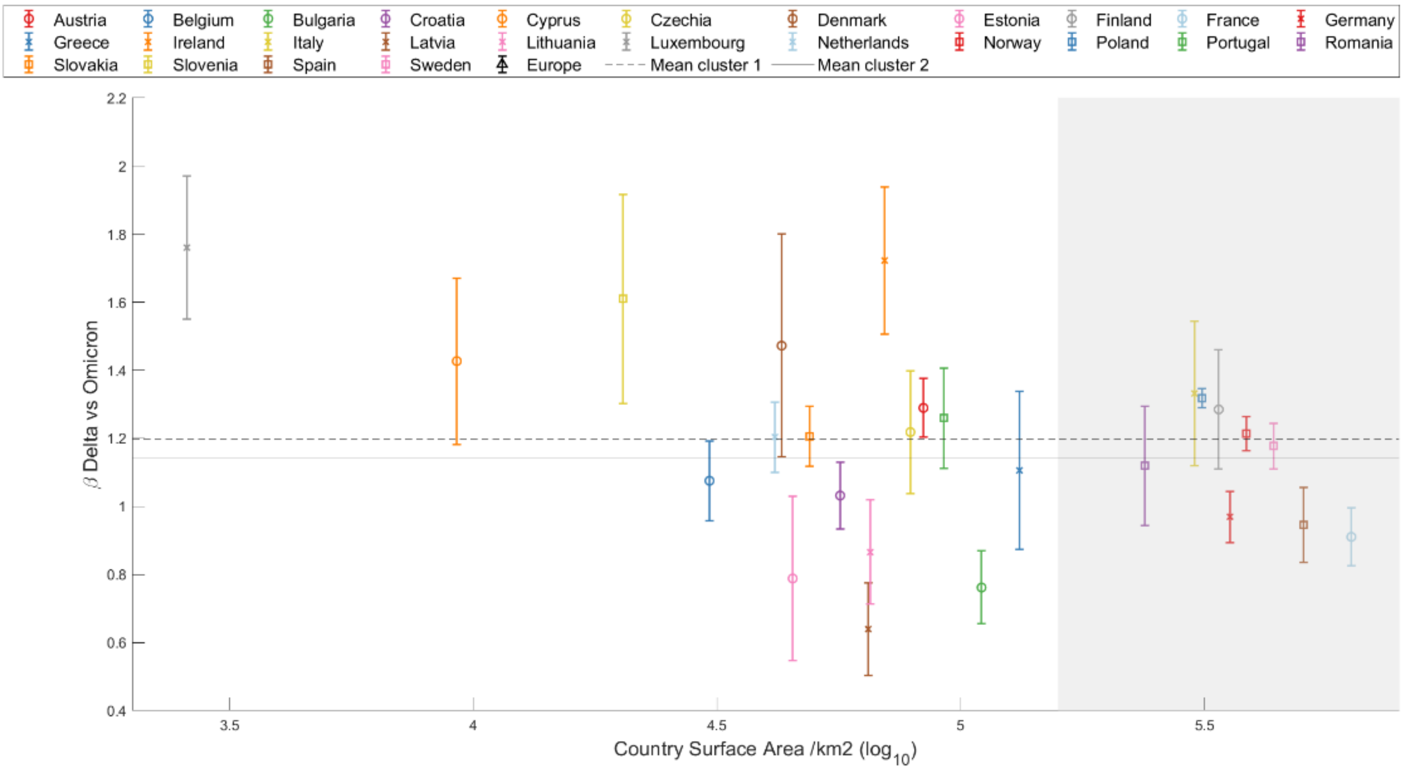

Figure S98 Increase of transmissibility for the Omicron substitution ($\Delta\beta$) plotted against the logarithm of the country's surface area, distinguishing two primary clusters: smaller countries (cluster 1) on a white background, and larger countries (cluster 2) on a gray background. Mean $\Delta\beta$ for both clusters are depicted in each substitution with horizontal lines (cluster 1: Dashed line; cluster 2: Solid line).

To proceed as in the main article, the next result displays the dependency of Δβ on the vaccination status of the country's population. Again, Figure S99 illustrates the substitution of the Alpha variant by the Delta variant: the correlation between the level of fully vaccinated individuals and the increase in transmissibility is clear.

The substitution of Alpha is not shown since when this variant emerged, the vast majority of countries had not yet started their vaccination policies. In the case of the Omicron variant, any type of relationship is lost.

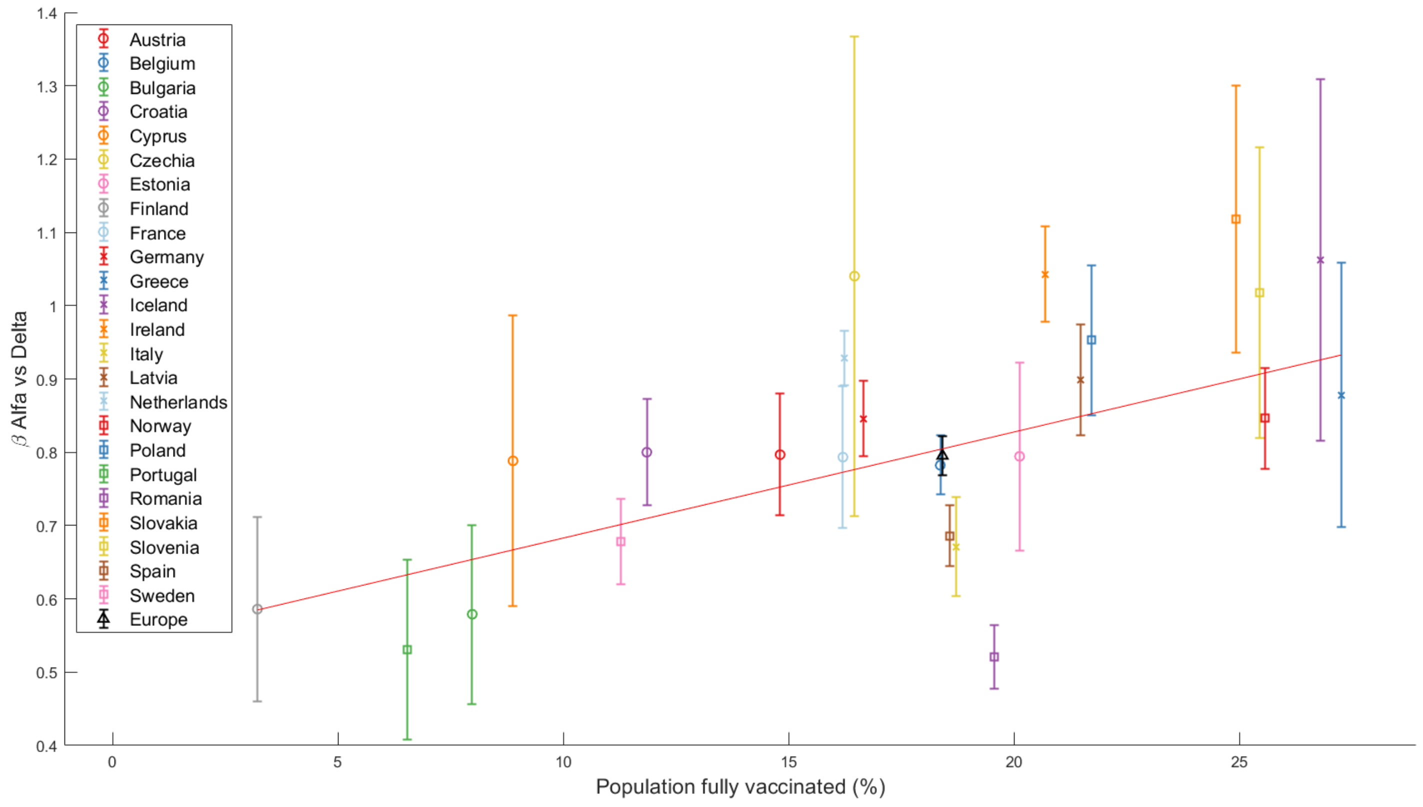

Figure S99 The increase in transmissibility $\Delta\beta$ is plotted against the percentage of fully vaccinated individuals at the beginning of the Alpha-Delta substitution ($Delta > 5\%$) with GISAID+TESSy databases. The red line marks the linear regression, clearly indicating an increasing trend.

The following and final images display the results for the effective reproduction number. In these, the average Rt is calculated when the variant begins to increase its number relative to the rest of the variants in a country, and the average when it is already above all others. Figure S100 to Figure S102, comparable to Figure 7 in the main article, display three diagrams with the position of this final / initial Rt with respect to the variant substitution.

As we already discussed in section 3.2.5 of the main article, we would expect the points to be situated above the diagonal line, which delimits a 1/1 ratio, indicating that cases increase when the new variant begins to dominate over the rest. As we can see in the figures, this holds true in most cases, although the result is slightly worse than with only the GISAID database.

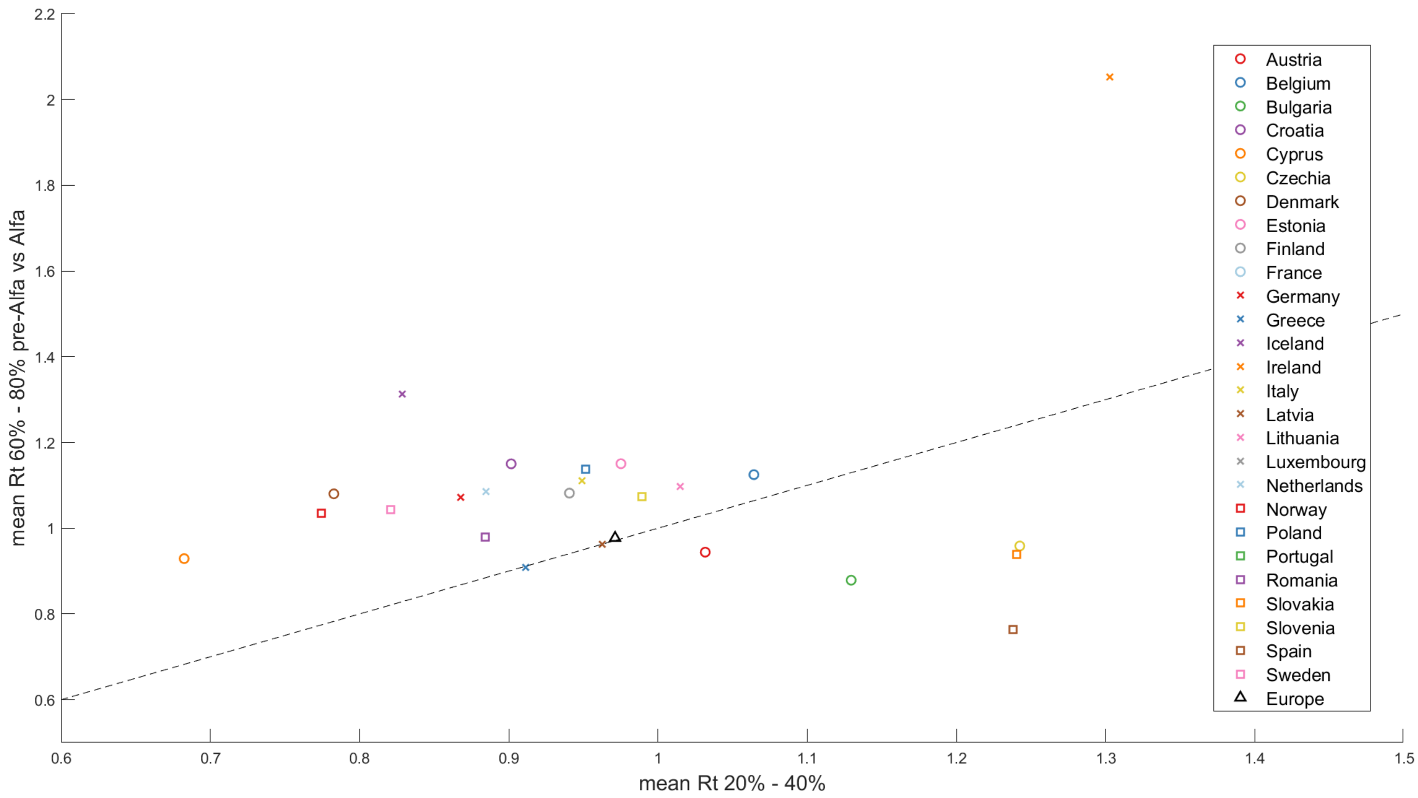

Figure S100 Effective reproduction number mean ($\overline{R_t}$) at the end of substitution (accounting for 60%-80% of the emerging variant) versus $\overline{R_t}$ at the beginning of the substitution (20%-40% of total). Results obtained for Alpha substitution and GISAID + TESSy databases.

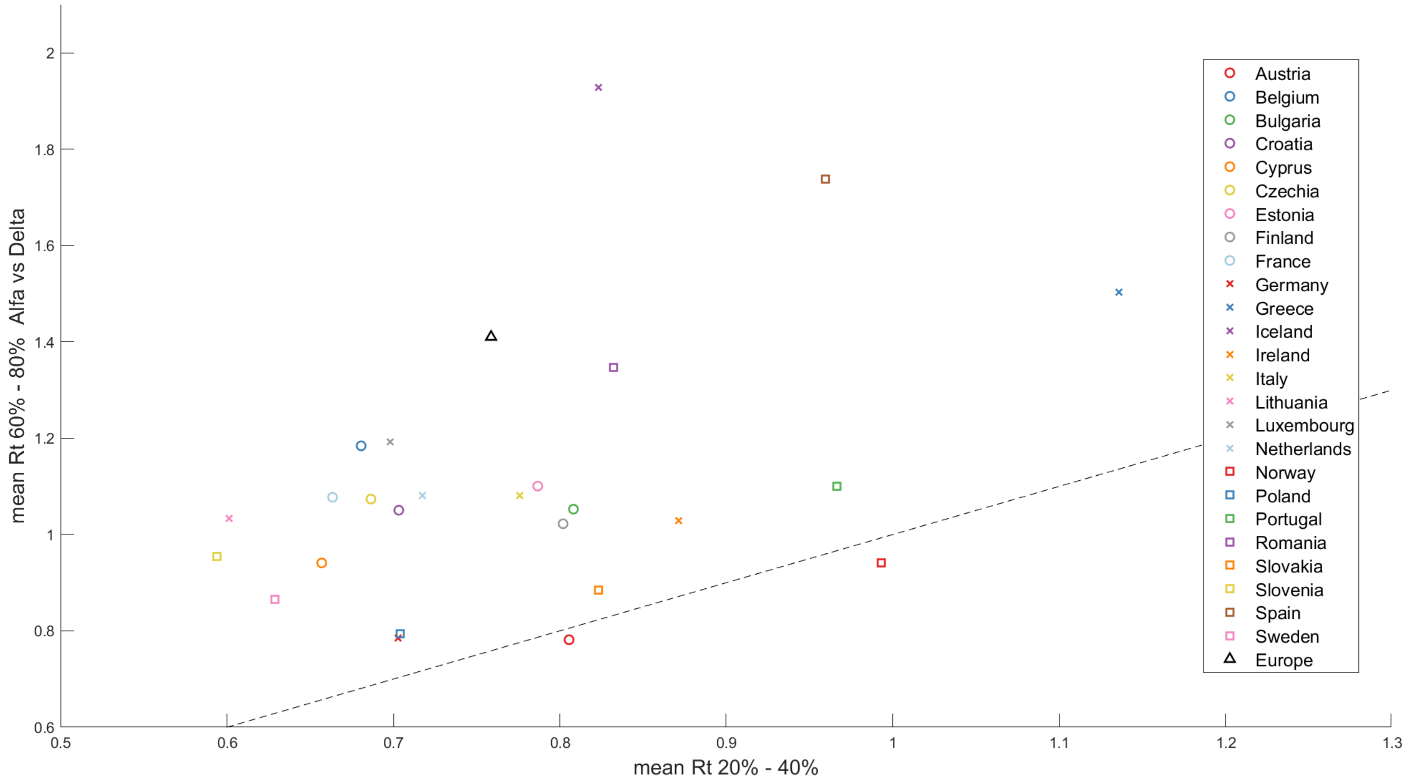

Figure S101 Effective reproduction number mean ($\overline{R_t}$) at the end of substitution (accounting for 60%-80% of the emerging variant) versus $\overline{R_t}$ at the beginning of the substitution (20%-40% of total). Results obtained for Delta substitution and GISAID + TESSy databases.

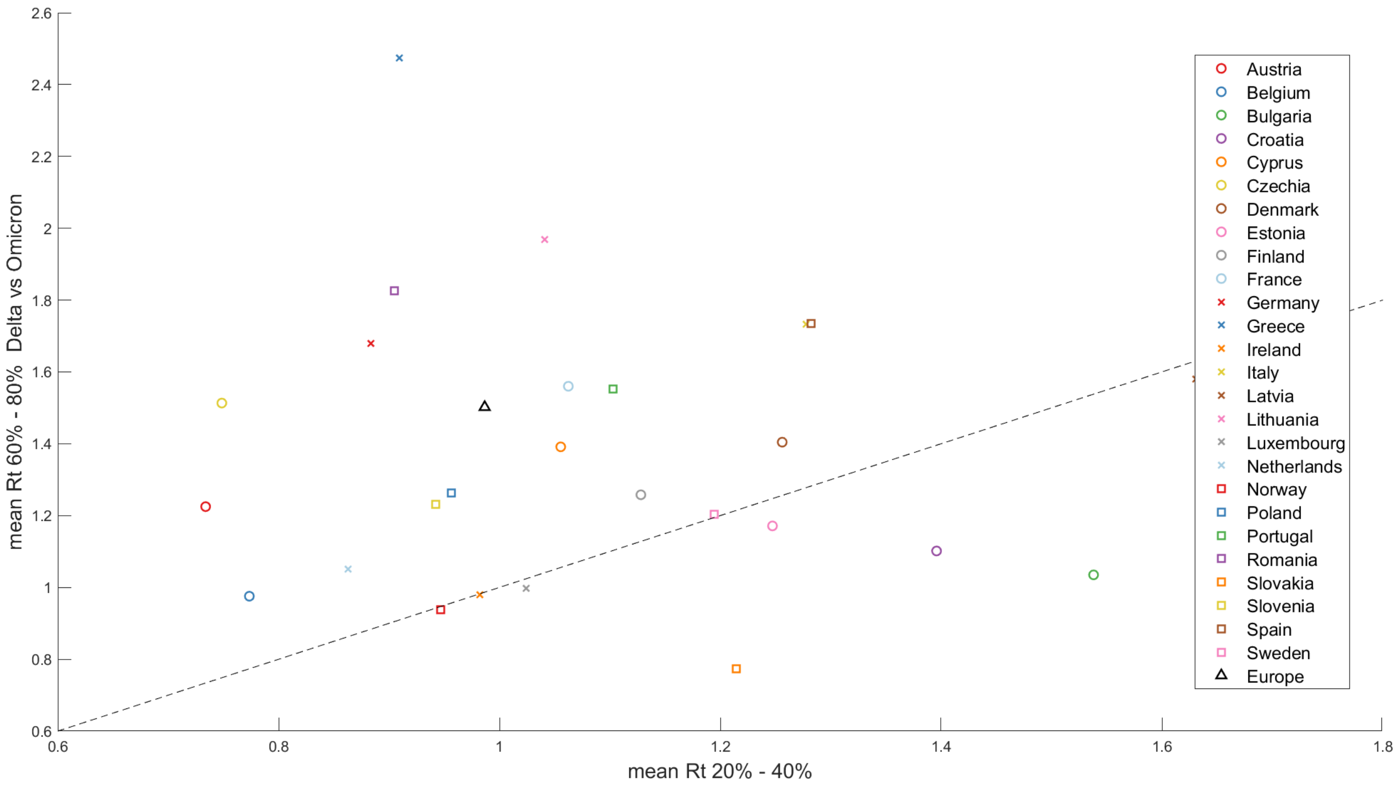

Figure S102 Effective reproduction number mean ($\overline{R_t}$) at the end of substitution (accounting for 60%-80% of the emerging variant) versus $\overline{R_t}$ at the beginning of the substitution (20%-40% of total). Results obtained for Omicron substitution and GISAID + TESSy databases.

Suppl. Material Text S12 – Transmissibility analysis in the context of two-variant problem

Following the same scheme as in the previous Suppl. Material Text S11, this last section presents the results obtained in the case of running the simulations with only two variants: the one that was dominant and the one that will be.

In the main article, along with the information and figures in Suppl. Material Text S7 ,we have already highlighted, that it is important to understand the difference in transmissibility between the main variants in order to be able to compare them directly. The results of Δβ with two variants are very similar to those obtained in the main article with the set of all the variants at play, so the conclusions are again the same as in the article. Simulating variant substitutions with all of them, as we have done in the main article, has the advantage of being able to estimate the daily percentage of each variant, and we believe that this is a very good feature if we are studying the evolution and dynamics of COVID-19 in Europe. For this reason, the main article is written based on the results of all the variants at play, while this Supplementary Material section simply aims to demonstrate that the same conclusions can be reached if we run the competition only between the variants that dominate at some point.

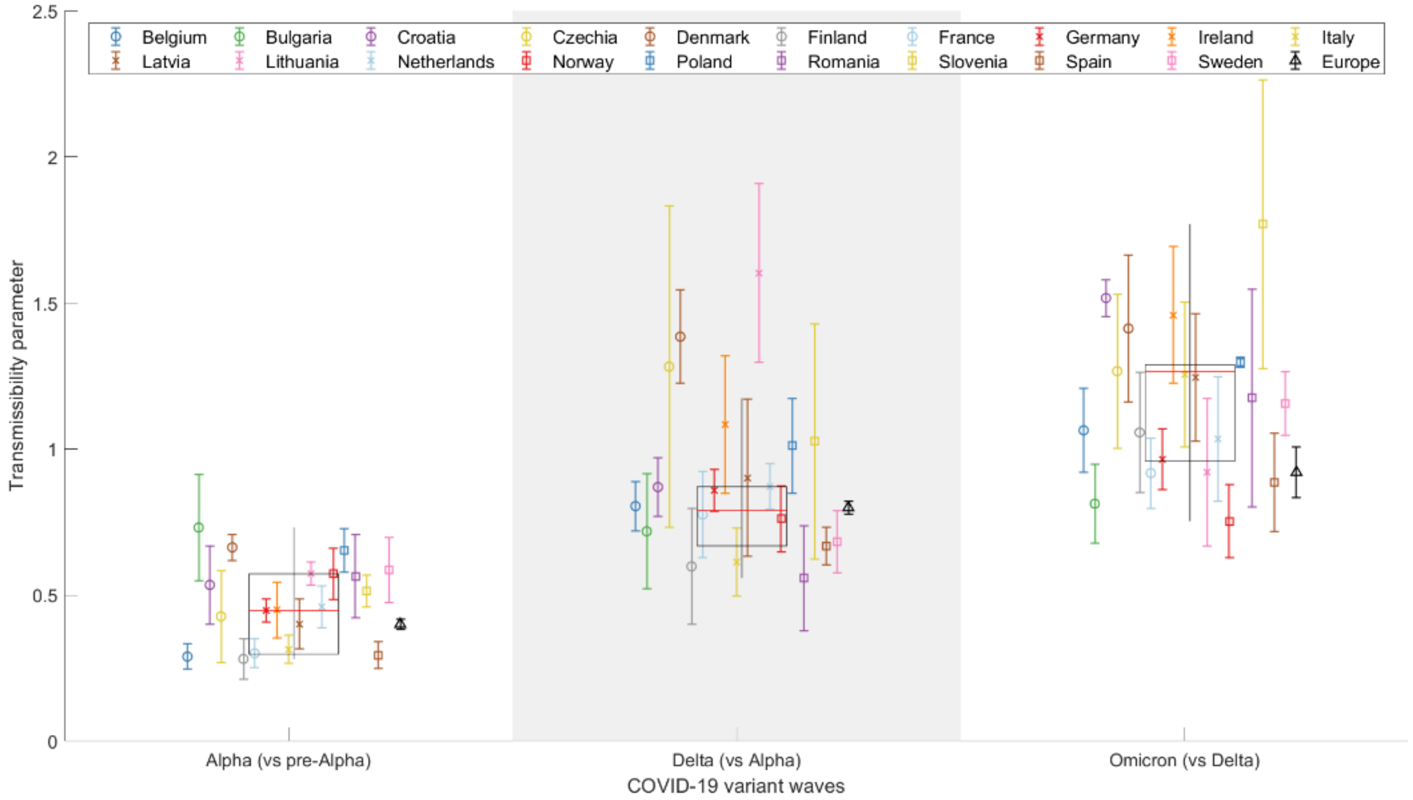

Figure S103 Representation of the increase in transmissibility parameter ($\Delta\beta$) for 19 European countries (circles, crosses, and squares) and Europe (black triangle) as a combination of the studied countries, based on the results for the two-variant substitutions Alpha, Delta, and Omicron. The x-axis simply distinguishes the three COVID-19 variant substitutions, while the y-axis displays the increment on the increase in transmissibility for each country.

The results of the evolution in each country for this competition between two variants can be found in Figures S41-S60 of the Supplementary Material Text 7. Although these figures show all six substitutions, in this section we will restrict ourselves to the three main substitutions, Alpha, Delta, and Omicron, following the same pattern as the article and the previous section. Below are the figures of $\Delta\beta$ as a function of the day of entry, the size of the country, the state of vaccination, and the relationship between Rt, with brief comments for the competition between two variants.

Figure S102 shows the evolution of $\Delta\beta$ in the three major substitutions. As before, it holds that $\Delta\beta_{\text{Alpha}} < \Delta\beta_{\text{Delta}} < \Delta\beta_{\text{Omicron}}$ and the numbers are very similar to those found in Figure 3. Table S5 shows the comparison with Europe and the average of all countries. The only exception is due to the Delta substitution, which, as happened in the main article, has a very small difference between Europe and the average of the countries, this time being slightly above.

If we look at the box plots of each substitution, we will see that this small discrepancy is due, again, to countries that are very far from acceptable intervals. In this case of competition of two variants, the second substitution (alpha vs delta) three countries will not be taken into account for the following studies: Czech Republic, Denmark, and Lithuania

Table S5 Results for Europe and for the average of all countries for all-variant and two-variant problem. First two rows are the same values than in Table 1.

	Alpha	Delta	Omicron
**Europe**	0.4041	0.7821	0.9114
**Average**	0.4477	0.7863	1.177
**Europe (2-variant)**	0.4005	0.7995	0.9205
**Average (2-variant)**	0.4464	0.7896	1.265

Figure S103 shows the study of $\Delta\beta$ with respect to the entry day of the new variant. This figure captures the same idea as Figure 4 of the main article, but now the result is only considering two variants (see Table S6), the one that dominated and the one that will dominate. The images are very similar and the conclusions are the same: the later a variant enters, the greater the increase in transmissibility.

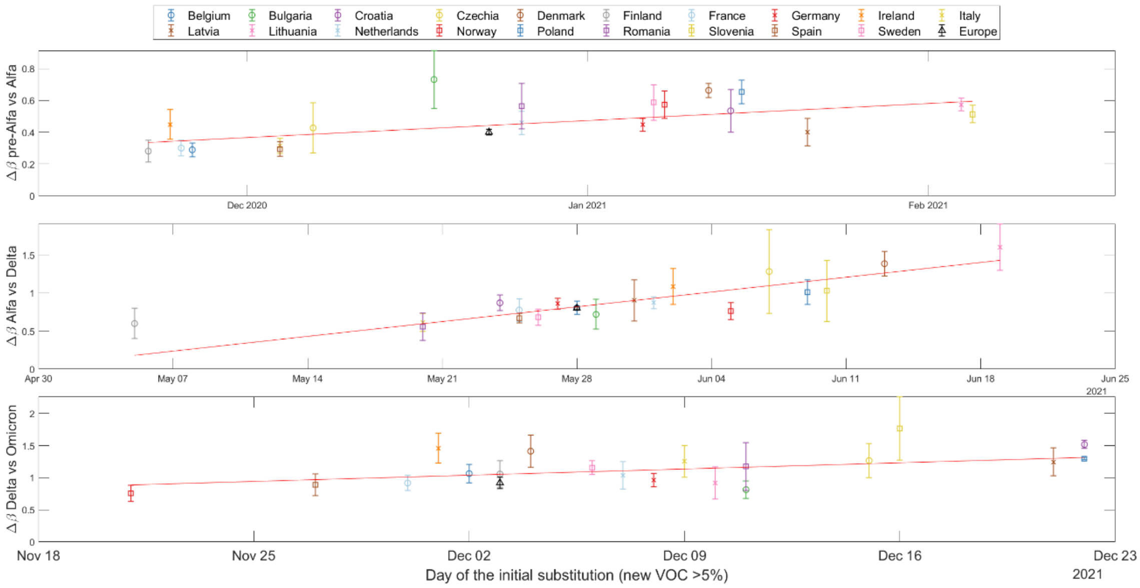

Figure S104 The increase in transmissibility ($\Delta\beta$) based on the day the emerging variant (Alpha on top, Delta in the middle, and Omicron at the bottom) exceeded 5% according to our 2-variant substitution model.

Table S6 Statistical results for trend analysis across three lineage transitions for all-variant (Table 2) and two-variant problem.

	**Alpha**	**Delta**	**Omicron**
$\rho_{5\%}$	0.609	0.804	0.523
p-value	0.005	$\sim 10^{-5}$	0.022
$\mathbf{R^2}$	0.6	0.67	0.5
$\rho_{5\%}$ **(2-variant)**	0.614	0.808	0.530
p-value **(2-variant)**	0.005	$\sim 10^{-5}$	0.019
$\mathbf{R^2}$ **(2-variant)**	0.55	0.63	0.5

The study of Δβ as a function of the country's area is represented in Figure S104, which is very similar to the one already shown in the main article, Figure 5. The results and conclusions are immediate: larger countries tend to have a smaller increase in transmissibility.

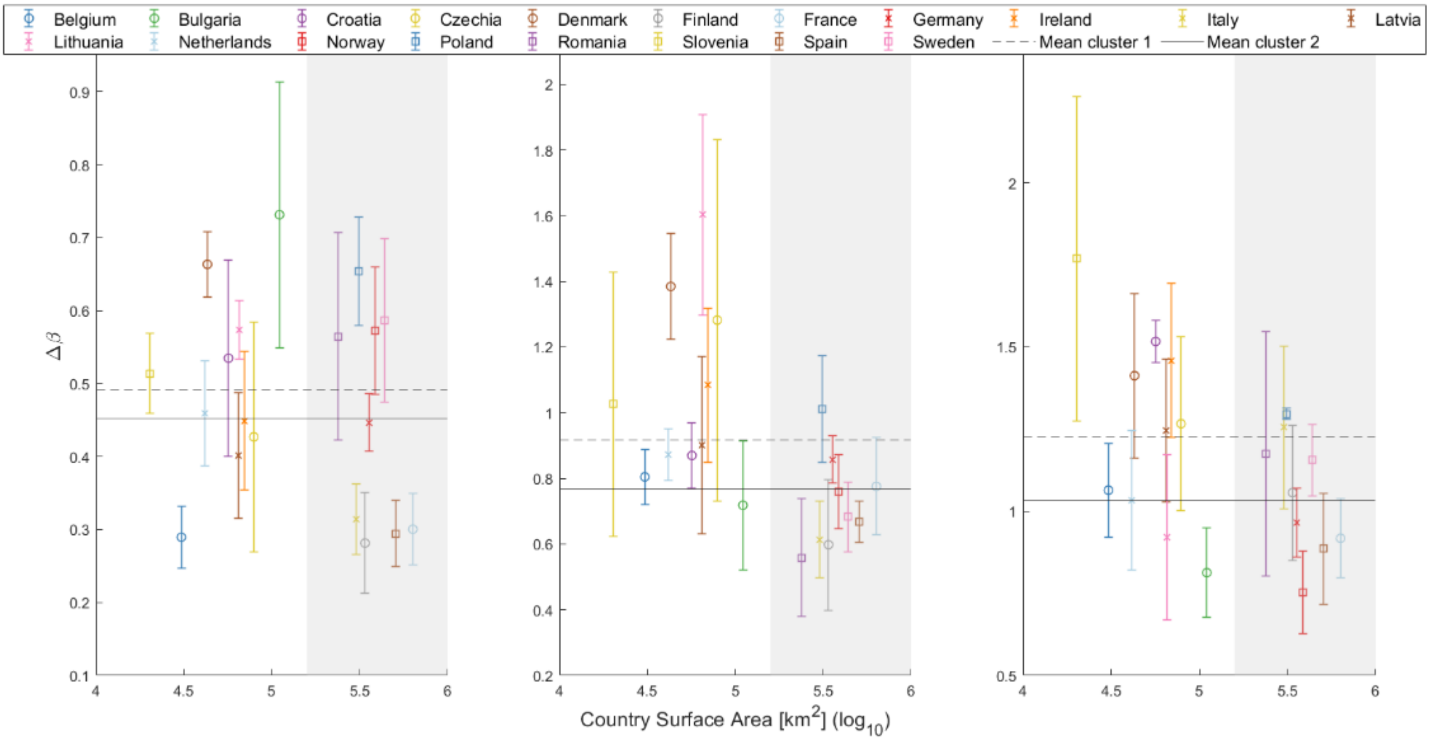

Figure S105 Increase in transmissibility ($\Delta\beta$) from two-variant analysis plotted against the log of the country's surface area, distinguishing two primary clusters: smaller countries (cluster 1) on a white background, and larger countries (cluster 2) on a gray background. Mean $\Delta\beta$ for both clusters are depicted in each substitution with horizontal lines (cluster 1: Dashed line; cluster 2: Solid line

The Δβ parameter again shows the same behavior with respect to vaccination. A relationship between them is only observed for the substitution from Alpha to Delta, Figure S105. The reasons have already been explained in section 3.2.4, in the article's discussion (section 4) and in Suppl. Material Text S10 . In this case, a $R^2 = 0.48$ and a Spearman's test coefficient of 0.55439 with a p-value = 0.015 < 0.05, indicating a clear correlation between the vaccination status and the increase in transmissibility.

Finally, the relationship between the effective reproduction number at the start of the new variant's entry and at the end of its rise (when it already dominates the entire national viral panorama), Figure S106, is very similar to the one seen in Figure 7, indicating that infections are much more numerous and the epidemic spreads more with the appearance of new variants.

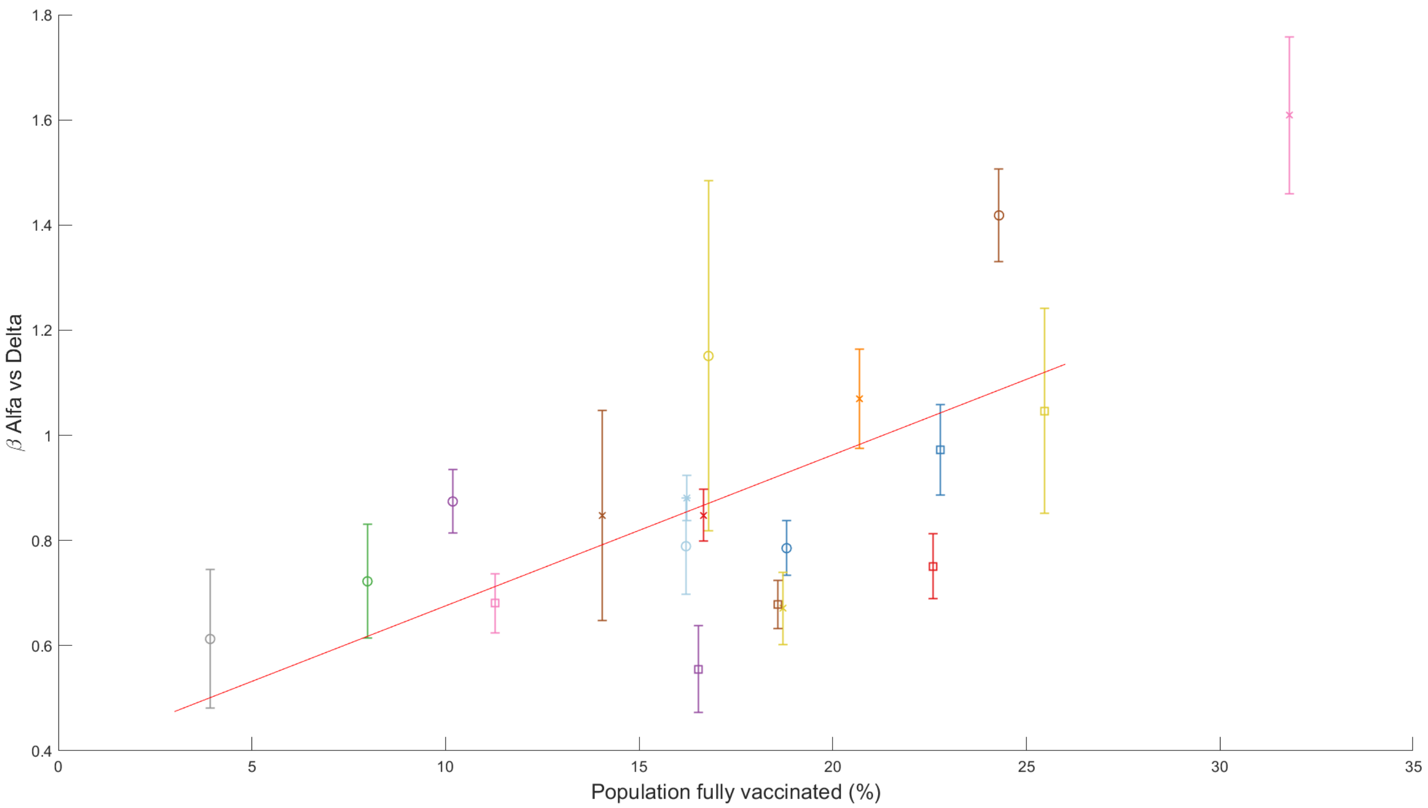

Figure S106 The increase in transmissibility $\Delta\beta$ for the two-variant simulation is plotted against the percentage of fully vaccinated individuals at the beginning of the Alpha-Delta substitution (Delta >5%). The red line marks the linear regression, clearly indicating an increasing trend.

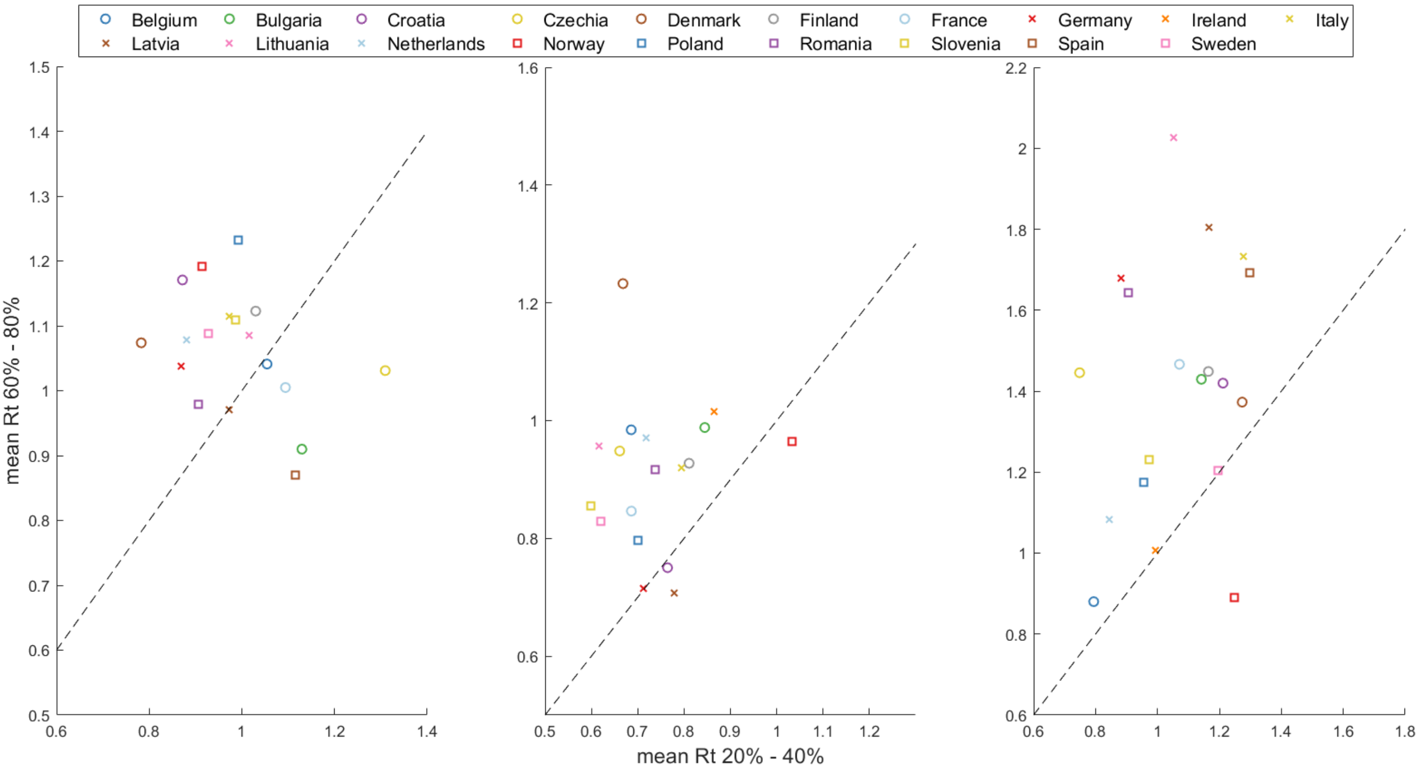

Figure S107 Effective reproduction number mean ($\overline{R_t}$) at the end of substitution (accounting for 60%-80% of the emerging variant) versus $\overline{R_t}$ at the beginning of the substitution (20%-40% of total). The dashed line indicates the $\frac{1}{1}$ threshold, where points above signify an aggravated pandemic state at the end of substitution, and points below suggest a less severe state upon introduction of a new variant.

Suppl. Material Text S13 – Extensive analysis of variant competitiveness (BQ.1 and XBB) across European countries

Here we present an expansion of Figure 9 from the main manuscript, extending the analysis to the 17 countries, covering only the last lineage substitution where the SARS-CoV-2 variants BQ.1 and XBB.1.5 are competing strongly.

Numerical results of the increase in transmissibility can be found in Table 3 of the main text.

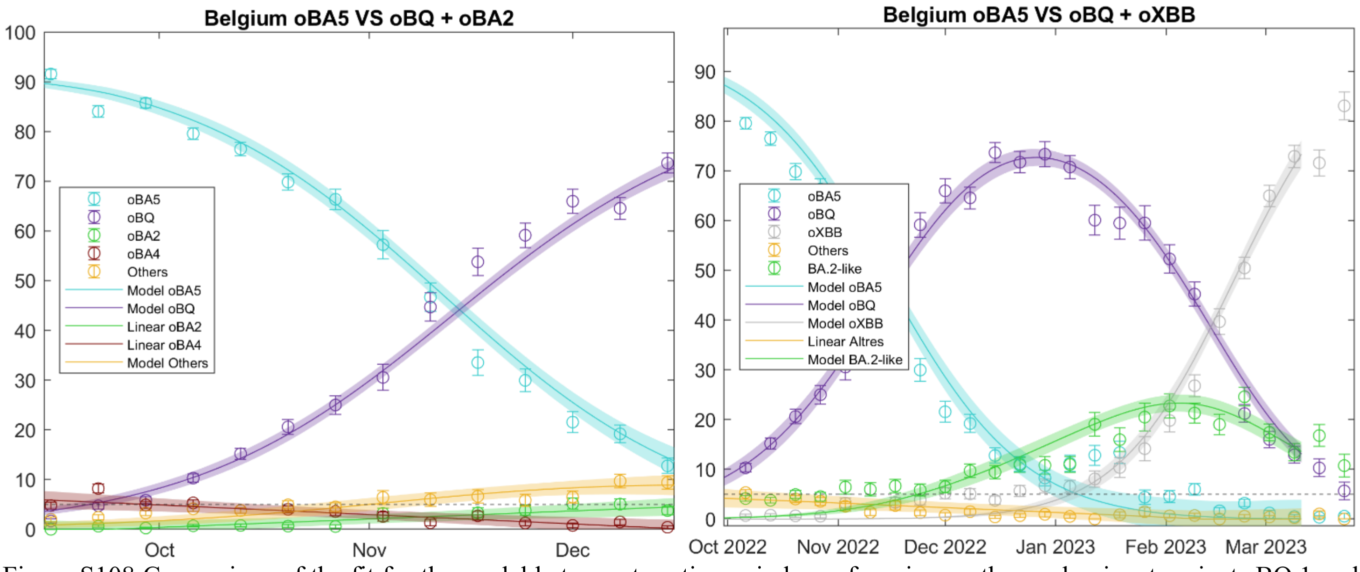

Figure S108 Comparison of the fit for the model between two-time windows, focusing on the predominant variants BQ.1 and XB for Belgium.

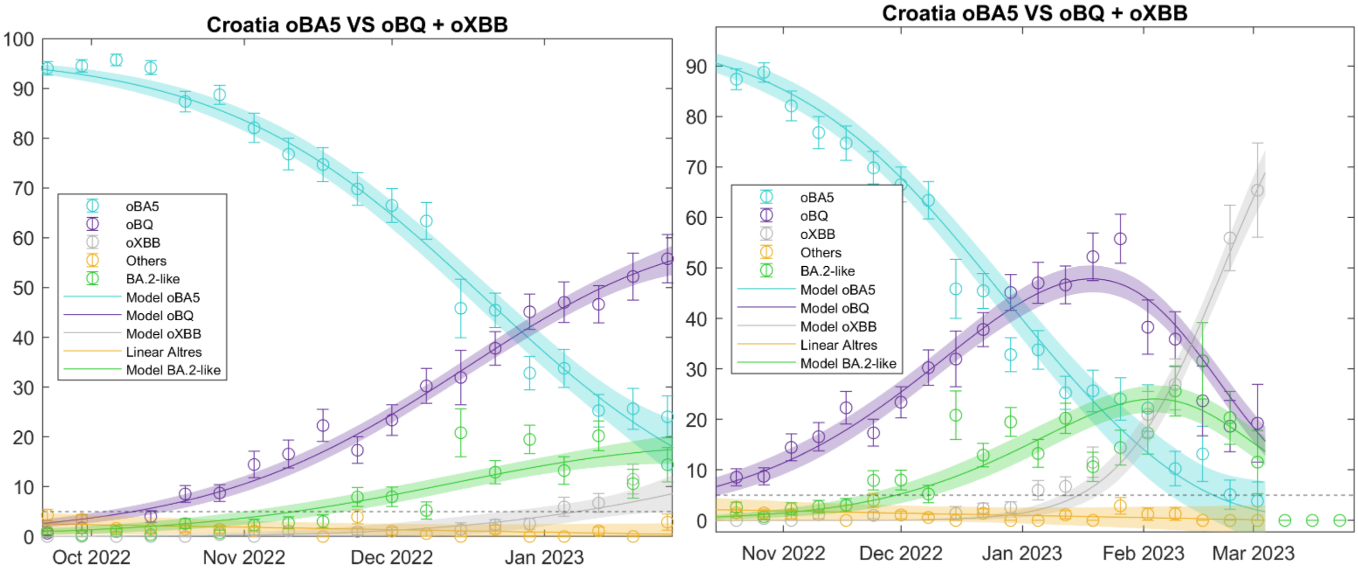

Figure S109 Comparison of the fit for the model between two-time windows, focusing on the predominant variants BQ.1 and XB for Croatia.

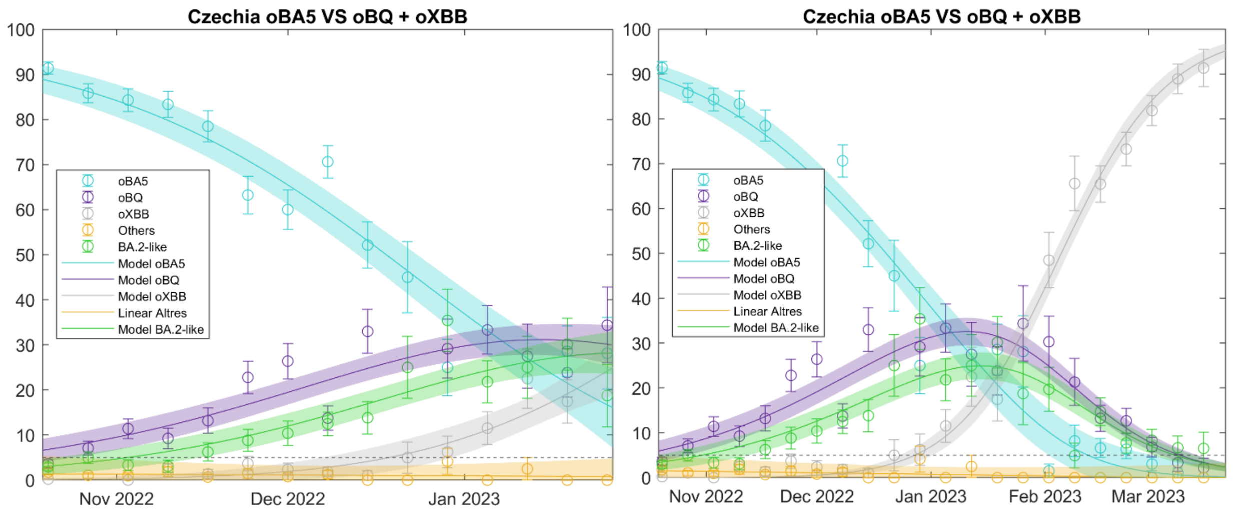

Figure S110 Comparison of the fit for the model between two-time windows, focusing on the predominant variants BQ.1 and XB for Czechia.

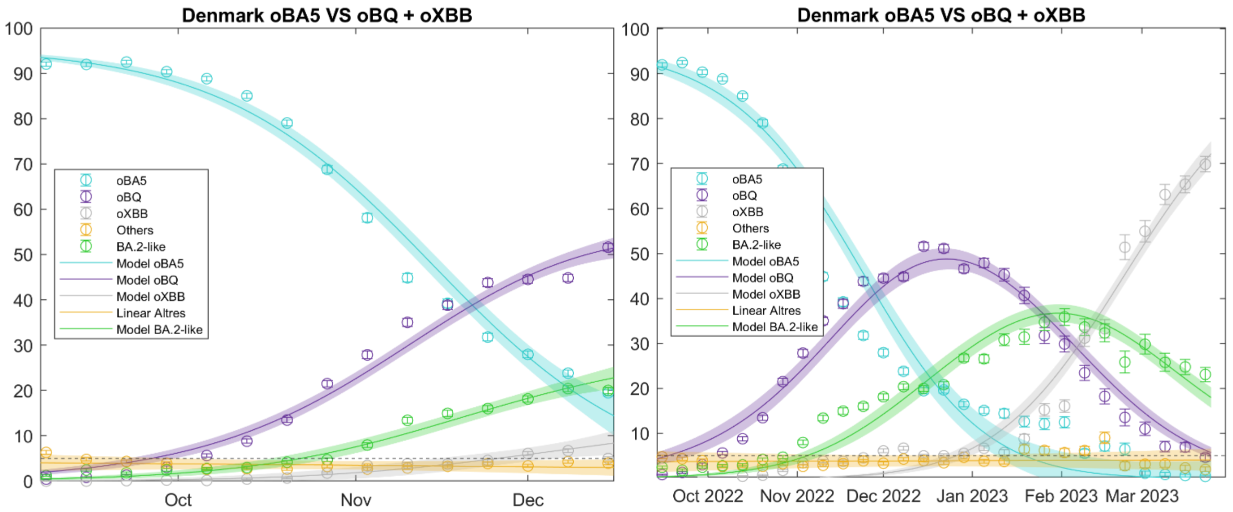

Figure S111 Comparison of the fit for the model between two-time windows, focusing on the predominant variants BQ.1 and XB for Denmark.

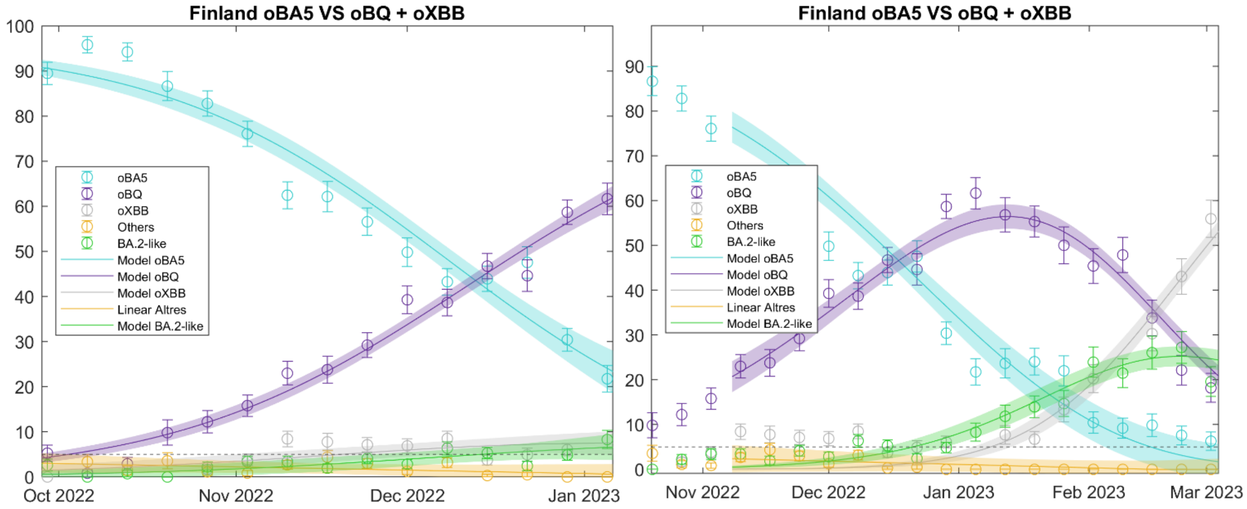

Figure S112 Comparison of the fit for the model between two-time windows, focusing on the predominant variants BQ.1 and XB for Finland.

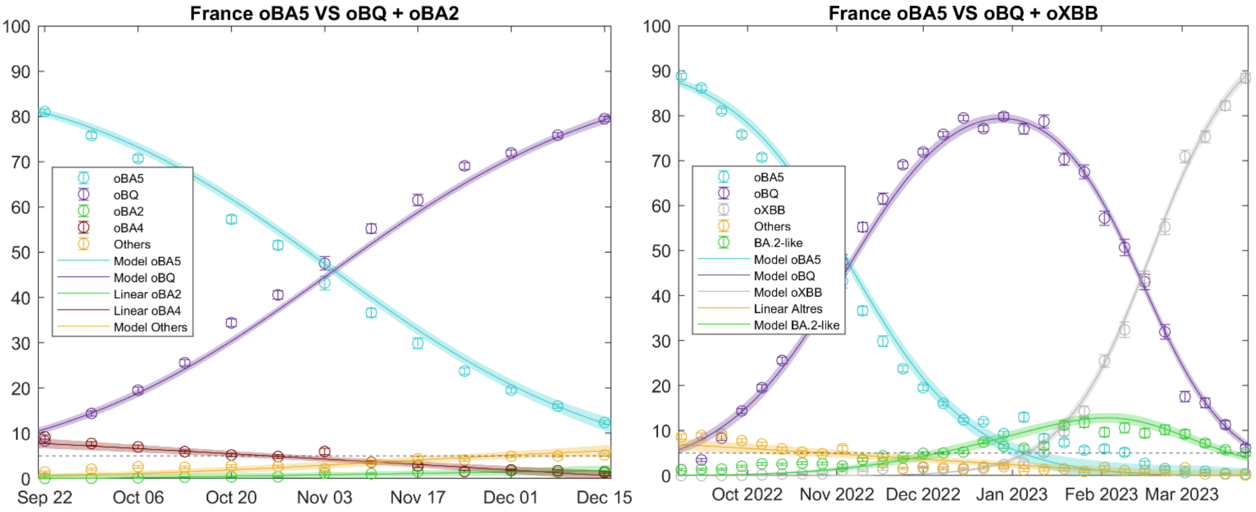

Figure S113 Comparison of the fit for the model between two-time windows, focusing on the predominant variants BQ.1 and XB for France.

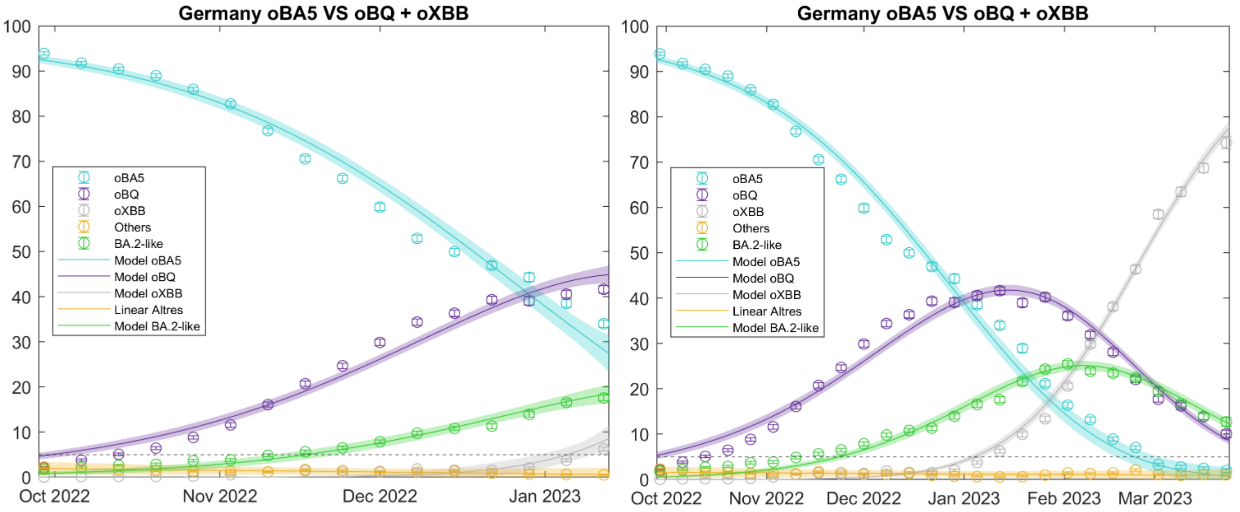

Figure S114 Comparison of the fit for the model between two-time windows, focusing on the predominant variants BQ.1 and XB for Germany.

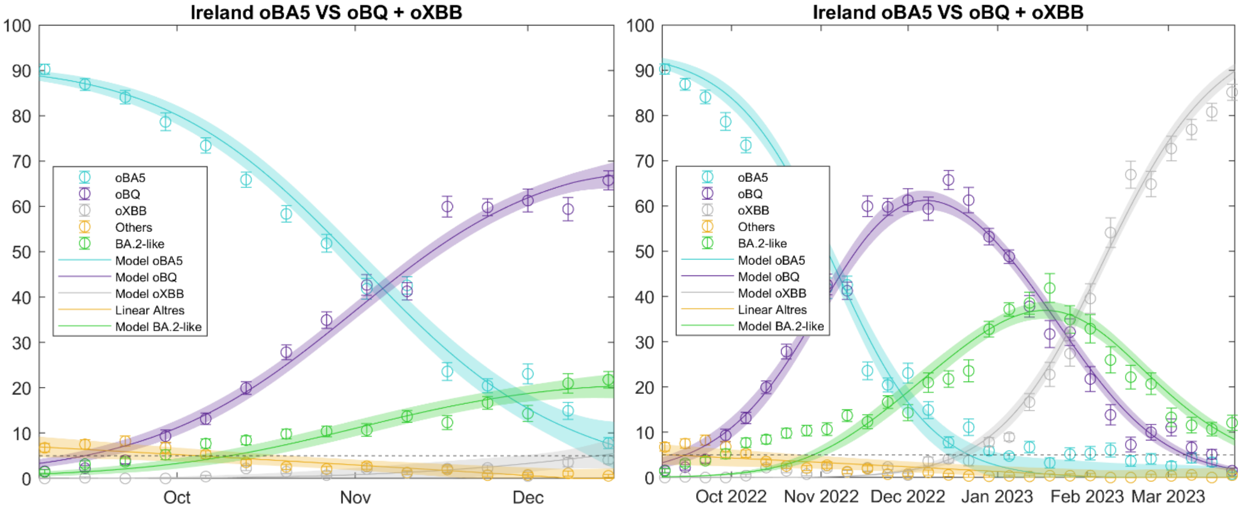

Figure S115 Comparison of the fit for the model between two-time windows, focusing on the predominant variants BQ.1 and XB for Ireland.

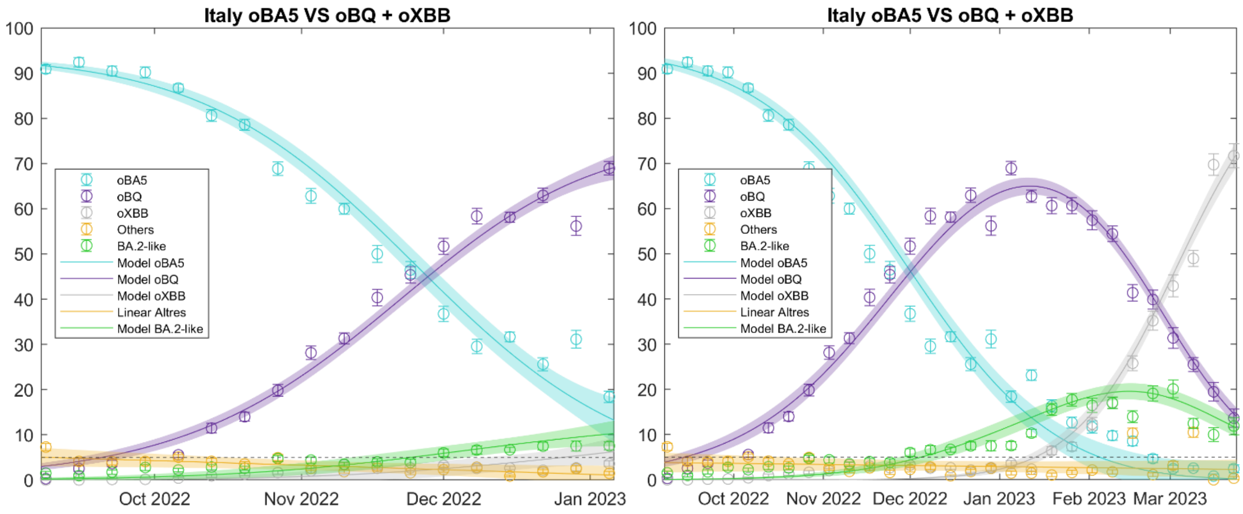

Figure S116 Comparison of the fit for the model between two-time windows, focusing on the predominant variants BQ.1 and XB for Italy.

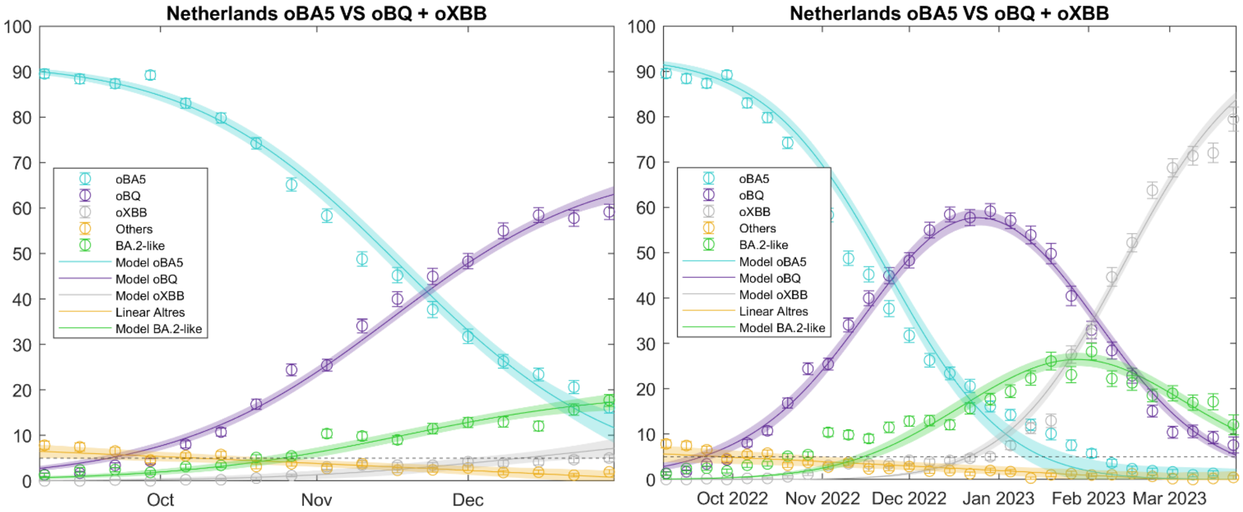

Figure S117 Comparison of the fit for the model between two-time windows, focusing on the predominant variants BQ.1 and XB for Netherlands.

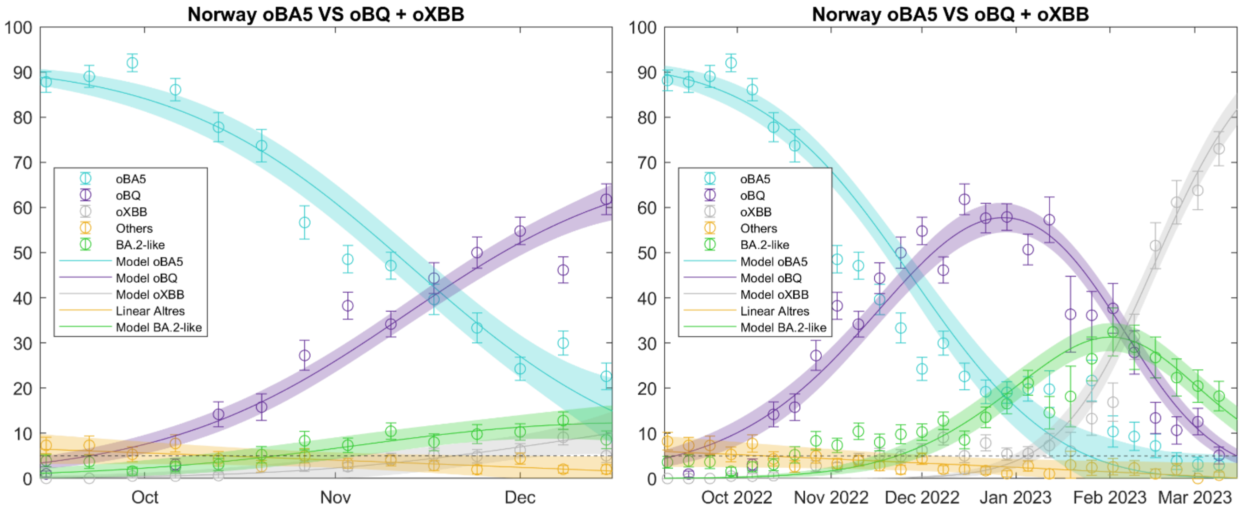

Figure S118 Comparison of the fit for the model between two-time windows, focusing on the predominant variants BQ.1 and XB for Norway.

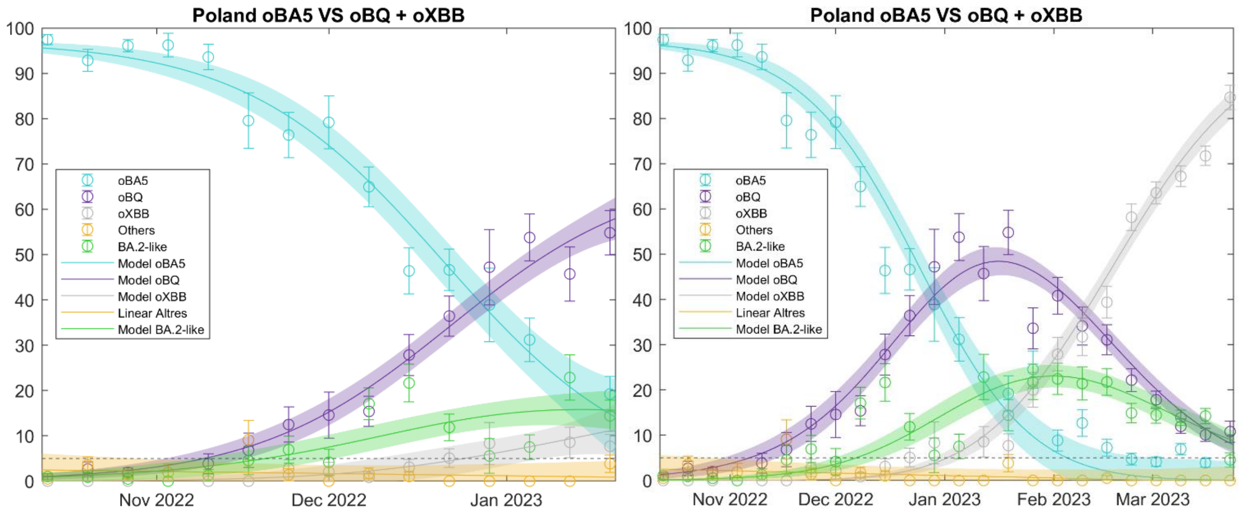

Figure S119 Comparison of the fit for the model between two-time windows, focusing on the predominant variants BQ.1 and XB for Poland.

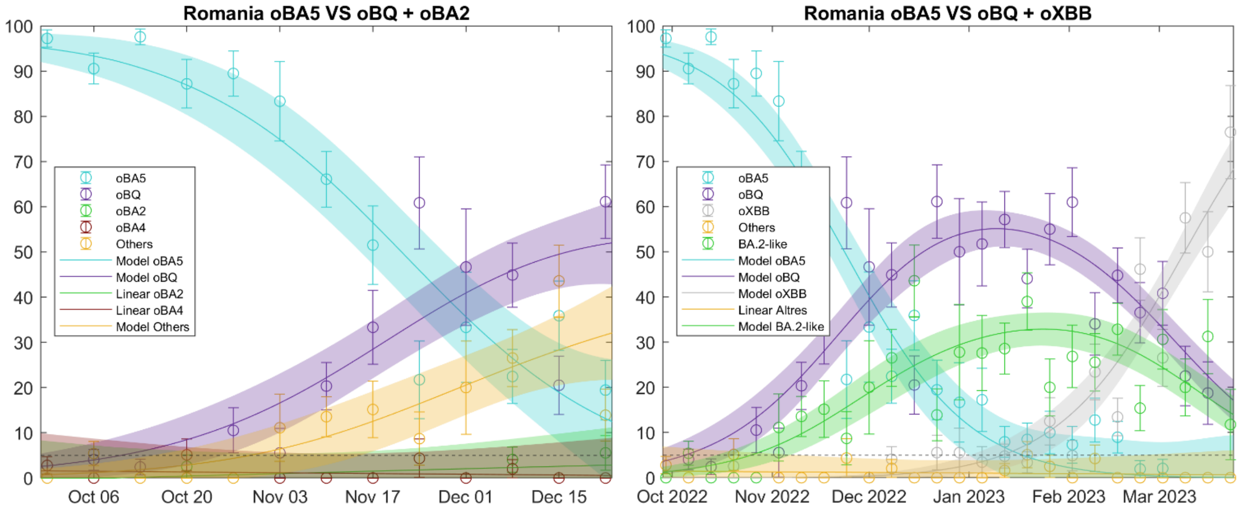

Figure S120 Comparison of the fit for the model between two-time windows, focusing on the predominant variants BQ.1 and XB for Romania.

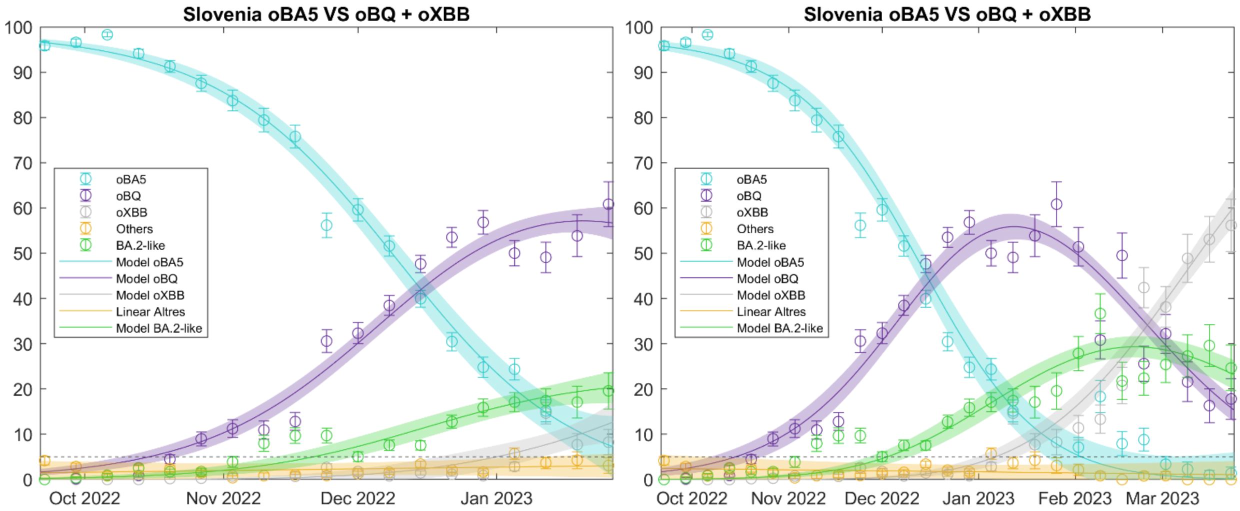

Figure S121 Comparison of the fit for the model between two-time windows, focusing on the predominant variants BQ.1 and XB for Slovenia.

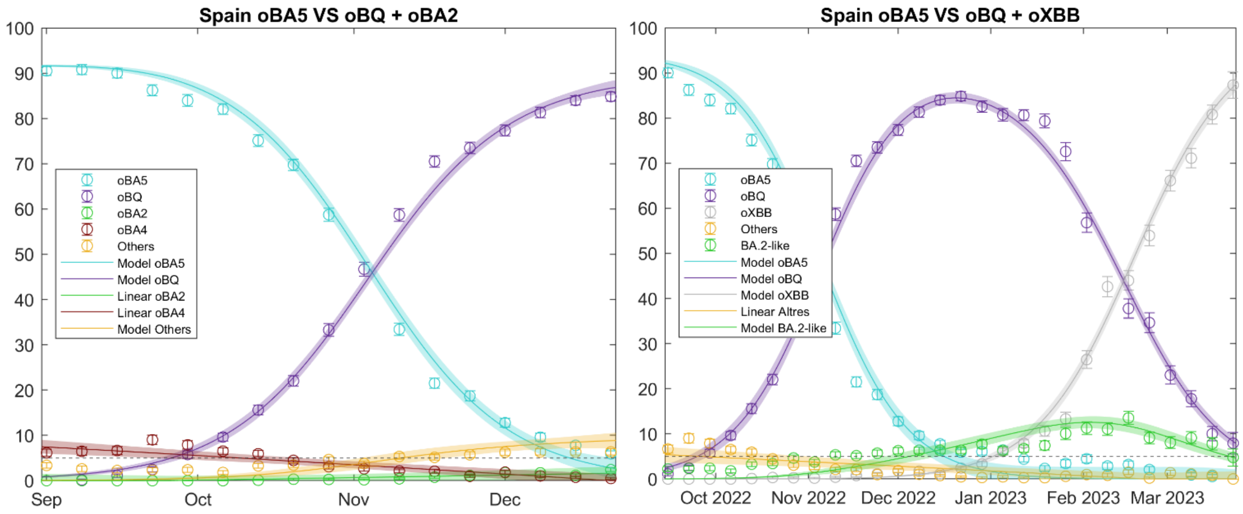

Figure S122 Comparison of the fit for the model between two-time windows, focusing on the predominant variants BQ.1 and XB for Spain.

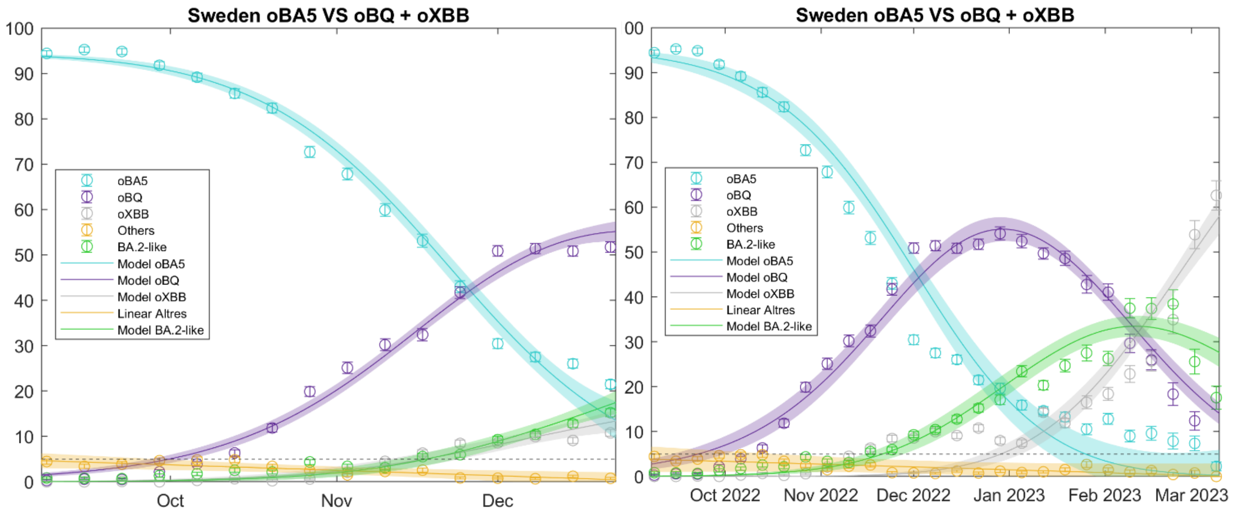

Figure S123 Comparison of the fit for the model between two-time windows, focusing on the predominant variants BQ.1 and XB for Sweden.

Supplementary Tables

Different spreadsheets with information are collected as:

Table 1a	Suppl_Mat_Table_1a_GISAID_data.xlsx	
Table 1b	Suppl_Mat_Table_1b_TESSy_data.xlsx	
Tables 2-9	Suppl_Mat_Tables_2-9.xlsx	
	Table 2:	GISAID vs TESSy (ECDC)
	Table 3:	Percentage GISAID per Country
	Table 4:	New cases & deaths
	Table 5:	Δβ Figure 1
	Table 6:	Δβ Figure 2
	Table 7:	Δβ Figure 3
	Table 8:	Different approaches
	Table 9:	All Δβ Figure 1
